# Cross-species insights from ART-D to uncover evolutionarily conserved oncogenic mechanisms

**DOI:** 10.1038/s44320-026-00215-8

**Published:** 2026-05-14

**Authors:** Yifan Guo, Jiadong Zheng, Yixin Wu, Kun Zhao, Dian Lv, Wenhan Liu, Mengling Wang, Jin-Yu Lu, Wenyan Xu, Xianping Wang, Xianjue Ma

**Affiliations:** 1 Fudan University, Shanghai, China; 2 State Key Laboratory of Gene Expression, School of Life Sciences, Westlake University, Hangzhou, China; 3 Westlake Laboratory of Life Sciences and Biomedicine, Hangzhou, China; 4 Department of Oncology, Montefiore Einstein Comprehensive Cancer Center, Albert Einstein College of Medicine, Bronx, NY USA

**Keywords:** Cancer, Signal Transduction

## Abstract

Cancer arises from oncogenic clones, yet the dynamic mechanisms driving their stepwise evolution toward malignancy remain incompletely understood. Here, we establish the Atlas of *Ras*-driven Tumors in *Drosophila* (ART-D), a systematic, cross-species platform that dissects the molecular and phenotypic trajectories of tumorigenesis across ten genetically defined *Ras*^*V12*^-driven models. By integrating longitudinal phenotypic profiling, we define three conserved stages of tumor development—initiation, promotion, and progression—distinguished by distinct shifts in tumor burden and tumor-induced cachexia. Transcriptomic analysis reveals stage-specific signaling rewiring: early tumorigenesis is characterized by co-activation of JAK/STAT, NF-κB/Toll, and MAPK pathways, whereas malignant progression is driven by Notch hyperactivation and Hippo pathway inactivation. Through integrative multi-omics and machine learning, we uncover an evolutionarily conserved pathogenic network coordinating JNK, NF-κB/Toll, Notch, and Hippo signaling, which we functionally validate across species. ART-D serves as a transformative resource bridging *Drosophila* genetics and human cancer biology, offering a robust framework for decoding conserved oncogenic principles and identifying of stage-specific vulnerabilities in *RAS*-driven cancers.

## Synopsis

Integrating *Drosophila* genetics with multimodal omics, the ART-D framework defines stage-specific paradigms of *Ras*-driven tumorigenesis and reveals evolutionarily conserved oncogenic signaling mechanisms in human *KRAS*/*NRAS*-driven cancers.

- Construction of the *Drosophila* Atlas of *Ras*-related Tumors (ART-D), a quantitative framework that defines stage-specific phenotypic progression features.
- Multimodal omics data analyses on ART-D uncovers a biphasic signaling paradigm governing *Ras*-driven tumorigenesis.
- Cross-species investigations reveal conserved synergistic interactions among pro-tumorigenic signaling pathways (JNK, Notch, NF-κB/Toll, and Hippo).
- Machine learning models validate the clinical translational utility of *Drosophila* models derived *Ras* tumor signatures.

## Abstract (web summary)

Integrating *Drosophila* genetics with multimodal omics, the ART-D framework defines stage-specific paradigms of *Ras*-driven tumorigenesis and reveals evolutionarily conserved oncogenic signaling mechanisms in human *KRAS*/*NRAS*-driven cancers.

## Graphical abstract

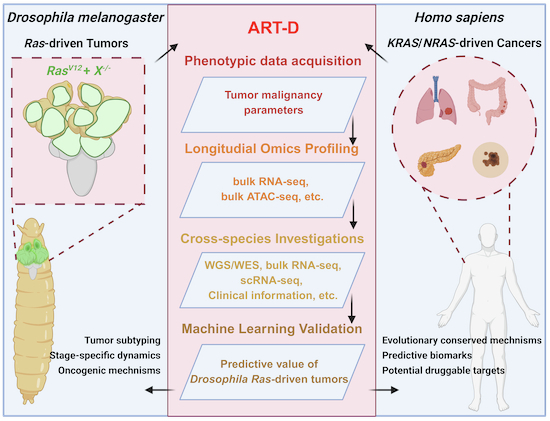

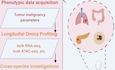

## Introduction

Cancer initiation is driven by oncogenic mutations that propel clonal evolution (Greaves and Maley, 2012; Greaves, 2015; Vendramin et al, 2021; Zhang et al, 2024), with *RAS* proto-oncogenes representing the most frequently mutated oncogene family across human malignancies (Pylayeva-Gupta et al, 2011; Prior et al, 2020; Johnson et al, 2022; Choucair et al, 2025). Recurrent mutations at codons 12, 13, and 61 lock RAS in a constitutively active state, leading to persistent downstream signaling and uncontrolled cellular proliferation (Prior et al, 2020; Mukhopadhyay et al, 2021; Johnson et al, 2022). Despite advances in mapping cancer genomes and identifying co-occurring or mutually exclusive mutations (e.g., *RAS-BRAF* exclusivity or *RAS-MET*/*ERBB2*-amplifications co-occurrence) (Stratton et al, 2009; Vogelstein et al, 2013; Pon and Marra, 2015; Scheffler et al, 2019; Tekle et al, 2021; Lee et al, 2022), our understanding of how RAS dynamically interfaces with tumor suppressor networks to orchestrate malignant transformation remains fundamentally limited. Current in vitro models often fail to recapitulate the spatiotemporal complexity of tumor evolution in vivo, particularly the dynamic transitions underlying clinical progression.

In this context, *Drosophila melanogaster* emerges as a uniquely powerful system for dissecting tumorigenesis. Compared to mammalian models such as *Mus musculus* or *Danio rerio*, the fruit fly offers unparalleled advantages in genetic tractability, rapid generation time, and scalability for high-throughput functional screening. Over the past decades, *Drosophila* research has been instrumental in defining foundational principles of cancer biology, from the discovery of hyperplastic/neoplastic tumor suppressors (Hariharan and Bilder, 2006), including core members of Hippo pathway (*hippo* [*hpo*] and *warts* [*wts*]) (Xu et al, 1995; Justice et al, 1995; Harvey et al, 2003; Wu et al, 2003; Jia et al, 2003; Pantalacci et al, 2003; Udan et al, 2003) and cell polarity genes (*scribble* [*scrib*], *discs large 1* [*dlg1*], and *lethal (2) giant larvae* [*l(2)gl*]) (Perrimon, 1988; Peng et al, 2000; Bilder and Perrimon, 2000; Bilder et al, 2000; Brumby, 2003), to the identification of pro-tumoral signals like c-Jun N-terminal kinase (JNK) and Notch (Kidd et al, 1983; Artavanis-Tsakonas et al, 1983; Riesgo-Escovar et al, 1996; Brumby and Richardson, 2005; Hariharan and Bilder, 2006; Wu et al, 2010). Combining oncogenic *Ras* (*Ras*^*V12*^) with specific cooperating mutations enables the faithful modeling of hallmark cancer phenotypes, including uncontrolled proliferation, tissue invasion, systemic cachexia, and therapy resistance (Brumby and Richardson, 2005; Sonoshita and Cagan, 2017; Enomoto et al, 2018). For instance, co-expression of *Ras*^*V12*^ with dysregulated *fiery mountain* (*fmt*), *emei* (*emei*), or *misshapen* (*msn*) activate JNK yet inactivate Hippo to promote tumorigenesis and invasiveness (Ma et al, 2017, 2020; Kong et al, 2021; Yang et al, 2023). Meanwhile, *Ras*^*V12*^ tumors with disrupted apical-basal polarity (e.g., *scrib* and *dlg*) secrete cachectic factors like Matrix metalloproteinase 1 (MMP1) and Ecdysone-inducible gene L2 (ImpL2) to remodel microenvironments and induce organism-wide wasting (Kwon et al, 2015; Figueroa-Clarevega and Bilder, 2015; Lodge et al, 2021; Liu et al, 2022). Despite these advances, a systematic, comparative framework for *RAS*-driven tumor models with diverse genetic backgrounds has been lacking. This gap has obscured the identification of conserved molecular trajectories and stage-specific vulnerabilities that could inform targeted therapeutic strategies.

A central challenge in cancer biology lies in decoding the molecular and phenotypic transitions that define tumor evolution—from initial clonal expansion to heterogeneous, invasive disease. This complexity hinders the translation of genotype into clinically meaningful outcomes (Zhang et al, 2024). Longitudinal analyses that integrate transcriptional dynamics with quantitative phenotypic readouts—such as tumor burden and systemic cachexia—are rare but essential for pinpointing critical intervention windows. To address this, we developed the Atlas of *Ras-*driven Tumors in *Drosophila* (ART-D), a cross-species platform encompassing ten genetically defined malignant models. By integrating deep phenotyping with longitudinal transcriptomics, we chart the conserved signaling trajectories that underlie tumorigenesis. Strikingly, multi-omics and machine learning analyses reveal that *Drosophila Ras*-driven tumors converge on a pathogenic network involving coordinated dysregulation of JNK, Notch, NF-κB/Toll, and Hippo pathways—a signature robustly mirrored in *KRAS*/*NRAS*-high human cancers. Our work establishes *Drosophila* as a dynamic, scalable model for dissecting the evolutionary trajectory of *RAS*-driven tumors and uncovers evolutionarily ancient, therapeutically targetable nodes in cancer progression.

## Results

### Tumor suppressors synergizing with *Ras*^*V12*^ to drive tumorigenesis

**Figure EV1:**
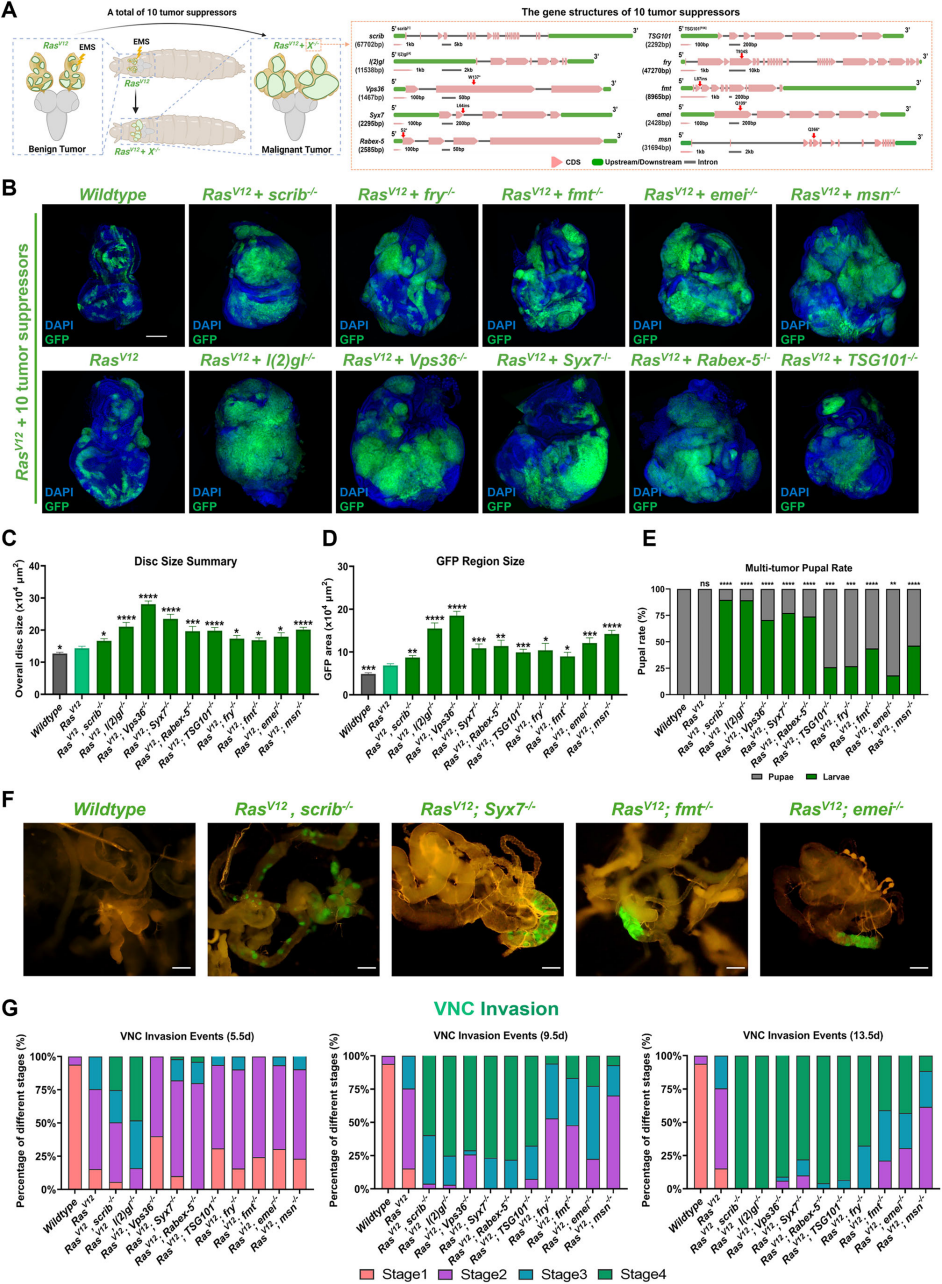
Ten *Drosophila Ras*-driven malignant tumor models. (**A**) The schematic diagram depicting the ethyl methanesulfonate (EMS) genetic screening of the *Drosophila* eye-antennal disc to identify novel tumor suppressors (*Vps36*, *Syx7*, *Rabex-5*, *TSG101*, *fry*, *fmt*, *emei*, and *msn*) on the *Drosophila* 3L chromosome, along with two well-known tumor suppressors (*scrib* and *l(2)gl*) that can synergize with oncogenic *Ras* (*Ras*^*V12*^) to drive malignant tumorigenesis. The mutations of ten tumor suppressors used in this study were denoted as indicated. The gene structure was drawn by using GSDS 2.0 (https://gsds.gao-lab.org/) with sequence downloaded from FlyBase (https://flybase.org/). (**B**) The malignant tumors driven by ten tumor suppressors in the context of *Ras* activation at 8 days AEL. Scale bar: 100 µm. (**C**) Quantification of the overall disc size of each tumor model at 8 days AEL. The *wildtype* and malignant tumor groups were compared to the *Ras* group by using Mann–Whitney *U* test, *n* > 30. Results were presented as means ± SEM. Significance of different genotypes were denoted as follows: ns >0.05, *P < 0.05, **P < 0.01, ***P < 0.001, ****P < 0.0001. For exact *P* values, in (**C**), from left to right, *P = 0.0323, *P = 0.0233, ****P < 0.0001, ****P < 0.0001, ****P < 0.0001, ***P = 0.0004, ****P < 0.0001, *P = 0.0135, *P = 0.0262, *P = 0.0193, ****P < 0.0001. (**D**) Quantification of the GFP region of each tumor model at 8 days AEL. The *wildtype* and malignant tumor groups were compared to the *Ras* group by using Mann–Whitney *U* test, *n* > 30. Results were presented as means ± SEM. Significance of different genotypes were denoted as follows: ns > 0.05, *P < 0.05, **P < 0.01, ***P < 0.001, ****P < 0.0001. For exact *P* values, in (**D**), from left to right, ***P = 0.0002, **P = 0.0083, ****P < 0.0001, ****P < 0.0001, ***P = 0.0010, **P = 0.0036, ***P = 0.0004, *P = 0.0147, *P = 0.0348, ***P = 0.0003, ****P < 0.0001. (**E**) Quantification of larval pupariation rate of ten malignant tumor models. The *Ras* group and malignant tumor groups were compared to *wildtype* group by using Chi-square test when the sample size was at least 40 (*n* ≥ 40) and the expected frequency in each cell was no less than 5 (*T* ≥ 5); otherwise, Fisher’s exact test was used. Significance of different genotypes were denoted as follows: ns >0.05, *P < 0.05, **P < 0.01, ***P < 0.001, ****P < 0.0001. For each group, *n* > 30. For exact *P* values, in (**E**), from left to right, ns *P* > 0.9999, ****P < 0.0001, ****P < 0.0001, ****P < 0.0001, ****P < 0.0001, ****P < 0.0001, ***P = 0.0001, ***P = 0.0001, ****P < 0.0001, **P = 0.0012, ****P < 0.0001. (**F**) The representative pictures of *Drosophila Ras*-driven tumors exhibiting aggressive intestinal metastasis at 8 days AEL. Scale bar: 200 µm. (**G**) The quantification of VNC invasion events of ten malignant tumor models at designated time points compared with *wildtype* discs and *Ras* benign tumors. The VNC invasion stage of collected samples were classified into four stages with the criterion shown in Fig. 1A.

We previously conducted a large-scale ethyl methanesulfonate (EMS)-induced forward genetic screen on *Drosophila* chromosome 3L using *ey*-FLP-based MACRM (mosaic analysis with repressible cell marker) system (Fig. EV1A) (Lee and Luo, 1999; Ma et al, 2017), with the aim of identifying novel tumor suppressors capable of synergizing with oncogenic *Ras* (*Ras*^*V12*^) to drive malignant tumors in the *Drosophila* eye-antennal disc (Lee and Luo, 1999; Ma et al, 2017, 2020; Kong et al, 2021). Among the candidate alleles identified, eight recessive-lethal alleles were characterized: *Vacuolar protein sorting 36* (*Vps36*^*#2586*^), *Syntaxin 7* (*Syx7*^*#3119*^), *Rabaptin-5-associated exchange factor for Rab5* (*Rabex-5*^*#3672*^), *Tumor susceptibility gene 101* (*TSG101*^*#3622*^; later replaced by *TSG101*^*P26*^ (Moberg et al, 2005) due to lethality), *furry* (*fry*^*#3792*^), *fmt*^*#3355*^(Ma et al, 2017), *emei*^*#566h*^ (Ma et al, 2020), and *msn*^*#3208*^ (Kong et al, 2021) (Fig. EV1A). Disruption of these genes through induction of homozygous mutant clones in *Ras*^*V12*^ cells led to enhanced tumor overgrowth and delayed pupariation of third instar larvae (Fig. EV1B–E). These phenotypic effects mirrored those observed in *Ras*^*V12*^ cells with disruptions in canonical cell polarity genes, such as *scrib*^*1*^ (Wu et al, 2010) and *l(2)gl*^*4*^ (Menéndez et al, 2010) (Fig. EV1B–E). Interestingly, by dissecting larvae bearing *Ras*-driven tumors, we also observed tumor metastasis in the intestinal tissues (Fig. EV1F), indicating that these *Ras*-driven tumors may possess the potential for further malignant progression. Thereby, summarizing these tumor suppressors, we obtained a cohort of ten *Drosophila Ras*-driven tumor (DRT) models.

### The Atlas of *Ras*-driven tumors in *Drosophila* (ART-D)

**Figure 1:**
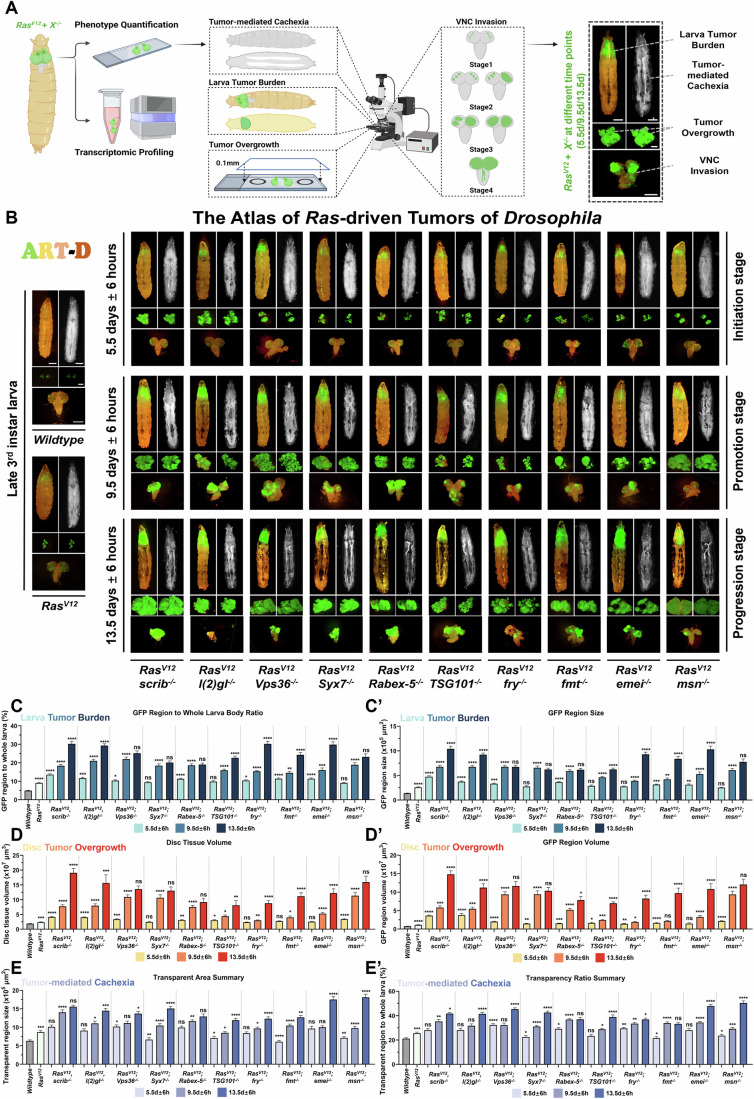
The Atlas of *Ras*-driven tumors in *Drosophila* (ART-D). (**A**) The schematic diagram outlining the protocol for harvesting malignant tumor samples at designated time points (5.5 d ± 6 h, 9.5 d ± 6 h, and 13.5 d ± 6 h AEL) to assess phenotypic data across four categories: larva tumor burden, tumor-mediated cachexia, tumor overgrowth, and ventral nerve cord (VNC) invasion. Scale bars, 500 µm for larva tumor burden and tumor-mediated cachexia, 400 µm for tumor overgrowth, and 400 µm for VNC invasion. (**B**) The Atlas of *Ras*-driven Tumors in *Drosophila* (ART-D). The summary of four representative phenotypic characteristics of normal tissue, *Ras* benign tumors, and ten malignant tumors at designated time points. Three stages with distinct phenotypic features were identified in ten *Drosophila Ras*-driven malignant tumors: initial stage, promotion stage, and progression stage. Scale bars: 500 µm for larva tumor burden and tumor-mediated cachexia, 400 µm for tumor overgrowth, and 400 µm for VNC invasion. (**C**–**E’**) The quantifications of larval tumor burden, tumor-mediated cachexia, and tumor overgrowth in ART-D. The larval tumor burden was quantified by the ratio of GFP region to whole larva (**C**) and the GFP region size (**C’**); the tumor overgrowth was quantified by the overall disc tissue volume (**D**) and the GFP region volume (**D’**); and the tumor-mediated cachexia was quantified by the transparent region size (**E**) and the ratio of transparent region to whole larva (**E’**), respectively. Statistical analysis for each group was performed using the Mann–Whitney *U* test and presented as mean ± SEM, *n* > 20. ns >0.05, *P < 0.05, **P < 0.01, ***P < 0.001, ****P < 0.0001, indicating the differences in larva tumor burden, tumor-mediated cachexia, or tumor overgrowth at the current stage compared to the previous stage, *Ras* group was compared with *wildtype* group, the initial stage tumors were compared with *Ras* group. For exact *P* values, in (**C**), from left to right, *Ras*^*V12*^ (****P < 0.0001), *Ras*^*V12*^*, scrib*^*−/−*^ (****P < 0.0001, ****P < 0.0001, ****P < 0.0001), *Ras*^*V12*^*, l(2)gl*^*−/−*^ (***P = 0.0004, ****P < 0.0001, ****P < 0.0001), *Ras*^*V12*^*; Vps36*^*−/−*^ (*P = 0.0347, ****P < 0.0001, ns *P* = 0.1010), *Ras*^*V12*^*; Syx7*^*−/−*^ (ns *P* = 0.8759, ****P < 0.0001, ns *P* = 0.2167), *Ras*^*V12*^*; Rabex-5*^*−/−*^ (****P < 0.0001, ****P < 0.0001, ns *P* = 0.3663), *Ras*^*V12*^*; TSG101*^*-/-*^ (ns *P* = 0.2431, ****P < 0.0001, ****P < 0.0001), *Ras*^*V12*^*; fry*^*−/−*^ (*P = 0.0188, ****P < 0.0001, ****P < 0.0001), *Ras*^*V12*^*; fmt*^*−/−*^ (****P < 0.0001, **P = 0.0015, ****P < 0.0001), *Ras*^*V12*^*; emei*^*−/−*^ (****P < 0.0001, ***P = 0.0008, ****P < 0.0001), *Ras*^*V12*^*; msn*^*−/−*^ (ns *P* = 0.9430, ****P < 0.0001, ns *P* = 0.0546); in (**C’**), from left to right, *Ras*^*V12*^ (****P < 0.0001), *Ras*^*V12*^*, scrib*^*-/-*^ (****P < 0.0001, ****P < 0.0001, ****P < 0.0001), *Ras*^*V12*^*, l(2)gl*^*−/−*^ (****P < 0.0001, ****P < 0.0001, ****P < 0.0001), *Ras*^*V12*^*; Vps36*^*−/−*^ (***P = 0.0002, ****P < 0.0001, ns *P* = 0.9452), *Ras*^*V12*^*; Syx7*^*−/−*^ (ns *P* = 0.8563, ****P < 0.0001, ns *P* = 0.7885), *Ras*^*V12*^*; Rabex-5*^*-/-*^ (****P < 0.0001, ****P < 0.0001, ns *P* = 0. 3592), *Ras*^*V12*^*; TSG101*^*−/−*^ (ns *P* = 0.5796, ****P < 0.0001, ****P < 0.0001), *Ras*^*V12*^*; fry*^*−/−*^ (ns *P* = 0.3900, ****P < 0.0001, ****P < 0.0001), *Ras*^*V12*^*; fmt*^*−/−*^ (***P = 0.0009, **P = 0.0032, ****P < 0.0001), *Ras*^*V12*^*; emei*^*−/−*^ (**P = 0.0053, ****P < 0.0001, ****P < 0.0001), *Ras*^*V12*^*; msn*^*−/−*^ (ns *P* = 0.6873, ****P < 0.0001, ns *P* = 0.0610); in (**D**), from left to right, *Ras*^*V12*^ (***P = 0.0005), *Ras*^*V12*^*, scrib*^*−/−*^ (****P < 0.0001, ****P < 0.0001, ****P < 0.0001), *Ras*^*V12*^*, l(2)gl*^*−/−*^ (****P < 0.0001, ****P < 0.0001, ***P = 0.0002), *Ras*^*V12*^*; Vps36*^*−/−*^ (***P = 0.0001, ****P < 0.0001, ns *P* = 0.1376), *Ras*^*V12*^*; Syx7*^*-/-*^ (ns *P* = 0.7011, ****P < 0.0001, ns *P* = 0.1083), *Ras*^*V12*^*; Rabex-5*^*−/−*^ (**P = 0.0052, ****P < 0.0001, ns *P* = 0.5225), *Ras*^*V12*^*; TSG101*^*−/−*^ (*P = 0.0221, *P = 0.0237, **P = 0.0050), *Ras*^*V12*^*; fry*^*−/−*^ (ns *P* = 0.8714, **P = 0.0021, ****P < 0.0001), *Ras*^*V12*^*; fmt*^*−/−*^ (ns *P* = 0.0795, *P = 0.0419, ****P < 0.0001), *Ras*^*V12*^*; emei*^*−/−*^ (ns *P* = 0.3199, ****P < 0.0001, ****P < 0.0001), *Ras*^*V12*^*; msn*^*−/−*^ (****P < 0.0001, ****P < 0.0001, ns *P* = 0.1646); in (**D’**), from left to right, *Ras*^*V12*^ (****P < 0.0001), *Ras*^*V12*^*, scrib*^*−/−*^ (****P < 0.0001, ***P = 0.0002, ****P < 0.0001), *Ras*^*V12*^*, l(2)gl*^*−/−*^ (****P < 0.0001, ***P = 0.0003, ****P < 0.0001), *Ras*^*V12*^*; Vps36*^*−/−*^ (****P < 0.0001, ****P < 0.0001, ns *P* = 0.4868), *Ras*^*V12*^*; Syx7*^*−/−*^ (**P = 0.0039, ****P < 0.0001, ns *P* = 0.3494), *Ras*^*V12*^*; Rabex-5*^*−/−*^ (**P = 0.0010, ****P < 0.0001, *P = 0.0328), *Ras*^*V12*^*; TSG101*^*−/−*^ (*P = 0.0241, ***P = 0.0008, ****P < 0.0001), *Ras*^*V12*^*; fry*^*−/−*^ (**P = 0.0056, *P = 0.0331, ****P < 0.0001), *Ras*^*V12*^*; fmt*^*−/−*^ (****P < 0.0001, ns *P* = 0.0635, ****P < 0.0001), *Ras*^*V12*^*; emei*^*−/−*^ (ns *P* = 0.2768, ****P < 0.0001, ****P < 0.0001), *Ras*^*V12*^*; msn*^*−/−*^ (****P < 0.0001, ****P < 0.0001, ns *P* = 0.4703); in (**E**), from left to right, *Ras*^*V12*^ (***P = 0.0001), *Ras*^*V12*^*, scrib*^*−/−*^ (ns *P* = 0.0781, ****P < 0.0001, ns *P* = 0.1841), *Ras*^*V12*^*, l(2)gl*^*-/-*^ (ns *P* = 0.5242, *P = 0.0152, ***P = 0.0002), *Ras*^*V12*^*; Vps36*^*−/−*^ (*P = 0.0244, ns *P* = 0.1214, *P = 0.0154), *Ras*^*V12*^*; Syx7*^*−/−*^ (**P = 0.0016, ****P < 0.0001, ****P < 0.0001), *Ras*^*V12*^*; Rabex-5*^*−/−*^ (ns *P* = 0.0609, **P = 0.0040, ns *P* = 0.4531), *Ras*^*V12*^*; TSG101*^*−/−*^ (*P = 0.0340, *P = 0.0115, ****P < 0.0001), *Ras*^*V12*^*; fry*^*−/−*^ (ns *P* = 0.9020, *P = 0.0390, ****P < 0.0001), *Ras*^*V12*^*; fmt*^*−/−*^ (****P < 0.0001, ****P < 0.0001, **P = 0.0036), *Ras*^*V12*^*; emei*^*−/−*^ (ns *P* = 0.4853, ns *P* = 0.2295, ****P < 0.0001), *Ras*^*V12*^*; msn*^*−/−*^ (**P = 0.0022, ****P < 0.0001, ****P < 0.0001); in (**E’**), from left to right, *Ras*^*V12*^ (***P = 0.0008), *Ras*^*V12*^*, scrib*^*−/−*^ (ns *P* = 0.4318, **P = 0.0018, *P = 0.0157), *Ras*^*V12*^*, l(2)gl*^*−/−*^ (ns *P* = 0.3331, ns *P* = 0.1352, ****P < 0.0001), *Ras*^*V12*^*; Vps36*^*−/−*^ (****P < 0.0001, ns *P* = 0.4470, ****P < 0.0001), *Ras*^*V12*^*; Syx7*^*−/−*^ (*P = 0.0196, ****P < 0.0001, ****P < 0.0001), *Ras*^*V12*^*; Rabex-5*^*−/−*^ (*P = 0.0136, ****P < 0.0001, ns *P* = 0.4453), *Ras*^*V12*^*; TSG101*^*−/−*^ (ns *P* = 0.2713, *P = 0.0101, ****P < 0.0001), *Ras*^*V12*^*; fry*^*−/−*^ (**P = 0.0015, **P = 0.0032, *P = 0.0317), *Ras*^*V12*^*; fmt*^*−/−*^ (*P = 0.0163, ****P < 0.0001, ns *P* = 0.6587), *Ras*^*V12*^*; emei*^*−/−*^ (ns *P* = 0.5673, ****P < 0.0001, ****P < 0.0001), *Ras*^*V12*^*; msn*^*−/−*^ (*P = 0.0315, ***P = 0.0004, ****P < 0.0001). Source data are available online for this figure.

Modeling tumorigenesis is crucial for deepening our understanding of cancer. To this end, we systematically characterized ten DRTs by sequentially tracking the tumorigenesis process through two complementary lenses: phenotypic malignancies and transcriptome dynamics (Fig. 1A). Phenotypic malignancies were evaluated using four key metrics: larval tumor burden, tumor-mediated cachexia, tumor overgrowth, and neighboring tissue invasion (Fig. 1A). We designated three time points for the collection of phenotypic and transcriptomic data: 5.5 days after egg laying (AEL), representing the standard duration for *Drosophila* larval development into pupae; 9.5 days AEL, an intermediate stage; and 13.5 days AEL, the maximum limit for late-stage malignant tumors prior to larval death in most cases (Fig. 1A). These data constitute the Atlas of *Ras*-driven Tumors in *Drosophila* (ART-D) (Fig. 1B). Through ART-D, we found all tumors exhibited incremental aggressive phenotypes over time and staged characteristics: (1) Initial stage (0–5.5 days AEL): Marked by tumor cell overgrowth. (2) Promotion stage (5.5–9.5 days AEL): Characterized by uncontrolled proliferation, onset of invasion, and early cachexia. (3) Progression stage (9.5–13.5 days AEL): Distinguished by severe cachexia and aggressive tissue invasion (Fig. 1B).

Larval tumor burden, quantified via GFP signal intensity and tumor-to-body ratio, reflected overall progression across genetic backgrounds. *Ras*^*V12*^ tumors with disruptions in *scrib*, *l(2)gl*, *TSG101*, *fry*, *fmt*, or *emei* showed steadily increasing burden from initial to progression stages. In contrast, tumors lacking *Vps36*, *Syx7*, *Rabex-5*, or *msn* peaked in burden during the promotion stage. It was noteworthy that the malignant tumors induced by the loss of *fry*, *fmt*, or *emei* exhibit a tumor burden at progression stages comparable to that resulting from the loss of cell polarity genes (*scirb* and *l(2)gl*) (Fig. 1C,C’). Tumor overgrowth, measured by volumetric analysis of whole tissues and the GFP-labeled regions, mirrored these trends: *scrib*, *l(2)gl*, *TSG101*, *fry*, *fmt*, and *emei* mutants exhibited dramatic expansion from promotion to progression stages, while *Vps36*, *Syx7*, *Rabex-5*, and *msn* mutants showed maximal proliferation rates earlier, during the promotion stage (Fig. 1D,D’). These results suggested that although ten DRTs exhibit malignant progression phenotypes, their growth rates differ.

Clinically, as the malignancy of the tumor progresses, patients often experience progressive and involuntary weight loss, particularly a decrease in skeletal muscle mass and loss of adipose tissue, leading to a gradually thinner body shape or significant wasting (Lodge et al, 2021; Liu et al, 2022). Similarly, in *Drosophila*, tumor-mediated cachexia manifests as progressive degradation of muscle and fat body tissues, leading to larval transparency or adult abdominal bloating (Lodge et al, 2021; Liu et al, 2022; Kwon et al, 2015). This phenotype serves as a hallmark of tumors that have entered the promotion stage (Fig. 1B). All DRTs exhibited significant increase in either transparent area or transparency ratio at promotion stage, except for *Vps36* loss (Fig. 1E,E’). Moreover, *Ras*^*V12*^ tumors lacking *l(2)gl*, *Vps36*, *Syx7*, *TSG101*, *fry*, *emei*, or *msn* all exhibited more drastic increase in both tumor-mediated cachexia parameters at progression stage (Fig. 1E,E’). Quantitative results suggested that tumor-induced cachexia begins to manifest during promotion stage of DRTs and progressively worsens as the tumor advances. Besides, the ventral nerve cord (VNC) was taken as neighboring tissue for invasion event quantification. Tumors with polarity defects (*scrib* or *l(2)gl* loss) displayed early invasion (25% stage 4 invasion at initial stage), increasing over time. Endocytosis-deficient tumors (*Vps36*, *Syx7*, *Rabex-5*, *TSG101*) showed severe invasion (>50% stage 4 invasion) by the promotion stage. *fry*-, *fmt*-, *emei*-, and *msn*-deficient tumors exhibited delayed invasion, peaking only in progression stages, with *msn* mutants showing weakest invasive phenotype (Fig. EV1G). Collectively, the ART-D comprehensively delineated the phenotypic characteristics of *Ras*^*V12*^ malignant tumors in stages, providing phenotypic references for elucidating the underlying molecular mechanisms.

### Phenotype-dependent tumor subtyping of *Drosophila Ras*-driven tumors

**Figure 2:**
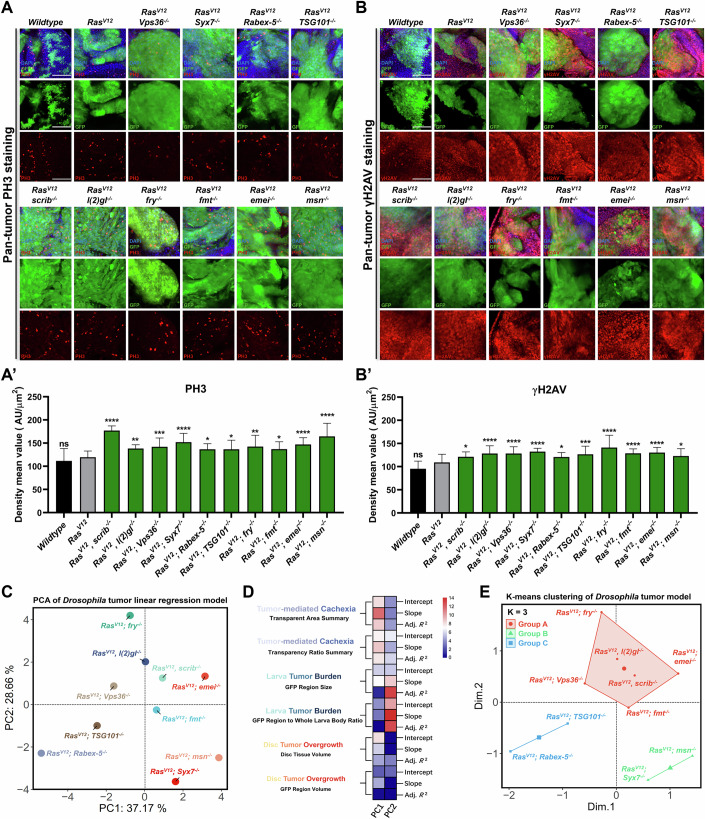
The classifications of *Drosophila Ras*-driven tumors. (**A**, **B**) Confocal images of eye-antennal discs or tumors harvested at 5.5–6.5 days AEL. Discs or tumors with indicated genotypes were stained with antibody against PH3 (**A**) and γH2AV (**B**). Scale bars: 50 µm. (**A’**–**B’**) Quantification results of immunofluorescence imaging of PH3 (**A**) and γH2AV (**B**). Kruskal–Wallis test was performed along with Dunn’s multiple comparisons test against the *Ras* group, *n* > 20. Results were presented as means ± SD. Significance of different genotypes were denoted as follows: ns > 0.05, *P < 0.05, **P < 0.01, ***P < 0.001, ****P < 0.0001. For exact *P* values, in (**A’**), from left to right, ns *P* > 0.9999, ****P < 0.0001, **P = 0.0012, ***P = 0.0006, ****P < 0.0001, *P = 0.0322, *P = 0.0250, **P = 0.0045, *P = 0.0264, ****P < 0.0001, ****P < 0.0001; in (**B’**), from left to right, ns *P* = 0.7060, *P = 0.0132, ****P < 0.0001, ****P < 0.0001, ****P < 0.0001, *P = 0.0400, ***P = 0.0008, ****P < 0.0001, ****P < 0.0001, ****P < 0.0001, *P = 0.0193. (**C**) The two-dimensional scatter plot visualizing the PCA projection of *Drosophila Ras*-driven tumors. The PCA was performed based on the robust linear regression model parameters of each *Ras*-driven tumor, including the intercept, slope, and adjusted R-squared values. (**D**) The contributions of PC1 and PC2 to the intercept, slope, and adjusted R-squared values of robust linear regression models representing tumor malignancies. (**E**) The K-means clustering of *Drosophila Ras*-driven tumors based on PCA results. A total of three groups were classified, group A comprises *scrib*-, *l(2)gl*-, *fry*-, *Vps36*-, and *emei*-deficient tumor models; group B comprises *Syx7*- and *msn*-deficient tumor models; group C comprises *Rabex5*- and *TSG101*-deficient tumor models. Source data are available online for this figure.

In addition to the four malignancy traits, we observed that DRTs exhibited hallmark characteristics of malignant tumor models, specifically strong autonomously overgrowth and genome instability at the initial stage of tumorigenesis, as evidenced by the immunofluorescent staining of PH3 and γH2AV (Fig. 2A–B’). However, the direct principal component analysis (PCA) of quantified malignancies revealed inter-tumor heterogeneity alongside similar progression directions among DRTs, indicating that progression trajectories diverged across different tumor models over time (Appendix Fig. S1A and Dataset EV1). To investigate the temporal divergence in phenotypic malignancy evolution across all DRTs, we applied robust linear regression to six digitized phenotypic malignancies by using sampling time as the independent variable. These parameters included GFP region size and GFP region to whole larva body ratio (Appendix Fig. S1B,B'), disc tissue volume and GFP region volume (Appendix Fig. S1C,C'), transparent area summary and transparency ratio summary (Appendix Fig. S1D,D').

The intercept primarily reflects malignancy at the initial stage, the slope indicates the progression rate from the initial to the advanced stage, and the adjusted *R*² values denote the goodness-of-fit of the regression models. Comparing the slopes of each regression equation with average regression equation, we found the *scrib*-, *l(2)gl*-, *fry*-, *emei*-, and *msn*-deficient tumors exhibited more aggressive progression on larva tumor burden than the average levels of all tumors, 0.59 for GFP region size and 1.81 for GFP region ratio (Appendix Fig. S2A,A'). Disruptions of *scrib*, *Syx7*, *emei*, or *msn* exhibited more aggressive progression on tumor overgrowth, 1.11 for disc tissue volume and 1.01 for GFP region volume (Appendix Fig. S2B,B'). *Ras*^*V12*^ tumors with *Syx7*, *emei*, or *msn* loss were also found more aggressive in tumor-mediated cachexia manifestations compared to the average progression level of all tumors, 0.732 for transparent area and 1.85 for transparency ratio (Appendix Fig. S2C,C'). The varying degrees of progression in different tumors prompted us to consider whether tumors can be classified based on these characteristics (Appendix Fig. S2D).

Using the intercepts, slopes, and adjusted R-squared values from robust linear regression models of each DRT as input, we conducted PCA to project the tumor models onto a two-dimensional map (Appendix Fig. S2D; Fig. 2C). The contributions of principal components (PC1 and PC2) to malignancies were shown, with PC1 capturing more features associated with tumor-mediated cachexia and PC2 reflecting more features related to larval tumor burden (Appendix Fig. S2D; Fig. 2D). Building on the PCA results, we applied K-means clustering for phenotype-dependent tumor subtyping. Consequently, Group A comprised *Ras*^*V12*^ tumors with deficiencies in *scrib*, *l(2)gl*, *Vps36*, *fry*, *emei*, or *fmt*; Group B consisted of tumors lacking *Syx7* or *msn*; and Group C included tumors deficient in *TSG101* or *Rabex-5* (Fig. 2E).

### Transcriptome dynamics of *Drosophila Ras*-driven tumor subtypes

**Figure 3:**
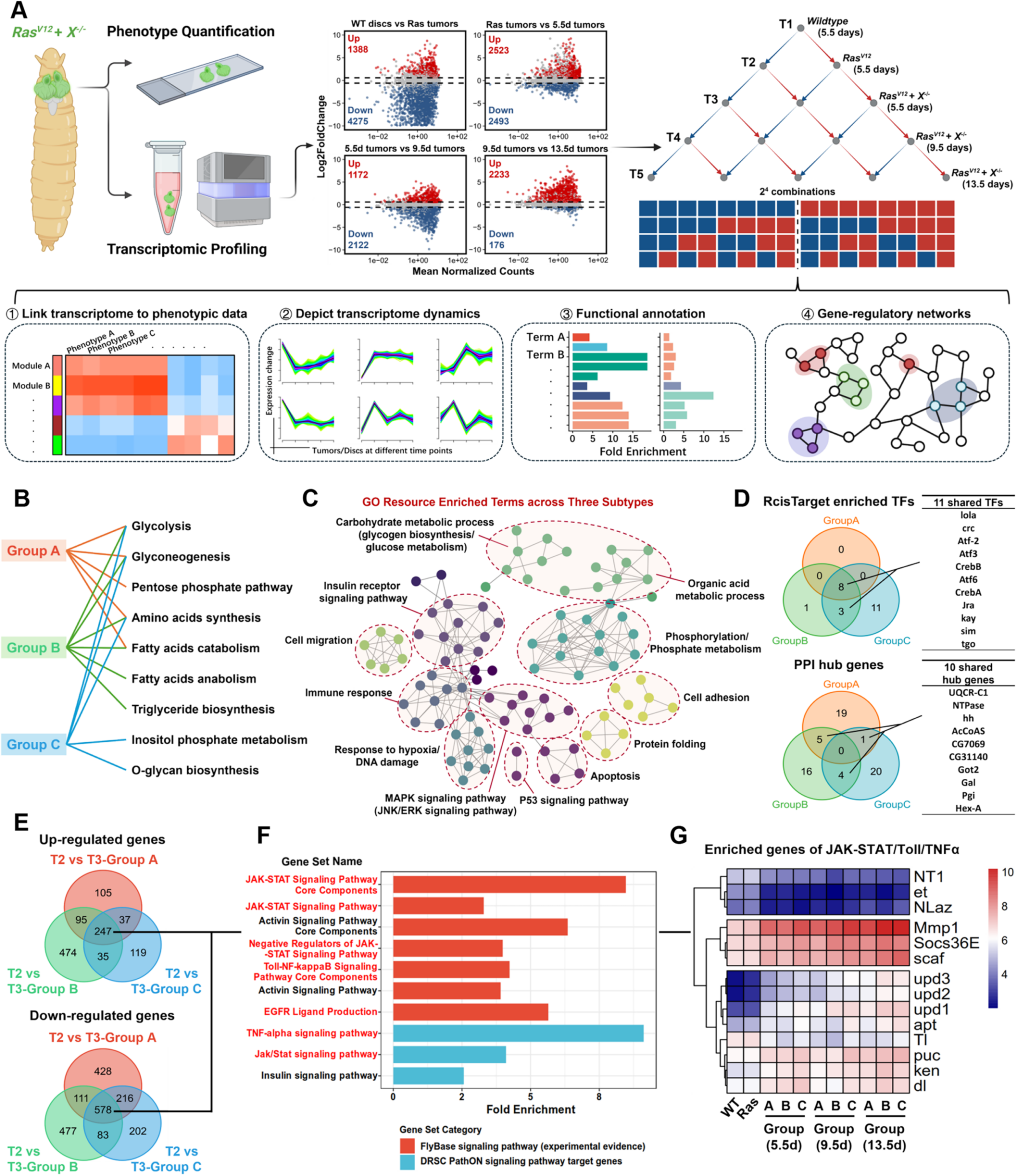
Dynamic transcriptomic profiles of different subtypes of *Drosophila Ras*-driven tumors. (**A**) The schematic diagram outlining the workflow for identifying tumor transcriptomic dynamics. The process began with the acquisition of bulk RNA-seq data from various tumors at different time points (Step 1), followed by the identification of DEGs (Step 2), the analysis of gene dynamic changing patterns (Step 3), and the extension of analyses (①~④) link transcriptomic changes with phenotypic malignancies (Step 4). (**B**) A summary of top-ranking terms enriched across different *Drosophila* databases of three subtypes of *Drosophila Ras*-driven tumors. (**C**) A gene regulatory network constructed based on GO terms that were significantly enriched. By intersecting GO terms and DEGs enriched in the three subtypes, the corresponding gene regulatory networks were constructed to explore the common biological changes among the three subtypes of tumor groups. (**D**) The Venn diagram depicting the overlap of shared TFs (top) and hub genes (bottom) in different groups of *Drosophila Ras*-driven tumors. The shared TFs (top) and hub genes (bottom) were listed on the corresponding table. (**E**–**G**) The Venn diagrams depicting the overlap of DEGs from different *Drosophila Ras*-driven tumor groups compared with *Ras*^*V12*^ benign tumors (**E**). The overlapped 247 upregulated genes (**E**, top) and 578 downregulated genes (**E**, bottom) were extracted for functional annotations, and the results were presented as a bar plot (**F**). The heatmap illustrated the expression levels of enriched genes from selected signaling pathways (**G**). Source data are available online for this figure.

To elucidate the molecular mechanisms driving malignant transformation across different tumor subtypes, we established five points to simulate longitudinal progression from baseline to progression stages: T1 (baseline; *wildtype* at 5.5 days AEL), T2 (benign; *Ras*^*V12*^ at 5.5 days AEL), T3 (initial; *Ras*^*V12*^*, X*^*−/−*^ at 5.5 days AEL), T4 (promotion; *Ras*^*V12*^*, X*^*−/−*^ at 9.5 days AEL), and T5 (progression; *Ras*^*V12*^*, X*^*−/−*^ at 13.5 days AEL). This trajectory reconstructed the progression from wild-type tissue to malignant tumors driven by genetic mutations. Theoretically, this progression trajectory encompassed 16 (2^4^) possible combinations of dynamic gene expression change patterns (Fig. 3A). Following batch effect correction (Appendix Fig. S3A and Dataset EV2), the direct PCA plots of all DRTs’ transcriptomic data exhibited substantial spatial divergence across distinct genetic contexts (Appendix Fig. S3B and Dataset EV3). Furthermore, we notified the sustained upregulation (clusters 1, 4, 14, 15) or downregulation (clusters 9, 19, 21) trends were the dominant patterns of all DRTs from T2 to T5 (Appendix Fig. S3C). However, not all observed dynamic changes were significantly associated with the tumor malignancy progression. Thereby, we conducted integrative analyses of phenotypic and transcriptomic data across different tumor subtypes to reveal dynamic transcriptomic features associated with malignant tumor progression (Fig. 3A).

**Figure EV2:**
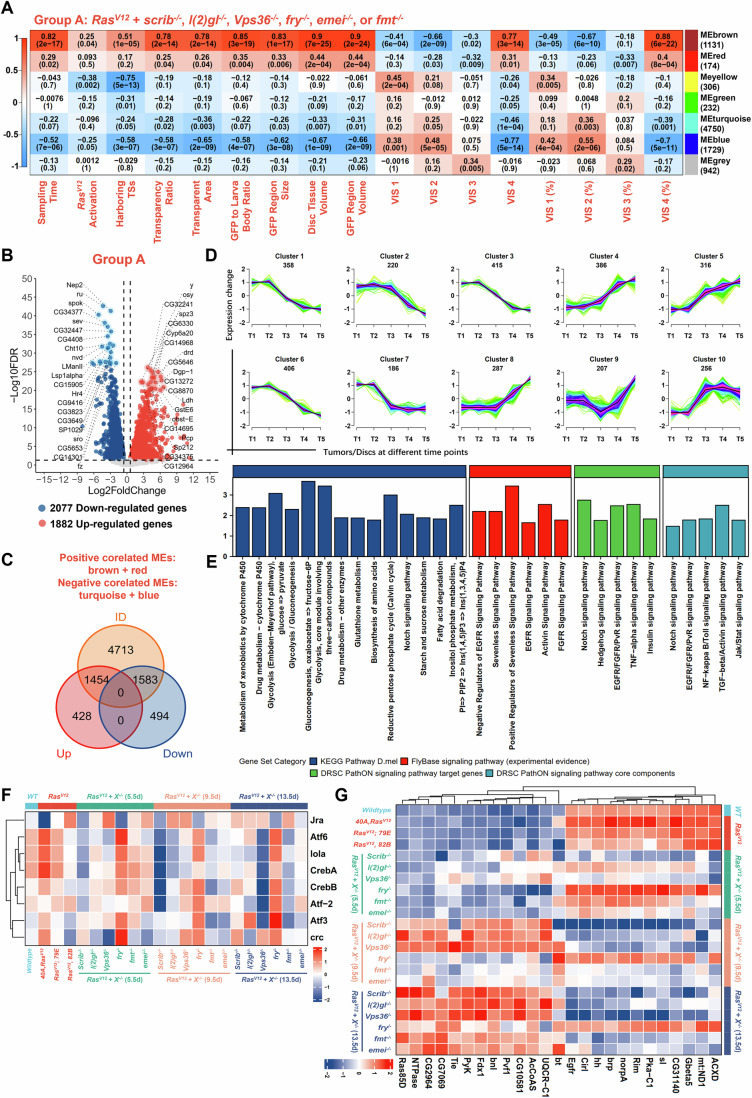
Dynamic transcriptomic profiles of Group A *Drosophila Ras*-driven tumors. (**A**) The WGCNA heatmap depicting the correlations between identified gene modules and phenotypic malignancies of Group A *Drosophila Ras*-driven tumors. The color intensity of each module represented the correlation coefficient, which was also displayed in the top line of the respective cell. Pearson correlation analysis was conducted to assess the associations between module eigengenes (MEs) and phenotypic data. The corresponding *P* value was shown in parentheses, with *P* values below 0.05 indicating statistically significant correlations. (**B**) The volcano plot of DEGs (T2 vs T5) in Group A of *Drosophila Ras*-driven tumors. The upregulated and downregulated genes were 1882 and 2077, respectively. (**C**) The Venn diagram depicting the overlap of genes in modules significantly associated with tumor malignancies (**A**) and DEGs (**B**). In total, 1454 genes were upregulated and significantly associated with tumor malignancies; 1583 genes were downregulated and significantly associated with tumor malignancies. (**D**) The line charts depicting dynamic transcriptomic changes of DEGs significantly associated with tumor malignancies in Group A *Drosophila Ras*-driven tumors (**C**), revealed by Mfuzz. (**E**) The bar plot depicting the functional annotations of candidate genes obtained from certain clusters (**D**), revealed by PANGEA. (**F**) The heatmap depicting the expression profiles of enriched TFs from T1 to T5. The TF enrichment of candidate genes derived from specific clusters (**D**) was performed using RcisTarget. (**G**) The heatmap depicting the expression profiles of identified hub genes from T1 to T5. The hub gene identification among candidate genes from specific clusters (**D**) was performed via STRING and BottleNeck.

In Group A DRTs, weighted gene co-expression network analysis (WGCNA) (Langfelder and Horvath, 2008) identified seven gene modules. Among these, the MEbrown and MEred gene modules showed significant positive correlations with multiple malignancies, whereas MEblue and MEturquoise gene modules exhibited significant negative correlations (Fig. EV2A). By extracting genes from these modules and intersecting them with differentially expressed genes (DEGs, T2 vs T5, Dataset EV4), we identified 1454 upregulated and 1583 downregulated DEGs significantly associated with tumor malignancy progression (Fig. EV2B,C). Subsequently, Mfuzz (Kumar and Futschik, 2007) clustering was applied to delineate the transcriptomic dynamics of these genes, revealing continuously upregulated gene clusters (Clusters 4, 5, and 8) and continuously downregulated gene clusters (Clusters 1, 2, 3, 6, and 7) from stage T1 to T5, which were selected as candidate gene sets (Fig. EV2D). Functional annotation by PANGEA indicated that, in Group A tumors, the biological processes significantly associated with phenotypic malignancies primarily included glycolysis, glyconeogenesis, pentose phosphate pathway, amino acid synthesis, and fatty acid metabolism. The signaling pathways significantly linked to phenotypic malignancies mainly involved the Notch, EGFR/FGFR/PvR, and TNF-α signaling pathways (Fig. EV2E).

**Figure EV3:**
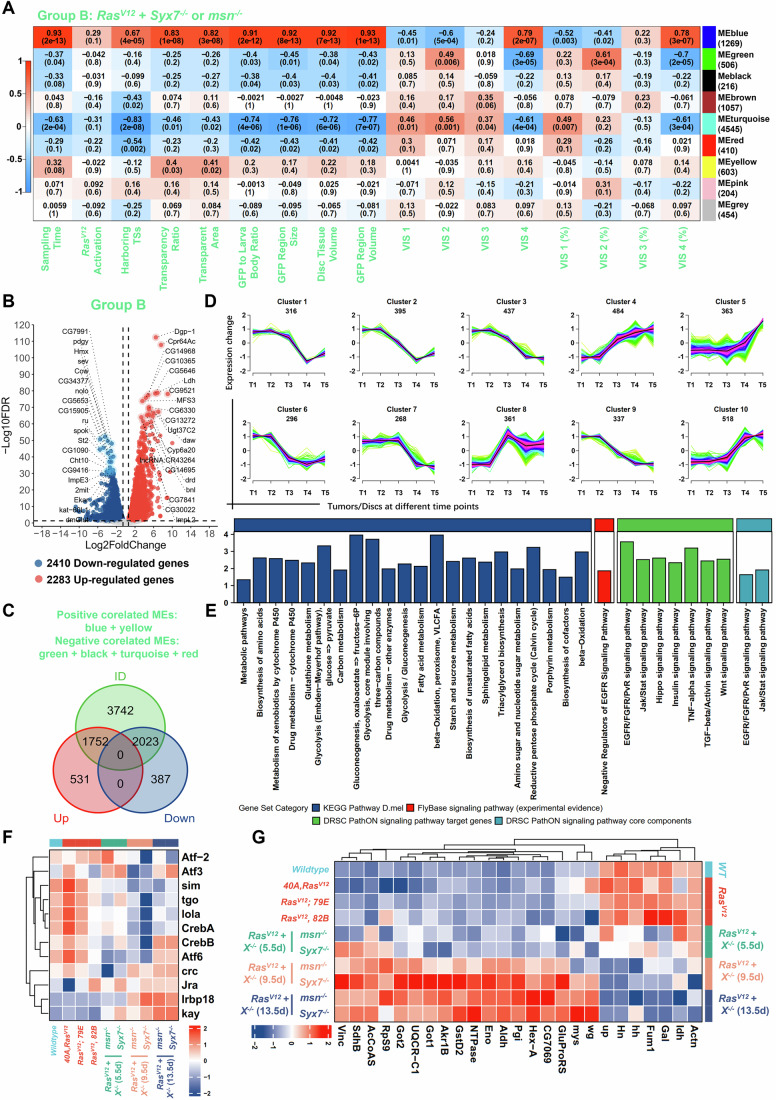
Dynamic transcriptomic profiles of Group B *Drosophila Ras*-driven tumors. (**A**) The WGCNA heatmap depicting the correlations between identified gene modules and phenotypic malignancies of Group B *Drosophila Ras*-driven tumors. The color intensity of each module represented the correlation coefficient, which was also displayed in the top line of the respective cell. Pearson correlation analysis was conducted to assess the associations between module eigengenes (MEs) and phenotypic data. The corresponding *P* value was shown in parentheses, with *P* values below 0.05 indicating statistically significant correlations. (**B**) The volcano plot of DEGs (T2 vs T5) in Group B of *Drosophila Ras*-driven tumors. The upregulated and downregulated genes were 2283 and 2410, respectively. (**C**) The Venn diagram depicting the overlap of genes in modules significantly associated with tumor malignancies (**A**) and DEGs (**B**). In all, 1752 genes were upregulated and significantly associated with tumor malignancies; 2023 genes were downregulated and significantly associated with tumor malignancies. (**D**) The line charts depicting dynamic transcriptomic changes of DEGs significantly associated with tumor malignancies in Group B *Drosophila Ras*-driven tumors (**C**), revealed by Mfuzz. (**E**) The bar plot depicting the functional annotations of candidate genes obtained from certain clusters (**D**), revealed by PANGEA. (**F**) The heatmap depicting the expression profiles of enriched TFs from T1 to T5. The TF enrichment of candidate genes derived from specific clusters (**D**) was performed using RcisTarget. (**G**) The heatmap depicting the expression profiles of identified hub genes from T1 to T5. The hub gene identification among candidate genes from specific clusters (**D**) was performed via STRING and BottleNeck.

**Figure EV4:**
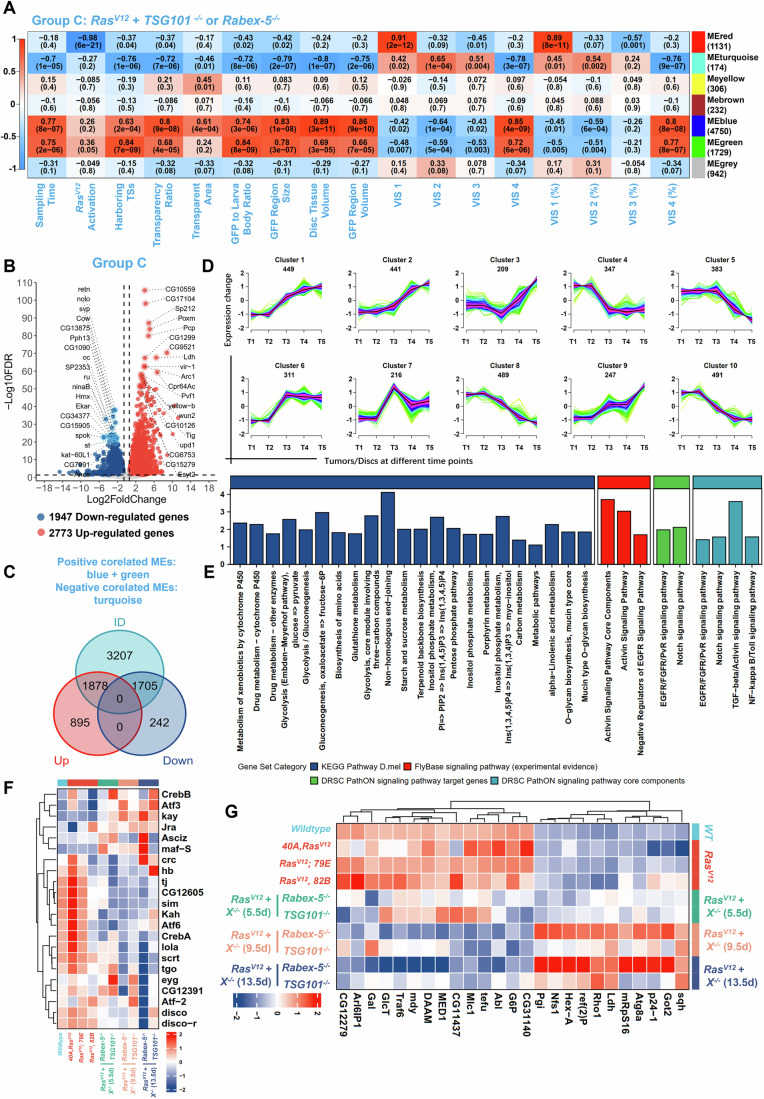
Dynamic transcriptomic profiles of Group C *Drosophila Ras*-driven tumors. (**A**) The WGCNA heatmap depicting the correlations between identified gene modules and phenotypic malignancies of Group C *Drosophila Ras*-driven tumors. The color intensity of each module represented the correlation coefficient, which was also displayed in the top line of the respective cell. Pearson correlation analysis was conducted to assess the associations between module eigengenes (MEs) and phenotypic data. The corresponding *P* value was shown in parentheses, with *P* values below 0.05 indicating statistically significant correlations. (**B**) The volcano plot of DEGs (T2 vs T5) in Group C of *Drosophila Ras*-driven tumors. The upregulated and downregulated genes were 2773 and 1947, respectively. (**C**) The Venn diagram depicting the overlap of genes in modules significantly associated with tumor malignancies (**A**) and DEGs (**B**). In all, 1878 genes were upregulated and significantly associated with tumor malignancies; 1705 genes were downregulated and significantly associated with tumor malignancies. (**D**) The line charts depicting dynamic transcriptomic changes of DEGs significantly associated with tumor malignancies in Group C *Drosophila Ras*-driven tumors (**C**), revealed by Mfuzz. (**E**) The bar plot depicting the functional annotations of candidate genes obtained from certain clusters (**D**), revealed by PANGEA. (**F**) The heatmap depicting the expression profiles of enriched TFs from T1 to T5. The TF enrichment of candidate genes derived from specific clusters (**D**) was performed using RcisTarget. (**G**) The heatmap depicting the expression profiles of identified hub genes from T1 to T5. The hub gene identification among candidate genes from specific clusters (**D**) was performed via STRING and BottleNeck.

By dissecting the transcriptome dynamics of Group B and Group C DRTs (Figs. EV3 and EV4), we found the functional annotation of continuously upregulated/downregulated gene clusters significantly associated with tumor malignant progression in Group B primarily involve amino acid synthesis, glycolysis and gluconeogenesis, fatty acid oxidation, synthesis of unsaturated fatty acids, and triglyceride biosynthesis. The signaling pathways significantly associated with tumor malignant progression mainly include EGFR/FGFR/PvR, JAK/STAT, and Hippo pathways (Fig. EV3A–E). In Group C DRTs, the biological processes significantly associated with tumor malignant progression, apart from glycolysis and gluconeogenesis, also include inositol phosphate metabolism and O-glycan biosynthesis. The signaling pathways significantly associated with tumor malignant progression mainly include TGFβ/Activin, EGFR/FGFR/PvR, and Notch pathways (Fig. EV4A–E).

By horizontally comparing the different tumor subtypes, we found the common features across tumor subtypes are enriched in biological processes such as glycolysis, gluconeogenesis, and amino acid metabolism, as well as the EGFR/FGFR/PvR signaling pathway, indicating the common characteristics for maintaining tumor malignant progression during T1 to T5 of DRTs. Meanwhile, we also found a large number of biological processes related to fatty acid metabolism enriched in Group B, while biological processes related to inositol phosphate metabolism and O-glycan biosynthesis were enriched in Group C, revealing the specific metabolic features of tumor Groups B and C in maintaining tumor malignant progression from T1 to T5 (Fig. 3B). Gene regulatory networks were constructed based on significantly enriched Gene Ontology (GO) terms. By intersecting these GO terms with DEGs across the three tumor subtypes, we developed corresponding networks to elucidate shared biological alterations among the groups, including glucose metabolism, glycogen biosynthesis, organic acid metabolism, and MAPK signaling pathway (Fig. 3C). Notably, in terms of signal transduction, both tumor Groups A and C were enriched in Notch signaling pathway and NF-κB/Toll signaling pathway, while tumor Groups A and B were enriched in the JAK/STAT signaling pathway. The Hippo signaling pathway was unique to Group B, and the TGFβ/Activin signaling pathway showed higher enrichment in Group C.

Through transcription factor enrichment analysis and hub gene identification of DEGs significantly associated with tumor malignant progression in different tumor subtypes, we found that Notch signaling pathway-related transcription factor *longitudinals lacking* (*lola*), as well as the MAPK signaling pathway-related transcription factors *Jun-related antigen* (*Jra*) and *kayak* (*kay*), were co-enriched in different tumor groups (Figs. 3D, EV2F, and EV3F). Based on the protein-protein interaction (PPI) networks established using STRING database (Mering, 2003; Szklarczyk et al, 2025), *Glutamate oxaloacetate transaminase 2* (*Got2*), *NTPase*, *Phosphoglucose isomerase* (*Pgi*), and *Hexokinase A* (*Hex-A*) were identified as hub genes in at least two tumor subtypes (Figs. 3D, EV2G, and EV3G). These results suggest that the biological processes or signaling pathways regulated by these transcription factors and hub genes may play important roles in maintaining tumor malignant progression.

### Activation of NF-κB/Toll and MAPK signaling in T3 stage across all tumor subtypes

To further investigate signaling pathways that were crucial for tumor initiation, we compared different subtypes of DRTs (T3) with *Ras* benign tumors (T2). The intersection showed 247 co-upregulated genes and 578 co-downregulated genes in DRTs at the T3 stage (Fig. 3E). Functional annotation revealed that these genes were primarily involved in regulating the JAK/STAT, NF-κB/Toll, and MAPK signaling pathways (Fig. 3F), consistent with the common features observed in the dynamic transcriptomic profiles of different tumor subtypes from T1 to T5. Additionally, we found that the expression levels of JAK/STAT ligands *Unpaireds* (*Upds*: *Upd1*, *Upd2*, and *Upd3*), the receptor *Toll* (*Tl*) and the transcription factor *dorsal* (*dl*) from the NF-κB/Toll signaling pathway, as well as the JNK signaling pathway target genes *MMP1* and *puckered* (*puc*), were higher at the T3 than T2, with expression levels increasing progressively during tumor development (Fig. 3G). Together, these findings suggest that the JAK/STAT, NF-κB/Toll, and MAPK signaling pathways may be simultaneously activated in various tumor subtypes at the T3 stage.

Bulk-level dynamic gene expression analyses may overlook tumor-specific characteristics. To address this, we used gene set variation analysis (GSVA) (Hänzelmann et al, 2013) and single-sample gene set enrichment analysis (ssGSEA) (Subramanian et al, 2005; Barbie et al, 2009) to score 15 major signaling pathways in *Drosophila* (Appendix Fig. S4A,B). To mitigate interference from antagonistic effects, we prioritized positive regulators of signaling pathways to evaluate pathway activation across ten *Drosophila* tumor models from T1 to T5 (Appendix Fig. S4A). Results showed consistent activation of JAK/STAT signaling from T3 to T5 in eight genotypic tumors, with activation in all genotypic tumors at the T5. Similarly, TNF-α signaling was activated at the T3 stage in nearly all tumors. Other MAPK-related signaling pathways (EGFR, FGFR, PvR, Sevenless, and Torso) were primarily activated at T3, although FGFR and Torso signaling were inactivated in endocytosis-impaired tumors (*Vps36*^*−/−*^, *Syx7*^*−/−*^, *Rabex-5*^*−/−*^, and *TSG101*^*−/−*^). The widespread activation of JAK/STAT and MAPK signaling since or at T3 in DRTs further elucidates the positive correlations between these two pathways and tumor progression at the bulk-level of transcriptome dynamics.

Notably, *l(2)gl*-, *TSG101*-, *fry*-, or *fmt*-deficient tumors exhibited co-activation of the NF-κB/Toll and NF-κB/Imd pathways at T3. Insulin signaling was activated at T3 in seven tumor types and remained continuously active from T1 to T5 in *Ras*^*V12*^ tumors with disrupted *l(2)gl*, *TSG101*, *emei*, *fmt*, or *msn*. Given that JNK signaling activation can lead to increased transcription of the ligands of JAK/STAT signaling *Upds*, we selected the JNK and NF-κB signaling pathways for experimental validation. Immunofluorescence staining revealed increased phosphorylation levels of the MAPK pathway effectors rolled (*rl/ERK*) and basket (*bsk/JNK*), as well as elevated protein levels of their downstream targets *MMP1* and *wingless* (*wg*), in all DRTs (Appendix Fig. S5A–D'), consistent with our hypothesis of extensive MAPK pathway activation. Furthermore, immunofluorescence staining of the key downstream transcription factors dl and Relish> (*Rel*) of NF-κB/Toll and NF-κB/Imd pathways showed consistently elevated dl protein levels across all DRTs, whereas Rel expression increased only in tumors with specific genotypes, such as *Ras*^*V12*^ tumors with *fmt* deficiency (Appendix Fig. S6A–B'). Collectively, these findings suggest that activation of the MAPK and NF-κB/Toll signaling pathways may represent common mechanisms underlying the malignant progression of DRTs.

### Notch activation and Hippo inactivation facilitate excessive tumor overgrowth

**Figure 4:**
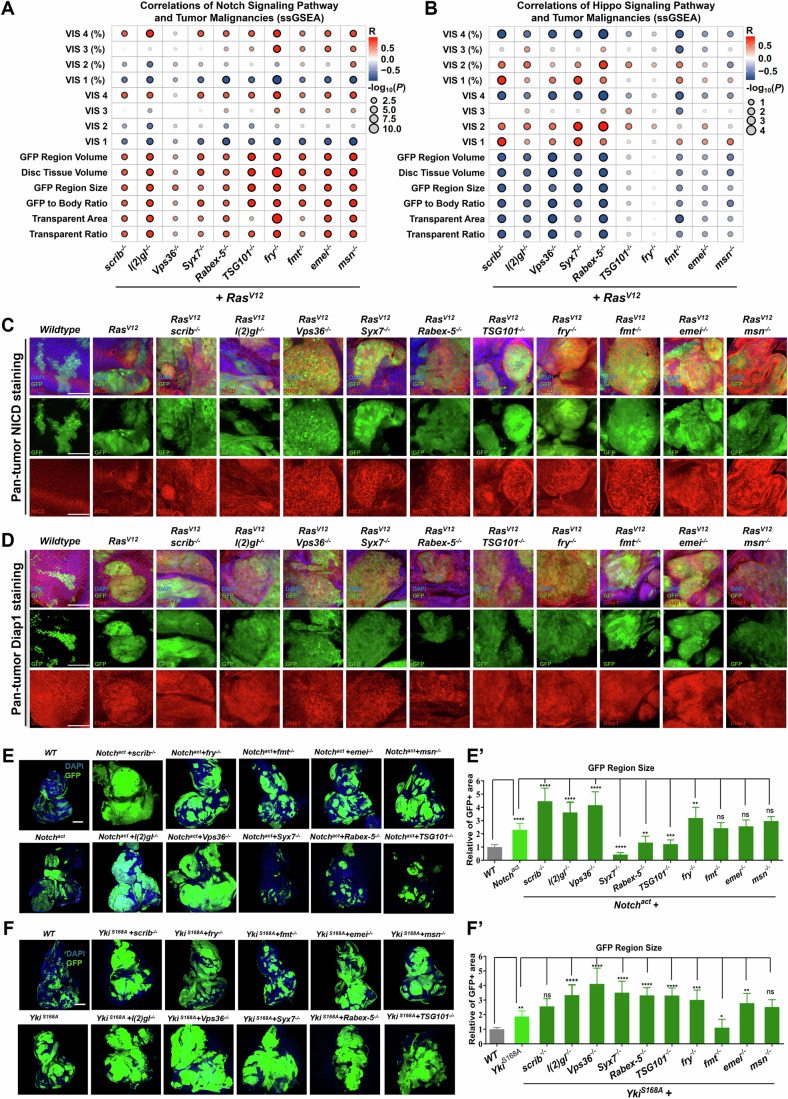
The tumor promoting role of Notch activation and Hippo inactivation within of *Drosophila Ras*-driven tumors. (**A**) The dot plot depicting the correlations between Notch signaling pathway scores and tumor malignancies. The signaling scores across ten *Drosophila Ras*-driven tumors were evaluated by ssGSEA. The correlations were evaluated by Pearson correlation test. The color of the dots indicated the Pearson correlation coefficients (R), while the size of the dots reflected the negative log10-transformed *P* values. (**B**) The dot plot depicting the correlations between Hippo signaling pathway scores and tumor malignancies. The signaling scores across ten *Drosophila Ras*-driven tumors were evaluated by ssGSEA. The correlations were evaluated by Pearson correlation test. The color of the dots indicated the Pearson correlation coefficients (R), while the size of the dots reflected the negative log10-transformed *P* values. (**C**,** D**) Confocal images of eye-antennal discs or tumors harvested at 5.5–6.5 days AEL. Discs or tumors with indicated genotypes were stained with antibodies against Notch and Hippo signaling pathway members, NICD (**C**) and Diap1 (**D**), respectively. Scale bars: 50 µm. (**E**–**E’**) The confocal images (**E**) and quantifications of GFP region size (**E’**) within discs or tumors overexpressing the activated form of *Notch* (*Notch*^*act*^) along with corresponding tumor suppressors. Statistical analysis for each group was performed using one-way ANOVA with Dunnett’s multiple comparisons test. Data are presented as mean ± SD (*n* > 10). Significance levels are denoted as follows: ns >0.05, *P < 0.05, **P < 0.01, ***P < 0.001, ****P < 0.0001. Scale bars: 100 µm. For exact *P* values, in (**E’**), from left to right, ****P < 0.0001, ****P < 0.0001, ****P < 0.0001, ****P < 0.0001, ****P < 0.0001, **P = 0.0014, ***P = 0.0003, **P = 0.0049, ns *P* = 0.9991, ns *P* = 0.8999, ns *P* = 0.0875. (**F**–**F’**) The confocal images (**F**) and quantifications of GFP region size (**F’**) within discs or tumors overexpressing the activated form of *Yki* (*Yki*^*S168A*^) along with corresponding tumor suppressors. Statistical analysis for each group was performed using one-way ANOVA with Dunnett’s multiple comparisons test. Data are presented as mean ± SD (*n* > 10). Significance levels are denoted as follows: ns >0.05, *P < 0.05, **P < 0.01, ***P < 0.001, ****P < 0.0001. Scale bars: 100 µm. For exact *P* values, in (**F’**), from left to right, **P = 0.0059, ns *P* = 0.0594, ****P < 0.0001, ****P < 0.0001, ****P < 0.0001, ****P < 0.0001, ****P < 0.0001, ***P = 0.0006, *P = 0.0193, **P = 0.0073, ns *P* = 0.902. Source data are available online for this figure.

To further assess the oncogenic potential of major signaling pathway dynamics, we correlated pathway activation status with tumor progression parameters. Notably, Notch signaling activation exhibited a positive correlation with tumor progression, whereas Hippo signaling activation inversely correlated with severity across DRTs (Fig. 4A,B; Appendix Fig. S7A,B). To validate the broad dysregulation of these pathways in DRTs, we performed immunofluorescent staining of key Notch components, including the ligand Delta (*Dl*) and Notch intracellular domain (*NICD*), and its target *dMyc*, and the transcriptional co-factor of the Hippo pathway, Yorkie (*Yki*), and its downstream target gene *Death-associated inhibitor of apoptosis 1* (*Diap1*). Compared to *wildtype* discs and *Ras* benign tumors, robust upregulation of Delta, NICD, dMyc, and Diap1, as well as increased nuclear translocation of Yki (Fig. 4C,D; Appendix Fig. S7C–C'), was observed across tumor samples, confirming Notch hyperactivation or Hippo inactivation.

Next, functional validation of Notch and Hippo signaling in tumorigenesis was conducted by expressing constitutively active *Notch* (*Notch*^*act*^) or *Yki* (*Yki*^*S168A*^) in GFP-marked cells deficient for specific tumor suppressors. *Notch*^*act*^ synergized with the loss of *scrib*, *l(2)gl*, *Vps36*, or *fry* to drive aggressive tumor overgrowth, characterized by giant larvae phenotypes in *scrib*- and *Vps36*-deficient tumors (Fig. 4E,E’; Appendix Fig. S8D,F). Conversely, *Notch*^*act*^ failed to promote clone expansion in cells lacking *Syx7*, *Rabex-5*, or *TSG101* (Fig. 4E,E’; Appendix Fig. S8D,F). The discrepancy of *Notch*^*act*^ synergizing endocytosis-defects may be due to the interference of different endocytosis processes. Previous studies showed defects in *Vps36*, *Syx7*, or *TSG101* could drive tumorigenesis and exhibit elevated Notch due to internalization, but the target gene expression only elevated in *TSG101*-deficient cells (Lu and Bilder, 2005; Vaccari et al, 2008; Herz et al, 2009). Similarly, *Yki*^*S168A*^ cooperated with defects in *l(2)gl*, *Vps36*, *Syx7*, *Rabex-5*, *TSG101*, *fry*, or *emei* to promote tumor overgrowth, inducing giant larvae phenotypes in polarity-deficient cells (Fig. 4F,F’; Appendix Fig. S8E,G). These results imply a context-dependent synergy between tumor suppressor loss and hyperactivated *Notch* or *Yki*. Intriguingly, we noticed that *l(2)gl*-, *Vps36*-, and *fry*- deficient cells exhibited unique potency, cooperating with multi oncogenic mutations (*Ras*^*V12*^, *Notch*^*act*^, or *Yki*^*S168A*^) to fuel malignancy (Figs. EV1B–E and  4E–F’), suggesting that these potent tumor suppressors may converge on shared mechanisms enabling tumor plasticity.

### Multi-omics analysis links JNK/Notch/Toll activation and Hippo inactivation to tumor progression

Among these three tumor suppressors, *fry* encodes a 3,479-amino acid protein that functions in a complex with the NDR (Nuclear Dbf2-driven) kinase tricornered (*trc*) and mob as tumor suppressor (*mats*) (Devroe et al, 2004; He et al, 2005; Chiba et al, 2009; Fang et al, 2010). Fry-NDR kinase signaling is implicated in regulating polarized cell growth and epidermal morphogenesis, with *fry* and *trc* mutants exhibiting increased cell volume (Fang and Adler, 2010; Fang et al, 2010). Notably, depletion of *FRY* could promote the nuclear localization of YAP/TAZ with decreased NDR1/2 kinase activities and YAP phosphorylation levels independent of LATS1/2 activity (Irie et al, 2020). Moreover, downregulation of the *trc* ortholog, *NDR1*, promotes epithelial-mesenchymal transition (EMT) in prostate cancer, while its overexpression suppresses metastatic phenotypes (Yue et al, 2018). These findings suggest a conserved tumor-suppressive role for *fry* in oncogenic contexts, though the underlying mechanisms remain unclear.

**Figure 5:**
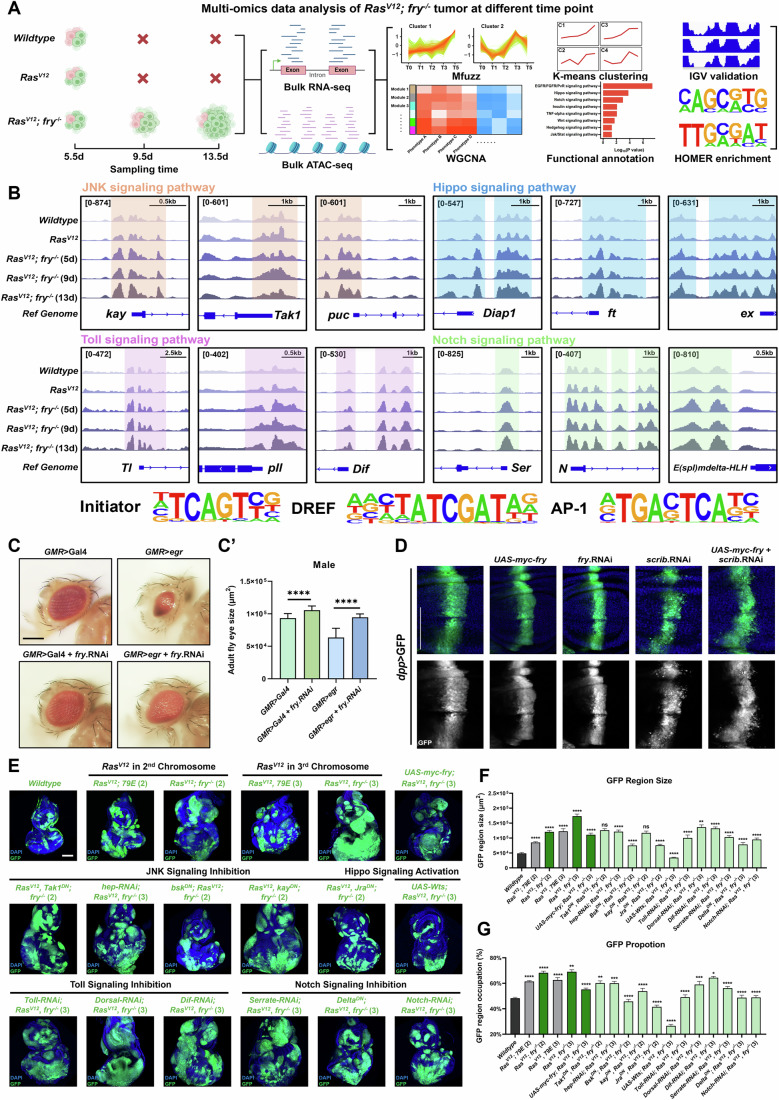
Multi-omics data analysis of *Ras*^*V12*^*; fry*^*−/−*^ tumors across different stages. (**A**) The flowchart depicting the multi-omics data analysis of *Ras*^*V12*^; *fry*^*−/−*^ tumors at various stages. Samples with indicated genotypes were harvested at designated time points (T1 to T5) for bulk RNA-seq and bulk ATAC-seq. Subsequent data analysis was conducted as outlined to uncover the mechanisms underlying tumorigenesis. (**B**) The IGV plot (top) illustrates the accessibility of the key effectors or target genes from specific signaling pathways, JNK signaling pathway (light orange), Notch signaling pathway (light green), Hippo signaling pathway (light blue), and Toll signaling pathway (light magenta). Data were normalized and presented as RPKM, with the maximum signal intensity and the scale bar for each region were denoted at the top. The identified top-ranked transcription factor binding motifs and their corresponding transcription factors (bottom). (**C**–**C’**) The light micrographs (**C**) and quantification results (**C’**) of adult eyes (Male) with the indicated genotypes. Scale bars: 200 µm. Statistical analysis for each group was performed using the Mann–Whitney *U* test or Student’s *t* test based on the variance. Results were presented as means ± SD, *n* > 25. Significance of different genotypes were denoted as follows: ns >0.05, *P < 0.05, **P < 0.01, ***P < 0.001, ****P < 0.0001. For exact *P* values, in (**C’**), from left to right, ****P < 0.0001, ****P < 0.0001. (**D**) The fluorescence micrographs of wing pouch regions. Wing discs were placed as anterior to the left and dorsal up with genotypes denoted. Scale bars: 100 µm. (**E**) The confocal images of JNK signaling inhibition, Hippo signaling inactivation, NF-κB/Toll signaling inhibition, and Notch signaling inhibition in *Ras*-driven *fry*-deficient tumors. To ensure precise data quantification, we generated *fry*-deficient tumors expressing *Ras*^*V12*^ on either the second (2nd) or third (3rd) chromosome of *Drosophila*, designated as *Ras*^*V12*^*; fry*^*−/−*^ (2) and *Ras*^*V12*^*, fry*^*−/−*^ (3), respectively, with the chromosomal location of *Ras*^*V12*^ indicated in parentheses. This nomenclature was applied consistently to both experimental and control groups, followed by statistical analysis of the corresponding tumor samples. Samples were observed at 5.5–6.5 days AEL. Scale bar: 100 µm. (**F**) Quantification of the GFP region size within eye-antennal disc across different genotypes in (**E**). Statistical significance was assessed using either the Mann–Whitney *U* test or Student’s *t* test, depending on variance homogeneity (*n* > 25). Experimental groups were compared against the corresponding *Ras* control group. Data are presented as means ± SEM. Significance levels are denoted as follows: ns >0.05, *P < 0.05, **P < 0.01, ***P < 0.001, ****P < 0.0001. For exact *P* values, from left to right, ****P < 0.0001, ****P < 0.0001, ****P < 0.0001, ****P < 0.0001, ****P < 0.0001, ns *P* = 0.5027,****P < 0.0001, ****P < 0.0001, ns *P* = 0.6979, ****P < 0.0001, ****P < 0.0001, ****P < 0.0001, **P = 0.0017, ****P < 0.0001, ****P < 0.0001, ****P < 0.0001, ****P < 0.0001. (**G**) Quantification of GFP region occupation within eye-antennal disc across different genotypes in (**E**). Statistical significance was assessed using either the Mann–Whitney *U* test or Student’s *t* test, depending on variance homogeneity (*n* > 25). Experimental groups were compared against the corresponding *Ras* control group. Data are presented as means ± SEM. Significance levels are denoted as follows: ns > 0.05, *P < 0.05, **P < 0.01, ***P < 0.001, ****P < 0.0001. For exact *P* values, from left to right, ****P < 0.0001, ****P < 0.0001, ****P < 0.0001, **P = 0.0095, ****P < 0.0001, **P = 0.0015, ***P = 0.0004, ****P < 0.0001, ****P < 0.0001, ****P < 0.0001, ****P < 0.0001, ****P < 0.0001, ***P = 0.0007, *P = 0.0134, ****P < 0.0001, ****P < 0.0001, ****P < 0.0001. Source data are available online for this figure.

To address this gap, we performed an integrative multi-omics analysis of bulk RNA-seq and Assay for Transposase-Accessible Chromatin with high-throughput sequencing (ATAC-seq) on *Ras*^*V12*^*; fry*^−/−^ tumors, a novel model previously uncharacterized in cancer studies, across distinct tumor stages (Fig. 5A). Initial DEG analysis revealed no sustained up- or downregulated candidates upon cross-stage comparison (Appendix Fig. S9A), we subsequently employed WGCNA to identify dynamic transcriptional modules that positively correlated with tumor progression metrics (e.g., tumor burden, cachexia severity, VNC invasion; Appendix Fig. S9B–F). Module gene re-clustering and pathway annotation highlighted enrichment of MAPK, NF-κB/Toll, Notch, and Hippo signaling pathways (Appendix Fig. S9F,G), suggesting their involvement in driving *Ras*^*V12*^*; fry*^*−/−*^ tumor malignancies. Temporal expression profiling revealed stage-specific activation: *Tl*, *mastermind* (*mam*), *Diap1*, *frizzled* (*fz*), and *wg* peaked at T3, while Insulin signaling genes (*GlyP*, *Lpin*, *Ilp2*, *Ilp3*, *Thor*) were mainly upregulated at T4-T5, coinciding with aggressive cachexia and invasion (Appendix Fig. S9F). Additionally, ATAC-seq confirmed elevated chromatin accessibility at promoter regions of key effectors/targets in JNK, Hippo, NF-κB/Toll, and Notch pathways across stages (Fig. 5B). Motif enrichment analysis of differentially accessible peaks identified top-ranked transcription factor binding sites for Initiator, DREF, and AP-1 (Fig. 5B). Collectively, our integrative analysis implicates JNK, NF-κB/Toll, Hippo, and Notch signaling as cooperative drivers of tumorigenesis in *Ras*-activated, *fry*-deficient contexts.

To investigate the physiological and pathological roles of *fry* in regulating the JNK, NF-κB/Toll, Hippo, and Notch signaling pathways, we knocked down *fry* expression in the posterior region of the *Drosophila* wing disc and examined the expression levels of target genes or key kinases associated with these pathways (Appendix Fig. S10A,B). No significant differences were observed compared to the *wildtype* controls. Similarly, *fry* knockdown in the thorax did not affect the JNK-dependent thorax closure process (Appendix Fig. S10C), but when downregulating *fry* expression in *Drosophila* adult eye showed a significant enlargement of adult eye size (Fig. 5C,C’; Appendix Fig. S10D,D'). While under pathological conditions, inhibition of *fry* rescued the *eiger* (*egr*)-induced small eye phenotype (Fig. 5C,C’; Appendix Fig. S10D,D'), and overexpression of *fry* partially suppressed *scrib* knockdown-induced JNK-dependent cell migration (Fig. 5D). These findings suggest that while *fry* does not exert a significant regulatory influence on these pathways under physiological conditions, it can modulate JNK-dependent processes in pathological contexts.

To validate the functional necessity of these pathways in driving tumorigenesis, we inhibited JNK signaling in *Ras*^*V12*^; *fry*^*−/−*^ tumors using RNAi lines or dominant-negative forms of its key effectors (*TGF-β activated kinase 1* [*Tak1*], *hemipterous* [*hep*], *bsk*, *kay*, and *Jra*). This intervention resulted in a marked reduction in tumor region size (marked by GFP) and tumor region occupation (Fig. 5E–G; Appendix Fig. S10E). Further investigation of the oncogenic roles of NF-κB/Toll, Notch, and Hippo pathways showed similar results. We found that knocking-down of *Tl* and *Dorsal-driven immunity factor* (*Dif*) to suppress NF-κB/Toll signaling efficiently inhibited tumor growth, whereas *dl* knockdown had no effect on overall disc size but showed consistent inhibition on tumor region size and occupation (Fig. 5E–G; Appendix Fig. S10E). When inhibiting the activity of Notch signaling pathway by suppressing the ligands (*Delta* and *Ser*) and receptor (*Notch*), we found these interventions significantly inhibit the *Ras*-driven tumor overgrowth (Fig. 5E–G; Appendix Fig. S10E). Notably, by overexpressing *warts* (*wts*) to activate Hippo signaling, we also observed a remarkable suppression on *Ras*^*V12*^, *fry*^*−/−*^ tumors (Fig. 5E–G; Appendix Fig. S10E). These findings align with the premise that Notch hyperactivation and Hippo inactivation are critical drivers of malignancy. Moreover, we found that interfering with the activity of either signaling pathway could significantly rescue the pupariation delay defect in larvae bearing *Ras*^*V12*^; *fry*^*−/−*^ tumors (Appendix Fig. S10F–G'). These findings confirm the functional necessity and synergistic interactions of JNK, NF-κB/Toll, Notch, and Hippo signaling pathways in driving tumorigenesis.

### Translational analysis of *Drosophila* tumor suppressors in human cancers

**Figure 6:**
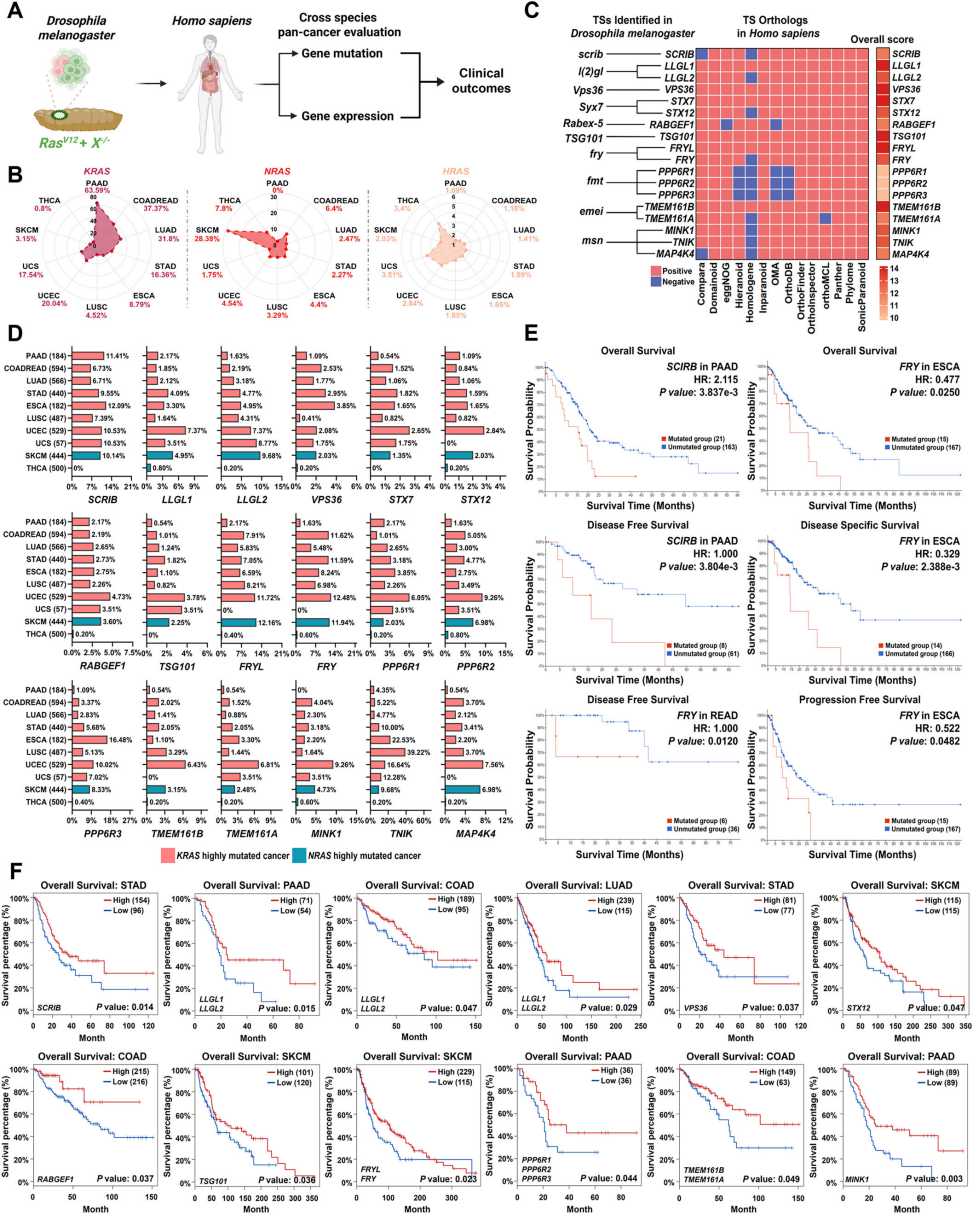
The pan-cancer evaluation of human orthologs in *KRAS*/*NRAS*-driven cancers. (**A**) The scheme depicting the evaluation of the impact of *Drosophila* tumor suppressor human orthologs on human cancers from two perspectives, gene mutation and gene expression. (**B**) The mutation rate of *RAS* family genes (*KRAS, NRAS, HRAS*) in ten cancers (PAAD, COADREAD, LUAD, STAD, ESCA, LUSC, UCEC, UCS, SKCM, THCA). Data was obtained from TCGA PanCancer Atlas Studies of cBioProtal. (**C**) The eighteen *Homo sapiens* orthologs correspond to ten *Drosophila* tumor suppressors. The human orthologs were identified via 14 databases, the overall scores of each orthologous genes across different databases were presented in the right column. (**D**) The mutation rate of eighteen human orthologs across the ten cancer types that have high mutation rate of *KRAS* or *NRAS*. (**E**) Kaplan–Meier survival curves illustrating the impact of human ortholog mutation status on patient survival in specific cancers. The Log-rank test was employed to assess the significance of differences between the curves; *P* < 0.05 was considered statistically significant. Exact sample sizes (*n*) and *P* values were indicated in each panel. (**F**) Kaplan–Meier survival curves illustrating the influence of human ortholog gene expression levels on overall survival in patients with *KRAS*/*NRAS*-driven cancers. The Log-rank test was employed to assess the significance of differences between the curves; *P* < 0.05 was considered statistically significant. Exact sample sizes (*n*) and *P* values were indicated in each panel. Source data are available online for this figure.

To assess the clinical relevance of *Drosophila* tumor suppressors in human cancers with *RAS* activation, we analyzed pan-cancer genomic data obtained from cBioPortal (Cerami et al, 2012; Gao et al, 2013; De Bruijn et al, 2023) and gene expression data from UCSC-Xena (Goldman et al, 2020) (Fig. 6A). Among cancers with high *RAS* family gene mutation rates, *KRAS* mutations predominated in pancreatic adenocarcinoma (PAAD: 63.6%), colorectal adenocarcinoma (COADREAD: 37.4%), non-small cell lung cancer (NSCLC: 19.2%; LUAD: 31.8%; LUSC: 4.5%), uterine corpus endometrial carcinoma (UCEC: 20.0%), uterine carcinosarcoma (UCS: 17.5%), stomach adenocarcinoma (STAD: 16.4%), and esophageal carcinoma (ESCA: 8.8%). *NRAS* mutations were prevalent in skin cutaneous melanoma (SKCM: 28.4%) and thyroid carcinoma (THCA: 7.8%), while *HRAS* mutations were rare (<4%) (Fig. 6B). Based on mutation frequency, PAAD, COADREAD, LUAD, STAD, ESCA, LUSC, UCEC, and UCS were classified as *KRAS*-driven; SKCM and THCA as *NRAS*-driven (Fig. 6B).

Using 14 public databases (Wheeler et al, 2006; Vilella et al, 2009; Kaduk et al, 2017; Emms and Kelly, 2019; Persson et al, 2019; Nevers et al, 2019; Thomas et al, 2022; Fuentes et al, 2022; Amos et al, 2022; Persson and Sonnhammer 2023; Hernández-Plaza et al, 2023; Kuznetsov et al, 2023; Cosentino et al, 2024; Altenhoff et al, 2024), we annotated these ten *Drosophila* tumor suppressors to 18 human orthologs: *scrib/SCRIB*; *l(2)gl*/*LLGL1*/*LLGL2*; *Vps36*/*VPS36*; *Syx7*/*STX7*/*STX12*; *Rabex-5*/*RABGEF1*; *TSG101*/*TSG101*; *fry*/*FRYL*/*FRY*; *fmt*/*PPP6R1*/*PPP6R2*/*PPP6R3*; *emei*/*TMEM161A*/*TMEM161B*; *msn*/*MINK1*/*TNIK*/*MAP4K4* (Fig. 6C). Mutation rates and expression levels of these orthologs were analyzed across *RAS*-driven cancers (Fig. 6D; Appendix Fig. S11A). *SCRIB* exhibited >10% mutation rates in PAAD, ESCA, UCEC, UCS, and SKCM, with downregulation in SKCM. *LLGL1*/*LLGL2* showed the highest mutation rates in UCEC (7.4%) and SKCM (9.7%), respectively, alongside reduced expression. Endocytosis-driven genes (*VPS36*, *STX7*, *STX12*, *RABGEF1*, *TSG101*) were frequently mutated in UCEC/SKCM and downregulated in these tumors, except *STX7* in SKCM. Notably, *FRYL* and *FRY* mutations exceeded 5% in most cancers (excluding PAAD, UCS, THCA), with *FRY* consistently downregulated across various cancers and co-decreased with *FRYL* in LUAD, LUSC, UCEC, and SKCM. These results suggest that the tumor suppressors identified in *Drosophila* model also exhibit high mutation rates and tumor suppressor potential in *KRAS*/*NRAS*-driven human cancers.

We then investigated the prognostic value of these human orthologs. In specific cancer types, single ortholog mutation is sufficient to confer a significant impact on the survival prognosis of patients (Fig. 6E). For instance, *SCRIB* mutations significantly threatened the overall survival (OS) and disease-free survival (DFS) of PAAD patients. Similarly, *FRY* mutations adversely affected the OS, disease-specific survival, and progression-free survival of ESCA patients. Besides, we observed significant positive correlations between the expression of human orthologs and the patient OS across various cancers (Fig. 6F). These findings suggest that the mutations and expression levels of these orthologs may serve as potential prognostic biomarkers in *KRAS*/*NRAS*-driven cancers.

### Cross-species validation of JNK, Toll, Notch, and Hippo pathways oncogenic potentials

**Figure EV5:**
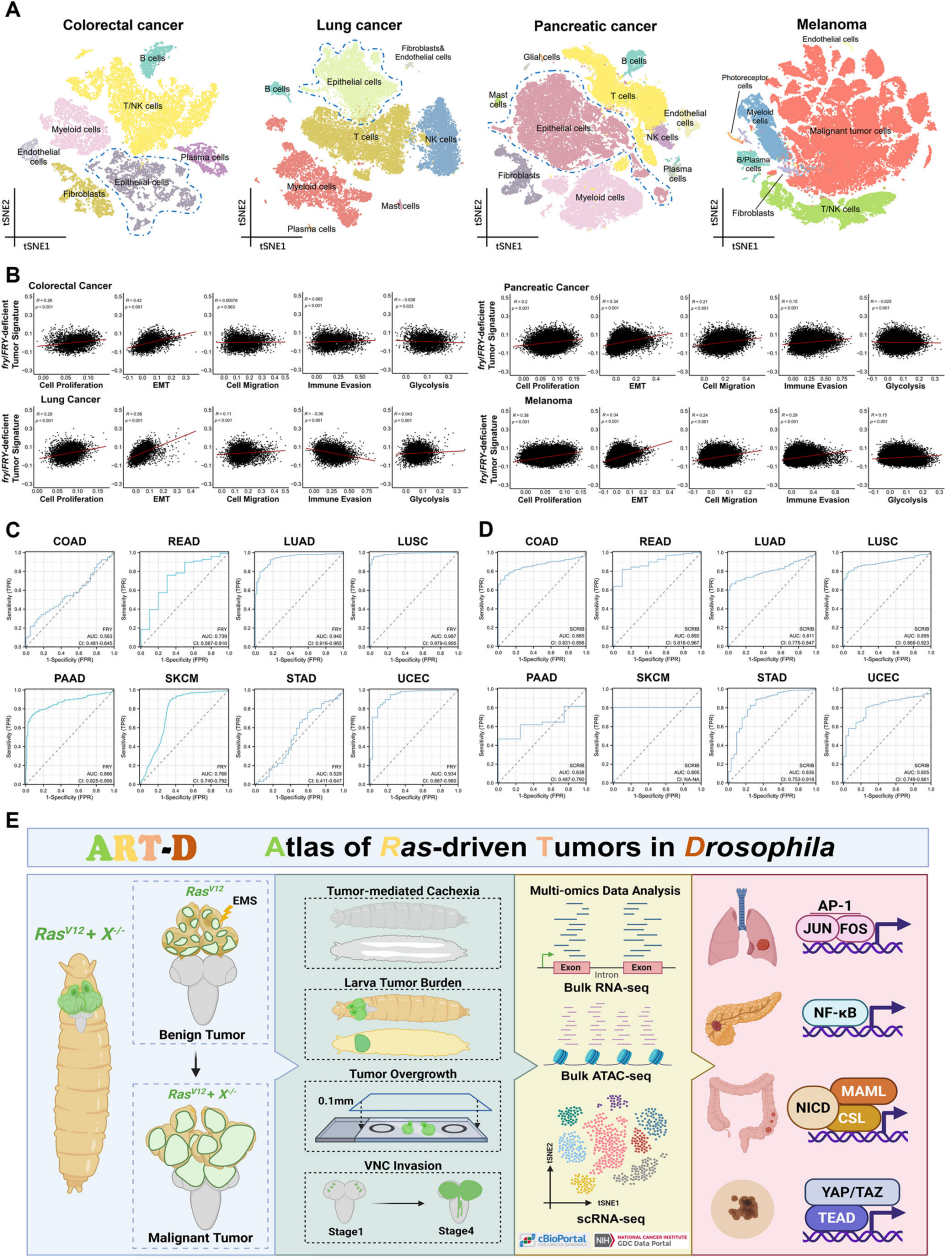
Cross-species insights from ART-D on evolutionarily conserved oncogenic mechanisms. (**A**) Single-cell data analysis for human *KRAS*/*NRAS*-driven cancers. A total of 174,385 high-quality cells were maintained, with 18,727 from colorectal cancer (CRC carrying *KRAS* oncogenic mutations), 25,059 from lung cancer (LUAD), 45,747 from pancreatic cancer (PAAD carrying *KRAS* oncogenic mutations), and 84,825 from melanoma, respectively. The corresponding cell type was annotated. (**B**) The correlation scatter plots illustrate the relationships between *fry*/*FRY*-deficient tumor signature scores and tumor malignancies scores across *KRAS*/*NRAS*-driven cancers, CRC, LUAD, PAAD, and melanoma. These correlations were determined using Spearman’s correlation analysis, with coefficients R and *P* values reported, where *P* < 0.05 indicates statistical significance. (**C**, **D**) The ROC evaluation of *FRY* (**C**) and *SCRIB* (**D**) diagnostic value across *KRAS*/*NRAS*-driven cancers. The AUC values of each cancer were presented with confidence intervals. The AUC higher than 0.85 was considered excellent performance. (**E**) The proposed model of this study. The model illustrated the utilization of ART-D phenotypic malignancies and dynamic transcriptomic profiles to uncover the oncogenic mechanisms driving tumorigenesis in *Drosophila Ras*-driven tumors. Subsequent integrated multi-omics data analysis revealed an evolutionarily conserved role of oncogenic signaling pathways in human *KRAS*/*NRAS*-driven cancers. The cross-species findings demonstrated strong performance in diagnosis and prediction of human cancers. By integrating *Drosophila* genetics with human oncology, ART-D offers a valuable resource for identifying tumor stage-specific therapeutic targets and advancing the treatment of RAS-driven malignancies.

With the idea of JNK, NF-κB/Toll, Notch, and Hippo pathways co-operation may be a shared mechanism to drive tumorigenesis, we integrated publicly available single-cell datasets from patients with *KRAS/NRAS-*driven cancers (CRC, LUAD, PAAD, and melanoma) and obtained 174,385 high-quality cells (Fig. EV5A) (Durante et al, 2020; Lee et al, 2020; Pandiani et al, 2021; Werba et al, 2023; Benitz et al, 2024; Mao et al, 2024). Malignant epithelial cells were isolated based on lineage markers and further stratified into subpopulations (Appendix Fig. S12A–D). We scored the corresponding pathway activity and observed heterogenous regulation of RAS, MAPK, JNK, Toll-like receptor (TLR), Notch, and Hippo signaling across tumors, with subclusters showing co-activation of multi signaling (Appendix Fig. S13A–D and Dataset EV5). This led us to further verify the existence of co-activation of these signaling pathways in *RAS*-activated cells (mimicking cells carrying *KRAS/NRAS* oncogenic mutations). By selecting the subclusters with top-ranking RAS signaling for correlation analysis (Fig. S14A), we found that, within *RAS*-activated cells, RAS signaling positively correlated with MAPK, JNK, TLR, and Notch activation and Hippo inactivation in all cancers, except for TLR in CRC and Notch in PAAD (Fig. S14B). This indicated a conserved co-activation pattern mirroring *Drosophila Ras*^*V12*^*; fry*^*−/−*^ tumors.

**Figure 7:**
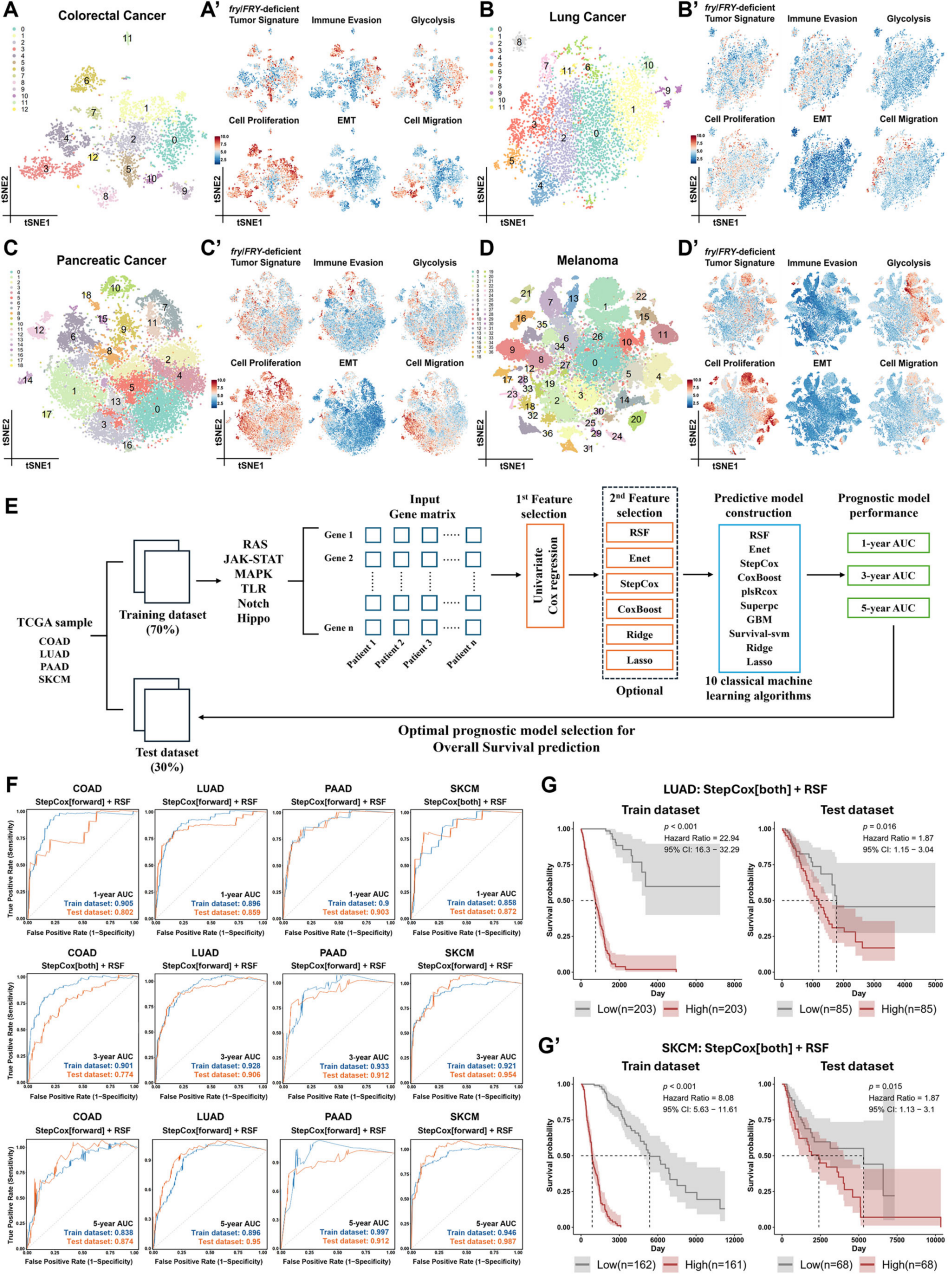
Cross-species validation of evolutionarily conserved oncogenic mechanisms from *Drosophila* to humans. (**A**–**D’**) The tSNE projections of re-clustered malignant tumor cells or tumor epithelial cells derived from *KRAS*/*NRAS*-driven cancers, CRC (**A**), LUAD (**B**), PAAD (**C**), and melanoma (**D**). The tSNE representations depict the scoring of tumor malignancies in re-clustered tumor epithelial cells, CRC (**A’**), LUAD (**B’**), PAAD (**C’**), and melanoma (**D’**). The evaluation of tumor malignancies encompasses *fry*/*FRY*-deficient tumor signature, cell proliferation, EMT, cell migration, immune evasion, and glycolysis. The scoring values were scaled from 0 to 10. (**E**) Workflow for constructing machine learning-based survival prognosis models for *KRAS*/*NRAS*-driven cancer patients using cross-species signature genes. TCGA samples from four cancer types were randomly partitioned into training (70%) and testing (30%) datasets. Employing machine learning approaches, key components of signaling pathways including MAPK, Notch, Hippo, and Toll were selected as candidate genes. Initial feature selection was performed via univariate Cox regression analysis. Subsequently, a total of 117 machine learning algorithm combinations, integrating secondary feature selection and model construction algorithms, were utilized to develop prognosis prediction models for 1-year, 3-year, and 5-year survival outcomes across distinct human *KRAS*/*NRAS*-driven cancers. (**F**) The optimal machine learning model for predicting the survival of 1-year (top), 3-year (middle), and 5-year (bottom) of *KRAS*/*NRAS*-driven cancer patients. The selected machine learning model was denoted under the cancer type. The AUC values of training datasets and test datasets were presented. The AUC higher than 0.85 was considered excellent performance. (**G**–**G’**) The applications of StepCox[both] + RSF model in predicting the overall survival of patients with LUAD (**G**) and SKCM (**G’**), respectively. The Log-rank test was employed to assess the significance of differences between the curves; *P* < 0.05 was considered statistically significant. Exact sample sizes (*n*) and *P* values were indicated in each panel. Source data are available online for this figure.

To illustrate the relationships between co-activation patterns and tumor malignancies, we employed ‘*fry*/*FRY*-deficient tumor signature’, summarized from JNK, TLR, and Notch activation and Hippo inactivation signatures, alongside signatures reflecting cell proliferation, EMT, cell migration, immune evasion, and glycolysis to systematically evaluate the tumor malignancies (Fig. 7A–D’; Dataset EV5). The *fry*/*FRY*-deficient tumor signature positively correlated with RAS signaling across cancers (R > 0.6) (Appendix Fig. S15A), suggesting its potential as a positive marker in *RAS*-hypermutated cancers. Overall, *fry*/*FRY*-deficient tumor signature positively correlated with cell proliferation, EMT, and cell migration (except for CRC) across four cancers. The correlations with immune evasion were negative in LUAD, while positive in PAAD and Melanoma. Although the correlation was extremely weak, this signature was significantly associated with glycolysis in all cancers (Fig. EV5B). Similar results were observed in *RAS*-activated cells, where *fry*/*FRY*-deficient tumor signature was also positively correlated with cell proliferation (except for CRC) and EMT, yet weakly and negatively correlated with glycolysis in all cancers (Appendix Fig. S15B–E). Additionally, the correlations with immune evasion were negative in CRC and LUAD while positive in PAAD and Melanoma, consistent with overall findings, suggesting *FRY* may play a dual role in regulating interactions between tumor cells and immune cells (Appendix Fig. S15B–E). Collectively, these results reveal an evolutionarily conserved transcriptomic signature in *RAS*-driven tumors, wherein the synergistic activation of JNK, TLR, and Notch pathways, coupled with Hippo inactivation, drives malignancy, underscoring their universal relevance in tumor progression.

To further elucidate the tumor-suppressive role of *fry/FRY* in human cancers, we divided six *KRAS*/*NRAS*-driven tumor samples into *FRY*-high and *FRY*-low groups based on the median expression levels of *FRY* for tumor purity and immune infiltration analysis (Appendix Fig. S16A,B). The analysis of tumor purity using ESTIMATE (Yoshihara et al, 2013) revealed that, stromal cell scores, immune cell scores, and Estimate scores were significantly higher in five cancers (COADREAD, LUADLUSC, SKCM, STAD, and UCEC) in *FRY*-high group compared to *FRY*-low group (except for immune cell score in UCEC) (Appendix Fig. S16A). This suggests that the high *FRY* expression may induce increased immune cell infiltration, elevated stromal cell counts, and decreased tumor purity. The detailed infiltration of immune cells was investigated via CIBERSORT (Newman et al, 2015), and we found that low *FRY* expression resulted in common features of increased infiltrations of activated mast cells, CD4 T follicular helper (TFH) cells, activated CD4 memory T cells, and CD8 T cells, along with the decreased M2 macrophages and naïve B cells (Appendix Fig. S16B), suggesting that downregulating *FRY* may activate mast cells to promote tumor overgrowth, while activate TFH cells to support B cell functions and further promote the CD8 T cell anti-tumor immunity. This hypothesis was supported by a murine LUAD model, where T cell-B cell interactions promote the tumor-specific TFH cells to secrete interleukin (IL)-21 to support the anti-tumoral function of CD8 T cells (Cui et al, 2021). However, the interactions between *FRY*-low tumor cells and various immune cells require further explorations to elucidate.

### Machine learning reveals the clinical relevance of DRTs features

To explore the diagnostic value of tumor suppressors in DRTs for human cancers, we conducted Receiver Operating Characteristic (ROC) curve diagnostic analyses on human homologous genes with high mutation rates, *FRY* and *SCRIB*. We found that *FRY* gene exhibited excellent diagnostic performance for LUAD, LUSC, PAAD, and UCEC (Area Under Curve [AUC] >0.85), while *SCRIB* exhibited excellent diagnostic performance in COAD, READ, and LUSC (AUC > 0.85) (Fig. EV5C,D). These results highlight the diagnostic value of *Drosophila* tumor models in clinical applications.

Furthermore, we seek to exploit the highly conserved nature of signaling pathways to establish optimal prognostic prediction models in various *KRAS*/*NRAS* cancers (Fig. 7E). To do this, we randomly divided The Cancer Genome Atlas (TCGA) samples of COAD, LUAD, PAAD, and SKCM into training set (70%) and testing set (30%). Using machine learning methods, we selected *Drosophila* tumor suppressors human orthologs and key components of signaling pathways as candidates (Dataset EV6). After filtering for prognostic features through univariate Cox regression analysis (Dataset EV6), we used 117 combinations of machine learning algorithms from Mime (Liu et al, 2024) to develop prognostic prediction models tailored to different human *KRAS*/*NRAS* cancers. By evaluating the performance of each model by calculating the ROC AUC values for 1-year, 3-year, and 5-year survival times in both training and testing sets (Fig. 7E; Appendix Figs. S17–S20), we elicited the optimal predictive models and found that gene sets based on features of DRTs performed well in predicting 1-year, 3-year, and 5-year survival outcomes for patients with COAD, LUAD, PAAD, and SKCM (AUC > 0.75), with particularly excellent performance for LUAD (AUC > 0.85), PAAD (AUC > 0.9), and SKCM (AUC > 0.85) patients (Fig. 7F).

Notably, the combinations of random forest (RSF) and stepwise Cox (StepCox), StepCox[forward] + RSF and StepCox[both] + RSF, performed well in predicting 1-year, 3-year, and 5-year survival outcomes for COAD, LUAD, PAAD, and SKCM patients. Thereby, we intersected the top five machine learning models ranked by 1-year, 3-year, and 5-year survival prediction performance for each cancer to identify models that performed excellently across all three survival time predictions and used these models to predict OS (Appendix Figs. S17–S20). Using the prediction model generated by the StepCox[both] + RSF algorithm, LUAD and SKCM patients were divided into high-risk and low-risk groups. Results showed that the OS of LUAD and SKCM patients in the high-risk group was significantly lower than that of patients in the low-risk group (Fig. 7G,G’), indicating the StepCox[both] + RSF algorithm can effectively predict the survival prognosis of *KRAS*/*NRAS* cancer patients based on features of DRTs.

## Discussion

In this study, we establish the Atlas of *Ras*-driven Tumors in *Drosophila* (ART-D), a relative comprehensive resource that systematically maps the dynamic progression of malignancy across ten genetic contexts. Using ART-D, we clearly define different stages of tumorigenesis: initial stage, promotion stage, and progression stage. Although all DRTs ultimately develop aggressive phenotypes, their progression trajectories vary significantly. Therefore, by employing robust linear regression, we classify DRTs into three distinct subtypes: Group A, B, and C. By integrating longitudinal phenotypic profiling with transcriptomic and multi-omics analyses, we delineate a cross-species unified framework for tumor progression. We identify coordinated dysregulation of key signaling pathways, JNK, Notch, NF-κB/Toll, and Hippo, emerge as central drivers of malignancy, with this dysregulation showing striking parallels to human cancers harboring frequent *RAS* mutations. Using machine learning to construct optimal prognostic prediction models tailored for human *KRAS*/*NRAS* cancers, we demonstrate that these evolutionarily conserved oncogenic mechanisms hold significant potential for clinical applications (Fig. EV5E).

### Bridging phenotype and genotype

Tumor progression involves dynamic accumulation of genetic and epigenetic alterations that collectively drive phenotypic evolution (Zahir et al, 2020; Flavahan et al, 2017; Biswas and De, 2021). While clinical samples typically offer only static snapshots, the ART-D model enables the real-time dissection of these dynamics in a genetically tractable system. A key innovation of our approach lies in shifting the analytical paradigm from gene-centric to phenotype-centric stratification. Traditional multi-timepoint studies often focus solely on gene expression trends, yet the scarcity of high-quality, longitudinal phenotypic data has historically hindered unbiased correlations between specific gene modules and malignant traits. To address this limitation, the ART-D initiates analysis by stratifying tumor subtypes based on malignant progression phenotypes. By integrating multi-timepoint transcriptomic data with these phenotypic profiles via Weighted Gene Co-expression Network Analysis (WGCNA), we identified gene modules significantly associated with characteristic phenotypes. Subsequent analyses, including gene trend assessment, Over-Representation Analysis (ORA), and gene regulatory network reconstruction, were then performed within these specific modules. This strategy yielded candidate genes and pathways that are not only statistically correlated with progression but also exhibit highly consistent temporal expression patterns. By bridging the gap between static genetic lesions and emergent dynamic phenotypes, ART-D provides a powerful platform for elucidating the molecular mechanisms driving tumor malignancy, an approach rarely utilized in *Drosophila* cancer research.

### Evolutionary conservation and the four-pathway nexus

Importantly, our study uncovers profound conservation of tumorigenic mechanisms between *Drosophila* and humans. Cross-species mapping of tumor suppressors identifies *SCRIB*, *LLGL1/LLGL2*, and *FRY* as key genes frequently dysregulated in *KRAS/NRAS*-mutant human cancers, where their loss correlates with poor patient survival. For example, loss of *SCRIB* synergizing *KRAS*^*G12D*^ induces aggressive lung tumors by activating MAPK signaling (Elsum et al, 2014), consistent with *Drosophila* models (Wu et al, 2010). Besides, *SCRIB* deficiency drives ductal hyperplasia in mammary models and cooperates with *KRAS*^*G12D*^ and *P53* loss to accelerate pancreatic adenocarcinoma metastasis (Bermejo-Rodriguez et al, 2024). Notably, *SCRIB* mis-localization also underlies adaptive resistance to *KRAS*^*G12C*^ inhibitors, underscoring its clinical relevance (Adachi et al, 2023). While earlier work highlighted pairwise pathway interactions (e.g., JNK-Hippo, Hippo-Notch) (Robinson and Moberg, 2011; Ma et al, 2017, 2020; Kong et al, 2021; Yang et al, 2023; Wang et al, 2024, 2026), we identify a four-pathway nexus in *Ras*^*V12*^*; fry*^*−/−*^ tumors: synergistic activation of JNK, NF-κB/Toll, and Notch, alongside Hippo inactivation. Strikingly, single-cell transcriptomics and machine learning investigation of *RAS*-mutant human cancers (pancreatic, colorectal, lung, melanoma) revealed analogous co-activation of these pathways. This suggests an evolutionarily conserved network driving malignancy, revealing pathway interdependencies that may represent therapeutic vulnerabilities.

### Limitations and future directions

Despite providing systematic insights into *Ras*-driven tumorigenesis, several limitations warrant acknowledgment. First, while the *Drosophila* model excels in genetic resolution, it lacks key components of the mammalian tumor microenvironment, such as adaptive immunity and interactions with stromal cells. Incorporating immune responses and co-culture systems into future studies may enhance translational relevance. Second, our current focus on *Ras* mutations combined with specific tumor suppressor losses provides a foundational framework; expanding this to broader oncogenic and tumor-suppressive landscapes will be necessary to uncover more general principles of tumorigenesis. Third, our initial ART-D analysis revealed significant heterogeneity in glucose, glycogen, and fatty acid metabolism across tumor subtypes. This highlights the imperative for future untargeted lipidomics and epigenomics studies across distinct genotypes and stages to elucidate the role of metabolites in sustaining malignancy and mediating tumor-organ crosstalk. Fourth, while we identified conserved synergistic interactions among pro-tumorigenic pathways, the precise molecular mechanisms governing the crosstalk between JNK, Notch, NF-κB, and Hippo remain undefined. Future work will employ tag-specific immunoprecipitation coupled with tandem mass spectrometry (IP-MS/MS) in both human cancer cell lines (overexpressing tagged FRY) and *Drosophila* tissues to identify direct *FRY*/*fry*-interacting proteins. Candidate binders will be validated to delineate the molecular scaffolds facilitating this four-pathway synergy. Finally, functional validation in human models, particularly patient-derived organoids and xenografts, is still lacking, which remains a crucial next step to assess the therapeutic potential of targeting these conserved pathways.

In summary, through systematic investigation of DRTs, we have developed a comprehensive platform, ART-D, that characterizes the progressive phenotypes and dynamic transcriptomic features of *Ras*-driven malignancies. This study reveals conserved cooperative mechanisms among JNK, Notch, NF-κB, and Hippo signaling pathways from *Drosophila Ras* tumors to human *KRAS*/*NRAS* cancers, establishing a systematic framework for cross-species translational cancer research. By integrating *Drosophila* genetics with human oncology, ART-D represents a valuable resource for identifying stage-specific therapeutic targets and advancing the treatment of *RAS*-driven cancers.

## Methods

**Table:** Reagents and tools table

Reagent/resource	Reference or source	Identifier or catalog number
**Experimental models:** ***Drosophila melanogaster***		
FRT40A	Gift from Tian Xu (Westlake University)	N/A
FRT79E	Gift from Tian Xu (Westlake University)	N/A
FRT82B	Gift from Tian Xu (Westlake University)	N/A
FRT40A, UAS-Ras^V12^/CyO	Gift from Tian Xu (Westlake University)	N/A
UAS-Ras^V12^; ri, FRT79E	Gift from Tian Xu (Westlake University)	N/A
UAS-Ras^V12^, ri, FRT79E	Gift from Tian Xu (Westlake University)	N/A
UAS-Ras^V12^, FRT82B	Gift from Tian Xu (Westlake University)	N/A
Sco/Cyo	Gift from Tian Xu (Westlake University)	N/A
TM3 Sb/TM6B Tb	Gift from Tian Xu (Westlake University)	N/A
Sp/Cyo; Sb/TM6B Tb	Gift from Tian Xu (Westlake University)	N/A
Sp; Sb/S-T	Gift from Tian Xu (Westlake University)	N/A
ey-Flp1; tub-Gal80 FRT40A; act>y + >Gal4, UAS-GFP	Gift from Tian Xu (Westlake University)	N/A
ey-Flp1; act>y + >Gal4, UAS-GFP; tub-Gal80, ri FRT 79E	Gift from Tian Xu (Westlake University)	N/A
ey-Flp1; Act>y + >Gal4, UASGFP; FRT82B, tub -Gal80	Gift from Tian Xu (Westlake University)	N/A
UAS-Ras^V12^, FRT82B, scrib^1^/TM6B Tb	Gift from Tian Xu (Westlake University)	N/A
l(2)gl^4^, FRT40A, UAS- Ras^V12^/S-T	Gift from Tian Xu (Westlake University)	N/A
UAS-Ras^V12^; Vps36^2586^, FRT79E/TM6B TB	Lab resource	N/A
UAS-Ras^V12^; Syx7^3119^, FRT79E/TM6B TB	Lab resource	N/A
UAS-Ras^V12^; Rabex-5^3672^, FRT79E/TM6B TB	Lab resource	N/A
UAS-Ras^V12^/CyO; TSG101^P26^, ri FRT79E/TM6B	Gift from Tatsushi Igaki (Kyoto University)	N/A
UAS-Ras^V12^; fry^3792^, FRT79E/TM6B TB	Lab resource	N/A
UAS-Ras^V12^; fmt^3355^, FRT79E/TM6B Tb	Lab resource	N/A
UAS-Ras^V12^; emei^566h^, FRT79E/TM6B Tb	Lab resource	N/A
UAS-Ras^V12^; msn^3208^, FRT79E/TM6B	Lab resource	N/A
w^1118^	Gift from Tian Xu (Westlake University)	N/A
GMR-Gal4^S^	Gift from Tian Xu (Westlake University)	N/A
UAS-Egr^R^; GMR-Gal4^W^/SM6-TM6B	Gift from Tian Xu (Westlake University)	N/A
pnr-Gal4/TM6B Tb	Gift from Tian Xu (Westlake University)	N/A
dpp-Gal4, UAS-GFP/TM6B	Gift from Tian Xu (Westlake University)	N/A
UAS-fry.RNAi	Vienna Drosophila Resource Center	VDRC40309
UAS-myc-fry	Bloomington Drosophila Stock Center	BDSC32105
UAS-scrib.RNAi	Gift from Tian Xu (Westlake University)	N/A
UAS-Notch^act^	Gift from Hai Huang (Zhejiang University)	N/A
UAS-Yki^S168A^	Bloomington Drosophila Stock Center	Cat# 28816
UAS-Ras^V12^, UAS-dTAK1^DN^	Gift from Shian Wu (Nankai University)	N/A
UAS-hep·RNAi	Tsinghua Fly Center	TH201501052.S
UAS-Bsk^DN^	Gift from Tian Xu (Westlake University)	N/A
UAS-Jun^DN^, UAS-Ras^V12^	Gift from M. Bienz (MRC Laboratory of Molecular Biology)	N/A
UAS-Fos^DN^, UAS-Ras^V12^	Gift from M. Bienz (MRC Laboratory of Molecular Biology)	N/A
UAS-Wts	Gift from Shian Wu (Nankai University)	N/A
UAS-Toll.RNAi	Vienna Drosophila Resource Center	VDRC330125
UAS-Dorasl.RNAi	Vienna Drosophila Resource Center	V45996 LX519
UAS-Dif.RNAi	Vienna Drosophila Resource Center	V30578 LX589
UAS-Ser.RNAi	Vienna Drosophila Resource Center	VDRC27172
UAS-Delta^DN^	Bloomington Drosophila Stock Center	BDSC26697
UAS-Notch.RNAi	Vienna Drosophila Resource Center	VDRC1112
**Antibodies**		
Rabbit polyclonal anti-Phospho-Histone H3 (Ser10)	Cell Signaling Technology	Cat# 9701
Mouse monoclonal anti-γH2AV, phosphorylated	Developmental Studies Hybridoma Bank	Cat# UNC93-5.2.1
		RRID: AB_2618077
Rabbit monoclonal anti-Phospho-p44/42 MAPK (Erk1/2) (Thr202/Tyr204)	Cell Signaling Technology	Cat# 4370
Mouse monoclonal anti-Mmp1	Developmental Studies Hybridoma Bank	Cat# 3A6B4; RRID: AB_579780,
		Cat# 3B8D12; RRID: AB_579781,
		Cat# 5H7B11; RRID: AB_579779
Rabbit polyclonal anti-active JNK	Promega	Cat# v2973;
		RRID: AB_3391716
Mouse monoclonal anti-Wg	Developmental Studies Hybridoma Bank	Cat# 4D4
		RRID: AB_528512
Mouse monoclonal anti-NICD	Developmental Studies Hybridoma Bank	Cat# C17.9C6
		RRID: AB_528410
Mouse monoclonal anti-DIAP1	Gift from Bruce Hay (California Institute of Technology)	N/A
Mouse monoclonal anti-Delta, extracellular domain	Developmental Studies Hybridoma Bank	Cat# C594.9B
Rabbit polyclonal anti-Yki	Gift from Duojia Pan (UT Southwestern Medical Center)	N/A
Mouse anti-dMyc	Developmental Studies Hybridoma Bank	Cat# P4C4-B10
		RRID: AB_2753231
Mouse monoclonal anti-Dorsal	Developmental Studies Hybridoma Bank	Cat# anti-dorsal 7A4
		RRID: AB_528204
Mouse monoclonal anti-Relish	Developmental Studies Hybridoma Bank	Cat# anti-Relish-C 21F3
		RRID: AB_1553772
**Chemicals, enzymes and other reagents**		
Fetal bovine Serum	Gibco	Cat# C11875500CP
Normal Goat Serum	Solarbio	Cat# SL038
DAPI	Beyotime	Cat# C1002
Trizol	Thermo Fisher	Cat# 15596026CN
**Oligonucleotides and other sequence-based reagents**		
PCR for msn-F	AATGTGAACATGAACAATGCCG	N/A
PCR for msn-R	ATGGTTTACATCATATGGATAAAAC	N/A
PCR for fmt-F	ACGCATTACTTTGTGAGAGCCG	N/A
PCR for fmt-R	ACTCGACTGCAAGTTTGTCGC	N/A
PCR for emei-F	GGTCACACTGCCCGCACAAAAG	N/A
PCR for emei-R	AAGCCGGGGAAGGTGAACAATG	N/A
PCR for fry-F	AAATCCTGGTGCCTGTGGCGG	N/A
PCR for fry-R	CGAGTTGTCTGTAAAAAGAAGTATC	N/A
PCR for Vps36-F	ATGAATCGCTTCGCTTATGTGG	N/A
PCR for Vps36-R	TTAGTCCCGGCCTAGCAGAAGATTTGG	N/A
PCR for Syx7-F	AAAAGCGAATGTGATATAAGTGGG	N/A
PCR for Syx7-R	TCCAAAGGATTGGGTGGGGAATT	N/A
PCR for Rabex-5-F	CAACGCTGGCAGCAGTAGAA	N/A
PCR for Rabex-5-R	TTCACGTCACTGTCCTCATCGG	N/A
**Software**		
Prism 9.0	GraphPad Software	RRID:SCR_002798
ImageJ	https://imagej.net/	RRID:SCR_003070
cellSens Dimension	https://lifescience.evidentscientific.com.cn/zh/software/cellsens/	N/A
FV31S-SW	https://www.microscope.healthcare.nikon.com/	N/A
OlyVIA 4.1.1	https://lifescience.evidentscientific.com.cn/zh/downloads/detail-iframe/?0[downloads][id]=847254102	N/A
ZEN 3.4 (blue edition)	https://www.zeiss.com/microscopy/en/service-support/downloads.html	N/A
BioRender	https://www.biorender.com	N/A
Cytoscape v3.10.4	https://cytoscape.org/	N/A
**Online webtools**		
PANGEA	https://www.flyrnai.org/tools/pangea/web/home/7227	N/A
STRING	https://string-db.org/	N/A
GEPIA2	http://gepia2.cancer-pku.cn/#index	N/A
cBioProtal	https://www.cbioportal.org/study/summary?id=5c8a7d55e4b046111fee2296	N/A
UCSC-Xena	https://xenabrowser.net/datapages/	N/A
**Deposited data**		
RNA-seq raw data ATAC-seq raw data	https://www.ncbi.nlm.nih.gov/bioproject/PRJNA1209517	PRJNA1209517
**Public data acquisition**		
Pan-cancer genomic mutation landscapes	https://www.cbioportal.org/study/summary?id=5c8a7d55e4b046111fee2296	TCGA PanCancer Atlas Studies from cBioProtal
Gene expression data and clinical data for TCGA samples	https://xenabrowser.net/datapages/	UCSC-Xena
scRNAseq data of melanoma	https://www.ncbi.nlm.nih.gov/geo/query/acc.cgi?acc=GSE139829	GSE139829
	https://www.ncbi.nlm.nih.gov/geo/query/acc.cgi?acc=GSE138665	GSE138665
scRNAseq data of colorectal cancer	https://www.ncbi.nlm.nih.gov/geo/query/acc.cgi?acc=GSE132465	GSE132465
	https://www.ncbi.nlm.nih.gov/geo/query/acc.cgi?acc=GSE144735	GSE144735
scRNAseq data of PAAD	https://www.ncbi.nlm.nih.gov/geo/query/acc.cgi?acc=GSE205013	GSE205013
scRNAseq data of LUAD	10.6084/m9.figshare.25027181	figshare.25027181

### Fly husbandry and genetics

*Drosophila* stocks and crosses were maintained on standard food at 25 °C under standard laboratory conditions (25 °C, 12:12 h light: dark). Standard cornmeal-yeast food containing 50 g corn flour, 24.5 g dry yeast, 9 g agar, 7.25 g white sugar, 30 g brown sugar, 4.4 ml propionic acid, 12.5 ml ethanol and 1.25 g nipagin per liter. A comprehensive list of the utilized *Drosophila* strains is provided in the reagents and tools table.

### EMS screen and mutant identification

EMS Screen details were described previously (Lee and Luo, 1999; Ma et al, 2017). A benign tumor model carrying the *Ras*^*V12*^ was established in the eye-antennal imaginal discs of *Drosophila* using the MARCM system (Lee and Luo, 1999). Single-gene mutations were induced in parent *Drosophila* (*UAS-Ras*^*V12*^; *79E*) through EMS treatment. After crossing with *79E* tester flies, homozygous mutant clones were generated specifically in the *Ras*^*V12*^ mutant cells. Novel tumor suppressor genes were identified based on the size of GFP-labeled regions and the tumor burden observed in the eye-antennal imaginal discs of the *Drosophila* larvae. By crossing *Drosophila* deficiency lines with mutant fly strains of candidate tumor suppressor genes, when homozygous lethality occurs, mutant alleles of specific genes are used for precise localization. For each mutant, *Drosophila* lines were examined by Sanger sequencing. Genomic DNA was rapidly extracted using squish buffer (10 mM Tris-Cl, pH 8.2, 1 mM EDTA, 25 mM NaCl, 200 µg/ml Proteinase K) and then added to the PCR reaction to amplify the target gene segment. The mutation sites were subsequently confirmed by Sanger sequencing. The primers used for PCR were listed in the reagents and tools table.

### Phenotypic malignancies acquisition

The four representative phenotypic characteristics of normal tissue, *Ras* benign tumors, and various malignant tumors were harvested at different time points (5.5 d ± 6 h, 9.5 d ± 6 h, and 13.5 d ± 6 h AEL) as indicated. Tested samples were randomly selected with proper biological replicates for quantifications (*n* > 20), whole larvae were photographed using a fluorescent stereomicroscope (Olympus, SZX16) with GFP fluorescence or bright field to quantify larval tumor burden and tumor-mediated cachexia. The ratio of GFP regions to the whole larva highlights the tumor growth burden on the host, while the overall transparency of the larvae reflects the degradation of fat bodies and muscles due to cachexia. Paired larvae were dissected in cold PBS, and the eye-antennal discs or tumors were separated from the VNC and brain tissues as much as possible. The eye-antennal discs or tumors were placed in a chamber with a 0.1 mm height to assess overall tissue volume or GFP region volume, quantifying tumor overgrowth. The relative occupation rate of the VNC, as a neighboring tissue, reflects the invasion ability of tumor cells. Quantifications of these images were performed using ImageJ 9.0 software.

### Larval pupariation rate quantification

Roughly ten males were crossed with twenty females per vial, and the vials were changed daily. The number of pupae per vial was counted at the indicated time points. The mean number of pupae was calculated for each genotype by averaging the results from three independent replicate vials.

### Larval cell migration, adult fly thorax closure, and eye size quantification

A cell migration model was established using *dpp*-*Gal4*; *UAS*-*GFP* to knock down *scrib*, thereby inducing a JNK-dependent cell migration phenotype. Subsequently, *fry* was either ectopically expressed or knocked down to assess phenotypic differences. Given that fly thorax closure is a JNK-dependent developmental process, *pnr*-*Gal4* was employed to knock down *fry* expression and investigate its regulatory role under physiological conditions. In the adult fly eye, *fry* expression was manipulated using *GMR*-*Gal4* alone or in combination with *UAS*-*Egr* to elucidate the regulatory function of *fry* in JNK signaling under both physiological and pathological conditions. Individuals were randomly selected for observation with proper biological replicates (n > 20). Imaging was performed using an Olympus SZX16 fluorescent stereomicroscope or a Zeiss LSM900 inverted confocal microscope. Scale bars were added using Olympus cellSens Dimension or ZEN 3.4 (blue edition) software. For the quantification of adult eye phenotypes, male and female flies were analyzed separately using ImageJ 9.0.

### Immunofluorescent staining and imaging

The imaginal discs of various genotypes were dissected from the third-instar larvae on the indicated days AEL. Larvae were randomly selected for dissections performed in cold PBS, and the discs or tumors were fixed with 4% paraformaldehyde for 15 min at room temperature with gentle shaking. Subsequently, the samples were washed three times with PBS containing 0.1% Triton X-100 (PBST) for 5 min each. After fixation and washing, samples were blocked in 10% goat serum in PBST for 30 min, followed by incubation with primary antibody (1:200) at 4 °C overnight. The primary antibodies used in this study are listed in the reagents and tools table. Samples were then washed three times with PBST for 10 min each at room temperature, followed by incubation with secondary antibody and DAPI (1:500 in PBST) for 2 h at room temperature. This was followed by three additional washes with PBST. The imaging of all samples was conducted using either an Olympus FV3000-BX63 confocal microscope (inverted), an Olympus Spin SR10 confocal microscope (inverted), or a Zesis LSM900 confocal microscope (inverted) equipped with ×10, ×20 or ×40 objectives. Images were processed using Olympus FV31S-SW, OlyVIA 4.1.1, or ZEN 3.4 (blue edition) to add scale bars, ImageJ 9.0 for quantification, and Photoshop CS5 (Adobe) for image merging and resizing.

### Regression analysis and PCA of *Drosophila Ras*-driven tumors

A robust linear regression model (“rlm” method) from MASS (v7.3-65) was applied to construct regression models for the numerical variables of tumor burden, tumor overgrowth, and tumor-induced cachexia in *Drosophila Ras*-driven malignant tumor progression. Scatter plots were generated using ggplot2 (v3.4.4), and regression equations were added using ggpmisc (v0.6.1). Based on robust linear regression equations of different tumor types, the slopes, intercepts, and adjusted *R*^2^ values from both individual tumor types and the overall *Ras*-driven tumor robust regression model were extracted for PCA analysis. The ten *Drosophila* tumor models were projected onto the two-dimensional interface formed by PC1 and PC2, and K-means clustering was applied to classify DRTs into distinct subtypes (*n* = 3). Additionally, the contributions of PC1 and PC2 to numeric variables reflecting tumor malignancy progression, including larval tumor burden, tumor overgrowth, and tumor-induced cachexia, were visualized using heatmaps generated with pheatmap (v1.0.12) and RColorBrewer (v1.1-3).

### Total RNA extraction

For control groups, a total of 200 eye-antennal discs (*wildtype* group, 3 biological replicates) or benign tumors (*Ras* group, 3 biological replicates) were harvested at the late stage of third instar larvae for total RNA extraction by using Trizol (Invitrogen, Carlsbad, CA) according to manual instruction. For case groups, a total of 100 eye-antennal malignant tumors from *Ras*^*V12*^*//X*^*−/−*^ (case group, 3 biological replicates) were harvested at three different time points (5.5 d ± 6 h, 9.5 d ± 6 h, and 13.5 d ± 6 h AEL) for total RNA extraction with the same procedures. The RNA samples were subjected to Nanodrop 2000 (Thermo Fisher Scientific) and 2100 bioanalyzer (Agilent) for quality control. Samples with concentrations higher than 30 ng/µl, OD260/OD230 ratios between 2.0 and 2.5, and RNA integrity number (RIN) values greater than 8.0 were considered as qualified samples for further library preparation.

### Library preparation and RNA-seq

For each sample, a total amount of 1–3 μg RNA was used as the input for library preparation by strictly following the standard protocol of VAHTS Universal V6 RNA-seq Library Prep Kit for Illumina^®^ (NR604-01/02). Briefly, mRNA was purified by using poly-T oligo-attached magnetic beads from total RNA. The short fragments of mRNA were obtained by adding the fragmentation buffer. After the first strand of cDNA was synthesized by using random hexamer primer and RNase H, the second strand synthesis was performed subsequently by using buffer, dNTPs, DNA polymerase I, and RNase H. And then, the double-stranded cDNA was purified by using QiaQuick PCR kit or AMPure P beads. The purified products of each sample were repaired at the end, added tail, and connected to the sequencing connector, then the appropriate fragment size was selected, and the final cDNA library was obtained by PCR amplification for further sequencing performed on Illumina NovaSeq 6000 platform with NovaSeq 6000 S4 Reagent kit V1.5.

### RNA-seq data processing and functional annotation

All eye-antennal disc/tumor transcriptomic data were processed and analyzed as follows. FastQC (v0.11.8) (https://github.com/s-andrews/FastQC) software was applied for data quality control and reads statistics. The high-quality reads (Q30 > 90%) were mapped to the Ensemble (Martin et al, 2023) *Drosophila melanogaster* reference genome (Drosophila_melanogaster.BDGP6.32.108) by using Hisat2 (Kim et al, 2019), followed by the gene feature counting with default settings by using HTSeq (Anders et al, 2015; Putri et al, 2022). The DEGs identification and ORA were conducted in R (v4.2.0) after removing batch effect using the *ComBat*_seq function of sva (v3.46.0) package (Leek et al, 2012). The gene count matrix after batch effect removal was provided (Dataset EV2). Genes meeting the criteria of |Fold-Change | >1.5 and FDR < 0.05 were identified as DEGs with the “RLE” method of edgeR (v3.40.2) (Robinson et al, 2010). The gene id transformation was performed by using biomaRt (Durinck et al, 2009). The ORA of DEGs were performed against terms in various databases, including FlyBase signaling pathway (https://flybase.org), Kyoto Encyclopedia of Genes and Genomes (KEGG) database (Kanehisa and Goto, 2000; Kanehisa et al, 2017), Gene Ontology (GO) consortium (Ashburner et al, 2000), and Drosophila RNAi Screening Center (DRSC) PathON database (https://fgr.hms.harvard.edu/) by using PANGEA webtools (Hu et al, 2023) or clusterProfiler package (Yu et al, 2012). Data visualization was relied on limma (v3.54.2), pheatmap (v1.0.12), RColorBrewer (v1.1-3), and ggplot2 (v3.4.4) packages.

### Linking phenotypic data with transcriptomic data

WGCNA (v1.73) was applied to identify modules of highly correlated genes and the modules significantly associated with sample phenotypic traits (Langfelder and Horvath, 2008). After pre-process of sample data to remove the outliers, the optimal soft threshold was chosen to convert the correlation matrix into an adjacency matrix. Modules were generated by using the *blockwiseModules* function. The cluster dendrograms were recorded and plotted to show the correlations of different modules. The Pearson correlation analysis was performed to screen the modules that were significantly associated with parameters of four representative phenotypic characteristics or genotypes. A *P* value less than 0.05 was considered significant. The modules of interest were selected for further analysis.

### Time-series analysis of transcriptomic data

The gene count matrix was transformed into Log2(cpm+1) for input. Two packages, Mfuzz (v2.58.0) and ClusterGVis (v0.1.2), were utilized to illustrate the gene dynamic change patterns across five pseudotime points, simulating the process of tumor progression from normal to malignant. For Mfuzz (Kumar and Futschik, 2007), the m value was estimated using the *mestimate* function, and genes with memberships below 0.2 were excluded. For ClusterGVis (https://github.com/junjunlab/ClusterGVis), *K*-means clustering was applied to generate 10 clusters, and the dynamic gene expression patterns were visualized using heatmaps with key genes highlighted.

### Protein and protein interaction network

Genes of interest were used as input to construct PPI networks using the STRING database (Mering, 2003; Szklarczyk et al, 2025), which were subsequently visualized in Cytoscape v3.10.4 software (Killcoyne et al, 2009). The selected genes were evaluated using 11 algorithms within the cytoHubba plugin, and the Bottleneck algorithm was chosen to identify hub genes (Chin et al, 2014). The top 25 hub genes were then selected.

### Transcription factors enrichment analysis

The enrichment of transcription factors was conducted by using RcisTarget (v1.26.0) (Aibar et al, 2017). The motifAnnotations_dmel was adopted for the annotations of motifs to TFs. The rankings of motif were set by using *importRankings* function to import the species corresponding file (dm6_v10_clust.genes_vs_motifs.rankings.feather) downloaded online (https://resources.aertslab.org/cistarget/).

### GSVA and ssGSEA

A total of 15 signaling pathways were selected, including lnR, Hippo, Notch, Toll, JAK_STAT, TNF-α_Eiger, FGFR, Hedgehog, Pvr, Wnt, Sevenless, Imd, Torso, BMP, and EGFR signaling pathways. For each signaling pathway, the list of positive regulators, negative regulators, and all members were collectively downloaded from FlyBase (https://flybase.org) (Thurmond et al, 2019; Gramates et al, 2022). The GSVA (Hänzelmann et al, 2013) and ssGSEA (Subramanian et al, 2005; Barbie et al, 2009) were performed locally using the gene lists with GSVA (v1.46.0). The average values of three replicates from each sample were subjected to Pearson correlation analysis with the average values of the basic phenotypes of tumor progression. A *P* value less than 0.05 was considered statistically significant.

### Library preparation and ATAC-seq

Either disc or tumor samples were digested into a single-cell suspension using the sCelLive^TM^ Tissue Dissociation Mix (Singleron) as previously described (Zhao et al, 2025). Cell viability and concentration were assessed using Trypan Blue staining (Cat#C0040, Solarbio) and Acridine Orange/Propidium Iodide (AO/PI) staining (#RE010212, Countstar). For each sample, a total of 2 × 10^5^ cells were used for ATAC-seq library preparation. The library preparation was conducted using the Hyperactive ATAC-Seq Library Prep Kit for Illumina (Vazyme, TD711) according to the manufacturer’s guidelines. Cells were centrifuged at 500 × *g* at 4 °C for 5 min, resuspended, and washed twice with 50 μl of TW Buffer. The cells were then resuspended in 50 μl of cold lysis buffer and incubated on ice for 5 min to isolate the nuclei. Following a 10 min centrifugation at 500 × *g* at 4 °C, the nuclei were incubated in a 50 μl fragmentation reaction mix at 37 °C for 30 min. The desired DNA was extracted using 100 μl of ATAC DNA Extract Beads, followed by two washes with 200 μl of fresh 80% ethanol and a final elution with 26 μl of nuclease-free ddH_2_O. Library amplification was performed by adding 60 μl of PCR mix to 20 μl of DNA product, following the program: 72 °C for 3 min; 95 °C for 3 min; 12 cycles of 98 °C for 10 s, 60 °C for 5 s, and 72 °C for 1 min; and a hold at 12 °C. The amplified ATAC-Seq library was further purified using ATAC DNA Clean Beads, followed by two washes with 200 μl of fresh 80% ethanol and a final elution with 22 μl of nuclease-free ddH_2_O. The final ATAC-seq libraries were sequenced using the Illumina NovaSeq 6000 platform.

### ATAC-seq data processing and analysis

The analytic procedures of ATAC-seq data were referred to recommended pipelines (Yan et al, 2020). The sequencing data obtained from the Illumina platform was processed by using FastQC (v0.11.8) software for quality control. Adapters were removed and reads were trimmed by using Cutadapt (v1.18) (Martin, 2011). The clean reads were first mapped to the Ensemble (Martin et al, 2023) *Escherichia coli* (*E. coli*) reference genome (GCF_000005845.2_ASM584v2_genomic) by using Bowtie2 (v2.4.2) (Langmead and Salzberg, 2012), the unrecognized reads were subsequently mapped to *Drosophila melanogaster* reference genome (Drosophila_melanogaster.BDGP6.32.108) with the parameters “--end-to-end --very-sensitive --no-mixed --no-discordant --phred33 -I 0 -X 2000 --no-unal”. After using SAMtools (v1.11) (Danecek *et al*, 2021) to transform SAM files into BAM files, Picard (v2.25.1) (https://broadinstitute.github.io/picard/) was applied to remove duplicates, and the reads were selectively maintained when meeting “mapping_quality >= 20”. The final BAM files of same genotype among the replicates were merged into one for peak calling, which is performed by using Model-based Analysis of ChIP-seq (MACS2, v2.2.6) (Zhang et al, 2008) with settings “-g dm -f BAMPE --nomodel --shift -100 --extsize 200”. Peaks were annotated by using ChIPseeker (Yu et al, 2015) package in R (v4.2.0), and the BAM files were transformed into bigwig files for signal enrichment visualization by using the *bamCoverage* function of BEDTools (v2.30.0) (Quinlan and Hall, 2010) with settings “--binSize 20 --normalizeUsing RPKM”. The average normalized signal values of peaks in *Drosophila* genome region were calculated and visualized by using DeepTools (v3.5.1) (Ramírez et al, 2016) and IGV (Robinson et al, 2011), respectively. The binding motifs were identified by using the *findMotifsGenome* or *findMotifs* functions of Homer (v4.11) (Heinz et al, 2010) according to conditions.

### Pan-cancer evaluation of *Drosophila* tumor suppressor ortholog mutation

The *Drosophila* tumor suppressor orthologs were identified using various databases (Wheeler et al, 2006; Vilella et al, 2009; Kaduk et al, 2017; Nevers et al, 2019; Emms and Kelly, 2019; Persson et al, 2019; Fuentes et al, 2022; Amos et al, 2022; Thomas et al, 2022; Hernández-Plaza et al, 2023; Kuznetsov et al, 2023; Persson and Sonnhammer 2023; Altenhoff et al, 2024; Cosentino et al, 2024). As a result, a total of 18 human orthologs were identified corresponding to ten *Drosophila* tumor suppressors. The mutation rates of *RAS* family genes (*KRAS*, *NRAS*, and *HRAS*) and *Drosophila* tumor suppressor orthologs were analyzed by using the pan-cancer genomic data obtained from cBioPortal (Cerami et al, 2012; Gao et al, 2013; De Bruijn et al, 2023). Samples were divided into two groups based on the target gene mutations, then prognostic impact was evaluated by performing Kaplan–Meier analyses with Log-rank test.

### Pan-cancer expression and prognosis of *Drosophila* tumor suppressor orthologs

The pan-cancer expression levels of 18 *Drosophila* tumor suppressor orthologs were investigated using a Log_2_(TPM + 1) transformed gene expression matrix, downloaded from UCSC-Xena (Goldman et al, 2020), encompassing TCGA (The Cancer Genome Atlas Research Network et al, 2013) and The Genotype-Tissue Expression (GTEx) (The GTEx Consortium et al, 2013) samples. The prognostic value of these human orthologs was assessed locally through Kaplan–Meier analyses with Log-rank tests, or via GEPIA2 online (Tang et al, 2019), employing appropriate cut-offs by using the *surv_cutpoint()* function of survminer package to delineate high and low group samples.

### Immune infiltration analysis

The immune infiltration of tumor samples from TCGA *KRAS*/*NRAS*-driven cancers were evaluated by using ESTIMATE (Yoshihara et al, 2013) and CIBERSORT (Newman et al, 2015), respectively. Tumor samples obtained from TCGA were divided into two groups based on the medial expression level of *fry* ortholog gene *FRY*. The tumor purity was evaluated by using ESTIMATE (Yoshihara et al, 2013). The relative infiltration level of various immune cell subpopulations were calculated by using CIBERSORT (Newman et al, 2015). The relationships between *FRY* expression and immune infiltrations in *KRAS*/*NRAS*-driven cancers were calculated via Wilcoxon rank-sum test.

### scRNA-seq data analysis

Six scRNA-seq datasets encompassing four cancer types: pancreatic cancer (PAAD), melanoma (SKCM), colorectal cancer (CRC), and lung adenocarcinoma (LUAD) were collected (Durante et al, 2020; Lee et al, 2020; Pandiani et al, 2021; Werba et al, 2023; Benitz et al, 2024; Mao et al, 2024). Each dataset included tumor cells, stromal cells, and immune cells. The scRNA-seq datasets were processed and analyzed using the Seurat package (v4.0.0) in R (v4.2.0). The high-quality cells were defined as follows: for CRC and LUAD, cells satisfied the criteria “1500< *nCount_RNA* < 40000; 200 < *nFeature_RNA* < 6000; the proportion of mitochondrial genes <15%” were maintained; for SKCM, cells satisfied the criteria “200< *nFeature_RNA* < 8000; the proportion of mitochondrial genes <10%” were maintained; for PAAD, cells satisfied the criteria “500 < *nFeature_RNA*; 1500 < *nCount_RNA*; the proportion of mitochondrial genes <15%” were maintained. The scRNA-seq data from different patients or different datasets were integrated by using canonical correlation analysis (CCA) with the *FindIntegrationAnchors* and *IntegrateData* functions (Cao et al, 2022). *NormalizeData* and *ScaleData* functions were used to normalize and scale gene counts, *FindVariableFeatures* function was applied to select 2500 top variable genes for PCA, and further dimensionality reduction using tSNE with *RunTSNE* function (Van der Maaten and Hinton, 2008). Primary cell clustering was performed using the Louvain algorithm with an appropriate resolution. The resulting Louvain clusters were visualized in a two-dimensional tSNE plot, and each cluster was annotated to known biological cell types using canonical marker genes as indicated. The cell types of interest were extracted by using *subset* function, followed by re-performing dimensionality reduction and cell clustering. The visualization of scRNA-seq data was dependent on dplyr (v1.1.4), ggsci (v3.0.0), cowplot (v1.1.1), gridExtra (v2.3), ggpubr (v0.6.0), reshape (0.8.9), tidyverse (2.0.0), RColorBrewer (v1.1-3), and ggplot2 (v3.4.4) packages.

### Scoring and correlation analysis of signaling pathways and tumor malignancies

The scoring of different signaling pathways and tumor malignancies was conducted by using the *AddModuleScore* function with the gene list provided (Dataset EV5). The *fry*/*FRY*-deficient tumor signature encompassed genes from JNK, TLR, Notch activation and Hippo inactivation. The signatures reflecting tumor malignancies were mainly constructed by gene sets recorded in the MSigDB (Liberzon et al, 2015) and were integrated with gene sets reported in literatures, including cell proliferation (GO_0008283), cell migration (WU_CELL_MIGRATION (Wu et al, 2008)), EMT (LIANG_EMT (Liang et al, 2021), KOHN_EMT_EPITHELIAL (Rajapakse et al, 2018), and HALLMARK_EMT), immune evasion (GOURMET_IMMUNE_EVASION (Gourmet et al, 2024)), and glycolysis (HALLMARK_GLYCOLYSIS). The *unique* function was applied to filter the replicated genes. The correlations between two signature gene sets were assessed by using Spearman’s correlation analysis, with coefficients R and *P* values reported, where *P* < 0.05 indicates statistical significance.

### The diagnostic value of *Drosophila* tumor suppressor orthologs

The UCSC-Xena TCGA samples from COAD, READ, LUAD, LUSC, PAAD, SKCM, STAD, and UCEC patients were utilized to evaluate the diagnostic value of Drosophila tumor suppressor orthologs. The gene expression matrix, transformed using log2(TPM + 1), was employed, and the diagnostic accuracy of these orthologs was assessed using the pROC (v1.18.0) package to construct ROC curves (Robin et al, 2011).

### Optimal prognostic models developed via 117 machine learning combinations

The TCGA samples of COAD, LUAD, PAAD, and SKCM were divided into training dataset (70%) and test datasets (30%) by using the *createDataPartition* function. The candidate gene signature gene list comprises members from JAK-STAT, MAPK, RAS, Hippo, Notch, and TLR signaling pathways (Dataset EV5). The development of prognostic models via machine learning was based on Mime package (Liu et al, 2024). The univariate Cox regression analysis was conducted to identify prognostic features as input variables, in which *P* values less than 0.05 considered statistically significant (Dataset EV6). These features were then subjected to a framework comprising 117 machine learning combinations generated from ten classical machine learning algorithms: random forest (RSF), elastic network (Enet), stepwise Cox (StepCox), CoxBoost, partial least squares regression for Cox (plsRcox), supervised principal components (superpc), generalized boosted regression models (GBM), survival support vector machine (survivalsvm), Ridge, and least absolute shrinkage and selection operator (Lasso). The 1-year, 3-year, and 5-year AUC values of ROC were evaluated in both training and test datasets. The top five predictive models based on 1-year, 3-year, and 5-year AUC rankings were combined to identify the optimal model for predicting overall survival in patients with COAD, LUAD, PAAD, and SKCM, respectively. For all ROC analyses, an AUC greater than 0.85 was considered to indicate excellent performance.

### Quantifications, statistical analyses, and illustrations

ImageJ (https://imagej.net/ij/) was used to quantify the area of the entire disc, the GFP region, larval transparency, the staining signal density, and other parameters. For each group, quantification was performed using a sufficient number of randomly selected samples, which is denoted in the figure legends. Statistical analyses were performed using GraphPad Prism software (version 9.0, GraphPad Software, San Diego, CA). Results are presented as the mean ± SEM or mean ± SD. Comparisons between two groups with a normal distribution and homogeneity of variance were conducted using an unpaired Student’s *t* test; otherwise, the Mann–Whitney *U* test was used. For experiments involving three or more groups, data with a Gaussian distribution of residuals were analyzed using ordinary one-way ANOVA; otherwise, the nonparametric Kruskal–Wallis test was employed with recommended multiple comparison test. The categorical variables, like larval pupariation rate, were assessed by using the Fisher’s exact test or Chi-square test. The correlation analysis was performed by using Pearson or Spearman’s correlation analysis. Patient survival was analyzed using Kaplan–Meier curves and compared via the Log-rank test. Patients with different cancer types were stratified into two groups based on mutation status or gene expression, as indicated. A *P* value < 0.05 was considered statistically significant (*P < 0.05, **P < 0.01, ***P < 0.001, ****P < 0.0001); exact *P* values were provided either within the panels or in the figure legends. Schematics and illustrations were created using BioRender.com.

## Supplementary information

Peer Review File

Appendix

Dataset EV1

Dataset EV2

Dataset EV3

Dataset EV4

Dataset EV5

Dataset EV6

Source data Fig. 1

Source data Fig. 2

Source data Fig. 3

Source data Fig. 4

Source data Fig. 5

Source data Fig. 6

Source data Fig. 7

Expanded View Figures

## Data Availability

Our bulk RNA-seq and ATAC-seq data for this study have been deposited in the National Center for Biotechnology Information’s BioProject with accession PRJNA1209517 (https://www.ncbi.nlm.nih.gov/bioproject/PRJNA1209517/). Data for TCGA PanCancer Atlas Studies were collected from cBioPortal (Cerami et al, 2012; Gao et al, 2013; De Bruijn et al, 2023). Gene expression data and clinical data for TCGA and GTEx samples were obtained from UCSC-Xena (https://xenabrowser.net/datapages/) (Goldman et al, 2020). The scRNA-seq data are available from the Gene Expression Omnibus (GEO) database under accession numbers GSE139829 (Durante et al, 2020), GSE138665 (Pandiani et al, 2021), GSE132465 (Lee et al, 2020), GSE144735 (Lee et al, 2020), and GSE205013 (Werba et al, 2023; Benitz et al, 2024), or from Figshare with the identifier 10.6084/m9.figshare.25027181 (Mao et al, 2024). The custom code used in this study is available on GitHub. The source data of this paper are collected in the following database record: biostudies:S-SCDT-10_1038-S44320-026-00215-8.

## References

[CR1] Adachi Y, Kimura R, Hirade K, Yanase S, Nishioka Y, Kasuga N, Yamaguchi R, Ebi H (2023) Scribble mis-localization induces adaptive resistance to KRAS G12C inhibitors through feedback activation of MAPK signaling mediated by YAP-induced MRAS. Nat Cancer 4:829–843 37277529 10.1038/s43018-023-00575-2

[CR2] Aibar S, González-Blas CB, Moerman T, Huynh-Thu VA, Imrichova H, Hulselmans G, Rambow F, Marine J-C, Geurts P, Aerts J et al (2017) SCENIC: single-cell regulatory network inference and clustering. Nat Methods 14:1083–1086 28991892 10.1038/nmeth.4463 PMC5937676

[CR3] Altenhoff AM, Warwick Vesztrocy A, Bernard C, Train C-M, Nicheperovich A, Prieto Baños S, Julca I, Moi D, Nevers Y, Majidian S et al (2024) OMA orthology in 2024: improved prokaryote coverage, ancestral and extant GO enrichment, a revamped synteny viewer and more in the OMA ecosystem. Nucleic Acids Res 52:D513–D521 37962356 10.1093/nar/gkad1020 PMC10767875

[CR4] Amos B, Aurrecoechea C, Barba M, Barreto A, Basenko EY, Bażant W, Belnap R, Blevins AS, Böhme U, Brestelli J et al (2022) VEuPathDB: the eukaryotic pathogen, vector and host bioinformatics resource center. Nucleic Acids Res 50:D898–D911 34718728 10.1093/nar/gkab929 PMC8728164

[CR5] Anders S, Pyl PT, Huber W (2015) HTSeq—a Python framework to work with high-throughput sequencing data. Bioinformatics 31:166–169 25260700 10.1093/bioinformatics/btu638 PMC4287950

[CR6] Artavanis-Tsakonas S, Muskavitch MA, Yedvobnick B (1983) Molecular cloning of Notch, a locus affecting neurogenesis in *Drosophila melanogaster*. Proc Natl Acad Sci USA 80:1977–1981 6403942 10.1073/pnas.80.7.1977 PMC393735

[CR7] Ashburner M, Ball CA, Blake JA, Botstein D, Butler H, Cherry JM, Davis AP, Dolinski K, Dwight SS, Eppig JT et al (2000) Gene Ontology: tool for the unification of biology. Nat Genet 25:25–29 10802651 10.1038/75556 PMC3037419

[CR8] Barbie DA, Tamayo P, Boehm JS, Kim SY, Moody SE, Dunn IF, Schinzel AC, Sandy P, Meylan E, Scholl C et al (2009) Systematic RNA interference reveals that oncogenic KRAS-driven cancers require TBK1. Nature 462:108–112 19847166 10.1038/nature08460 PMC2783335

[CR9] Benitz S, Steep A, Nasser MM, Preall J, Mahajan UM, McQuithey H, Loveless I, Davis ET, Wen H-J, Long DW et al (2024) ROR2 regulates cellular plasticity in pancreatic neoplasia and adenocarcinoma. Cancer Discov 14:2162–2182 38975886 10.1158/2159-8290.CD-24-0137 PMC11528200

[CR10] Bermejo-Rodriguez C, Araos Henríquez J, Caligiuri G, Pinto Teles S, Park Y, Evans A, Barrera LN, Neesse A, Grützmann R, Aust D et al (2024) Scribble deficiency promotes pancreatic ductal adenocarcinoma development and metastasis. Cancer Res 84:2968–2984 39037766 10.1158/0008-5472.CAN-23-3419 PMC12662065

[CR11] Bilder D, Li M, Perrimon N (2000) Cooperative regulation of cell polarity and growth by Drosophila tumor suppressors. Science 289:113–116 10884224 10.1126/science.289.5476.113

[CR12] Bilder D, Perrimon N (2000) Localization of apical epithelial determinants by the basolateral PDZ protein scribble. Nature 403:676–680 10688207 10.1038/35001108

[CR13] Biswas A, De S (2021) Drivers of dynamic intratumor heterogeneity and phenotypic plasticity. Am J Physiol-Cell Physiol 320:C750–C760 33657326 10.1152/ajpcell.00575.2020 PMC8163571

[CR14] Brumby AM (2003) scribble mutants cooperate with oncogenic Ras or Notch to cause neoplastic overgrowth in Drosophila. EMBO J 22:5769–5779 14592975 10.1093/emboj/cdg548 PMC275405

[CR15] Brumby AM, Richardson HE (2005) Using *Drosophila melanogaster* to map human cancer pathways. Nat Rev Cancer 5:626–639 16034367 10.1038/nrc1671

[CR16] Cao Y, Fu L, Wu J, Peng Q, Nie Q, Zhang J, Xie X (2022) Integrated analysis of multimodal single-cell data with structural similarity. Nucleic Acids Res 50:e121 36130281 10.1093/nar/gkac781 PMC9757079

[CR17] Cerami E, Gao J, Dogrusoz U, Gross BE, Sumer SO, Aksoy BA, Jacobsen A, Byrne CJ, Heuer ML, Larsson E et al (2012) The cBio cancer genomics portal: an open platform for exploring multidimensional cancer genomics data. Cancer Discov 2:401–404 22588877 10.1158/2159-8290.CD-12-0095 PMC3956037

[CR18] Chiba S, Ikeda M, Katsunuma K, Ohashi K, Mizuno K (2009) MST2- and furry-mediated activation of NDR1 kinase is critical for precise alignment of mitotic chromosomes. Curr Biol 19:675–681 19327996 10.1016/j.cub.2009.02.054

[CR19] Chin C-H, Chen S-H, Wu H-H, Ho C-W, Ko M-T, Lin C-Y (2014) cytoHubba: identifying hub objects and sub-networks from complex interactome. BMC Syst Biol 8:S11 25521941 10.1186/1752-0509-8-S4-S11 PMC4290687

[CR20] Choucair K, Imtiaz H, Uddin MH, Nagasaka M, Al-Hallak MN, Philip PA, El-Rayes B, Pasche BC, Azmi AS (2025) Targeting KRAS mutations: orchestrating cancer evolution and therapeutic challenges. Sig Transduct Target Ther 10:385 10.1038/s41392-025-02473-8 PMC12660860 41309533

[CR21] Cosentino S, Sriswasdi S, Iwasaki W (2024) SonicParanoid2: fast, accurate, and comprehensive orthology inference with machine learning and language models. Genome Biol 25:195 39054525 10.1186/s13059-024-03298-4 PMC11270883

[CR22] Cui C, Wang J, Fagerberg E, Chen P-M, Connolly KA, Damo M, Cheung JF, Mao T, Askari AS, Chen S et al (2021) Neoantigen-driven B cell and CD4 T follicular helper cell collaboration promotes anti-tumor CD8 T cell responses. Cell 184:6101–6118.e13 34852236 10.1016/j.cell.2021.11.007 PMC8671355

[CR23] Danecek P, Bonfield JK, Liddle J, Marshall J, Ohan V, Pollard MO, Whitwham A, Keane T, McCarthy SA, Davies RM et al (2021) Twelve years of SAMtools and BCFtools. GigaScience 10:giab008 33590861 10.1093/gigascience/giab008 PMC7931819

[CR24] De Bruijn I, Kundra R, Mastrogiacomo B, Tran TN, Sikina L, Mazor T, Li X, Ochoa A, Zhao G, Lai B et al (2023) Analysis and visualization of longitudinal genomic and clinical data from the AACR Project GENIE Biopharma Collaborative in cBioPortal. Cancer Res 83:3861–3867 37668528 10.1158/0008-5472.CAN-23-0816 PMC10690089

[CR25] Devroe E, Erdjument-Bromage H, Tempst P, Silver PA (2004) Human mob proteins regulate the NDR1 and NDR2 serine-threonine kinases. J Biol Chem 279:24444–24451 15067004 10.1074/jbc.M401999200

[CR26] Durante MA, Rodriguez DA, Kurtenbach S, Kuznetsov JN, Sanchez MI, Decatur CL, Snyder H, Feun LG, Livingstone AS, Harbour JW (2020) Single-cell analysis reveals new evolutionary complexity in uveal melanoma. Nat Commun 11:496 31980621 10.1038/s41467-019-14256-1 PMC6981133

[CR27] Durinck S, Spellman PT, Birney E, Huber W (2009) Mapping identifiers for the integration of genomic datasets with the R/Bioconductor package biomaRt. Nat Protoc 4:1184–1191 19617889 10.1038/nprot.2009.97 PMC3159387

[CR28] Elsum IA, Yates LL, Pearson HB, Phesse TJ, Long F, O’Donoghue R, Ernst M, Cullinane C, Humbert PO (2014) Scrib heterozygosity predisposes to lung cancer and cooperates with KRas hyperactivation to accelerate lung cancer progression in vivo. Oncogene 33:5523–5533 24276238 10.1038/onc.2013.498

[CR29] Emms DM, Kelly S (2019) OrthoFinder: phylogenetic orthology inference for comparative genomics. Genome Biol 20:238 31727128 10.1186/s13059-019-1832-y PMC6857279

[CR30] Enomoto M, Siow C, Igaki T (2018) Drosophila as a cancer model. In: Yamaguchi M (ed) Drosophila models for human diseases. Springer Singapore, Singapore, pp 173–194 10.1007/978-981-13-0529-0_10 29951820

[CR31] Fang X, Adler PN (2010) Regulation of cell shape, wing hair initiation and the actin cytoskeleton by Trc/Fry and Wts/Mats complexes. Dev Biol 341:360–374 20211163 10.1016/j.ydbio.2010.02.029 PMC2862085

[CR32] Fang X, Lu Q, Emoto K, Adler PN (2010) The Drosophila Fry protein interacts with Trc and is highly mobile in vivo. BMC Dev Biol 10:40 20406475 10.1186/1471-213X-10-40 PMC2868802

[CR33] Figueroa-Clarevega A, Bilder D (2015) Malignant Drosophila tumors interrupt insulin signaling to induce cachexia-like wasting. Dev Cell 33:47–55 25850672 10.1016/j.devcel.2015.03.001 PMC4390765

[CR34] Flavahan WA, Gaskell E, Bernstein BE (2017) Epigenetic plasticity and the hallmarks of cancer. Science 357:eaal2380 28729483 10.1126/science.aal2380 PMC5940341

[CR35] Fuentes D, Molina M, Chorostecki U, Capella-Gutiérrez S, Marcet-Houben M, Gabaldón T (2022) PhylomeDB V5: an expanding repository for genome-wide catalogues of annotated gene phylogenies. Nucleic Acids Res 50:D1062–D1068 34718760 10.1093/nar/gkab966 PMC8728271

[CR36] Gao J, Aksoy BA, Dogrusoz U, Dresdner G, Gross B, Sumer SO, Sun Y, Jacobsen A, Sinha R, Larsson E et al (2013) Integrative analysis of complex cancer genomics and clinical profiles using the cBioPortal. Sci Signal 6:pl1 10.1126/scisignal.2004088 PMC4160307 23550210

[CR37] Goldman MJ, Craft B, Hastie M, Repečka K, McDade F, Kamath A, Banerjee A, Luo Y, Rogers D, Brooks AN et al (2020) Visualizing and interpreting cancer genomics data via the Xena platform. Nat Biotechnol 38:675–678 32444850 10.1038/s41587-020-0546-8 PMC7386072

[CR38] Gourmet L, Sottoriva A, Walker-Samuel S, Secrier M, Zapata L (2024) Immune evasion impacts the landscape of driver genes during cancer evolution. Genome Biol 25:168 38926878 10.1186/s13059-024-03302-x PMC11210199

[CR39] Gramates LS, Agapite J, Attrill H, Calvi BR, Crosby MA, Dos Santos G, Goodman JL, Goutte-Gattat D, Jenkins VK, Kaufman T et al (2022) FlyBase: a guided tour of highlighted features. Genetics 220:iyac035 35266522 10.1093/genetics/iyac035 PMC8982030

[CR40] Greaves M (2015) Evolutionary determinants of cancer. Cancer Discov 5:806–820 26193902 10.1158/2159-8290.CD-15-0439 PMC4539576

[CR41] Greaves M, Maley CC (2012) Clonal evolution in cancer. Nature 481:306–313 22258609 10.1038/nature10762 PMC3367003

[CR42] Hänzelmann S, Castelo R, Guinney J (2013) GSVA: gene set variation analysis for microarray and RNA-Seq data. BMC Bioinforma 14:7 10.1186/1471-2105-14-7 PMC3618321 23323831

[CR43] Hariharan IK, Bilder D (2006) Regulation of imaginal disc growth by tumor-suppressor genes in Drosophila. Annu Rev Genet 40:335–361 16872256 10.1146/annurev.genet.39.073003.100738

[CR44] Harvey KF, Pfleger CM, Hariharan IK (2003) The Drosophila Mst ortholog, hippo, restricts growth and cell proliferation and promotes apoptosis. Cell 114:457–467 12941274 10.1016/s0092-8674(03)00557-9

[CR45] He Y, Emoto K, Fang X, Ren N, Tian X, Jan Y-N, Adler PN (2005) *Drosophila* Mob family proteins interact with the related tricornered (Trc) and warts (Wts) kinases. MBoC 16:4139–4152 15975907 10.1091/mbc.E05-01-0018 PMC1196325

[CR46] Heinz S, Benner C, Spann N, Bertolino E, Lin YC, Laslo P, Cheng JX, Murre C, Singh H, Glass CK (2010) Simple combinations of lineage-determining transcription factors prime cis-regulatory elements required for macrophage and B cell identities. Mol Cell 38:576–589 20513432 10.1016/j.molcel.2010.05.004 PMC2898526

[CR47] Hernández-Plaza A, Szklarczyk D, Botas J, Cantalapiedra CP, Giner-Lamia J, Mende DR, Kirsch R, Rattei T, Letunic I, Jensen LJ et al (2023) eggNOG 6.0: enabling comparative genomics across 12 535 organisms. Nucleic Acids Res 51:D389–D394 36399505 10.1093/nar/gkac1022 PMC9825578

[CR48] Herz H-M, Woodfield SE, Chen Z, Bolduc C, Bergmann A (2009) Common and distinct genetic properties of ESCRT-II components in Drosophila. PLoS ONE 4:e4165 19132102 10.1371/journal.pone.0004165 PMC2613530

[CR49] Hu Y, Comjean A, Attrill H, Antonazzo G, Thurmond J, Chen W, Li F, Chao T, Mohr SE, Brown NH et al (2023) PANGEA: a new gene set enrichment tool for Drosophila and common research organisms. Nucleic Acids Res 51:W419–W426 37125646 10.1093/nar/gkad331 PMC10320058

[CR50] Irie K, Nagai T, Mizuno K (2020) Furry protein suppresses nuclear localization of yes-associated protein (YAP) by activating NDR kinase and binding to YAP. J Biol Chem 295:3017–3028 31996378 10.1074/jbc.RA119.010783 PMC7062186

[CR51] Jia J, Zhang W, Wang B, Trinko R, Jiang J (2003) The Drosophila Ste20 family kinase dMST functions as a tumor suppressor by restricting cell proliferation and promoting apoptosis. Genes Dev 17:2514–2519 14561774 10.1101/gad.1134003 PMC218145

[CR52] Johnson C, Burkhart DL, Haigis KM (2022) Classification of *KRAS* -activating mutations and the implications for therapeutic intervention. Cancer Discov 12:913–923 35373279 10.1158/2159-8290.CD-22-0035 PMC8988514

[CR53] Justice RW, Zilian O, Woods DF, Noll M, Bryant PJ (1995) The Drosophila tumor suppressor gene warts encodes a homolog of human myotonic dystrophy kinase and is required for the control of cell shape and proliferation. Genes Dev 9:534–546 7698644 10.1101/gad.9.5.534

[CR54] Kaduk M, Riegler C, Lemp O, Sonnhammer ELL (2017) HieranoiDB: a database of orthologs inferred by Hieranoid. Nucleic Acids Res 45:D687–D690 27742821 10.1093/nar/gkw923 PMC5210627

[CR55] Kanehisa M, Furumichi M, Tanabe M, Sato Y, Morishima K (2017) KEGG: new perspectives on genomes, pathways, diseases and drugs. Nucleic Acids Res 45:D353–D361 27899662 10.1093/nar/gkw1092 PMC5210567

[CR56] Kanehisa M, Goto S (2000) KEGG: Kyoto encyclopedia of genes and genomes. Nucleic Acids Res 28:27–30 10592173 10.1093/nar/28.1.27 PMC102409

[CR57] Kidd S, Lockett TJ, Young MW (1983) The Notch locus of *Drosophila melanogaster*. Cell 34:421–433 6193889 10.1016/0092-8674(83)90376-8

[CR58] Killcoyne S, Carter GW, Smith J, Boyle J (2009) Cytoscape: a community-based framework for network modeling. In: Nikolsky Y, Bryant J (eds) Protein networks and pathway analysis. Humana Press, Totowa, NJ, pp 219–239 10.1007/978-1-60761-175-2_12 19597788

[CR59] Kim D, Paggi JM, Park C, Bennett C, Salzberg SL (2019) Graph-based genome alignment and genotyping with HISAT2 and HISAT-genotype. Nat Biotechnol 37:907–915 31375807 10.1038/s41587-019-0201-4 PMC7605509

[CR60] Kong D, Lu J-Y, Li X, Zhao S, Xu W, Fang J, Wang X, Ma X (2021) Misshapen disruption cooperates with RasV12 to drive tumorigenesis. Cells 10:894 33919765 10.3390/cells10040894 PMC8070713

[CR61] Kumar L, Futschik ME (2007) Mfuzz: a software package for soft clustering of microarray data. Bioinformation 2:5–7 18084642 10.6026/97320630002005 PMC2139991

[CR62] Kuznetsov D, Tegenfeldt F, Manni M, Seppey M, Berkeley M, Kriventseva EV, Zdobnov EM (2023) OrthoDB v11: annotation of orthologs in the widest sampling of organismal diversity. Nucleic Acids Res 51:D445–D451 36350662 10.1093/nar/gkac998 PMC9825584

[CR63] Kwon Y, Song W, Droujinine IA, Hu Y, Asara JM, Perrimon N (2015) Systemic Organ wasting induced by localized expression of the secreted insulin/IGF antagonist ImpL2. Dev Cell 33:36–46 25850671 10.1016/j.devcel.2015.02.012 PMC4437243

[CR64] Langfelder P, Horvath S (2008) WGCNA: an R package for weighted correlation network analysis. BMC Bioinforma 9:559 10.1186/1471-2105-9-559 PMC2631488 19114008

[CR65] Langmead B, Salzberg SL (2012) Fast gapped-read alignment with Bowtie 2. Nat Methods 9:357–359 22388286 10.1038/nmeth.1923 PMC3322381

[CR66] Lee H-O, Hong Y, Etlioglu HE, Cho YB, Pomella V, Van Den Bosch B, Vanhecke J, Verbandt S, Hong H, Min J-W et al (2020) Lineage-dependent gene expression programs influence the immune landscape of colorectal cancer. Nat Genet 52:594–603 32451460 10.1038/s41588-020-0636-z

[CR67] Lee JK, Sivakumar S, Schrock AB, Madison R, Fabrizio D, Gjoerup O, Ross JS, Frampton GM, Napalkov P, Montesion M et al (2022) Comprehensive pan-cancer genomic landscape of KRAS altered cancers and real-world outcomes in solid tumors. NPJ Precis Onc 6:1–14 10.1038/s41698-022-00334-z PMC9734185 36494601

[CR68] Lee T, Luo L (1999) Mosaic analysis with a repressible cell marker for studies of gene function in neuronal morphogenesis. Neuron 22:451–461 10197526 10.1016/s0896-6273(00)80701-1

[CR69] Leek JT, Johnson WE, Parker HS, Jaffe AE, Storey JD (2012) The sva package for removing batch effects and other unwanted variation in high-throughput experiments. Bioinformatics 28:882–883 22257669 10.1093/bioinformatics/bts034 PMC3307112

[CR70] Liang F, Wang R, Du Q, Zhu S (2021) An epithelial–mesenchymal transition hallmark gene-based risk score system in head and neck squamous-cell carcinoma. Int J Gen Med 14:4219–4227 10.2147/IJGM.S327632 PMC8354775 34393501

[CR71] Liberzon A, Birger C, Thorvaldsdóttir H, Ghandi M, Mesirov JP, Tamayo P (2015) The molecular signatures database hallmark gene set collection. cels 1:417–425 10.1016/j.cels.2015.12.004 PMC4707969 26771021

[CR72] Liu H, Zhang W, Zhang Y, Adegboro AA, Fasoranti DO, Dai L, Pan Z, Liu H, Xiong Y, Li W et al (2024) Mime: a flexible machine-learning framework to construct and visualize models for clinical characteristics prediction and feature selection. Comput Struct Biotechnol J 23:2798–2810 39055398 10.1016/j.csbj.2024.06.035 PMC11269309

[CR73] Liu Y, Saavedra P, Perrimon N (2022) Cancer cachexia: lessons from Drosophila. Dis Models Mechan 15:dmm049298 10.1242/dmm.049298 PMC8961677 35319749

[CR74] Lodge W, Zavortink M, Golenkina S, Froldi F, Dark C, Cheung S, Parker BL, Blazev R, Bakopoulos D, Christie EL et al (2021) Tumor-derived MMPs regulate cachexia in a Drosophila cancer model. Dev Cell 56:2664–2680.e6 34473940 10.1016/j.devcel.2021.08.008

[CR75] Lu H, Bilder D (2005) Endocytic control of epithelial polarity and proliferation in Drosophila. Nat Cell Biol 7:1232–1239 16258546 10.1038/ncb1324

[CR76] Ma X, Lu J-Y, Dong Y, Li D, Malagon JN, Xu T (2017) PP6 Disruption synergizes with oncogenic Ras to promote JNK-dependent tumor growth and invasion. Cell Rep 19:2657–2664 28658615 10.1016/j.celrep.2017.05.092 PMC5580353

[CR77] Ma X, Lu J-Y, Moraru A, Teleman AA, Fang J, Qiu Y, Liu P, Xu T (2020) A novel regulator of ER Ca2+ drives Hippo-mediated tumorigenesis. Oncogene 39:1378–1387 31649333 10.1038/s41388-019-1076-z

[CR78] Mao S, Wang Y, Chao N, Zeng L, Zhang L (2024) Integrated analysis of single-cell RNA-seq and bulk RNA-seq reveals immune suppression subtypes and establishes a novel signature for determining the prognosis in lung adenocarcinoma. Cell Oncol 47:1697–1713 10.1007/s13402-024-00948-4 PMC12974075 38616208

[CR79] Martin FJ, Amode MR, Aneja A, Austine-Orimoloye O, Azov AG, Barnes I, Becker A, Bennett R, Berry A, Bhai J et al (2023) Ensembl 2023. Nucleic Acids Res 51:D933–D941 36318249 10.1093/nar/gkac958 PMC9825606

[CR80] Martin M (2011) Cutadapt removes adapter sequences from high-throughput sequencing reads. EMBnet J 17:10–12

[CR81] Menéndez J, Pérez-Garijo A, Calleja M, Morata G (2010) A tumor-suppressing mechanism in Drosophila involving cell competition and the Hippo pathway. Proc Natl Acad Sci USA 107:14651–14656 20679206 10.1073/pnas.1009376107 PMC2930466

[CR82] Mering CV (2003) STRING: a database of predicted functional associations between proteins. Nucleic Acids Res 31:258–261 12519996 10.1093/nar/gkg034 PMC165481

[CR83] Moberg KH, Schelble S, Burdick SK, Hariharan IK (2005) Mutations in erupted, the Drosophila ortholog of mammalian tumor susceptibility gene 101, elicit non-cell-autonomous overgrowth. Dev Cell 9:699–710 16256744 10.1016/j.devcel.2005.09.018

[CR84] Mukhopadhyay S, Vander Heiden MG, McCormick F (2021) The metabolic landscape of RAS-driven cancers from biology to therapy. Nat Cancer 2:271–283 33870211 10.1038/s43018-021-00184-x PMC8045781

[CR85] Nevers Y, Kress A, Defosset A, Ripp R, Linard B, Thompson JD, Poch O, Lecompte O (2019) OrthoInspector 3.0: open portal for comparative genomics. Nucleic Acids Res 47:D411–D418 30380106 10.1093/nar/gky1068 PMC6323921

[CR86] Newman AM, Liu CL, Green MR, Gentles AJ, Feng W, Xu Y, Hoang CD, Diehn M, Alizadeh AA (2015) Robust enumeration of cell subsets from tissue expression profiles. Nat Methods 12:453–457 25822800 10.1038/nmeth.3337 PMC4739640

[CR87] Pandiani C, Strub T, Nottet N, Cheli Y, Gambi G, Bille K, Husser C, Dalmasso M, Béranger G, Lassalle S et al (2021) Single-cell RNA sequencing reveals intratumoral heterogeneity in primary uveal melanomas and identifies HES6 as a driver of the metastatic disease. Cell Death Differ 28:1990–2000 33462406 10.1038/s41418-020-00730-7 PMC8185008

[CR88] Pantalacci S, Tapon N, Léopold P (2003) The Salvador partner Hippo promotes apoptosis and cell-cycle exit in Drosophila. Nat Cell Biol 5:921–927 14502295 10.1038/ncb1051

[CR89] Peng C-Y, Manning L, Albertson R, Doe CQ (2000) The tumour-suppressor genes lgl and dlg regulate basal protein targeting in Drosophila neuroblasts. Nature 408:596–600 11117748 10.1038/35046094

[CR90] Perrimon N (1988) The maternal effect of *lethal(1)discs-large-1:* a recessive oncogene of *Drosophila melanogaster*. Dev Biol 127:392–407 3132409 10.1016/0012-1606(88)90326-0

[CR91] Persson E, Kaduk M, Forslund SK, Sonnhammer ELL (2019) Domainoid: domain-oriented orthology inference. BMC Bioinforma 20:523 10.1186/s12859-019-3137-2 PMC6816169 31660857

[CR92] Persson E, Sonnhammer ELL (2023) InParanoiDB 9: ortholog groups for protein domains and full-length proteins. J Mol Biol 435:168001 36764355 10.1016/j.jmb.2023.168001

[CR93] Pon JR, Marra MA (2015) Driver and passenger mutations in cancer. Annu Rev Pathol Mech Dis 10:25–50 10.1146/annurev-pathol-012414-040312 25340638

[CR94] Prior IA, Hood FE, Hartley JL (2020) The frequency of Ras mutations in cancer. Cancer Res 80:2969–2974 32209560 10.1158/0008-5472.CAN-19-3682 PMC7367715

[CR95] Putri GH, Anders S, Pyl PT, Pimanda JE, Zanini F (2022) Analysing high-throughput sequencing data in Python with HTSeq 2.0. Bioinformatics 38:2943–2945 35561197 10.1093/bioinformatics/btac166 PMC9113351

[CR96] Pylayeva-Gupta Y, Grabocka E, Bar-Sagi D (2011) RAS oncogenes: weaving a tumorigenic web. Nat Rev Cancer 11:761–774 21993244 10.1038/nrc3106 PMC3632399

[CR97] Quinlan AR, Hall IM (2010) BEDTools: a flexible suite of utilities for comparing genomic features. Bioinformatics 26:841–842 20110278 10.1093/bioinformatics/btq033 PMC2832824

[CR98] Rajapakse VN, Luna A, Yamade M, Loman L, Varma S, Sunshine M, Iorio F, Sousa FG, Elloumi F, Aladjem MI et al (2018) CellMinerCDB for integrative cross-database genomics and pharmacogenomics analyses of cancer cell lines. iScience 10:247–264 30553813 10.1016/j.isci.2018.11.029 PMC6302245

[CR99] Ramírez F, Ryan DP, Grüning B, Bhardwaj V, Kilpert F, Richter AS, Heyne S, Dündar F, Manke T (2016) deepTools2: a next generation web server for deep-sequencing data analysis. Nucleic Acids Res 44:W160–W165 27079975 10.1093/nar/gkw257 PMC4987876

[CR100] Riesgo-Escovar JR, Jenni M, Fritz A, Hafen E (1996) The Drosophila Jun-N-terminal kinase is required for cell morphogenesis but not for DJun-dependent cell fate specification in the eye. Genes Dev 10:2759–2768 8946916 10.1101/gad.10.21.2759

[CR101] Robin X, Turck N, Hainard A, Tiberti N, Lisacek F, Sanchez J-C, Müller M (2011) pROC: an open-source package for R and S+ to analyze and compare ROC curves. BMC Bioinforma 12:77 10.1186/1471-2105-12-77 PMC3068975 21414208

[CR102] Robinson BS, Moberg KH (2011) Drosophila endocytic neoplastic tumor suppressor genes regulate Sav/Wts/Hpo signaling and the c-Jun N-terminal kinase pathway. Cell Cycle 10:4110–4118 22101275 10.4161/cc.10.23.18243 PMC3272291

[CR103] Robinson JT, Thorvaldsdóttir H, Winckler W, Guttman M, Lander ES, Getz G, Mesirov JP (2011) Integrative genomics viewer. Nat Biotechnol 29:24–26 21221095 10.1038/nbt.1754 PMC3346182

[CR104] Robinson MD, McCarthy DJ, Smyth GK (2010) edgeR: a Bioconductor package for differential expression analysis of digital gene expression data. Bioinformatics 26:139–140 19910308 10.1093/bioinformatics/btp616 PMC2796818

[CR105] Scheffler M, Ihle MA, Hein R, Merkelbach-Bruse S, Scheel AH, Siemanowski J, Brägelmann J, Kron A, Abedpour N, Ueckeroth F et al (2019) K-ras mutation subtypes in NSCLC and associated co-occuring mutations in other oncogenic pathways. J Thorac Oncol 14:606–616 30605727 10.1016/j.jtho.2018.12.013

[CR106] Sonoshita M, Cagan RL (2017) Modeling human cancers in Drosophila. Curr Top Dev Biol 121:287–309 10.1016/bs.ctdb.2016.07.008 28057303

[CR107] Stratton MR, Campbell PJ, Futreal PA (2009) The cancer genome. Nature 458:719–724 19360079 10.1038/nature07943 PMC2821689

[CR108] Subramanian A, Tamayo P, Mootha VK, Mukherjee S, Ebert BL, Gillette MA, Paulovich A, Pomeroy SL, Golub TR, Lander ES et al (2005) Gene set enrichment analysis: a knowledge-based approach for interpreting genome-wide expression profiles. Proc Natl Acad Sci 102:15545–15550 16199517 10.1073/pnas.0506580102 PMC1239896

[CR109] Szklarczyk D, Nastou K, Koutrouli M, Kirsch R, Mehryary F, Hachilif R, Hu D, Peluso ME, Huang Q, Fang T et al (2025) The STRING database in 2025: protein networks with directionality of regulation. Nucleic Acids Res 53:D730–D737 39558183 10.1093/nar/gkae1113 PMC11701646

[CR110] Tang Z, Kang B, Li C, Chen T, Zhang Z (2019) GEPIA2: an enhanced web server for large-scale expression profiling and interactive analysis. Nucleic Acids Res 47:W556–W560 31114875 10.1093/nar/gkz430 PMC6602440

[CR111] Tekle GE, Bernasocchi T, Unni AM, Bertoni F, Rossi D, Rubin MA, Theurillat J-P (2021) Co-occurrence and mutual exclusivity: what cross-cancer mutation patterns can tell us. Trends Cancer 7:823–836 34031014 10.1016/j.trecan.2021.04.009

[CR112] The Cancer Genome Atlas Research Network, Weinstein JN, Collisson EA, Mills GB, Shaw KRM, Ozenberger BA, Ellrott K, Shmulevich I, Sander C, Stuart JM (2013) The Cancer Genome Atlas Pan-Cancer analysis project. Nat Genet 45:1113–1120 24071849 10.1038/ng.2764 PMC3919969

[CR113] The GTEx Consortium, Lonsdale J, Thomas J, Salvatore M, Phillips R, Lo E, Shad S, Hasz R, Walters G, Garcia F et al (2013) The Genotype-Tissue Expression (GTEx) project. Nat Genet 45:580–585 23715323 10.1038/ng.2653 PMC4010069

[CR114] Thomas PD, Ebert D, Muruganujan A, Mushayahama T, Albou L-P, Mi H (2022) PANTHER: making genome-scale phylogenetics accessible to all. Protein Sci 31:8–22 34717010 10.1002/pro.4218 PMC8740835

[CR115] Thurmond J, Goodman JL, Strelets VB, Attrill H, Gramates LS, Marygold SJ, Matthews BB, Millburn G, Antonazzo G, Trovisco V et al (2019) FlyBase 2.0: the next generation. Nucleic Acids Res 47:D759–D765 30364959 10.1093/nar/gky1003 PMC6323960

[CR116] Udan RS, Kango-Singh M, Nolo R, Tao C, Halder G (2003) Hippo promotes proliferation arrest and apoptosis in the Salvador/Warts pathway. Nat Cell Biol 5:914–920 14502294 10.1038/ncb1050

[CR117] Vaccari T, Lu H, Kanwar R, Fortini ME, Bilder D (2008) Endosomal entry regulates Notch receptor activation in *Drosophila melanogaster*. J Cell Biol 180:755–762 18299346 10.1083/jcb.200708127 PMC2265571

[CR118] Van der Maaten L, Hinton G (2008) Visualizing data using t-SNE. J Machine Learn Res 9:11

[CR119] Vendramin R, Litchfield K, Swanton C (2021) Cancer evolution: Darwin and beyond. EMBO J 40:e108389 34459009 10.15252/embj.2021108389 PMC8441388

[CR120] Vilella AJ, Severin J, Ureta-Vidal A, Heng L, Durbin R, Birney E (2009) EnsemblCompara GeneTrees: complete, duplication-aware phylogenetic trees in vertebrates. Genome Res 19:327–335 19029536 10.1101/gr.073585.107 PMC2652215

[CR121] Vogelstein B, Papadopoulos N, Velculescu VE, Zhou S, Diaz LA, Kinzler KW (2013) Cancer genome landscapes. Science 339:1546–1558 23539594 10.1126/science.1235122 PMC3749880

[CR122] Wang X, Guo Y, Lin P, Yu M, Song S, Xu W, Kong D, Wang Y, Zhang Y, Lu F et al (2024) Nuclear receptor E75/NR1D2 promotes tumor malignant transformation by integrating Hippo and Notch pathways. EMBO J 43:6336–6363 39516282 10.1038/s44318-024-00290-3 PMC11649922

[CR123] Wang X, Guo Y, Wang C, Mu J, Ma X (2026) E75-induced Toll/NF-κB signaling cooperates with Notch and Hippo pathways to promote tumor malignancy. J Genet Genomics 53:547–550 41177452 10.1016/j.jgg.2025.10.006

[CR124] Werba G, Weissinger D, Kawaler EA, Zhao E, Kalfakakou D, Dhara S, Wang L, Lim HB, Oh G, Jing X et al (2023) Single-cell RNA sequencing reveals the effects of chemotherapy on human pancreatic adenocarcinoma and its tumor microenvironment. Nat Commun 14:797 36781852 10.1038/s41467-023-36296-4 PMC9925748

[CR125] Wheeler DL, Barrett T, Benson DA, Bryant SH, Canese K, Chetvernin V, Church DM, DiCuccio M, Edgar R, Federhen S et al (2006) Database resources of the National Center for Biotechnology Information. Nucleic Acids Res 34:D173–D180 16381840 10.1093/nar/gkj158 PMC1347520

[CR126] Wu M, Pastor-Pareja JC, Xu T (2010) Interaction between RasV12 and scribbled clones induces tumour growth and invasion. Nature 463:545–548 20072127 10.1038/nature08702 PMC2835536

[CR127] Wu S, Huang J, Dong J, Pan D (2003) Hippo encodes a Ste-20 family protein kinase that restricts cell proliferation and promotes apoptosis in conjunction with Salvador and Warts. Cell 114:445–456 12941273 10.1016/s0092-8674(03)00549-x

[CR128] Wu Y, Siadaty MS, Berens ME, Hampton GM, Theodorescu D (2008) Overlapping gene expression profiles of cell migration and tumor invasion in human bladder cancer identify metallothionein 1E and nicotinamide N-methyltransferase as novel regulators of cell migration. Oncogene 27:6679–6689 18724390 10.1038/onc.2008.264 PMC5373842

[CR129] Xu T, Wang W, Zhang S, Stewart RA, Yu W (1995) Identifying tumor suppressors in genetic mosaics: the Drosophila lats gene encodes a putative protein kinase. Development 121:1053–1063 7743921 10.1242/dev.121.4.1053

[CR130] Yan F, Powell DR, Curtis DJ, Wong NC (2020) From reads to insight: a hitchhiker’s guide to ATAC-seq data analysis. Genome Biol 21:22 32014034 10.1186/s13059-020-1929-3 PMC6996192

[CR131] Yang S, Guo Y, Ma X (2023) Loss of the emei tumor suppressor promotes tumorigenesis via the JNK and Hippo pathway. Genes Dis 10:329–331 37223519 10.1016/j.gendis.2022.02.020 PMC10201582

[CR132] Yoshihara K, Shahmoradgoli M, Martínez E, Vegesna R, Kim H, Torres-Garcia W, Treviño V, Shen H, Laird PW, Levine DA et al (2013) Inferring tumour purity and stromal and immune cell admixture from expression data. Nat Commun 4:2612 24113773 10.1038/ncomms3612 PMC3826632

[CR133] Yu G, Wang L-G, Han Y, He Q-Y (2012) clusterProfiler: an R package for comparing biological themes among gene clusters. OMICS: A J Integr Biol 16:284–287 10.1089/omi.2011.0118 PMC3339379 22455463

[CR134] Yu G, Wang L-G, He Q-Y (2015) ChIPseeker: an R/Bioconductor package for ChIP peak annotation, comparison and visualization. Bioinformatics 31:2382–2383 25765347 10.1093/bioinformatics/btv145

[CR135] Yue J, Sun H, Liu S, Yu F, Wang S, Wang F, Shen R, Zhu F, Zhang L, Shao C (2018) Downregulation of NDR 1 contributes to metastasis of prostate cancer cells via activating epithelial-mesenchymal transition. Cancer Med 7:3200–3212 29733518 10.1002/cam4.1532 PMC6051198

[CR136] Zahir N, Sun R, Gallahan D, Gatenby RA, Curtis C (2020) Characterizing the ecological and evolutionary dynamics of cancer. Nat Genet 52:759–767 32719518 10.1038/s41588-020-0668-4

[CR137] Zhang S, Xiao X, Yi Y, Wang X, Zhu L, Shen Y, Lin D, Wu C (2024) Tumor initiation and early tumorigenesis: molecular mechanisms and interventional targets. Sig Transduct Target Ther 9:149 10.1038/s41392-024-01848-7 PMC11189549 38890350

[CR138] Zhang Y, Liu T, Meyer CA, Eeckhoute J, Johnson DS, Bernstein BE, Nusbaum C, Myers RM, Brown M, Li W et al (2008) Model-based analysis of ChIP-Seq (MACS). Genome Biol 9:R137 18798982 10.1186/gb-2008-9-9-r137 PMC2592715

[CR139] Zhao S, Guo Y, Kuang X, Li X, Wu C, Lin P, Xie Q, Zhai Z, Kong D, Ma X (2025) Hemocytes facilitate interclonal cooperation-induced tumor malignancy by hijacking the innate immune system in Drosophila. EMBO J 44:5394–5428 40846901 10.1038/s44318-025-00547-5 PMC12489090

