# Supplementary material for: Cross-species insights from ART-D to uncover evolutionarily conserved oncogenic mechanisms
Source: Mol Syst Biol. 2026 May 14;22(8):1214–51. doi: 10.1038/s44320-026-00215-8 (PMC13433787; doi:10.1038/s44320-026-00215-8)
Supplement: Supplementary file 1 [file 44320_2026_215_MOESM16_ESM.pdf]

Expanded View Figures

Figure EV1. Ten *Drosophila Ras*-driven malignant tumor models.

(**A**) The schematic diagram depicting the ethyl methanesulfonate (EMS) genetic screening of the *Drosophila* eye-antennal disc to identify novel tumor suppressors (*Vps36*, *Syx7*, *Rabex-5*, *TSG101*, *fry*, *fmt*, *emei*, and *msn*) on the *Drosophila* 3L chromosome, along with two well-known tumor suppressors (*scrib* and *l(2)gl*) that can synergize with oncogenic *Ras* (Ras^{V12}) to drive malignant tumorigenesis. The mutations of ten tumor suppressors used in this study were denoted as indicated. The gene structure was drawn by using GSDS 2.0 (https://gsds.gao-lab.org/) with sequence downloaded from FlyBase (https://flybase.org/). (**B**) The malignant tumors driven by ten tumor suppressors in the context of *Ras* activation at 8 days AEL. Scale bar: 100 μm. (**C**) Quantification of the overall disc size of each tumor model at 8 days AEL. The *wildtype* and malignant tumor groups were compared to the *Ras* group by using Mann–Whitney *U* test, $n > 30$. Results were presented as means ± SEM. Significance of different genotypes were denoted as follows: ns >0.05, $^{*}P < 0.05$, $^{**}P < 0.01$, $^{***}P < 0.001$, $^{****}P < 0.0001$. For exact *P* values, in (**C**), from left to right, $^{*}P = 0.0323$, $^{*}P = 0.0233$, $^{****}P < 0.0001$, $^{****}P < 0.0001$, $^{****}P < 0.0001$, $^{***}P = 0.0004$, $^{****}P < 0.0001$, $^{*}P = 0.0135$, $^{*}P = 0.0262$, $^{*}P = 0.0193$, $^{****}P < 0.0001$. (**D**) Quantification of the GFP region of each tumor model at 8 days AEL. The *wildtype* and malignant tumor groups were compared to the *Ras* group by using Mann–Whitney *U* test, $n > 30$. Results were presented as means ± SEM. Significance of different genotypes were denoted as follows: ns > 0.05, $^{*}P < 0.05$, $^{**}P < 0.01$, $^{***}P < 0.001$, $^{****}P < 0.0001$. For exact *P* values, in (**D**), from left to right, $^{***}P = 0.0002$, $^{**}P = 0.0083$, $^{****}P < 0.0001$, $^{****}P < 0.0001$, $^{***}P = 0.0010$, $^{**}P = 0.0036$, $^{***}P = 0.0004$, $^{*}P = 0.0147$, $^{*}P = 0.0348$, $^{***}P = 0.0003$, $^{****}P < 0.0001$. (**E**) Quantification of larval pupariation rate of ten malignant tumor models. The *Ras* group and malignant tumor groups were compared to *wildtype* group by using Chi-square test when the sample size was at least 40 ($n \geq 40$) and the expected frequency in each cell was no less than 5 ($T \geq 5$); otherwise, Fisher's exact test was used. Significance of different genotypes were denoted as follows: ns >0.05, $^{*}P < 0.05$, $^{**}P < 0.01$, $^{***}P < 0.001$, $^{****}P < 0.0001$. For each group, $n > 30$. For exact *P* values, in (**E**), from left to right, ns $P > 0.9999$, $^{****}P < 0.0001$, $^{****}P < 0.0001$, $^{****}P < 0.0001$, $^{****}P < 0.0001$, $^{****}P < 0.0001$, $^{***}P = 0.0001$, $^{***}P = 0.0001$, $^{****}P < 0.0001$, $^{**}P = 0.0012$, $^{****}P < 0.0001$. (**F**) The representative pictures of *Drosophila Ras*-driven tumors exhibiting aggressive intestinal metastasis at 8 days AEL. Scale bar: 200 μm. (**G**) The quantification of VNC invasion events of ten malignant tumor models at designated time points compared with *wildtype* discs and *Ras* benign tumors. The VNC invasion stage of collected samples were classified into four stages with the criterion shown in Fig. 1A.

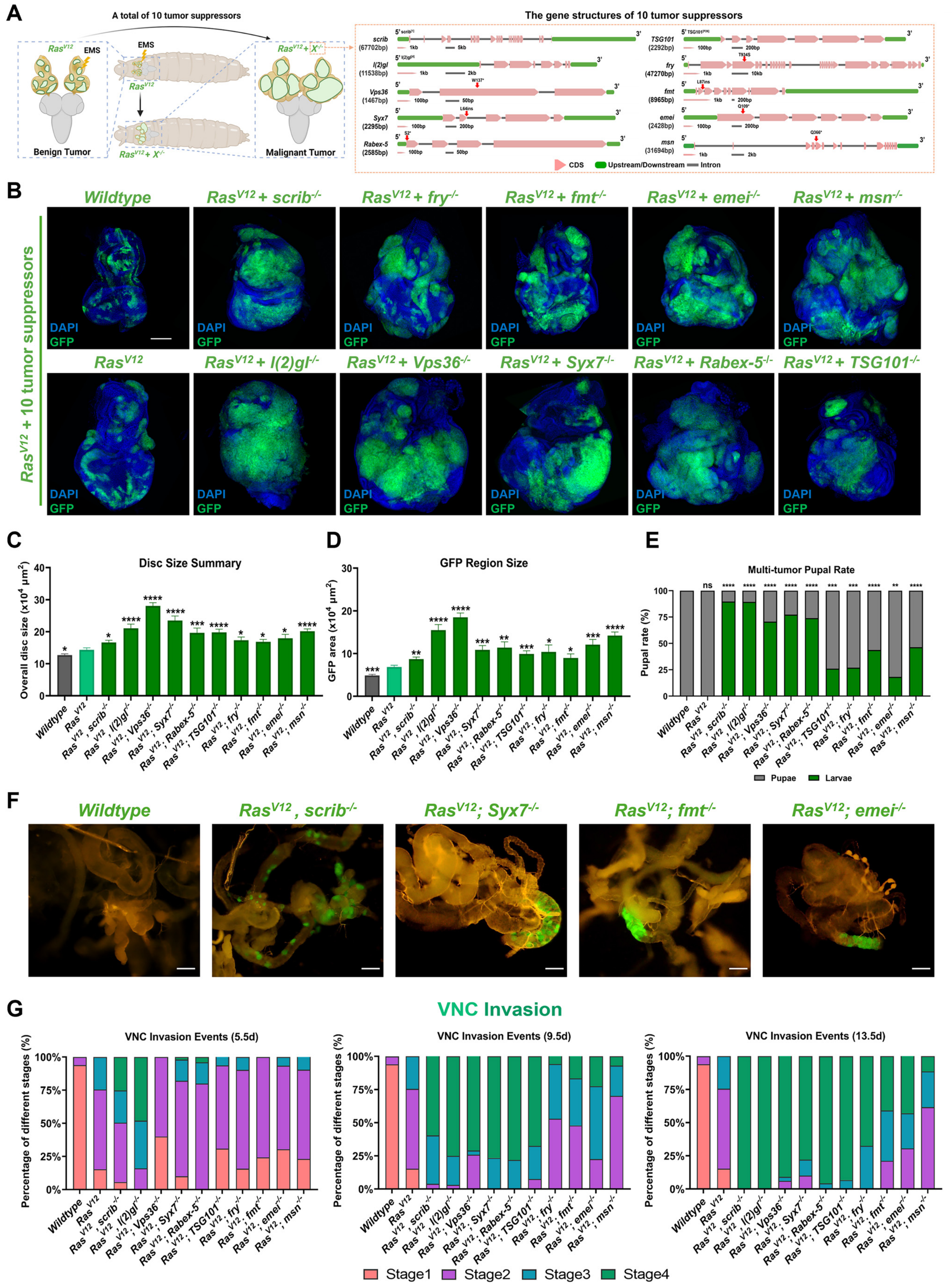
A
A total of 10 tumor suppressors
The gene structures of 10 tumor suppressors
Benign Tumor
Malignant Tumor
EMS
CDS
Upstream/Downstream
Intron
B
Wildtype
Rasᵛ¹² + 10 tumor suppressors
DAPI
GFP
C
Disc Size Summary
Overall disc size (x10⁴ μm²)
D
GFP Region Size
GFP area (x10⁴ μm²)
E
Multi-tumor Pupal Rate
Pupal rate (%)
Pupae
Larvae
F
G
VNC Invasion
VNC Invasion Events (5.5d)
VNC Invasion Events (9.5d)
VNC Invasion Events (13.5d)
Percentage of different stages (%)
Stage1
Stage2
Stage3
Stage4

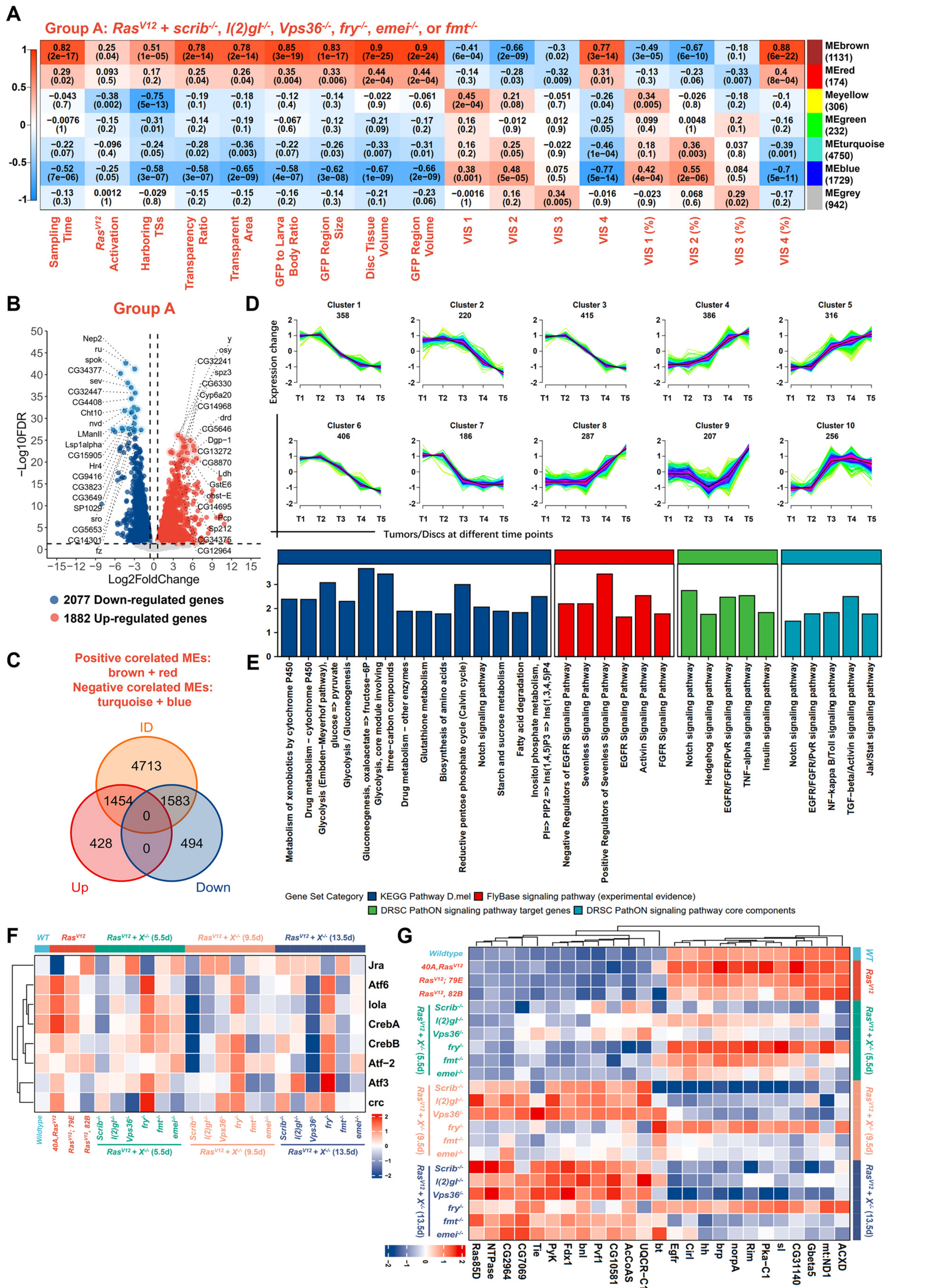

A
Group A: Ras^V12 + scrib-/-, l(2)gl-/-, Vps36-/-, fry-/-, emei-/-, or fmt-/-
0.82 (2e−17) 0.25 (0.04) 0.51 (1e−05) 0.78 (2e−14) 0.78 (2e−14) 0.85 (3e−19) 0.83 (1e−17) 0.9 (7e−25) 0.9 (2e−24) −0.41 (6e−04) −0.66 (2e−09) −0.3 (0.02) 0.77 (3e−14) −0.49 (3e−05) −0.67 (6e−10) −0.18 (0.1) 0.88 (6e−22)
0.29 (0.02) 0.093 (0.5) 0.17 (0.2) 0.25 (0.04) 0.26 (0.04) 0.35 (0.004) 0.33 (0.006) 0.44 (2e−04) 0.44 (2e−04) −0.14 (0.3) −0.28 (0.03) −0.32 (0.009) 0.31 (0.01) −0.13 (0.3) −0.23 (0.06) −0.33 (0.007) 0.4 (8e−04)
−0.043 (0.7) −0.38 (0.002) −0.75 (5e−13) −0.19 (0.1) −0.18 (0.1) −0.12 (0.4) −0.14 (0.3) −0.022 (0.9) −0.061 (0.6) 0.45 (2e−04) 0.21 (0.08) −0.051 (0.7) −0.26 (0.04) 0.34 (0.005) −0.026 (0.8) −0.18 (0.2) −0.1 (0.4)
−0.0076 (1) −0.15 (0.2) −0.31 (0.01) −0.14 (0.2) −0.19 (0.1) −0.067 (0.6) −0.12 (0.3) −0.21 (0.09) −0.17 (0.2) 0.16 (0.2) −0.012 (0.9) 0.012 (0.9) −0.25 (0.05) 0.099 (0.4) 0.0048 (1) 0.2 (0.1) −0.16 (0.2)
−0.22 (0.07) −0.096 (0.4) −0.24 (0.05) −0.28 (0.02) −0.36 (0.003) −0.22 (0.07) −0.26 (0.03) −0.33 (0.007) −0.31 (0.01) 0.16 (0.2) 0.25 (0.05) −0.022 (0.9) −0.46 (1e−04) 0.18 (0.1) 0.36 (0.003) 0.037 (0.8) −0.39 (0.001)
−0.52 (7e−06) −0.25 (0.05) −0.58 (3e−07) −0.58 (3e−07) −0.65 (2e−09) −0.58 (4e−07) −0.62 (3e−08) −0.67 (1e−09) −0.66 (2e−09) 0.38 (0.001) 0.48 (5e−05) 0.075 (0.5) −0.77 (5e−14) 0.42 (4e−04) 0.55 (2e−06) 0.084 (0.5) −0.7 (5e−11)
−0.13 (0.3) 0.0012 (1) −0.029 (0.8) −0.15 (0.2) −0.15 (0.2) −0.16 (0.2) −0.14 (0.3) −0.21 (0.1) −0.23 (0.06) −0.0016 (1) 0.16 (0.2) 0.34 (0.005) −0.016 (0.9) −0.023 (0.9) 0.068 (0.6) 0.29 (0.02) −0.17 (0.2)
MEbrown (1131)
MEred (174)
MEyellow (306)
MEgreen (232)
MEturquoise (4750)
MEblue (1729)
MEgrey (942)
Sampling Time
Ras^V12 Activation
Harboring TSs
Transparency Ratio
Transparent Area
GFP to Larva Body Ratio
GFP Region Size
Disc Tissue Volume
GFP Region Volume
VIS 1
VIS 2
VIS 3
VIS 4
VIS 1 (%)
VIS 2 (%)
VIS 3 (%)
VIS 4 (%)
B
Group A
−Log10FDR
Log2FoldChange
Nep2 ru spok CG34377 sev CG32447 CG4408 Cht10 nvd LManII Lsp1alpha CG15905 Hr4 CG9416 CG3823 CG3649 SP1029 sro CG5653 CG14301 fz
y osy CG32241 spz3 CG6330 Cyp6a20 CG14968 drd CG5646 Dgp−1 CG13272 CG8870 Ldh GstE6 obst−E CG14695 Pcp Sp212 CG34375 CG12964
2077 Down-regulated genes
1882 Up-regulated genes
C
Positive corelated MEs: brown + red
Negative corelated MEs: turquoise + blue
ID
Up
Down
4713 1454 1583 0 428 0 494
D
Expression change
Cluster 1 358
Cluster 2 220
Cluster 3 415
Cluster 4 386
Cluster 5 316
Cluster 6 406
Cluster 7 186
Cluster 8 287
Cluster 9 207
Cluster 10 256
T1 T2 T3 T4 T5
Tumors/Discs at different time points
E
Metabolism of xenobiotics by cytochrome P450
Drug metabolism − cytochrome P450
Glycolysis (Embden−Meyerhof pathway), glucose => pyruvate
Glycolysis / Gluconeogenesis
Gluconeogenesis, oxaloacetate => fructose−6P
Glycolysis, core module involving three−carbon compounds
Drug metabolism − other enzymes
Glutathione metabolism
Biosynthesis of amino acids
Reductive pentose phosphate cycle (Calvin cycle)
Notch signaling pathway
Starch and sucrose metabolism
Fatty acid degradation
Inositol phosphate metabolism, PI=> PIP2 => Ins(1,4,5)P3 => Ins(1,3,4,5)P4
Negative Regulators of EGFR Signaling Pathway
Sevenless Signaling Pathway
Positive Regulators of Sevenless Signaling Pathway
EGFR Signaling Pathway
Activin Signaling Pathway
FGFR Signaling Pathway
Notch signaling pathway
Hedgehog signaling pathway
EGFR/FGFR/PvR signaling pathway
TNF−alpha signaling pathway
Insulin signaling pathway
Notch signaling pathway
EGFR/FGFR/PvR signaling pathway
NF−kappa B/Toll signaling pathway
TGF−beta/Activin signaling pathway
Jak/Stat signaling pathway
Gene Set Category KEGG Pathway D.mel FlyBase signaling pathway (experimental evidence) DRSC PathON signaling pathway target genes DRSC PathON signaling pathway core components
F
WT Ras^V12 Ras^V12 + X-/- (5.5d) Ras^V12 + X-/- (9.5d) Ras^V12 + X-/- (13.5d)
Jra
Atf6
lola
CrebA
CrebB
Atf−2
Atf3
crc
Wildtype 40A,Ras^V12 Ras^V12; 79E Ras^V12; 82B Scrib-/- l(2)gl-/- Vps36-/- fry-/- fmt-/- emei-/-
G
Wildtype
40A,Ras^V12
Ras^V12; 79E
Ras^V12, 82B
Scrib-/- l(2)gl-/- Vps36-/- fry-/- fmt-/- emei-/-
Ras^V12 + X-/- (5.5d)
Ras^V12 + X-/- (9.5d)
Ras^V12 + X-/- (13.5d)
Ras85D NTPase CG2964 CG7069 Tie PyK Fdx1 bnl Pvf1 CG10581 AcCoAS UQCR−C1 bt Egfr Ciril hh brp norpA Rim Pka−C1 sl CG31140 Gbeta5 mt:ND1 ACXD

Figure EV2. Dynamic transcriptomic profiles of Group A *Drosophila Ras*-driven tumors.

(**A**) The WGCNA heatmap depicting the correlations between identified gene modules and phenotypic malignancies of Group A *Drosophila Ras*-driven tumors. The color intensity of each module represented the correlation coefficient, which was also displayed in the top line of the respective cell. Pearson correlation analysis was conducted to assess the associations between module eigengenes (MEs) and phenotypic data. The corresponding *P* value was shown in parentheses, with *P* values below 0.05 indicating statistically significant correlations. (**B**) The volcano plot of DEGs (T2 vs T5) in Group A of *Drosophila Ras*-driven tumors. The upregulated and downregulated genes were 1882 and 2077, respectively. (**C**) The Venn diagram depicting the overlap of genes in modules significantly associated with tumor malignancies (**A**) and DEGs (**B**). In total, 1454 genes were upregulated and significantly associated with tumor malignancies; 1583 genes were downregulated and significantly associated with tumor malignancies. (**D**) The line charts depicting dynamic transcriptomic changes of DEGs significantly associated with tumor malignancies in Group A *Drosophila Ras*-driven tumors (**C**), revealed by Mfuzz. (**E**) The bar plot depicting the functional annotations of candidate genes obtained from certain clusters (**D**), revealed by PANGEA. (**F**) The heatmap depicting the expression profiles of enriched TFs from T1 to T5. The TF enrichment of candidate genes derived from specific clusters (**D**) was performed using RcisTarget. (**G**) The heatmap depicting the expression profiles of identified hub genes from T1 to T5. The hub gene identification among candidate genes from specific clusters (**D**) was performed via STRING and BottleNeck.

 © The Author(s)

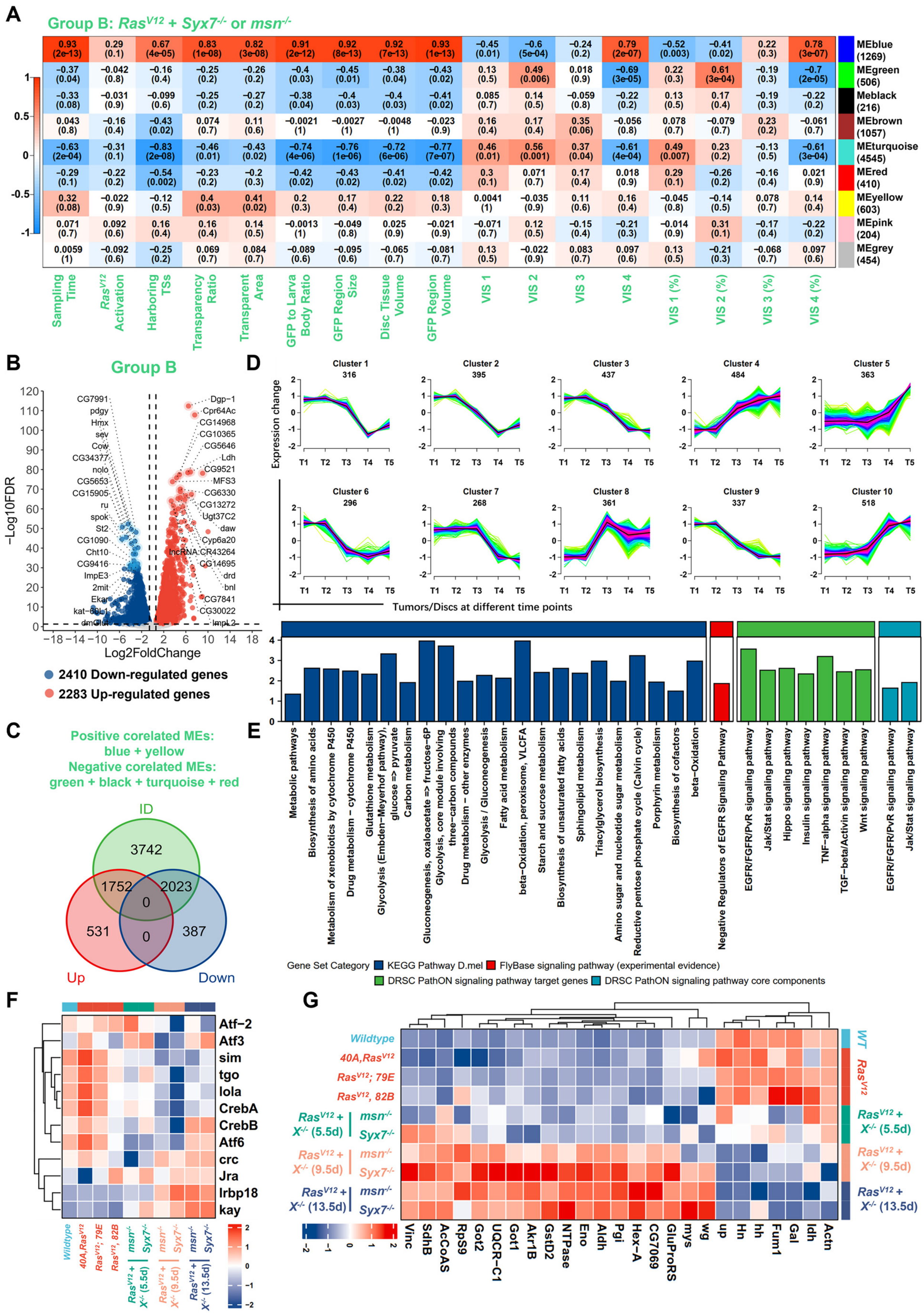
A
Group B: Ras^{V12} + $Syx7^{-/-}$ or $msn^{-/-}$
0.93 (2e-13) 0.29 (0.1) 0.67 (4e-05) 0.83 (1e-08) 0.82 (3e-08) 0.91 (2e-12) 0.92 (8e-13) 0.92 (7e-13) 0.93 (1e-13) −0.45 (0.01) −0.6 (5e-04) −0.24 (0.2) 0.79 (2e-07) −0.52 (0.003) −0.41 (0.02) 0.22 (0.3) 0.78 (3e-07)
−0.37 (0.04) −0.042 (0.8) −0.16 (0.4) −0.25 (0.2) −0.26 (0.2) −0.4 (0.03) −0.45 (0.01) −0.38 (0.04) −0.43 (0.02) 0.13 (0.5) 0.49 (0.006) 0.018 (0.9) −0.69 (3e-05) 0.22 (0.3) 0.61 (3e-04) −0.19 (0.3) −0.7 (2e-05)
−0.33 (0.08) −0.031 (0.9) −0.099 (0.6) −0.25 (0.2) −0.27 (0.2) −0.38 (0.04) −0.4 (0.03) −0.4 (0.03) −0.41 (0.02) 0.085 (0.7) 0.14 (0.5) −0.059 (0.8) −0.22 (0.2) 0.13 (0.5) 0.17 (0.4) −0.19 (0.3) −0.22 (0.2)
0.043 (0.8) −0.16 (0.4) −0.43 (0.02) 0.074 (0.7) 0.11 (0.6) −0.0021 (1) −0.0027 (1) −0.0048 (1) −0.023 (0.9) 0.16 (0.4) 0.17 (0.4) 0.35 (0.06) −0.056 (0.8) 0.078 (0.7) −0.079 (0.7) 0.23 (0.2) −0.061 (0.7)
−0.63 (2e-04) −0.31 (0.1) −0.83 (2e-08) −0.46 (0.01) −0.43 (0.02) −0.74 (4e-06) −0.76 (1e-06) −0.72 (6e-06) −0.77 (7e-07) 0.46 (0.01) 0.56 (0.001) 0.37 (0.04) −0.61 (4e-04) 0.49 (0.007) 0.23 (0.2) −0.13 (0.5) −0.61 (3e-04)
−0.29 (0.1) −0.22 (0.2) −0.54 (0.002) −0.23 (0.2) −0.2 (0.3) −0.42 (0.02) −0.43 (0.02) −0.41 (0.02) −0.42 (0.02) 0.3 (0.1) 0.071 (0.7) 0.17 (0.4) 0.018 (0.9) 0.29 (0.1) −0.26 (0.2) −0.16 (0.4) 0.021 (0.9)
0.32 (0.08) −0.022 (0.9) −0.12 (0.5) 0.4 (0.03) 0.41 (0.02) 0.2 (0.3) 0.17 (0.4) 0.22 (0.2) 0.18 (0.3) 0.0041 (1) −0.035 (0.9) 0.11 (0.6) 0.16 (0.4) −0.045 (0.8) −0.14 (0.5) 0.078 (0.7) 0.14 (0.4)
0.071 (0.7) 0.092 (0.6) 0.16 (0.4) 0.16 (0.4) 0.14 (0.5) −0.0013 (1) −0.049 (0.8) 0.025 (0.9) −0.021 (0.9) −0.071 (0.7) 0.12 (0.5) −0.15 (0.4) −0.21 (0.3) −0.014 (0.9) 0.31 (0.1) −0.17 (0.4) −0.22 (0.2)
0.0059 (1) −0.092 (0.6) −0.25 (0.2) 0.069 (0.7) 0.084 (0.7) −0.089 (0.6) −0.095 (0.6) −0.065 (0.7) −0.081 (0.7) 0.13 (0.5) −0.022 (0.9) 0.083 (0.7) 0.097 (0.6) 0.13 (0.5) −0.21 (0.3) −0.068 (0.7) 0.097 (0.6)
MEblue (1269)
MEgreen (506)
Meblack (216)
MEbrown (1057)
MEturquoise (4545)
MEred (410)
MEyellow (603)
MEpink (204)
MEgrey (454)
1 0.5 0 -0.5 -1
Sampling Time
Ras^{V12} Activation
Harboring TSs
Transparency Ratio
Transparent Area
GFP to Larva Body Ratio
GFP Region Size
Disc Tissue Volume
GFP Region Volume
VIS 1
VIS 2
VIS 3
VIS 4
VIS 1 (%)
VIS 2 (%)
VIS 3 (%)
VIS 4 (%)
B
Group B
−Log10FDR
Log2FoldChange
CG7991 pdgy Hmx sev Cow CG34377 nolo CG5653 CG15905 ru spok St2 CG1090 Cht10 CG9416 ImpE3 2mit Ekar kat-60L1 dmGlut
Dgp-1 Cpr64Ac CG14968 CG10365 CG5646 Ldh CG9521 MFS3 CG6330 CG13272 Ugt37C2 daw Cyp6a20 lncRNA:CR43264 CG14695 drd bnl CG7841 CG30022 ImpL2
2410 Down-regulated genes
2283 Up-regulated genes
C
Positive corelated MEs: blue + yellow
Negative corelated MEs: green + black + turquoise + red
ID
3742
1752
2023
0
531
0
387
Up
Down
D
Cluster 1 316
Cluster 2 395
Cluster 3 437
Cluster 4 484
Cluster 5 363
Cluster 6 296
Cluster 7 268
Cluster 8 361
Cluster 9 337
Cluster 10 518
Expression change
T1 T2 T3 T4 T5
Tumors/Discs at different time points
E
Metabolic pathways
Biosynthesis of amino acids
Metabolism of xenobiotics by cytochrome P450
Drug metabolism − cytochrome P450
Glutathione metabolism
Glycolysis (Embden−Meyerhof pathway), glucose => pyruvate
Carbon metabolism
Gluconeogenesis, oxaloacetate => fructose−6P
Glycolysis, core module involving three−carbon compounds
Drug metabolism − other enzymes
Glycolysis / Gluconeogenesis
Fatty acid metabolism
beta−Oxidation, peroxisome, VLCFA
Starch and sucrose metabolism
Biosynthesis of unsaturated fatty acids
Sphingolipid metabolism
Triacylglycerol biosynthesis
Amino sugar and nucleotide sugar metabolism
Reductive pentose phosphate cycle (Calvin cycle)
Porphyrin metabolism
Biosynthesis of cofactors
beta−Oxidation
Negative Regulators of EGFR Signaling Pathway
EGFR/FGFR/PvR signaling pathway
Jak/Stat signaling pathway
Hippo signaling pathway
Insulin signaling pathway
TNF−alpha signaling pathway
TGF−beta/Activin signaling pathway
Wnt signaling pathway
EGFR/FGFR/PvR signaling pathway
Jak/Stat signaling pathway
Gene Set Category KEGG Pathway D.mel FlyBase signaling pathway (experimental evidence) DRSC PathON signaling pathway target genes DRSC PathON signaling pathway core components
F
Atf−2 Atf3 sim tgo lola CrebA CrebB Atf6 crc Jra Irbp18 kay
Wildtype
40A,Ras^{V12}
Ras^{V12}; 79E
Ras^{V12}, 82B
Ras^{V12} + $X^{-/-}$ (5.5d) $msn^{-/-}$ $Syx7^{-/-}$
Ras^{V12} + $X^{-/-}$ (9.5d) $msn^{-/-}$ $Syx7^{-/-}$
Ras^{V12} + $X^{-/-}$ (13.5d) $msn^{-/-}$ $Syx7^{-/-}$
2 1 0 −1 −2
G
Wildtype
40A,Ras^{V12}
Ras^{V12}; 79E
Ras^{V12}, 82B
Ras^{V12} + $X^{-/-}$ (5.5d) $msn^{-/-}$ $Syx7^{-/-}$
Ras^{V12} + $X^{-/-}$ (9.5d) $msn^{-/-}$ $Syx7^{-/-}$
Ras^{V12} + $X^{-/-}$ (13.5d) $msn^{-/-}$ $Syx7^{-/-}$
WT
Ras^{V12}
Ras^{V12} + $X^{-/-}$ (5.5d)
Ras^{V12} + $X^{-/-}$ (9.5d)
Ras^{V12} + $X^{-/-}$ (13.5d)
−2 −1 0 1 2
Vinc SdhB AcCoAS RpS9 Got2 UQCR−C1 Got1 Akr1B GstD2 NTPase Eno Aldh Pgi Hex−A CG7069 GluProRS mys wg up Hn hh Fum1 Gal Idh Actn

Figure EV3. Dynamic transcriptomic profiles of Group B *Drosophila Ras*-driven tumors.

(**A**) The WGCNA heatmap depicting the correlations between identified gene modules and phenotypic malignancies of Group B *Drosophila Ras*-driven tumors. The color intensity of each module represented the correlation coefficient, which was also displayed in the top line of the respective cell. Pearson correlation analysis was conducted to assess the associations between module eigengenes (MEs) and phenotypic data. The corresponding *P* value was shown in parentheses, with *P* values below 0.05 indicating statistically significant correlations. (**B**) The volcano plot of DEGs (T2 vs T5) in Group B of *Drosophila Ras*-driven tumors. The upregulated and downregulated genes were 2283 and 2410, respectively. (**C**) The Venn diagram depicting the overlap of genes in modules significantly associated with tumor malignancies (**A**) and DEGs (**B**). In all, 1752 genes were upregulated and significantly associated with tumor malignancies; 2023 genes were downregulated and significantly associated with tumor malignancies. (**D**) The line charts depicting dynamic transcriptomic changes of DEGs significantly associated with tumor malignancies in Group B *Drosophila Ras*-driven tumors (**C**), revealed by Mfuzz. (**E**) The bar plot depicting the functional annotations of candidate genes obtained from certain clusters (**D**), revealed by PANGEA. (**F**) The heatmap depicting the expression profiles of enriched TFs from T1 to T5. The TF enrichment of candidate genes derived from specific clusters (**D**) was performed using RcisTarget. (**G**) The heatmap depicting the expression profiles of identified hub genes from T1 to T5. The hub gene identification among candidate genes from specific clusters (**D**) was performed via STRING and BottleNeck.

 © The Author(s)

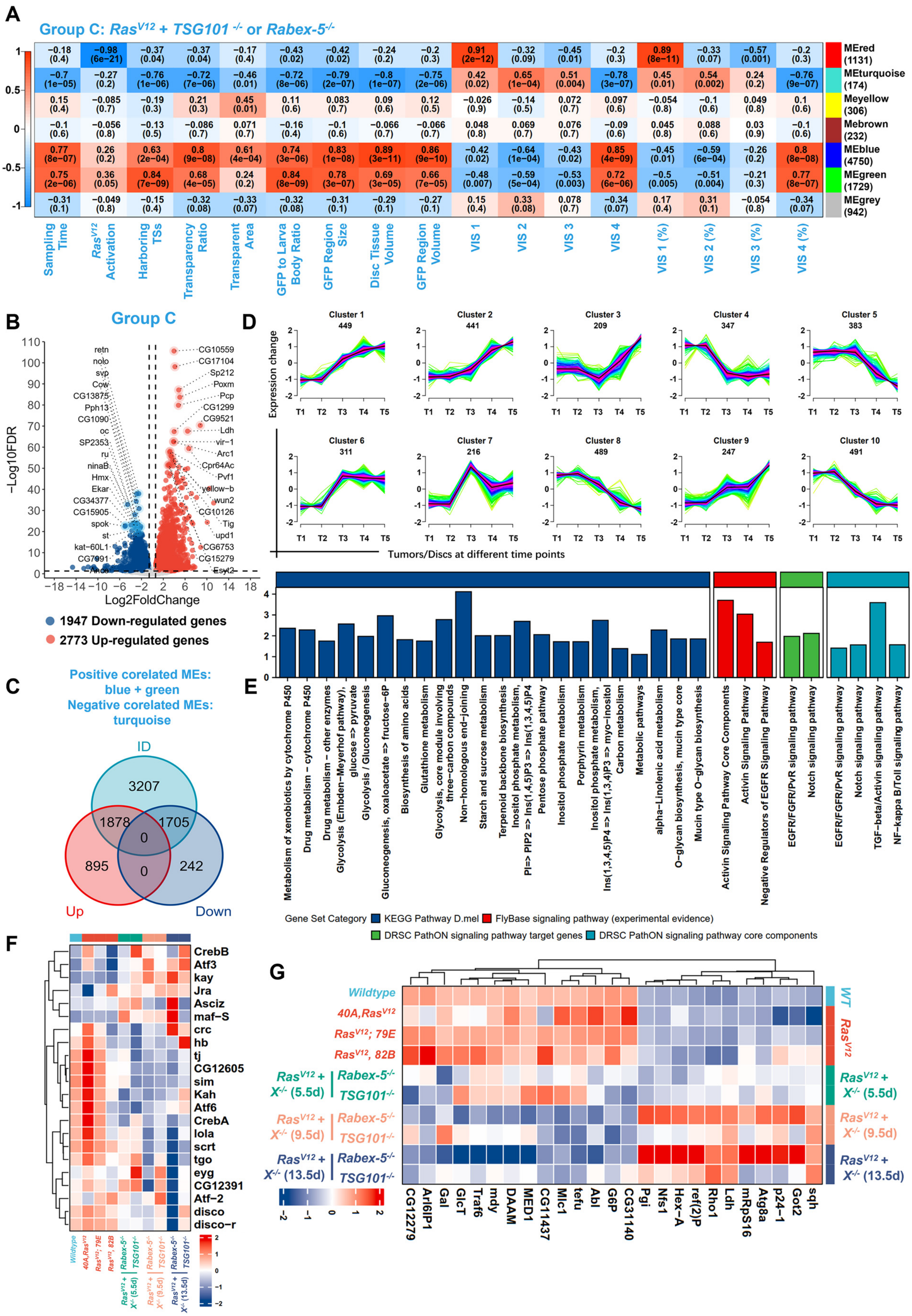
A
Group C: Ras^{V12} + $TSG101^{-/-}$ or $Rabex-5^{-/-}$
−0.18 (0.4) −0.98 (6e−21) −0.37 (0.04) −0.37 (0.04) −0.17 (0.4) −0.43 (0.02) −0.42 (0.02) −0.24 (0.2) −0.2 (0.3) 0.91 (2e−12) −0.32 (0.09) −0.45 (0.01) −0.2 (0.3) 0.89 (8e−11) −0.33 (0.07) −0.57 (0.001) −0.2 (0.3)
−0.7 (1e−05) −0.27 (0.2) −0.76 (1e−06) −0.72 (7e−06) −0.46 (0.01) −0.72 (8e−06) −0.79 (2e−07) −0.8 (1e−07) −0.75 (2e−06) 0.42 (0.02) 0.65 (1e−04) 0.51 (0.004) −0.78 (3e−07) 0.45 (0.01) 0.54 (0.002) 0.24 (0.2) −0.76 (9e−07)
0.15 (0.4) −0.085 (0.7) −0.19 (0.3) 0.21 (0.3) 0.45 (0.01) 0.11 (0.6) 0.083 (0.7) 0.09 (0.6) 0.12 (0.5) −0.026 (0.9) −0.14 (0.5) 0.072 (0.7) 0.097 (0.6) −0.054 (0.8) −0.1 (0.6) 0.049 (0.8) 0.1 (0.6)
−0.1 (0.6) −0.056 (0.8) −0.13 (0.5) −0.086 (0.7) 0.071 (0.7) −0.16 (0.4) −0.1 (0.6) −0.066 (0.7) −0.066 (0.7) 0.048 (0.8) 0.069 (0.7) 0.076 (0.7) −0.09 (0.6) 0.045 (0.8) 0.088 (0.6) 0.03 (0.9) −0.1 (0.6)
0.77 (8e−07) 0.26 (0.2) 0.63 (2e−04) 0.8 (9e−08) 0.61 (4e−04) 0.74 (3e−06) 0.83 (1e−08) 0.89 (3e−11) 0.86 (9e−10) −0.42 (0.02) −0.64 (1e−04) −0.43 (0.02) 0.85 (4e−09) −0.45 (0.01) −0.59 (6e−04) −0.26 (0.2) 0.8 (8e−08)
0.75 (2e−06) 0.36 (0.05) 0.84 (7e−09) 0.68 (4e−05) 0.24 (0.2) 0.84 (8e−09) 0.78 (3e−07) 0.69 (3e−05) 0.66 (7e−05) −0.48 (0.007) −0.59 (5e−04) −0.53 (0.003) 0.72 (6e−06) −0.5 (0.005) −0.51 (0.004) −0.21 (0.3) 0.77 (8e−07)
−0.31 (0.1) −0.049 (0.8) −0.15 (0.4) −0.32 (0.08) −0.33 (0.07) −0.32 (0.08) −0.31 (0.1) −0.29 (0.1) −0.27 (0.1) 0.15 (0.4) 0.33 (0.08) 0.078 (0.7) −0.34 (0.07) 0.17 (0.4) 0.31 (0.1) −0.054 (0.8) −0.34 (0.07)
MEred (1131)
MEturquoise (174)
Meyellow (306)
Mebrown (232)
MEblue (4750)
MEgreen (1729)
MEgrey (942)
Sampling Time
Ras^{V12} Activation
Harboring TSs
Transparency Ratio
Transparent Area
GFP to Larva Body Ratio
GFP Region Size
Disc Tissue Volume
GFP Region Volume
VIS 1
VIS 2
VIS 3
VIS 4
VIS 1 (%)
VIS 2 (%)
VIS 3 (%)
VIS 4 (%)
B
Group C
−Log10FDR
Log2FoldChange
retn, nolo, svp, Cow, CG13875, Pph13, CG1090, oc, SP2353, ru, ninaB, Hmx, Ekar, CG34377, CG15905, spok, st, kat−60L1, CG7091, Anox
CG10559, CG17104, Sp212, Poxm, Pcp, CG1299, CG9521, Ldh, vir−1, Arc1, Cpr64Ac, Pvf1, yellow−b, wun2, CG10126, Tig, upd1, CG6753, CG15279, Esyt2
1947 Down-regulated genes
2773 Up-regulated genes
C
Positive corelated MEs: blue + green
Negative corelated MEs: turquoise
ID
3207
1878
1705
0
895
0
242
Up
Down
D
Cluster 1 449
Cluster 2 441
Cluster 3 209
Cluster 4 347
Cluster 5 383
Cluster 6 311
Cluster 7 216
Cluster 8 489
Cluster 9 247
Cluster 10 491
Expression change
Tumors/Discs at different time points
E
Metabolism of xenobiotics by cytochrome P450
Drug metabolism − cytochrome P450
Drug metabolism − other enzymes
Glycolysis (Embden−Meyerhof pathway), glucose => pyruvate
Glycolysis / Gluconeogenesis
Gluconeogenesis, oxaloacetate => fructose−6P
Biosynthesis of amino acids
Glutathione metabolism
Glycolysis, core module involving three−carbon compounds
Non−homologous end−joining
Starch and sucrose metabolism
Terpenoid backbone biosynthesis
Inositol phosphate metabolism, PI=> PIP2 => Ins(1,4,5)P3 => Ins(1,3,4,5)P4
Pentose phosphate pathway
Inositol phosphate metabolism
Porphyrin metabolism
Inositol phosphate metabolism, Ins(1,3,4,5)P4 => Ins(1,3,4)P3 => myo−inositol
Carbon metabolism
Metabolic pathways
alpha−Linolenic acid metabolism
O−glycan biosynthesis, mucin type core
Mucin type O−glycan biosynthesis
Activin Signaling Pathway Core Components
Activin Signaling Pathway
Negative Regulators of EGFR Signaling Pathway
EGFR/FGFR/PvR signaling pathway
Notch signaling pathway
EGFR/FGFR/PvR signaling pathway
Notch signaling pathway
TGF−beta/Activin signaling pathway
NF−kappa B/Toll signaling pathway
Gene Set Category
KEGG Pathway D.mel
FlyBase signaling pathway (experimental evidence)
DRSC PathON signaling pathway target genes
DRSC PathON signaling pathway core components
F
CrebB, Atf3, kay, Jra, Asciz, maf−S, crc, hb, tj, CG12605, sim, Kah, Atf6, CrebA, lola, scrt, tgo, eyg, CG12391, Atf−2, disco, disco−r
Wildtype
40A,Ras^{V12}
Ras^{V12}; 79E
Ras^{V12}, 82B
Ras^{V12} + $X^{-/-}$ (5.5d) | $Rabex-5^{-/-}$ | $TSG101^{-/-}$
Ras^{V12} + $X^{-/-}$ (9.5d) | $Rabex-5^{-/-}$ | $TSG101^{-/-}$
Ras^{V12} + $X^{-/-}$ (13.5d) | $Rabex-5^{-/-}$ | $TSG101^{-/-}$
G
Wildtype
40A,Ras^{V12}
Ras^{V12}; 79E
Ras^{V12}, 82B
Ras^{V12} + $X^{-/-}$ (5.5d) | $Rabex-5^{-/-}$ | $TSG101^{-/-}$
Ras^{V12} + $X^{-/-}$ (9.5d) | $Rabex-5^{-/-}$ | $TSG101^{-/-}$
Ras^{V12} + $X^{-/-}$ (13.5d) | $Rabex-5^{-/-}$ | $TSG101^{-/-}$
WT
Ras^{V12}
Ras^{V12} + $X^{-/-}$ (5.5d)
Ras^{V12} + $X^{-/-}$ (9.5d)
Ras^{V12} + $X^{-/-}$ (13.5d)
CG12279, Arl6IP1, Gal, GlcT, Traf6, mdy, DAAM, MED1, CG11437, Mlc1, tefu, Abl, G6P, CG31140, Pgi, Nfs1, Hex−A, ref(2)P, Rho1, Ldh, mRpS16, Atg8a, p24−1, Got2, sqh

Figure EV4. Dynamic transcriptomic profiles of Group C *Drosophila Ras*-driven tumors.

(**A**) The WGCNA heatmap depicting the correlations between identified gene modules and phenotypic malignancies of Group C *Drosophila Ras*-driven tumors. The color intensity of each module represented the correlation coefficient, which was also displayed in the top line of the respective cell. Pearson correlation analysis was conducted to assess the associations between module eigengenes (MEs) and phenotypic data. The corresponding *P* value was shown in parentheses, with *P* values below 0.05 indicating statistically significant correlations. (**B**) The volcano plot of DEGs (T2 vs T5) in Group C of *Drosophila Ras*-driven tumors. The upregulated and downregulated genes were 2773 and 1947, respectively. (**C**) The Venn diagram depicting the overlap of genes in modules significantly associated with tumor malignancies (**A**) and DEGs (**B**). In all, 1878 genes were upregulated and significantly associated with tumor malignancies; 1705 genes were downregulated and significantly associated with tumor malignancies. (**D**) The line charts depicting dynamic transcriptomic changes of DEGs significantly associated with tumor malignancies in Group C *Drosophila Ras*-driven tumors (**C**), revealed by Mfuzz. (**E**) The bar plot depicting the functional annotations of candidate genes obtained from certain clusters (**D**), revealed by PANGEA. (**F**) The heatmap depicting the expression profiles of enriched TFs from T1 to T5. The TF enrichment of candidate genes derived from specific clusters (**D**) was performed using RcisTarget. (**G**) The heatmap depicting the expression profiles of identified hub genes from T1 to T5. The hub gene identification among candidate genes from specific clusters (**D**) was performed via STRING and BottleNeck.

 © The Author(s)

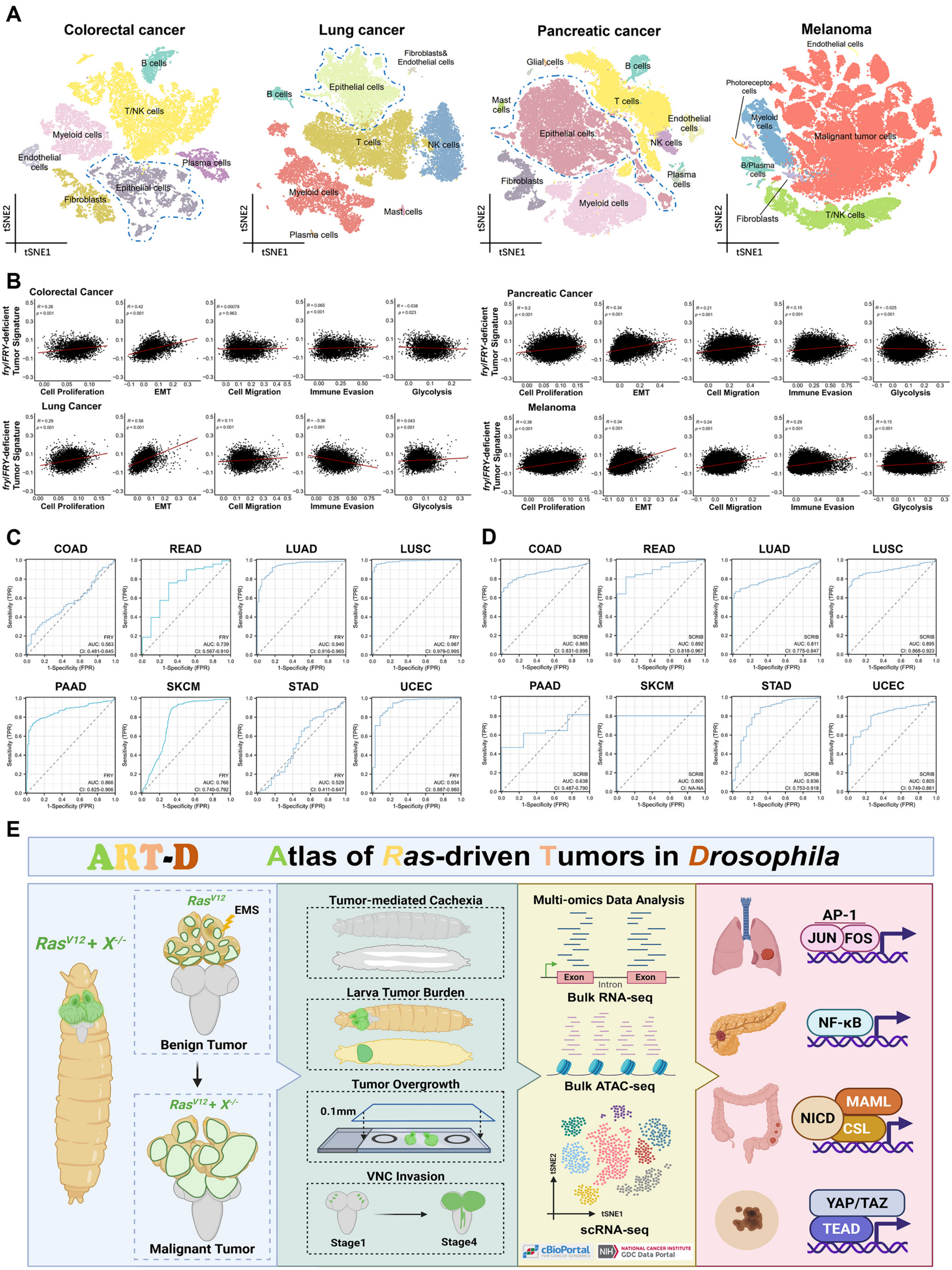
A
Colorectal cancer
B cells
T/NK cells
Myeloid cells
Endothelial cells
Plasma cells
Epithelial cells
Fibroblasts
tSNE2
tSNE1
Lung cancer
Fibroblasts& Endothelial cells
B cells
Epithelial cells
T cells
NK cells
Myeloid cells
Mast cells
Plasma cells
Pancreatic cancer
Glial cells
B cells
Mast cells
T cells
Epithelial cells
Endothelial cells
NK cells
Plasma cells
Fibroblasts
Myeloid cells
Melanoma
Endothelial cells
Photoreceptor cells
Myeloid cells
Malignant tumor cells
B/Plasma cells
Fibroblasts
T/NK cells
B
Colorectal Cancer
Pancreatic Cancer
Lung Cancer
Melanoma
fry/FRY-deficient Tumor Signature
Cell Proliferation
EMT
Cell Migration
Immune Evasion
Glycolysis
C
COAD
READ
LUAD
LUSC
PAAD
SKCM
STAD
UCEC
Sensitivity (TPR)
1-Specificity (FPR)
FRY
AUC: 0.563
CI: 0.481-0.645
AUC: 0.739
CI: 0.567-0.910
AUC: 0.940
CI: 0.916-0.965
AUC: 0.987
CI: 0.979-0.995
AUC: 0.866
CI: 0.825-0.906
AUC: 0.766
CI: 0.740-0.792
AUC: 0.529
CI: 0.411-0.647
AUC: 0.934
CI: 0.887-0.980
D
COAD
READ
LUAD
LUSC
PAAD
SKCM
STAD
UCEC
SCRIB
AUC: 0.865
CI: 0.831-0.898
AUC: 0.892
CI: 0.818-0.967
AUC: 0.811
CI: 0.775-0.847
AUC: 0.895
CI: 0.868-0.923
AUC: 0.638
CI: 0.487-0.790
AUC: 0.805
CI: NA-NA
AUC: 0.836
CI: 0.753-0.918
AUC: 0.805
CI: 0.749-0.861
E
ART-D
Atlas of Ras-driven Tumors in Drosophila
RasV12 + X-/-
RasV12
EMS
Benign Tumor
RasV12 + X-/-
Malignant Tumor
Tumor-mediated Cachexia
Larva Tumor Burden
Tumor Overgrowth
0.1mm
VNC Invasion
Stage1
Stage4
Multi-omics Data Analysis
Exon
Intron
Exon
Bulk RNA-seq
Bulk ATAC-seq
tSNE2
tSNE1
scRNA-seq
cBioPortal
NIH
NATIONAL CANCER INSTITUTE GDC Data Portal
AP-1
JUN
FOS
NF-κB
MAML
NICD
CSL
YAP/TAZ
TEAD

Figure EV5. Cross-species insights from ART-D on evolutionarily conserved oncogenic mechanisms.

(**A**) Single-cell data analysis for human *KRAS/NRAS*-driven cancers. A total of 174,385 high-quality cells were maintained, with 18,727 from colorectal cancer (CRC carrying *KRAS* oncogenic mutations), 25,059 from lung cancer (LUAD), 45,747 from pancreatic cancer (PAAD carrying *KRAS* oncogenic mutations), and 84,825 from melanoma, respectively. The corresponding cell type was annotated. (**B**) The correlation scatter plots illustrate the relationships between *fry/FRY*-deficient tumor signature scores and tumor malignancies scores across *KRAS/NRAS*-driven cancers, CRC, LUAD, PAAD, and melanoma. These correlations were determined using Spearman's correlation analysis, with coefficients R and *P* values reported, where $P < 0.05$ indicates statistical significance. (**C**, **D**) The ROC evaluation of *FRY* (**C**) and *SCRIB* (**D**) diagnostic value across *KRAS/NRAS*-driven cancers. The AUC values of each cancer were presented with confidence intervals. The AUC higher than 0.85 was considered excellent performance. (**E**) The proposed model of this study. The model illustrated the utilization of ART-D phenotypic malignancies and dynamic transcriptomic profiles to uncover the oncogenic mechanisms driving tumorigenesis in *Drosophila Ras*-driven tumors. Subsequent integrated multi-omics data analysis revealed an evolutionarily conserved role of oncogenic signaling pathways in human *KRAS/NRAS*-driven cancers. The cross-species findings demonstrated strong performance in diagnosis and prediction of human cancers. By integrating *Drosophila* genetics with human oncology, ART-D offers a valuable resource for identifying tumor stage-specific therapeutic targets and advancing the treatment of RAS-driven malignancies.

 © The Author(s)
