# Supplementary material for: Cross-species insights from ART-D to uncover evolutionarily conserved oncogenic mechanisms
Source: Mol Syst Biol. 2026 May 14;22(8):1214–51. doi: 10.1038/s44320-026-00215-8 (PMC13433787; doi:10.1038/s44320-026-00215-8)
Supplement: Supplementary file 2 [file 44320_2026_215_MOESM1_ESM.pdf]

Cross-Species Insights from ART-D to Uncover Evolutionarily Conserved Oncogenic Mechanisms

Yifan Guo, Jiadong Zheng, Yixin Wu, Kun Zhao, Dian Lv, Wenhan Liu, Mengling Wang, Jinyu Lu, Wenyan Xu, Xianping Wang, and Xianjue Ma

Corresponding author(s): Xianjue Ma (maxianjue@westlake.edu.cn) , Xianping Wang (wangxianping@westlake.edu.cn), Yifan Guo (guoyifan@westlake.edu.cn), Wenyan Xu (xuwenyan@westlake.edu.cn)

Review Timeline:

Submission Date:	17th Oct 25
Editorial Decision:	5th Dec 25
Revision Received:	18th Mar 26
Editorial Decision:	17th Apr 26
Revision Received:	21st Apr 26
Accepted:	28th Apr 26

Editor: Jingyi Hou

Transaction Report:

(Note: With the exception of the correction of typographical or spelling errors that could be a source of ambiguity, letters and reports are not edited. Depending on transfer agreements, referee reports obtained elsewhere may or may not be included in this compilation. Referee reports are anonymous unless the Referee chooses to sign their reports.)

5th Dec 2025

Manuscript Number: MSB-2025-13410
Title: Cross-Species Insights from ART-D to Uncover Evolutionarily Conserved Oncogenic Mechanisms
Author: Yifan Guo
Jiadong Zheng
Yixin Wu
Kun Zhao
Dian Lv
Wenhan Liu
Mengling Wang
Jinyu Lu
Wenyan Xu
Xianping Wang
Xianjue Ma

Dear Xianjue,

Thank you for submitting your work to Molecular Systems Biology. First, I would like to apologise for the delay in the review process, which was due to the late arrival of the reviewers' reports. We have now received evaluations from two of the three reviewers who had agreed to assess your manuscript. Despite several reminders, we were unable to obtain a report from Reviewer #3. In the interest of time and given that the recommendations from the two received reviews are aligned, I have decided to proceed with a decision rather than delay the process further.

As you will see from the comments below, both Reviewer #1 and Reviewer #2 find the manuscript of interest and are overall supportive. They do, however, raise several issues that should be satisfactorily addressed in a revised version of your work.

Without reiterating all the reviewers' points, the key issues that require attention are as follows:

- We think Reviewer #1's request for incorporation of data on metabolic and physiological parameters goes beyond the scope of a major revision for the current manuscript. However, these aspects should be properly discussed in the text.

- Regarding Reviewer #2's comment on Figure 8, the added value of this analysis should be articulated more clearly, and any potential overstatement should be avoided.

All other issues raised by the reviewers need to be satisfactorily addressed. As you may already know, our editorial policy allows in principle a single round of major revision so it is essential to provide responses to the reviewers' comments that are as complete as possible. Please feel free to contact me in case you would like to discuss in further detail any of the issues raised by the reviewers.

On a more editorial level, we would ask you to address the following issues:

- Please provide a .docx formatted version of the manuscript text (including legends for main figures, EV figures and tables). Please make sure that the changes are highlighted to be clearly visible.

- Please provide individual production quality figure files as .eps, .tif, .jpg (one file per figure). When assembling figures, please refer to our figure preparation guideline in order to ensure proper formatting and readability in print as well as on screen: https://media.springernature.com/original/springer-cms/rest/v1/content/27825798/data/v1

-Please provide a .docx formatted letter INCLUDING the reviewers' reports and your detailed point-by-point responses to their comments. As part of the EMBO Press transparent editorial process, the point-by-point response is part of the Review Process File (RPF), which will be published alongside your paper.

-Please note that all corresponding authors are required to supply an ORCID ID for their name upon submission of a revised manuscript.

-We replaced Supplementary Information with Expanded View (EV) Figures and Tables that are collapsible/expandable online. A maximum of 5 EV Figures can be typeset. EV Figures should be cited as 'Figure EV1, Figure EV2" etc... in the text and their respective legends should be included in the main text after the legends of regular figures.

Additional Tables/Datasets should be labeled and referred to as Table EV1, Dataset EV1, etc. Legends have to be provided in a

separate tab in case of .xls files. Alternatively, the legend can be supplied as a separate text file (README) and zipped together with the Table/Dataset file.

For the figures and tables that you do NOT wish to display as Expanded View figures, they should be bundled together with their legends in a single PDF file called *Appendix*, which should start with a short Table of Content. Each legend should be below the corresponding Figure/Table in the Appendix. Appendix figures and tables should be referred to in the main text as: "Appendix Figure S1, Appendix Figure S2, Appendix Table S1" etc. See detailed instructions regarding expanded view here: https://link.springer.com/journal/44320/submission-guidelines#cms-Expanded-View-data.

-Before submitting your revision, primary datasets (and computer code, where appropriate) produced in this study need to be deposited in an appropriate public database.

Please remember to provide a reviewer password if the datasets are not yet public.
The accession numbers and database should be listed in a formal "Data Availability" section (placed after Materials & Method) that follows the model below (see also https://link.springer.com/partners/embo-press/editorial-policies#Data%20availability%20statement). Please note that the Data Availability Section is restricted to new primary data that are part of this study.

Data availability
The datasets (and computer code) produced in this study are available in the following databases:
- RNA-Seq data: Gene Expression Omnibus GSE46843 (https://www.ncbi.nlm.nih.gov/geo/query/acc.cgi?acc=GSE46843)
- [data type]: [name of the resource] [accession number/identifier/doi] ([URL or identifiers.org/DATABASE:ACCESSION])
*** Note - All links should resolve to a page where the data can be accessed. ***

- At EMBO Press we ask authors to provide source data for the main manuscript figures. You will receive a separate email with instructions for providing source data with your revised manuscript, including how to upload and organize the files.
Additional information on source data and instruction on how to label the files are available < https://link.springer.com/journal/44320/submission-guidelines#cms-Source-data >

- Our journal encourages inclusion of *data citations in the reference list* to directly cite datasets that were re-used and obtained from public databases. Data citations in the article text are distinct from normal bibliographical citations and should directly link to the database records from which the data can be accessed. In the main text, data citations are formatted as follows: "Data ref: Smith et al, 2001". In the Reference list, data citations must be labeled with "[DATASET]". A data reference must provide the database name, accession number/identifiers and a resolvable link to the landing page from which the data can be accessed at the end of the reference. Further instructions are available at < https://link.springer.com/journal/44320/submission-guidelines#cms-Reference-guidelines >.

- We updated our journal's competing interests policy in January 2022 and request authors to consider both actual and perceived competing interests. Please review the policy (https://link.springer.com/partners/embo-press/editorial-policies#Competing%20interest%20disclosures) and update your competing interests if necessary.
Please use the heading "Disclosure statement and competing interests".

- All Materials and Methods need to be described in the main text using our 'Structured Methods' format. According to this format, the Methods section includes a Reagents and Tools Table (listing key reagents, experimental models, software and relevant equipment and including their sources and relevant identifiers) followed by a Methods and Protocols section describing the methods, ideally using a step-by-step protocol format. The aim is to facilitate adoption of the methodologies across labs.

Please download and fill our Reagents and Tools Table template (.docx), which you can find in our author guidelines: https://link.springer.com/journal/44320/submission-guidelines#structuredmethods.

When submitting your revised manuscript, please do not include the Reagents and Tools Table in the Methods section of the manuscript but upload it as a separate file choosing the file type "Reagent Table".

-Regarding data quantification:
Please ensure to specify the name of the statistical test used to generate error bars and P values, the number (n) of independent experiments (please specify technical or biological replicates) underlying each data point and the test used to calculate p-values in each figure legend. Discussion of statistical methodology can be reported in the materials and methods section, but figure legends should contain a basic description of n, P and the test applied.
Graphs must include a description of the bars and the error bars (s.d., s.e.m.).
Please also include scale bars in all microscopy images.

- Please provide a "standfirst text" summarizing the study in one or two sentences (approximately 250 characters, including space), three to four "bullet points" highlighting the main findings and a "synopsis image" (550px width and 400-600 px height, PNG format) to highlight the paper on our homepage.

Please refer to published papers for examples.

When you resubmit your manuscript, please download our CHECKLIST (https://media.springernature.com/original/springer-cms/rest/v1/content/27825796/data/v1) and include the completed form in your submission.
Please note that the Author Checklist will be published alongside the paper as part of the transparent process .

If you feel you can satisfactorily deal with these points and those listed by the referees, you may wish to submit a revised version of your manuscript. Please attach a covering letter giving details of the way in which you have handled each of the points raised by the referees. A revised manuscript will be once again subject to review and you probably understand that we can give you no guarantee at this stage that the eventual outcome will be favorable.

I look forward to receiving the revised manuscript soon.

Kind regards,
Jingyi

Jingyi Hou, PhD
Senior Editor
Molecular Systems Biology

We realize that it is difficult to revise to a specific deadline. In the interest of protecting the conceptual advance provided by the work, we recommend a revision within 3 months (5th Mar 2026). Please discuss the revision progress ahead of this time with the editor if you require more time to complete the revisions.

IMPORTANT: Please read our guidelines for revised submissions: https://link.springer.com/journal/44320/submission-guidelines#cms-Revised-submissions

*** PLEASE NOTE *** As part of the EMBO Press transparent editorial process initiative (see our Editorial at https://dx.doi.org/10.1038/msb.2010.72), Molecular Systems Biology publishes online a Review Process File with each accepted manuscripts. This file will be published in conjunction with your paper and will include the anonymous referee reports, your point-by-point response and all pertinent correspondence relating to the manuscript. If you do NOT want this File to be published, please inform the editorial office at contact@molsystbiol.org within 14 days upon receipt of the present letter.

Reviewer #1:

This is an ambitious and technically sophisticated study that aims to establish a systematic, cross-species platform that dissects the molecular and phenotypic trajectories of tumorigenesis through ten genetically defined RasV12-driven Drosophila models. Through longitudinal phenotypic analysis, the authors identified three consistent stages of tumor development-initiation, promotion, and progression-characterized by progressive changes in tumor size and tumor-induced wasting. The authors also revealed conserved cooperative mechanisms among JNK, Notch, NF-κB, and Hippo signaling pathways from Drosophila Ras tumors to human KRAS/NRAS cancers, establishing a systematic framework for cross-species translational cancer research.

While the central conceptual advance remains somewhat unclear and the mechanistic depth and translational relevance are limited, the study is underpinned by an impressive volume of data from integrative multi-omics and machine learning analyses, and the results broadly support the conclusions. I have a few comments that if addressed could improve the impact of the paper:

Major points
The authors propose that ART-D represents a transformative resource linking Drosophila genetics with human cancer biology, providing a systematic framework to uncover conserved oncogenic mechanisms and to enable precise targeting of stage-specific vulnerabilities in RAS-driven cancers. However, the current dataset lacks sufficient depth to justify the term 'resource'. I strongly recommend incorporating data on key metabolic and physiological parameters commonly assessed in Drosophila cancer

models, including trehalose, glucose, glycogen, TAG, ATP levels, muscle mass, bloating phenotype, food intake, and developmental timing.

In Figures 2A, 2B, 4C, 4D, 5E, and 5F, the authors should quantify the immunostaining signals and present the data in graphs.

In Figure 5C, the authors should quantify the eye area and include the results in a graph.Minor points

Minor points:
The authors should explain in the manuscript why they chose the three specific time points (5.5 AEL, 9.5 AEL, and 13.5 AEL).

In Figure 1B (13.5 AEL), although the quantified GFP+ region size in Rasv12, msn-/- tumors shows no significant difference compared with other tumor-bearing flies, the GFP fluorescence in the representative image appears substantially weaker. This discrepancy could be misleading; I therefore recommend replacing it with a more representative image.

The images in Figure 6B and 6E are difficult to see clearly; please increase their resolution for better visibility.

Reviewer #2:

In the manuscript by Guo et al, the authors provided a comparative study of various fly and human tumor driven by oncogenic Ras. This study provided interesting insights into the evolutionarily conserved tumorigenesis trajectory driven by oncogenic Ras. While the manuscript is clearly valuable for the general cancer biology community, I find the data analysis unnecessarily bulky. Overall, I would recommend the authors streamline the manuscript before publication.

My specific comments are:

1. Figure 1C/D, the authors seemed to classify these mutants into two groups. It is difficult to read from current plotting, The plots may benefit from clearly ordering these mutants into two groups to highlight the claimed trends. If all ten tumor models are similar, that is also fine.

2. I find it is somewhat vague to use transparence area/ratio as an indicator for cachexia. I think the manuscript can benefit from clear cachexia definitions and assays.

3. For Figure 2C-E, PCA and K-means clustering are methods to visualize high dimensional data. With only three parameters (intercept, slope, adjust R2), the authors can easily visualize these parameters in a 3-D plot. Phenotypically, they measured tumor sizes at 3 time points and the invasion index. Various transformations do not change the information available for classification of the ten tumor models. It is much better to directly draw their interpretations from Figure S3 or Figure 1.

4. It wasn't obvious from Line 142 what transcriptomics datasets that the authors have generated. They can simply state that they generated transcriptomics datasets from different tumor models at different time points without confusing readers with pseudotime points. This would be an appropriate place to visualize their ten tumor models across time in a PCA plot.

5. It was not clear to me why the authors pick the Fry tumors to perform multi-omics analysis in Figure 5.

6. For Figure 6F, how do the authors classify the expression levels of these proteins as high and low?

7. I find Figure 8 existing only for trendy purpose and would recommend removing it to avoid overstatement. One can extract predictive gene signatures in TCGA datasets easily and there is no benchmark to say that DRTs are better.

Response to reviewer's comments (*MS# MSB-2025-13410*):

Dear Editor and Reviewers,

We sincerely thank you for your constructive feedback and insightful comments on our manuscript. We are particularly grateful for the opportunity to submit a revised version for further evaluation. In response to the concerns raised, we have conducted additional experiments and thoroughly revised the manuscript. Below, we provide a point-by-point response to each comment. For your convenience, all changes in the revised text are **highlighted in red**.

We hope that these revisions adequately address your concerns and that the manuscript is now suitable for publication.

Reviewer #1:

This is an ambitious and technically sophisticated study that aims to establish a systematic, cross-species platform that dissects the molecular and phenotypic trajectories of tumorigenesis through ten genetically defined RasV12-driven Drosophila models. Through longitudinal phenotypic analysis, the authors identified three consistent stages of tumor development-initiation, promotion, and progression-characterized by progressive changes in tumor size and tumor-induced wasting. The authors also revealed conserved cooperative mechanisms among JNK, Notch, NF-κB, and Hippo signaling pathways from Drosophila Ras tumors to human KRAS/NRAS cancers, establishing a systematic framework for cross-species translational cancer research.

While the central conceptual advance remains somewhat unclear and the mechanistic depth and translational relevance are limited, the study is underpinned by an impressive volume of data from integrative multi-omics and machine learning analyses, and the results broadly support the conclusions. I have a few comments that if addressed could improve the impact of the paper:

Response: Thank you for your encouraging assessment and recognition of our systematic framework for cross-species translational research of our study. We have addressed your concerns regarding conceptual clarity, mechanistic depth, and translational relevance through additional analyses and textual revisions, which we believe have significantly strengthened our findings. Below, we provide our point-by-point responses.

Major points

The authors propose that ART-D represents a transformative resource linking Drosophila genetics with human cancer biology, providing a systematic framework to uncover conserved oncogenic mechanisms and to enable precise targeting of stage-specific vulnerabilities in RAS-driven cancers. However, the current dataset lacks sufficient depth to justify the term 'resource'. I strongly recommend incorporating data on key metabolic and physiological parameters commonly assessed in Drosophila cancer models, including trehalose, glucose, glycogen, TAG, ATP levels, muscle mass, bloating phenotype, food intake, and developmental timing.

Response: Thank you for this insightful suggestion. We fully agree that incorporating key metabolic and physiological parameters—such as trehalose, glucose, glycogen, TAG, ATP levels,

muscle mass, bloating phenotype, food intake, and developmental timing**—**is critical for defining a comprehensive cancer model resource. Our current ART-D dataset already reveals substantial disparities in biological processes associated with glucose metabolism, glycogen synthesis, and fatty acid metabolism across different *Ras* tumor subtypes (see **Figs. 3B, 3C, and EV2–EV4**). These findings highlight the complex metabolic reprogramming inherent in these models and underscore the necessity for further untargeted lipidomics to precisely quantify the role of specific metabolites in sustaining malignant progression.

Moreover, we acknowledge the importance of systemic physiological changes, such as tumor-mediated cachexia driven by muscle and fat body degradation. While previous studies have identified key cachexia factors regulating tumor metabolism (e.g., Lee et al., *PNAS* 2021; Lodge et al., *Dev. Cell* 2021; Liu et al., *DMM* 2022), a systematic integration of these phenotypes with multi-omics data remains an emerging frontier. In this context, we position ART-D as a foundational **catalyst** and **systematic framework** designed to guide such in-depth explorations. We are currently actively expanding this work to investigate the interplay between malignant tumors and distant organs (muscle and fat body) in both larval and adult stages (unpublished). We believe that future supplementary studies integrating lipidomics and epigenomics will further solidify ART-D's status as a complete "resource".

Regarding the current manuscript, while large-scale corresponding omics data for human samples remain incomplete, we have added a comprehensive discussion on the research significance of these metabolic and physiological dimensions. We have revised the text to explicitly frame ART-D as a starting point for these critical investigations rather than a final exhaustive catalog. Please refer to the revised manuscript on **page 9, lines 188–200**, and **page 21, lines 538–545**, **Figs. 3B, 3C, and EV2E, EV3E, EV3E**.

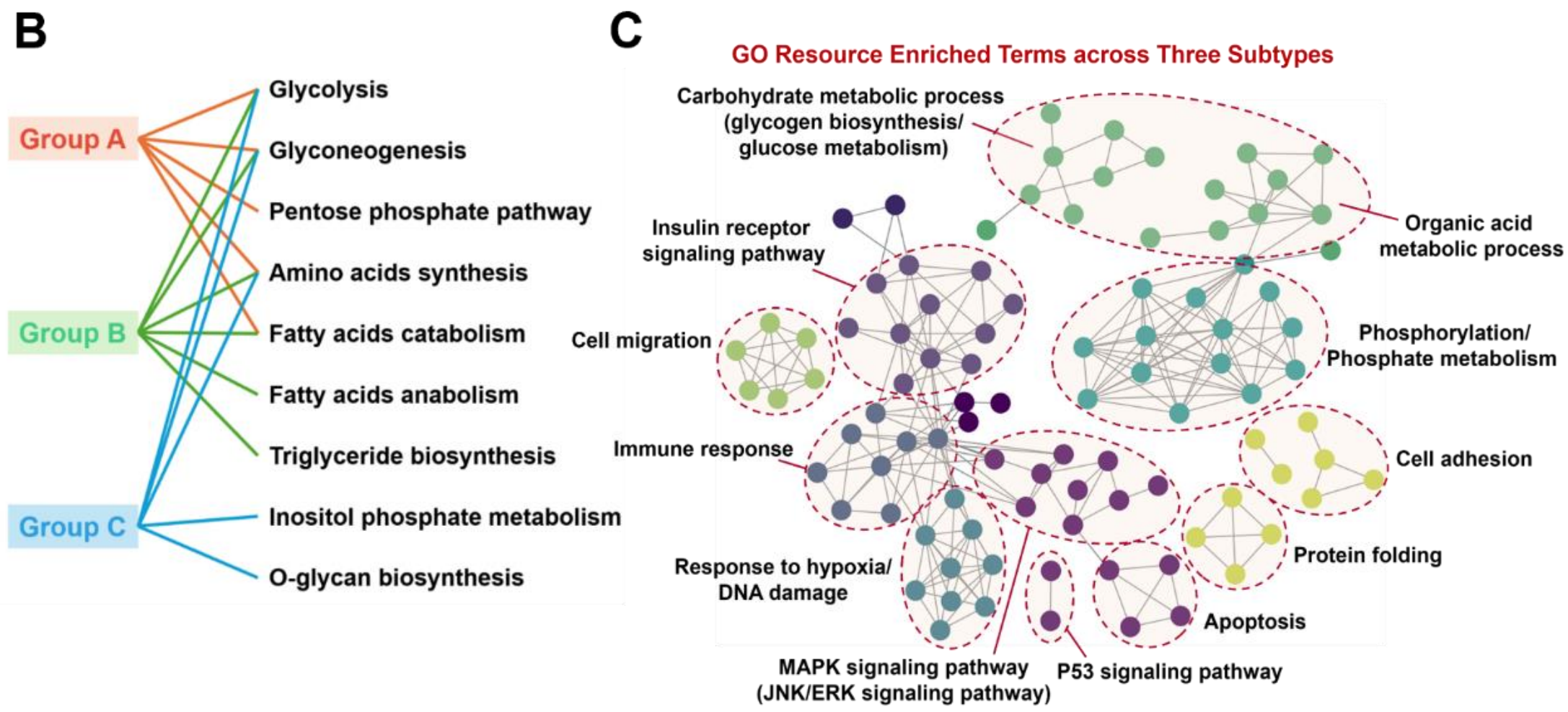

Figure 3. Dynamic transcriptomic profiles of different subtypes of *Drosophila Ras*-driven tumors.

(B) A summary of top-ranking terms enriched across different *Drosophila* databases of three subtypes of *Drosophila Ras*-driven tumors.

(C) A gene regulatory network constructed based on GO terms that were significantly enriched. By intersecting GO terms and DEGs enriched in the three subtypes, the corresponding gene regulatory networks were constructed to explore the common biological changes among the three

subtypes of tumor groups.

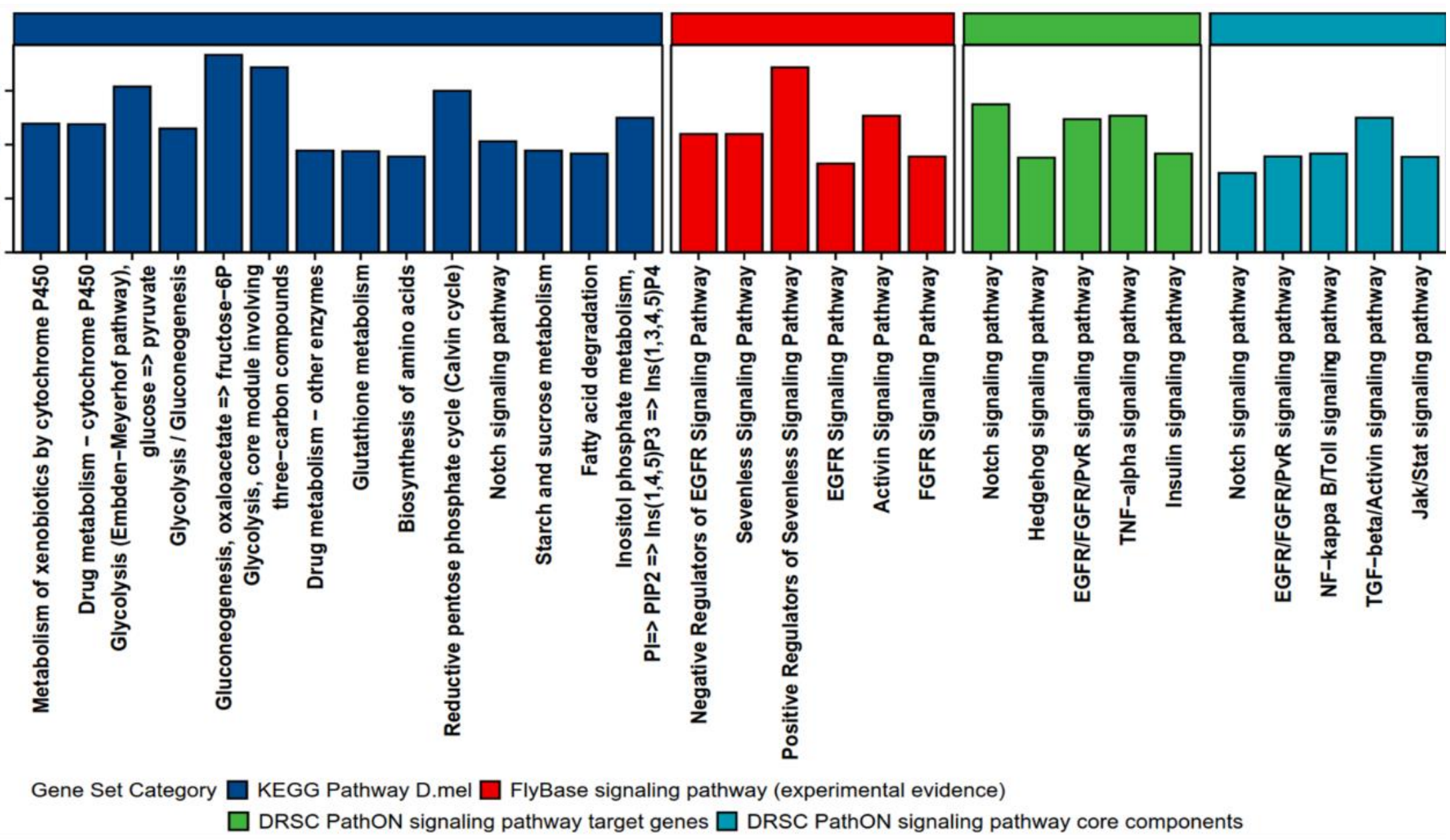

Figure EV2. Dynamic transcriptomic profiles of Group A *Drosophila Ras*-driven tumors.

(E) The bar plot depicting the functional annotations of candidate genes obtained from certain clusters (D), revealed by PANGEA.

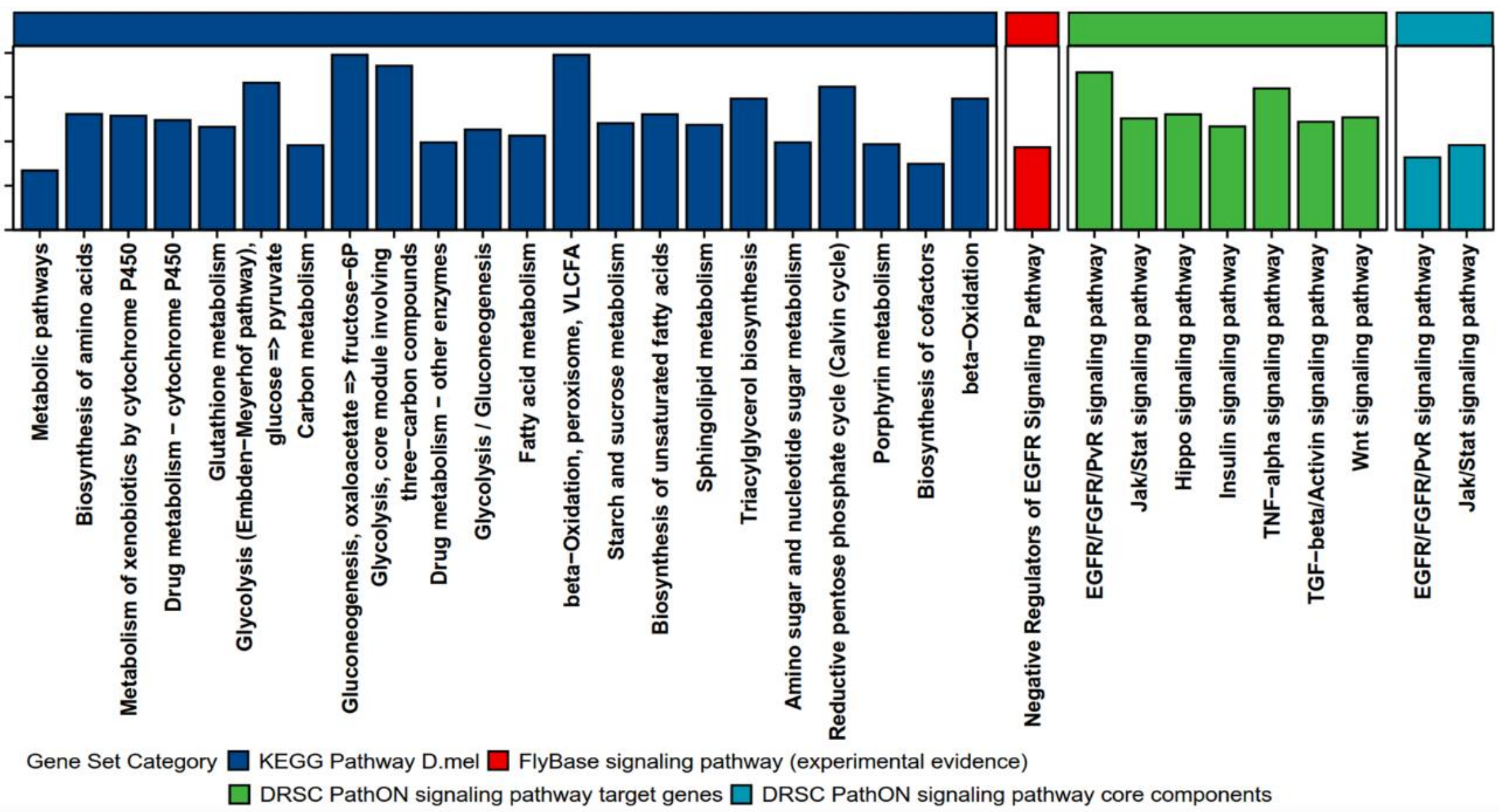

Figure EV3. Dynamic transcriptomic profiles of Group B *Drosophila Ras*-driven tumors.

(E) The bar plot depicting the functional annotations of candidate genes obtained from certain clusters (D), revealed by PANGEA.

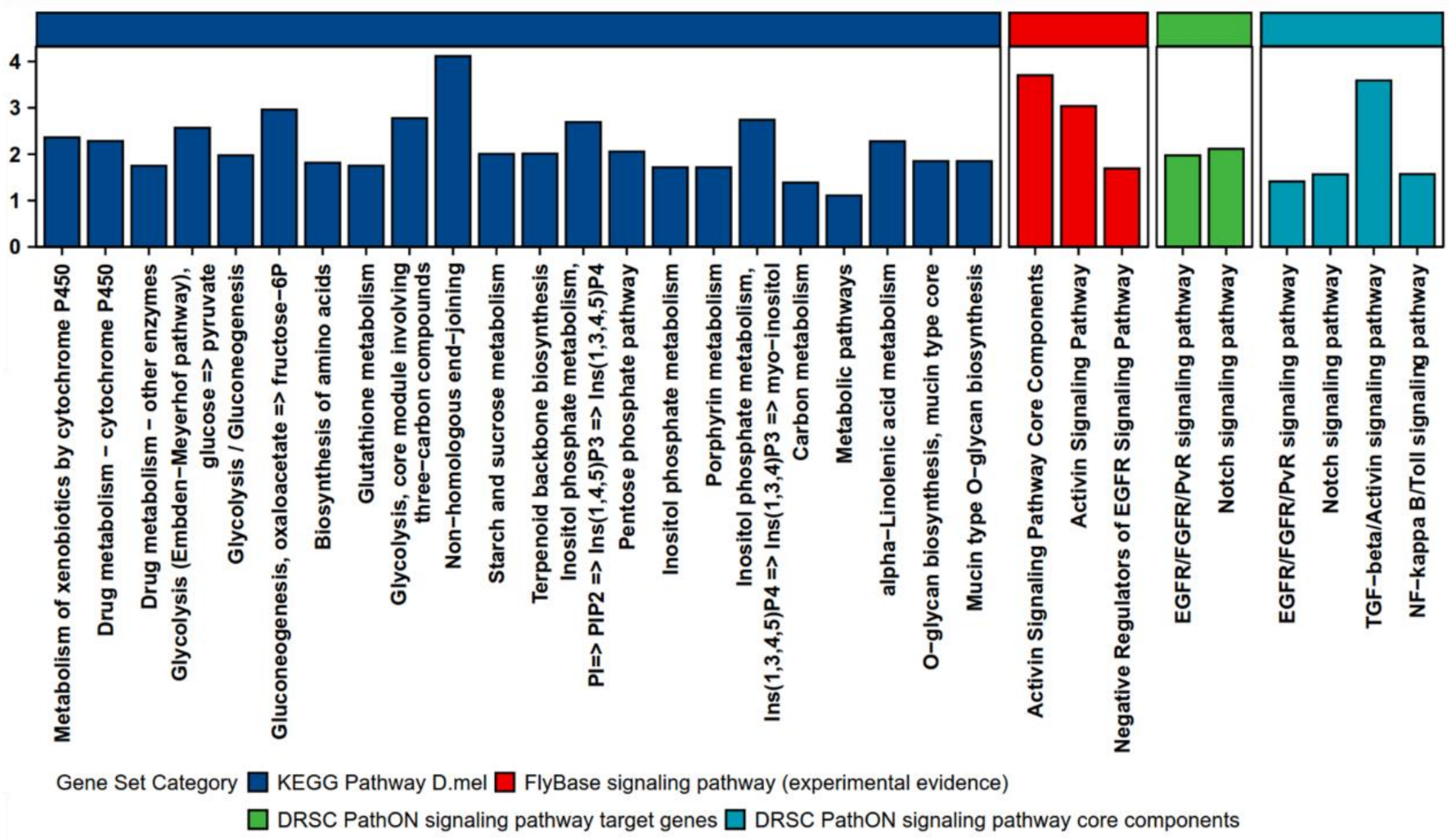

Figure EV4. Dynamic transcriptomic profiles of Group C *Drosophila Ras*-driven tumors.

(E) The bar plot depicting the functional annotations of candidate genes obtained from certain clusters (D), revealed by PANGEA.

In Figures 2A, 2B, 4C, 4D, 5E, and 5F, the authors should quantify the immunostaining signals and present the data in graphs.

Response: In the revision, we have now quantified the immunostaining signals for the indicated figures, as well as relevant appendix figures, and included statistical analyses to support our conclusions. These new quantitative data are presented in **Figs. 2A', 2B', 5F, and 5G**, and **Appendix Figs. S5A'–S5D', S6A', S6B', S7C, S7D, S8A'–S8C', S10E**, and the revised description on **page 14, lines 335–354**.

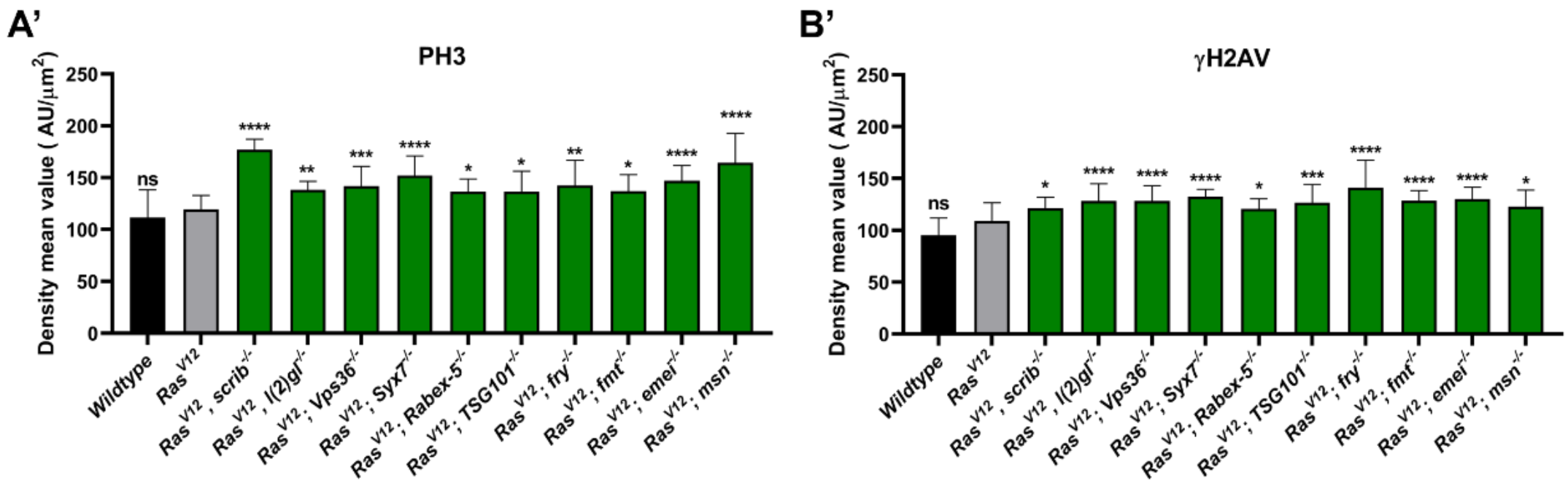

Figure 2. The classifications of *Drosophila Ras*-driven tumors.

(A'-B') Quantification results of immunofluorescence imaging of PH3 (A) and γH2AV (B). Kruskal-Wallis test was performed along with Dunn's multiple comparisons test against the *Ras* group, n >20. Results were presented as means ± SD. Significance of different genotypes were denoted as follows: ns > 0.05, *P < 0.05, **P < 0.01, ***P < 0.001, ****P < 0.0001.

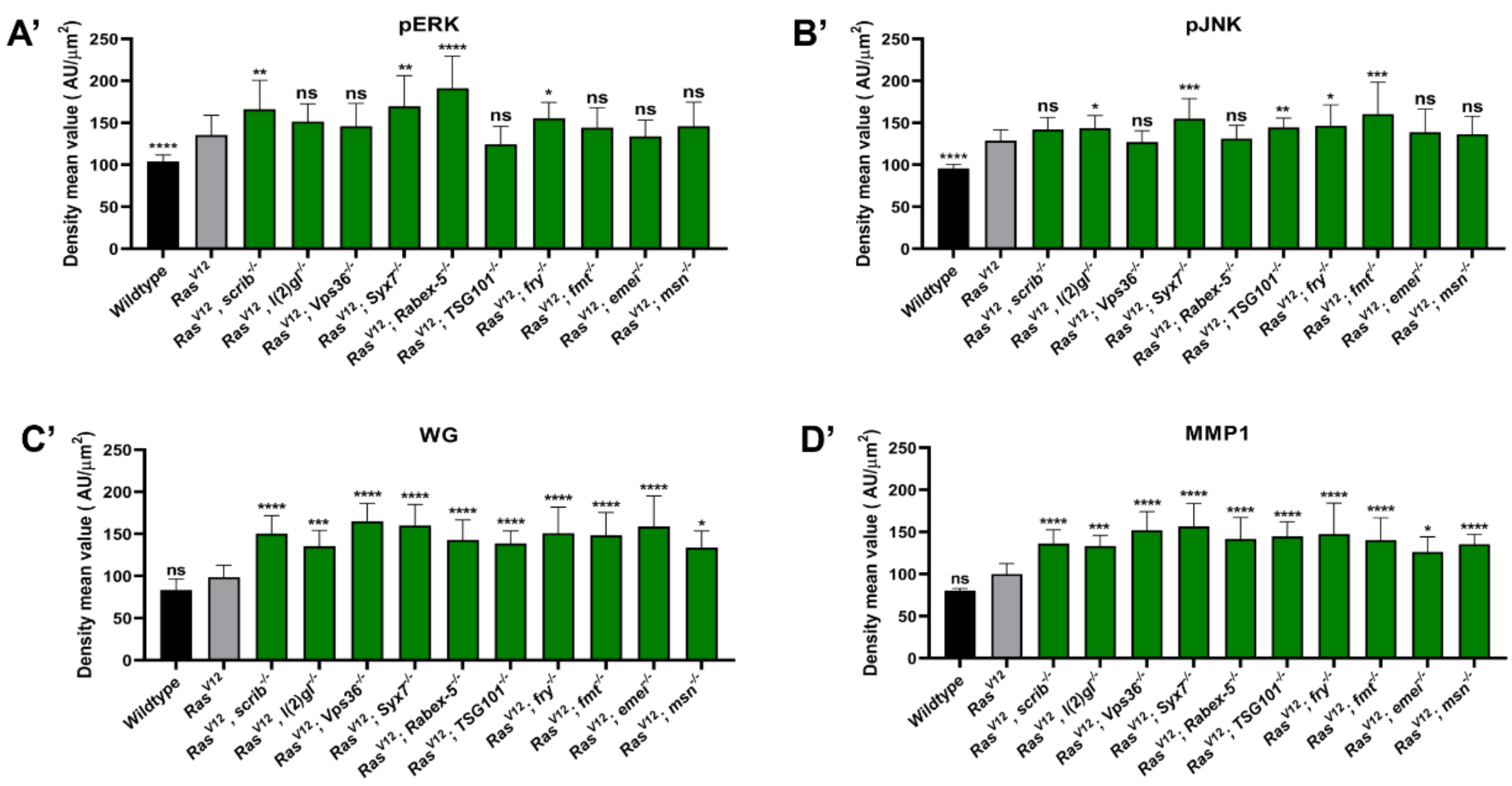

Appendix figure S5. The activation of MAPK signaling pathways at the initial stage of *Drosophila Ras*-driven tumors.

(A'-D') Quantification results of immunofluorescence imaging of pERK (A'), pJNK (B'), Wg (C'), and MMP1 (D'). Kruskal-Wallis test was performed along with Dunn's multiple comparisons test against the *Ras* group, n >20. Results were presented as means ± SD. Significance of different genotypes were denoted as follows: ns > 0.05, **P* < 0.05, ***P* < 0.01, ****P* < 0.001, *****P* < 0.0001.

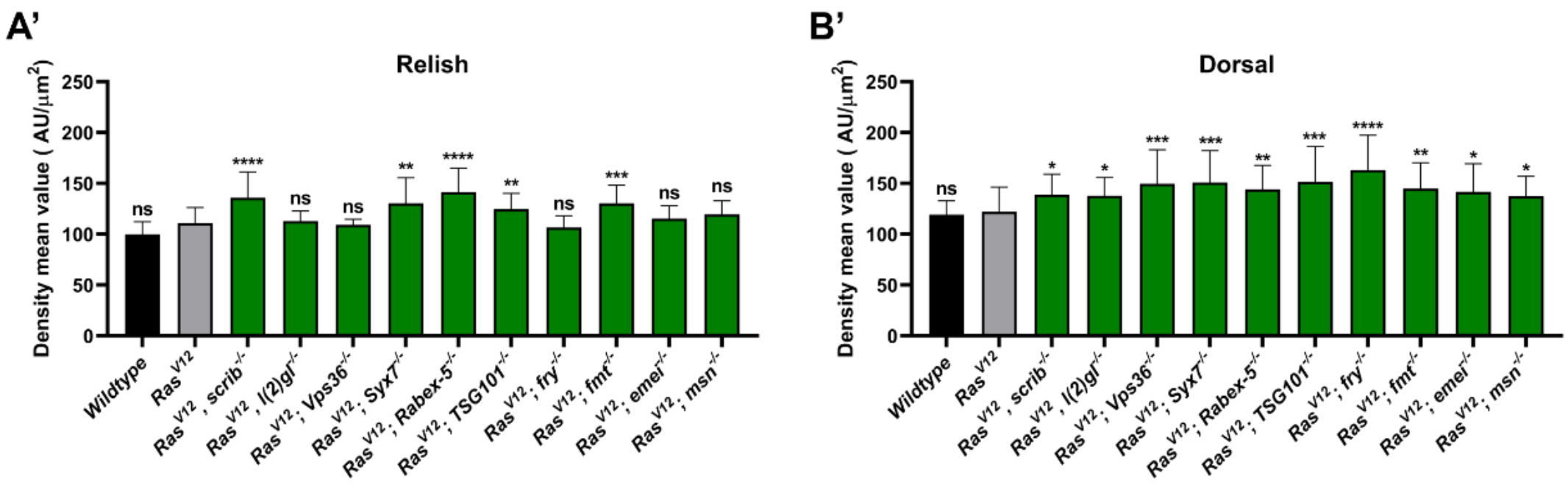

Appendix figure S6. The activation of NFκB/Imd and NFκB/Toll signaling pathways at the initial stage of *Drosophila Ras*-driven tumors.

(A'-B') Quantification results of immunofluorescence imaging of Relish (A') and Dorsal (B'). Kruskal-Wallis test was performed along with Dunn's multiple comparisons test against the *Ras* group, n >20. Results were presented as means ± SD. Significance of different genotypes were denoted as follows: ns > 0.05, **P* < 0.05, ***P* < 0.01, ****P* < 0.001, *****P* < 0.0001.

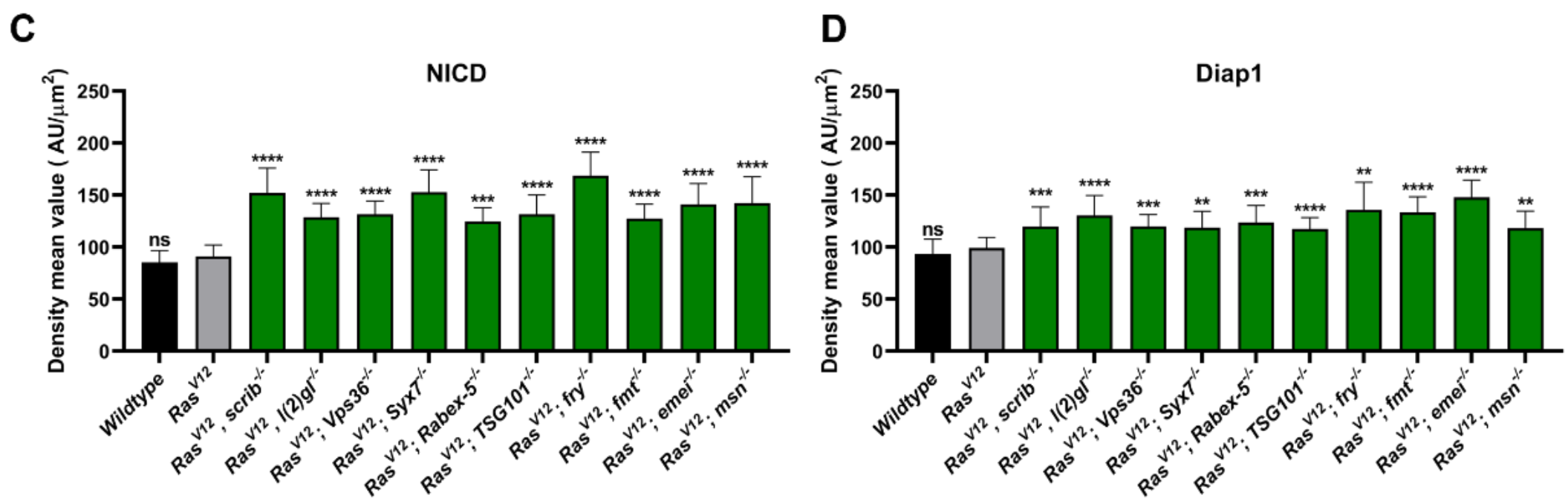

Appendix figure S7. The Notch activation and Hippo inactivation across different *Drosophila Ras*-driven tumors.

(C-D) Quantification results of immunofluorescence imaging of NICD (C) and Diap1 (D). Kruskal-Wallis test was performed along with Dunn's multiple comparisons test against the *Ras* group, n >20. Results were presented as means ± SD. Significance of different genotypes were denoted as follows: ns > 0.05, **P* < 0.05, ***P* < 0.01, ****P* < 0.001, *****P* < 0.0001.

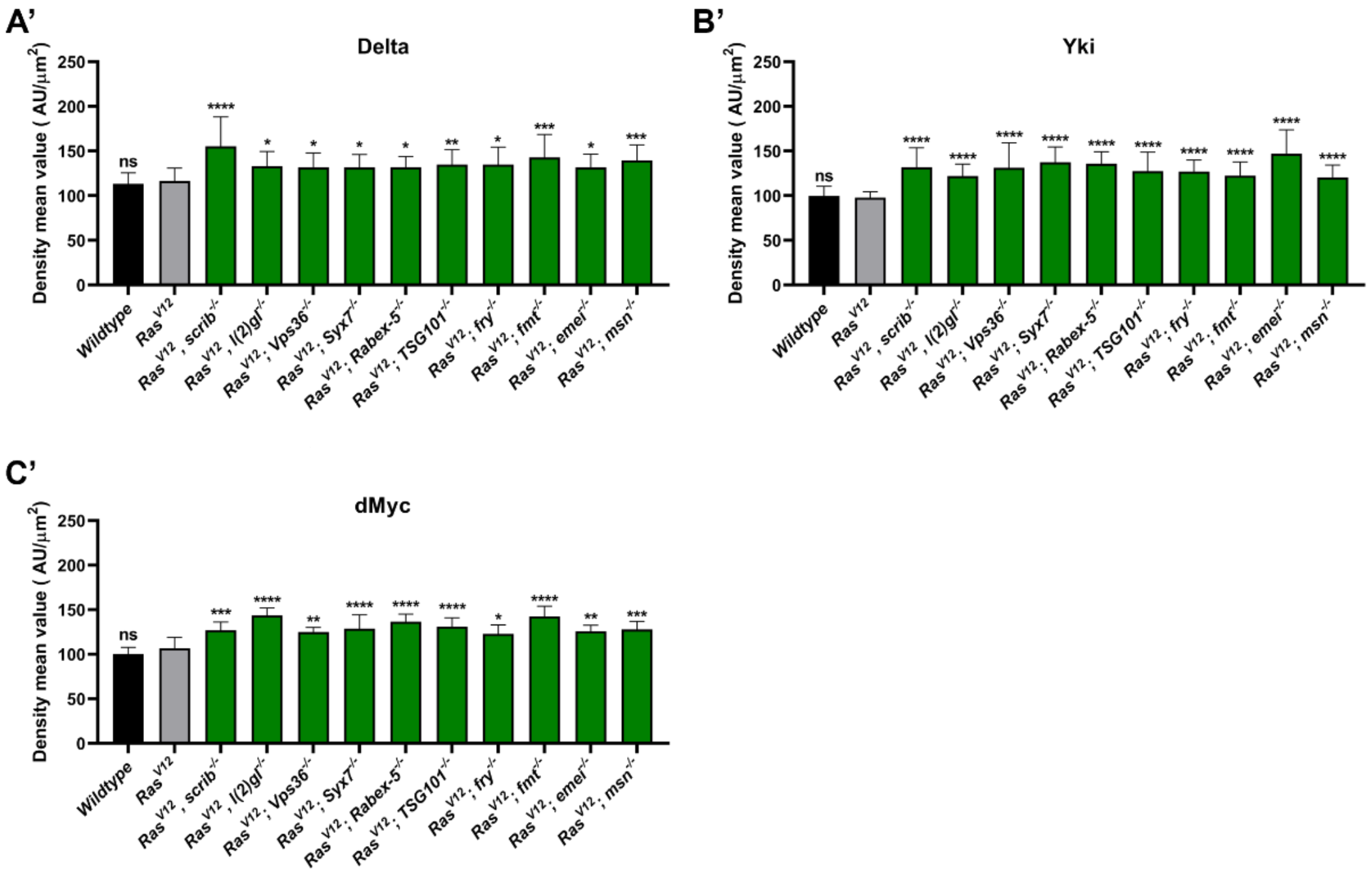

Appendix figure S8. The tumor promoting roles of Notch activation and Hippo inactivation across different *Drosophila Ras*-driven tumors.

(A'-C') Quantification results of immunofluorescence imaging of Delta (A'), Yki (B') and dMyc (C'). Kruskal-Wallis test was performed along with Dunn's multiple comparisons test against the *Ras* group, n >20. Results were presented as means ± SD. Significance of different genotypes were denoted as follows: ns > 0.05, **P* < 0.05, ***P* < 0.01, ****P* < 0.001, *****P* < 0.0001.

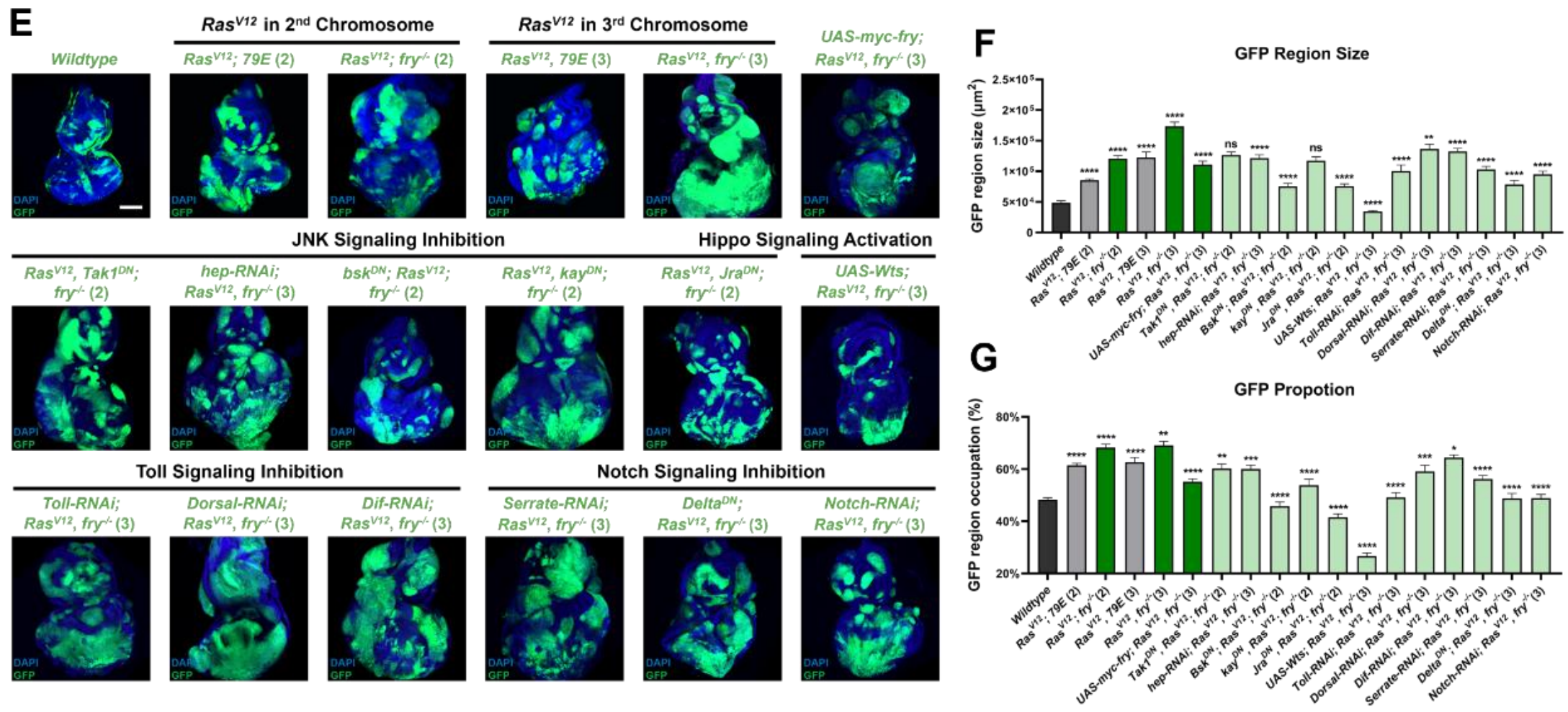

Figure 5. Multi-omics data analysis of *Ras^{V12}; fry$^{-/-}$* tumors across different stages.

(E) The confocal images of JNK signaling inhibition, Hippo signaling inactivation, NF-κB/Toll signaling inhibition, and Notch signaling inhibition in *Ras*-driven *fry*-deficient tumors. To ensure precise data quantification, we generated *fry*-deficient tumors expressing *Ras^{V12}* on either the second (2nd) or third (3rd) chromosome of *Drosophila*, designated as *Ras^{V12}; fry$^{-/-}$* (2) and *Ras^{V12}, fry$^{-/-}$* (3), respectively, with the chromosomal location of *Ras^{V12}* indicated in parentheses. This nomenclature was applied consistently to both experimental and control groups, followed by statistical analysis of the corresponding tumor samples. Samples were observed at 5.5-6.5 days AEL. Scale bar: 100 µm.

(F) Quantification of the GFP region size within eye-antennal disc across different genotypes in Fig. 5E. Statistical significance was assessed using either the Mann–Whitney U-test or Student's t-test, depending on variance homogeneity (n > 25). Experimental groups were compared against the corresponding *Ras* control group. Data are presented as means ± SEM. Significance levels are denoted as follows: ns > 0.05, *P < 0.05, **P < 0.01, ***P < 0.001, ****P < 0.0001.

(G) Quantification of GFP region occupation within eye-antennal disc across different genotypes in Fig. 5E. Statistical significance was assessed using either the Mann–Whitney U-test or Student's t-test, depending on variance homogeneity (n > 25). Experimental groups were compared against the corresponding *Ras* control group. Data are presented as means ± SEM. Significance levels are denoted as follows: ns > 0.05, *P < 0.05, **P < 0.01, ***P < 0.001, ****P < 0.0001.

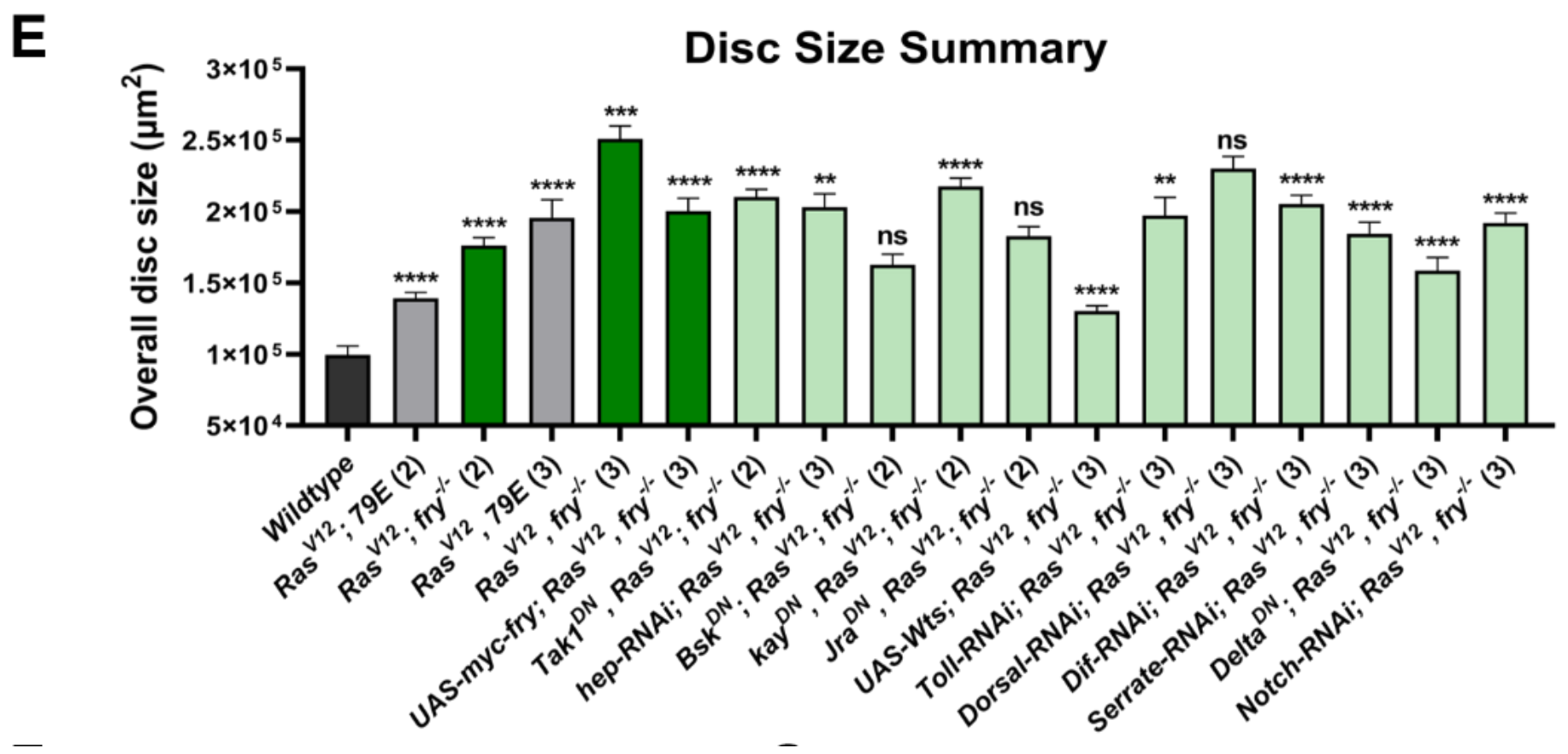

Appendix figure S10. Evaluation of physiological and pathological functions of *fry*.

(E) Quantification of overall eye-antennal disc size across different genotypes. Statistical significance was assessed using either the Mann–Whitney U-test or Student's t-test, depending on variance homogeneity (n > 25). Experimental groups were compared against the corresponding Ras control group. Data are presented as means ± SEM. Significance levels are denoted as follows: ns > 0.05, **P* < 0.05, ***P* < 0.01, ****P* < 0.001, *****P* < 0.0001.

In Figure 5C, the authors should quantify the eye area and include the results in a graph.

Response: We appreciate this recommendation. In the revised manuscript, we have quantified the adult eye areas for both male and female flies separately and presented the results graphically. These data can be found in **Figs. 5C and 5C', Appendix Figs. S10D and S10D'**, and the updated description on **page 14, lines 326–332**.

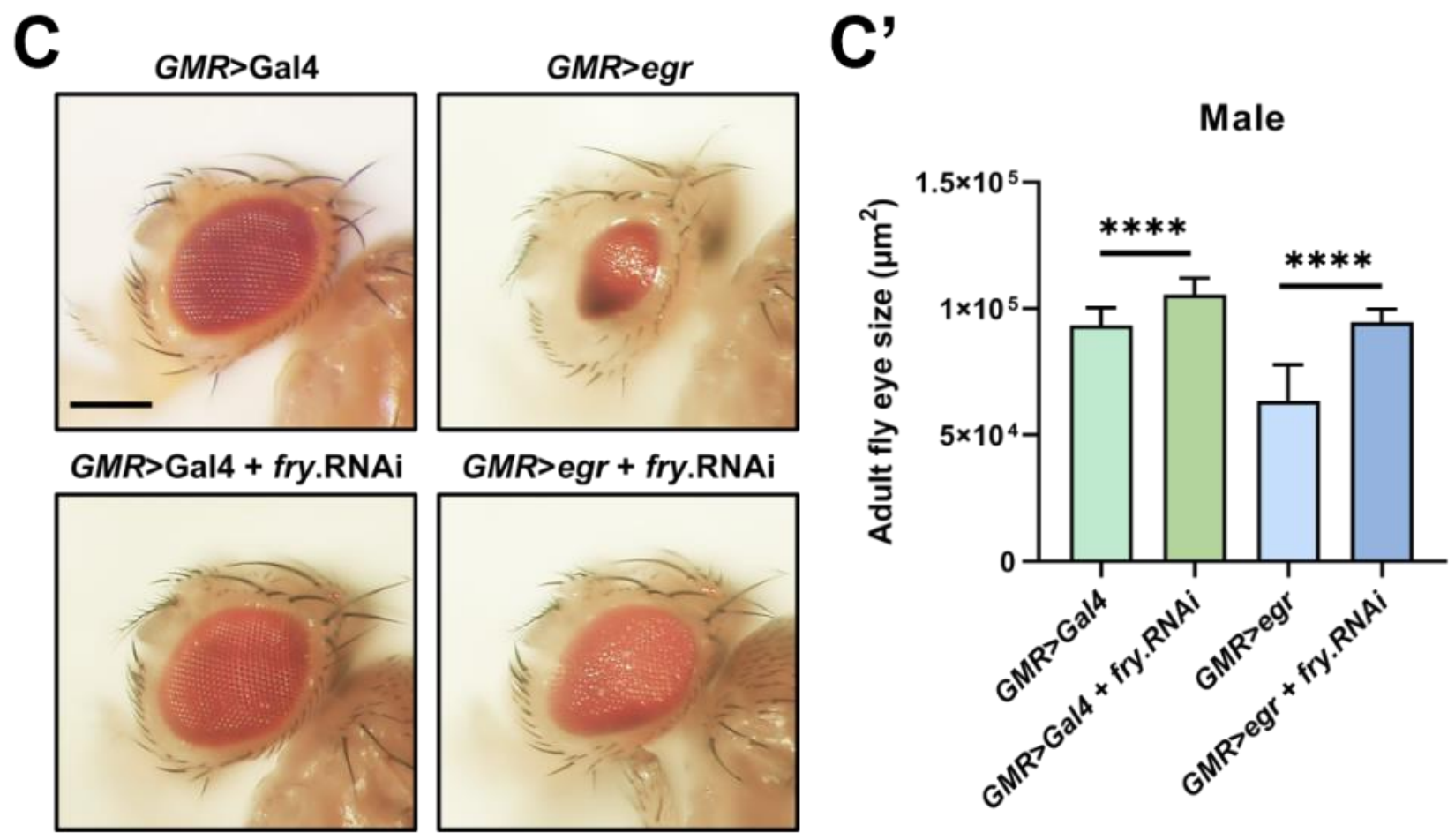

Figure 5. Multi-omics data analysis of *Ras^{V12}; fry$^{-/-}$* tumors across different stages.

(C-C') The light micrographs (C) and quantification results (C') of adult eyes (Male) with the indicated genotypes. Scale bars: 200 μm. Statistical analysis for each group was performed using the Mann-Whitney U-test or Student's t-test based on the variance. Results were presented as means ± SD, n >25. Significance of different genotypes were denoted as follows: ns > 0.05, **P* < 0.05, ***P* < 0.01, ****P* < 0.001, *****P* < 0.0001.

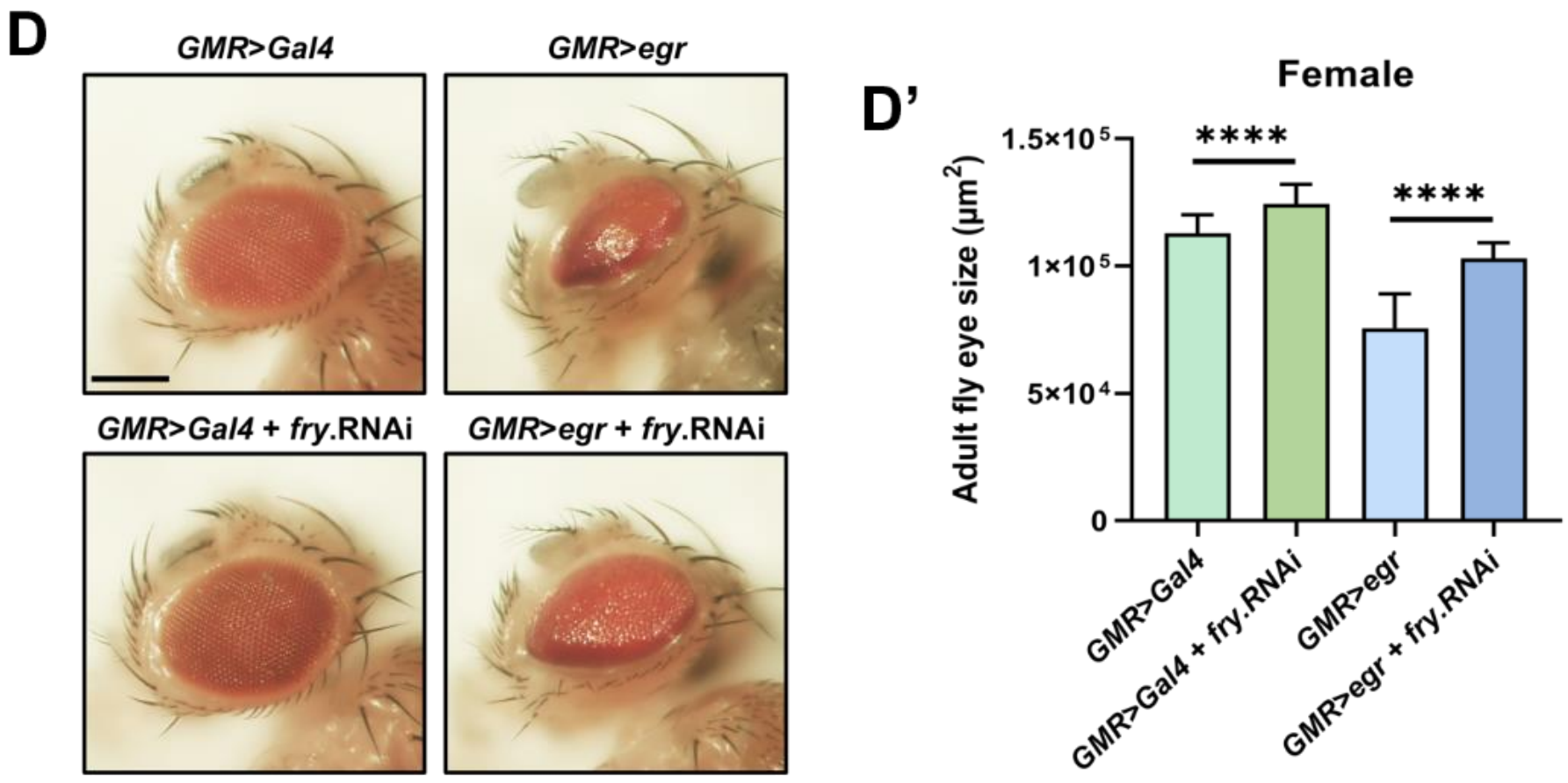

Appendix figure S10. Evaluation of physiological and pathological functions of *fry*.

(D-D') The light micrographs (D) and quantification results (D') of adult eyes (Female) with the indicated genotypes. Scale bars: 200 μm. Statistical analysis for each group was performed using the Mann-Whitney U-test or Student's t-test based on the variance. Results were presented as means ± SD, n >25. Significance of different genotypes were denoted as follows: ns > 0.05, **P* < 0.05, ***P* < 0.01, ****P* < 0.001, *****P* < 0.0001.

Minor points:

The authors should explain in the manuscript why they chose the three specific time points (5.5 AEL, 9.5 AEL, and 13.5 AEL).

Response: Thank you for this important query. The time points selected for ART-D were determined based on the following rationale. **1) 5.5 days AEL**: This corresponds to the standard duration for wild-type larvae to reach pupariation. Assessing this time point allows us to effectively determine whether the Ras^{V12} + $X^{-/-}$ combination confers pupariation delay. **2) 13.5 days AEL**: This represents the upper limit for harvesting late-stage malignant tumors. Our preliminary data indicated that non-pupated larvae rarely survive or remain viable beyond this timeframe. **3) 9.5 days AEL**: This serves as a critical intermediate time point to capture tumor progression between the initial delay (5.5 days) and the terminal stage (13.5 days). We have added a detailed explanation of this rationale to the revised manuscript (**Page 5, Lines 71–76**).

In Figure 1B (13.5 AEL), although the quantified GFP+ region size in Rasv12, msn-/- tumors shows no significant difference compared with other tumor-bearing flies, the GFP fluorescence in the representative image appears substantially weaker. This discrepancy could be misleading; I therefore recommend replacing it with a more representative image.

Response: We appreciate your keen observation regarding the representative image in Figure 1B. Although we have replaced the image as recommended, we wish to clarify that the apparent difference in fluorescence intensity does not contradict our quantitative findings. As shown in the statistical analysis (**Figs. 1C and 1C'**), while the tumor size (GFP region size and ratio) of Ras^{V12}, $msn^{-/-}$ genotypes at the progression stage (13.5 days AEL) appears visually distinct, the difference

is **not statistically significant (“ns”) when compared to the promotion stage** (9.5 days AEL). The image selected for Figure 1B was chosen because it accurately reflects the average morphological characteristics and fluorescence distribution observed across the biological replicates for this specific genotype and time point, rather than an outlier with maximal intensity.

The images in Figure 6B and 6E are difficult to see clearly; please increase their resolution for better visibility.

Response: Thank you for pointing this out. We have replaced Figures 6B and 6E with high-resolution versions in the revised manuscript to ensure all details are clearly visible.

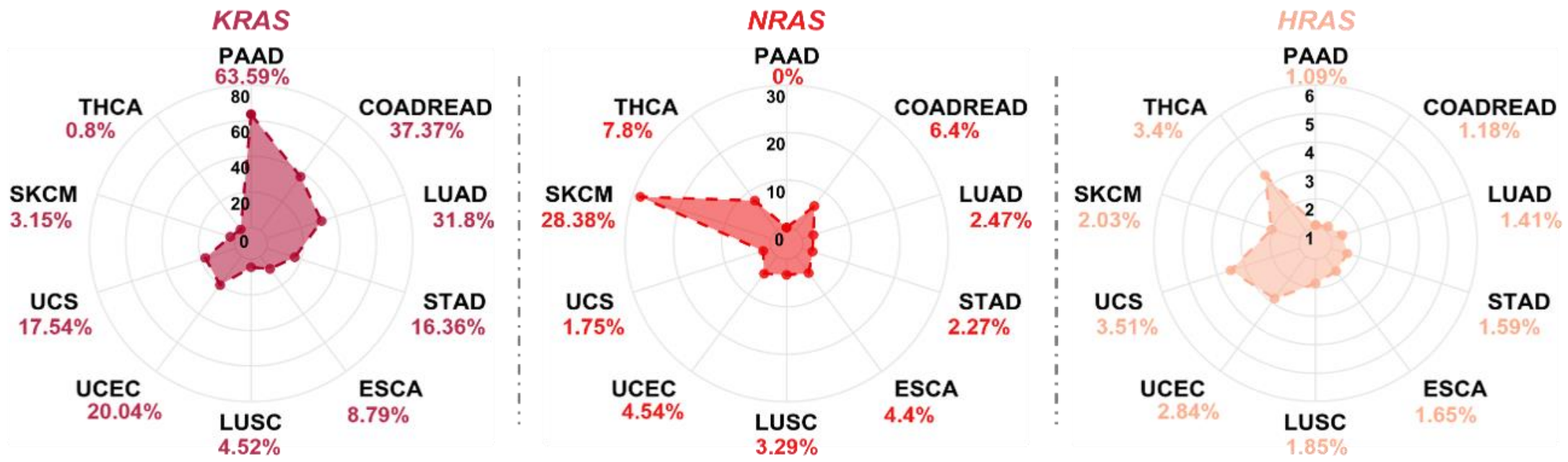

Figure 6. The pan-cancer evaluation of human orthologs in *KRAS*/*NRAS*-driven cancers

(B) The mutation rate of *RAS* family genes (*KRAS*, *NRAS*, *HRAS*) in ten cancers (PAAD, COADREAD, LUAD, STAD, ESCA, LUSC, UCEC, UCS, SKCM, THCA). Data was obtained from TCGA PanCancer Atlas Studies of cBioProtal.

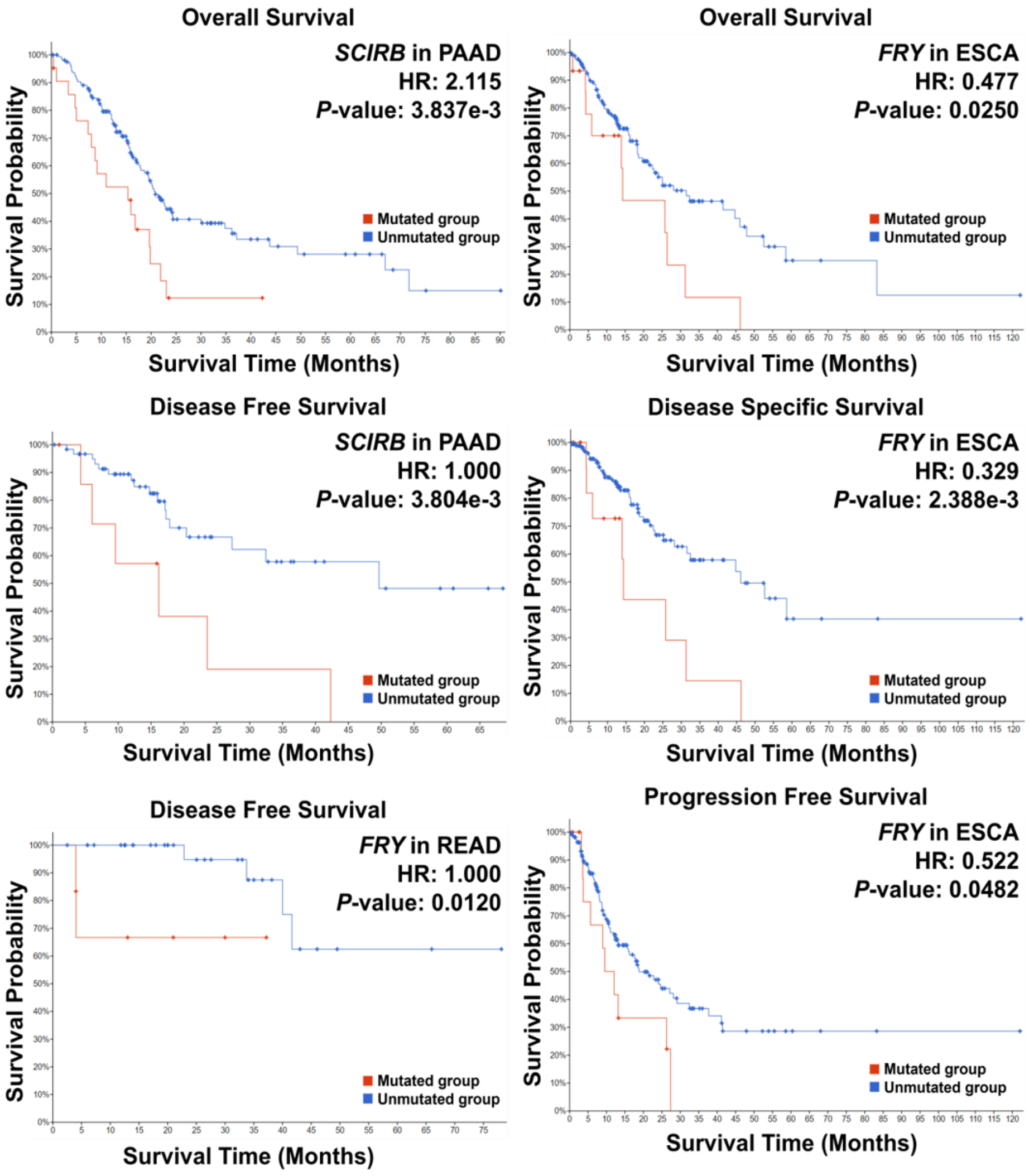

Figure 6. The pan-cancer evaluation of human orthologs in *KRAS*/*NRAS*-driven cancers

(E) Kaplan-Meier survival curves illustrating the impact of human ortholog mutation status on patient survival in specific cancers.

Reviewer #2:

In the manuscript by Guo et al, the authors provided a comparative study of various fly and human tumor driven by oncogenic Ras. This study provided interesting insights into the evolutionarily conserved tumorigenesis trajectory driven by oncogenic Ras. While the manuscript is clearly valuable for the general cancer biology community, I find the data analysis unnecessarily bulky. Overall, I would recommend the authors streamline the manuscript before publication.

Response: Thank you for recognizing the value of our comparative study and the insights into conserved tumorigenesis trajectories. We agree that the data analysis was overly dense. In this revision, we have streamlined the manuscript by moving detailed analytical procedures and related data to Appendix file, and refining the main text to focus on key biological findings, thereby improving clarity and readability without compromising scientific rigor.

My specific comments are:

1. Figure 1C/D, the authors seemed to classify these mutants into two groups. It is difficult to read from current plotting, The plots may benefit from clearly ordering these mutants into two groups to highlight the claimed trends. If all ten tumor models are similar, that is also fine.

Response: Thank you for this insightful suggestion. To address this, we have reorganized the mutants in Figures 1C and 1D based on their physiological functions: cell polarity regulators (*scrib*, *l(2)gl*), endocytosis regulators (*Vps36*, *Syx7*, *Rabex-5*, *TSG101*), and others (*fry*, *fmt*, *emei*, *msn*). This arrangement was intended to highlight potential trends among functionally related genes. However, our analysis revealed that progression patterns are not strictly uniform across functional groups. Specifically, only *scrib* and *l(2)gl* (polarity) and *Vps36*, *Syx7*, and *Rabex-5* (endocytosis) exhibited comparable progression patterns across both malignant indicators (Figs. 1C–1D'). Meanwhile, *fry*, *fmt*, and *emei* showed similar trends only in larval tumor burden (Figs. 1C, 1C'), while the remaining genes displayed distinct characteristics. Given this heterogeneity, we agree that visual grouping alone is insufficient. Consequently, we have performed Principal Component Analysis (PCA) and K-means clustering based on tumor malignant progression metrics to objectively delineate subtypes according to phenotypic similarities (Appendix Fig. S2D, Figs. 2C-2E). These new analyses are now included in the revised manuscript to better define the relationships between these mutants.

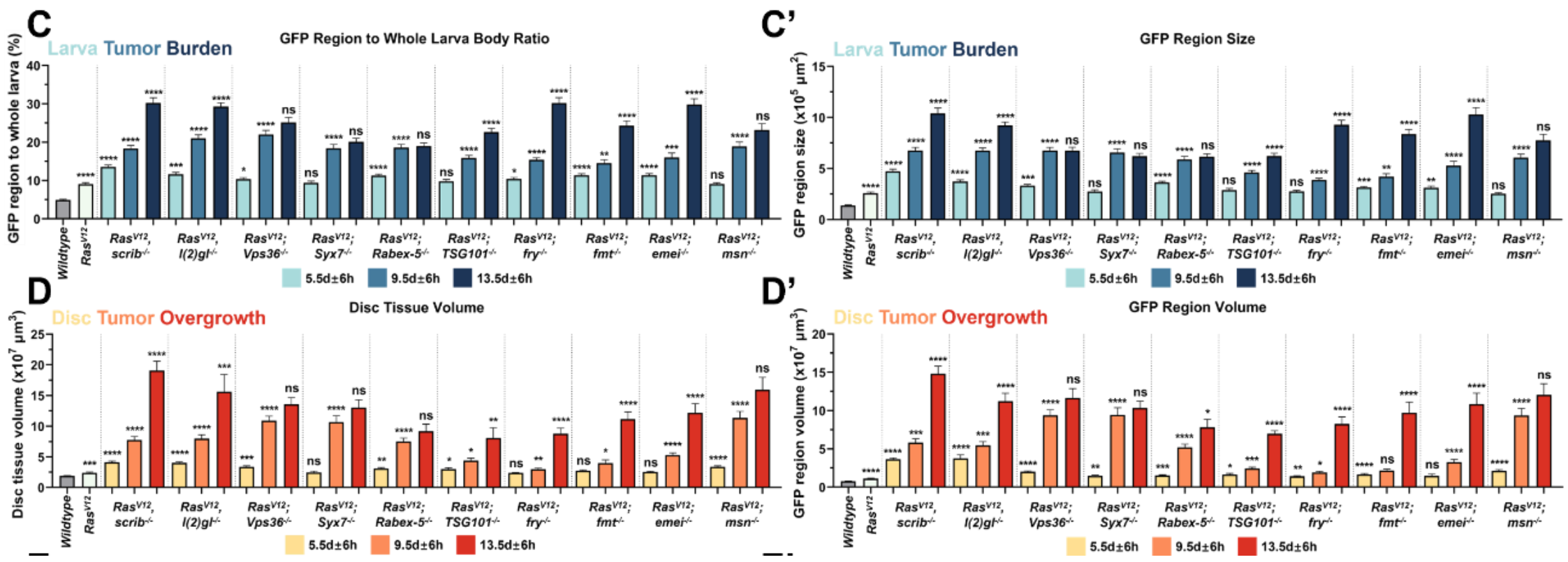

Figure 1. The Atlas of *Ras*-driven Tumors in *Drosophila* (ART-D).

(C-D') The quantifications of larval tumor burden, tumor-mediated cachexia, and tumor overgrowth in ART-D. The larval tumor burden was quantified by the GFP region size (C) and the ratio of GFP region to whole larva (C'); the tumor overgrowth was quantified by the overall disc tissue volume (D) and the GFP region volume (D'), respectively. Statistical analysis for each group was performed using the Mann-Whitney U-test and presented as mean ± SEM, n >20. ns > 0.05, *P < 0.05, **P < 0.01, ***P < 0.001, ****P < 0.0001, indicating the differences in larva tumor burden, tumor-mediated cachexia, or tumor overgrowth at the current stage compared to the previous stage, *Ras* group was compared with *wildtype* group, the initial stage tumors were compared with *Ras* group.

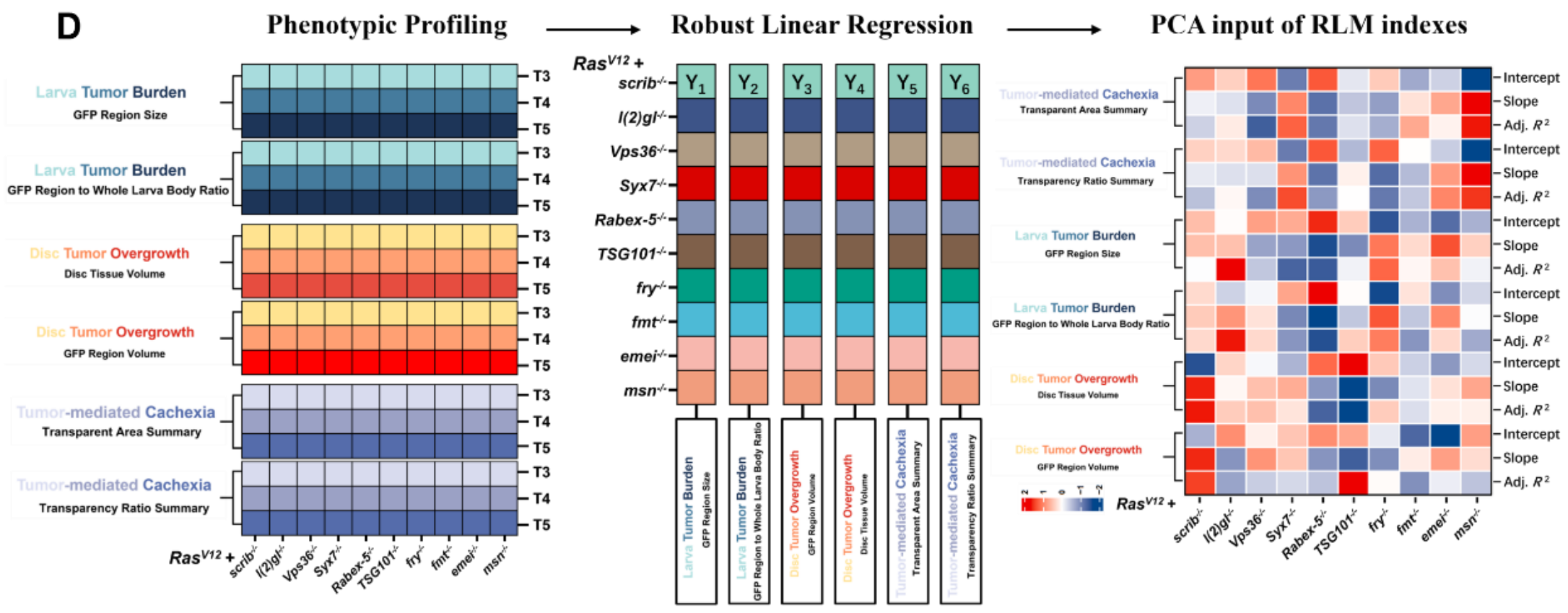

Appendix figure S2. The robust linear regression model of tumor malignancies in different *Drosophila Ras*-driven tumors.

(D) Schematic diagram illustrating principal component analysis (PCA) performed on robust regression models constructed from phenotypic data across various DRTs. Using the intercept, slope, and adjusted R-squared values of these models as the input matrix, the further PCA analysis was conducted in conjunction with K-means clustering. This approach facilitates the identification of subtypes based on malignant progression phenotypes while preserving a quantitative focus on phenotypic dynamics.

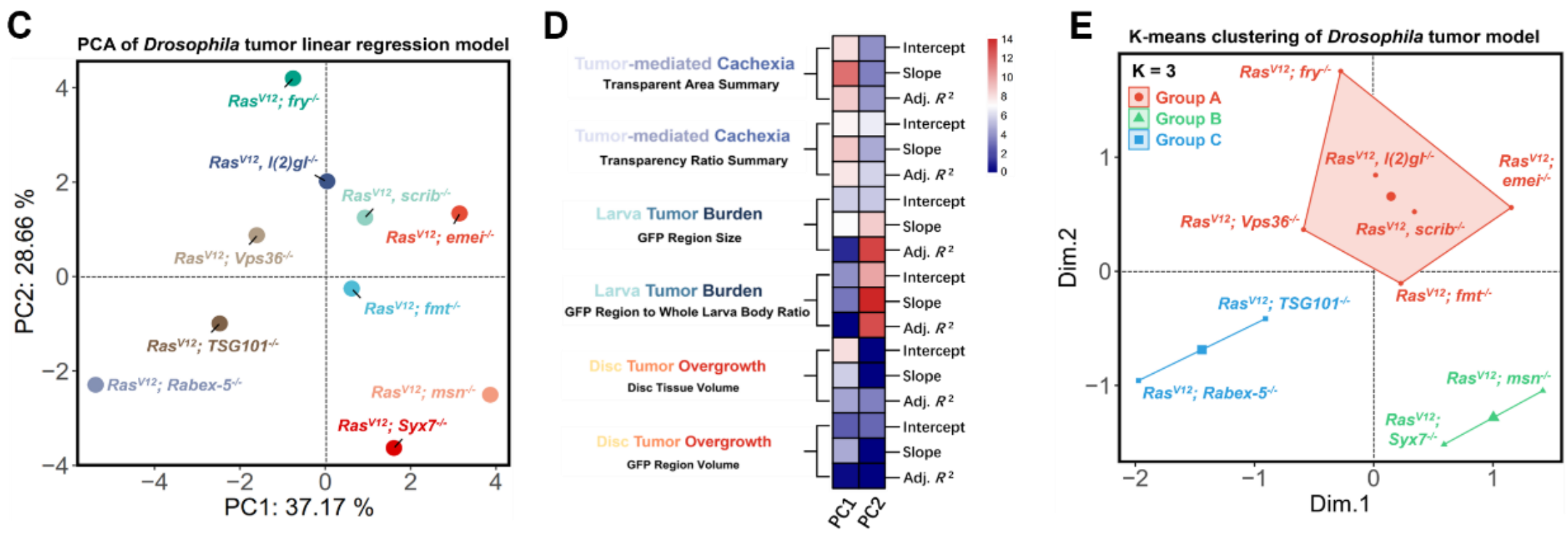

Figure 2. The classifications of *Drosophila Ras*-driven tumors.

(C) The two-dimensional scatter plot visualizing the PCA projection of *Drosophila Ras*-driven tumors. The PCA was performed based on the robust linear regression model parameters of each *Ras*-driven tumor, including the intercept, slope, and adjusted R-squared values.

(D) The contributions of PC1 and PC2 to the intercept, slope, and adjusted R-squared values of robust linear regression models representing tumor malignancies.

(E) The K-means clustering of *Drosophila Ras*-driven tumors based on PCA results. A total of three groups were classified, group A comprises *scrib*-, *l(2)gl*-, *fry*-, *Vps36*-, and *emei*-deficient tumor models; group B comprises *Syx7*- and *msn*-deficient tumor models; group C comprises *Rabex5*- and *TSG101*-deficient tumor models.

2. I find it is somewhat vague to use transparence area/ratio as an indicator for cachexia. I think the manuscript can benefit from clear cachexia definitions and assays.

Response: We appreciate this important point regarding the definition and quantification of cachexia. In the revised manuscript, we have explicitly defined cachexia by integrating fundamental clinical manifestations with established characteristics of tumor-induced wasting in *Drosophila* models. We have clarified the rationale for using "Transparent Area Summary" and "Transparency Ratio" (**Figs. 1E, 1E'**) as **robust, non-invasive proxies** for quantifying tissue wasting and lipid depletion in larvae. These metrics align with previously validated assays in the field. A detailed description of these definitions and the justification for our chosen metrics can be found on **page 6, lines 95–100** of the revised text.

3. For Figure 2C-E, PCA and K-means clustering are methods to visualize high dimensional data. With only three parameters (intercept, slope, adjust R2), the authors can easily visualize these parameters in a 3-D plot. Phenotypically, they measured tumor sizes at 3 time points and the invasion index. Various transformations do not change the information available for classification of the ten tumor models. It is much better to directly draw their interpretations from Figure S3 or Figure 1.

Response: Thank you for this insightful suggestion. Following the construction of robust linear regression models for each tumor across the three time points (Appendix Fig. S2A–S2C'), we initially attempted to visualize the three model parameters (intercept, slope, and adjusted R^2) in a three-dimensional space as recommended. However, we found that generating separate 3D plots for each of the six phenotypic metrics resulted in a fragmented view that did not effectively classify the ten tumor models. To address this, we integrated these parameters into a unified matrix and applied PCA and K-means clustering. This approach captures the combined contributions of intercepts, slopes, and adjusted R^2 values within the principal component loadings, thereby establishing a robust framework for tracking specific malignancy metrics across models. Furthermore, this strategy enables the identification of subtypes based on malignant progression phenotypes while maintaining a quantitative focus on the phenotypic dynamics illustrated in Figure 1. Guided by your comment, we have added a schematic diagram illustrating this analytical workflow (from linear regression to PCA) to clarify our rationale. These updates can be found in **Appendix Fig. S2D, Figs. 2C–2E, and page 7, lines 136–145** of the revised manuscript.

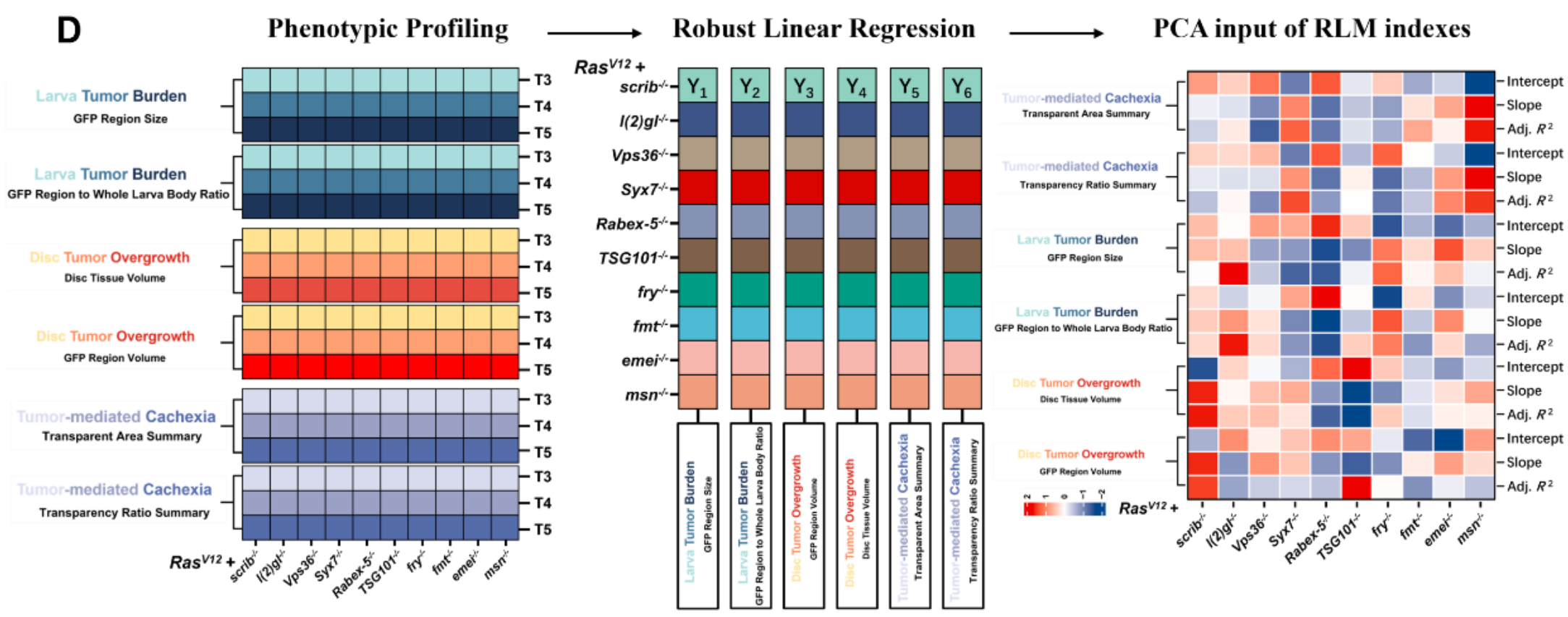

Appendix figure S2. The robust linear regression model of tumor malignancies in different *Drosophila Ras*-driven tumors.

(D) Schematic diagram illustrating principal component analysis (PCA) performed on robust

regression models constructed from phenotypic data across various DRTs. Using the intercept, slope, and adjusted R-squared values of these models as the input matrix, the further PCA analysis was conducted in conjunction with K-means clustering. This approach facilitates the identification of subtypes based on malignant progression phenotypes while preserving a quantitative focus on phenotypic dynamics.

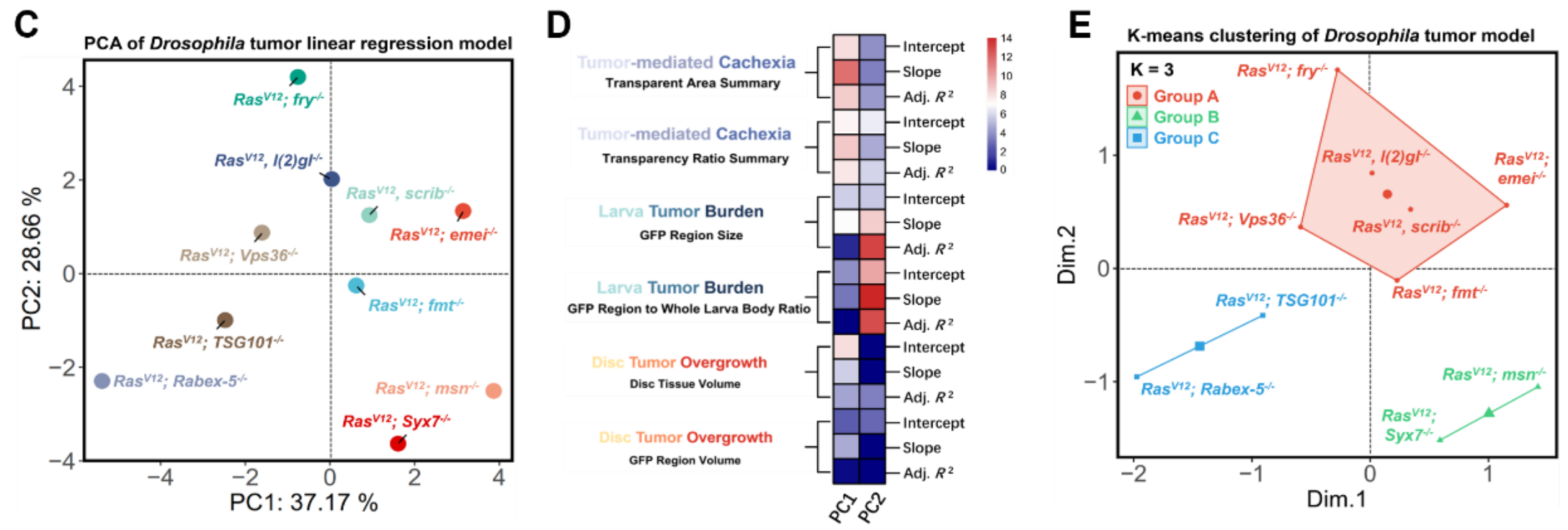

Figure 2. The classifications of *Drosophila Ras*-driven tumors.

(C) The two-dimensional scatter plot visualizing the PCA projection of *Drosophila Ras*-driven tumors. The PCA was performed based on the robust linear regression model parameters of each *Ras*-driven tumor, including the intercept, slope, and adjusted R-squared values.

(D) The contributions of PC1 and PC2 to the intercept, slope, and adjusted R-squared values of robust linear regression models representing tumor malignancies.

(E) The K-means clustering of *Drosophila Ras*-driven tumors based on PCA results. A total of three groups were classified, group A comprises *scrib*-, *l(2)gl*-, *fry*-, *Vps36*-, and *emei*-deficient tumor models; group B comprises *Syx7*- and *msn*-deficient tumor models; group C comprises *Rabex5*- and *TSG101*-deficient tumor models.

4. It wasn't obvious from Line 142 what transcriptomics datasets that the authors have generated. They can simply state that they generated transcriptomics datasets from different tumor models at different time points without confusing readers with pseudotime points. This would be an appropriate place to visualize their ten tumor models across time in a PCA plot.

Response: Thank you for your feedback regarding the clarity of our transcriptomics data description. Tumorigenesis is a dynamic, multi-step process characterized by significant spatiotemporal heterogeneity. In this study, we reconstructed the evolutionary trajectory from wild-type tissue to malignant tumors driven by sequential genetic mutations. Specifically, we defined five stages: wild-type tissue (T1), benign *Ras*^{V12}-mutant tumors (T2), and malignant tumors with increasing proliferative capacity (T3–T5). Because these stages represent a reconstructed progression rather than longitudinal samples from a single individual, we originally termed this a “pseudo-time points”. We agree that this terminology may cause confusion when referring to the actual sampling events. To address this, we have revised the text to clearly distinguish between the **“sampling time points”** (referring to the five distinct genotype groups) and the **“progression trajectory”** (representing the reconstructed temporal sequence from T1 to T5). We have removed the ambiguous use of “pseudotime points” when describing the datasets to

ensure clarity. These clarifications are detailed on **page 8, lines 150–155** of the revised manuscript.

5. It was not clear to me why the authors pick the Fry tumors to perform multi-omics analysis in Figure 5.

Response: We appreciate this important question. Our selection of *fry*-deficient tumors for multi-omics analysis was driven by two key considerations: **1) Addressing a Knowledge Gap:** While existing evidence suggests that *fry* and the associated NDR-kinase pathway possess tumor-suppressive functions, *Ras*-driven *fry*-deficient tumors have not been previously characterized. This represents a significant gap in our understanding of this specific tumor type. **2) Phenotypic Classification:** Based on statistical metrics of malignant progression and unbiased phenotypic clustering, *Ras*-driven *fry*-deficient tumors group within "Group A" alongside well-established malignant models (e.g., Ras^{V12},$scrib^{-/-}$ and Ras^{V12}, $l(2)gl^{-/-}$). Furthermore, we observed that *fry* deficiency, similar to *l(2)gl* and *Vps36* deficiencies, uniquely cooperates with various oncogenic drivers (Ras^{V12}, $Notch^{act}$, or Yki^{S168A}) to fuel malignancy. These findings indicate that Ras^{V12}, $fry^{-/-}$ tumors represent a **distinct and valuable model of malignancy**. Consequently, we performed an in-depth multi-omics investigation of these tumors to elucidate the molecular mechanisms driving their progression and to validate the feasibility of our ART-D integrated analysis framework.

6. For Figure 6F, how do the authors classify the expression levels of these proteins as high and low?

Response: Thank you for pointing out this ambiguity. We wish to clarify that the survival analysis in Figure 6F is based on **gene expression levels** of human orthologs across various *KRAS/NRAS*-driven cancers, rather than protein expression levels. We have corrected the figure legend to explicitly state "human ortholog gene expression levels". Regarding the classification, the optimal cut-off values to delineate "high" and "low" expression groups were determined using the *surv_cutpoint()* function from the *survminer* R package. Detailed descriptions of this methodology can be found in the revised manuscript (**Page 30, Lines 787-790; Page 53, Lines 1344-1345**).

7. I find Figure 8 existing only for trendy purpose and would recommend removing it to avoid overstatement. One can extract predictive gene signatures in TCGA datasets easily and there is no benchmark to say that DRTs are better.

Response: We sincerely appreciate this constructive critique. We agree that the original presentation of Figure 8 risked overstating our findings without a direct benchmark against other methods. In response, we have **consolidated Figures 7 and 8**, retaining only the data that substantively supports our core conclusions.

We have revised the text to clarify that this study does not claim superior predictive performance of DRT features compared to other established signatures for 1-, 3-, or 5-year survival. Instead, the primary value demonstrated here is the robust applicability of the ART-D framework across human *KRAS/NRAS*-driven cancers (achieving an AUC > 0.85). We acknowledge that a systematic comparison with other indicators (e.g., Tumor Mutational Burden,

Copy Number Variation) using advanced machine learning algorithms is a vital next step. Please refer to the updated **Figure 7 and Figure EV5** in the revised manuscript for the consolidated data and refined discussion.

Fig 7.

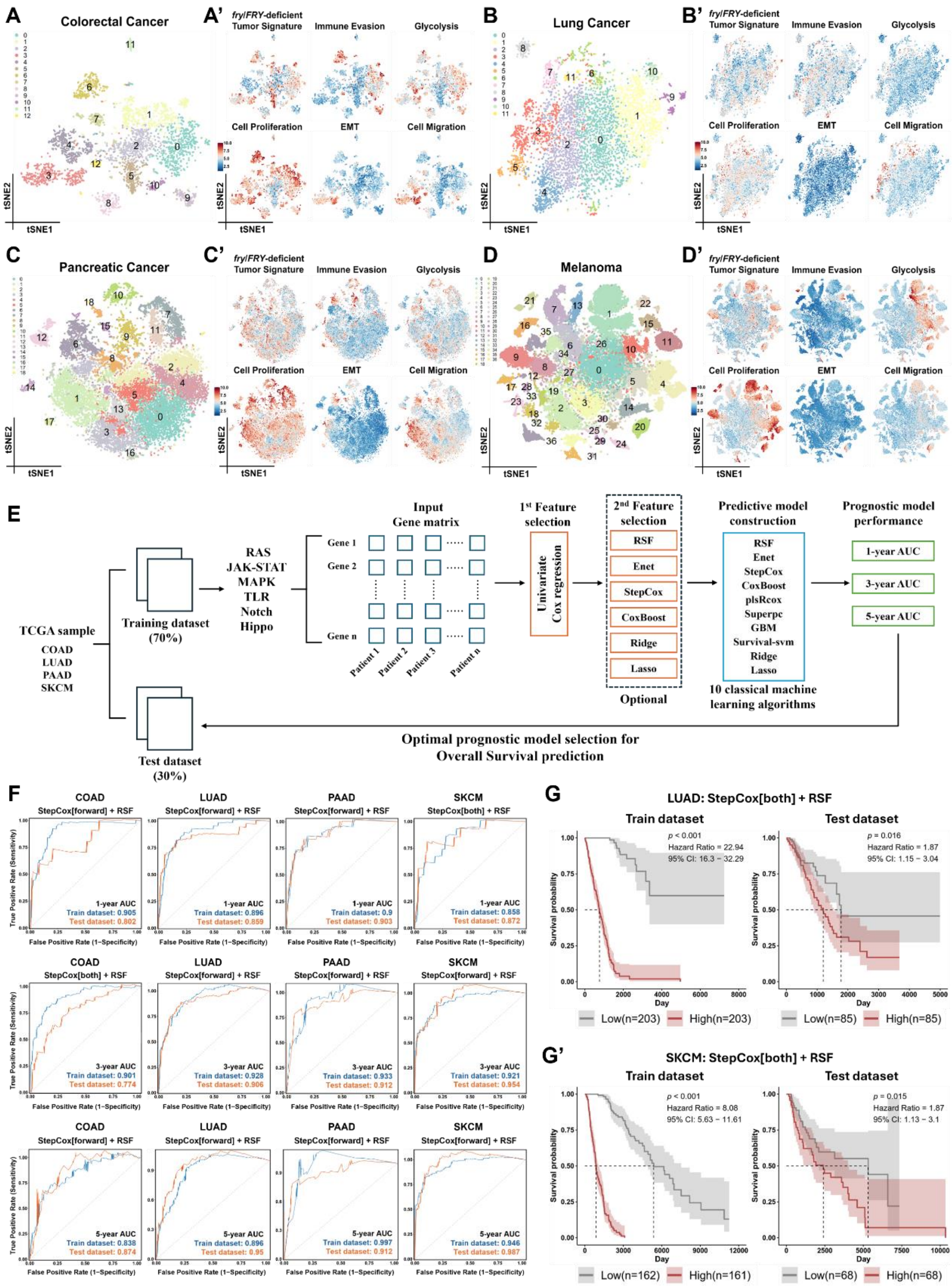

Figure 7. Cross-species validation of evolutionarily conserved oncogenic mechanisms from *Drosophila* to humans.

(See legends in the revised manuscript)

Fig EV5.

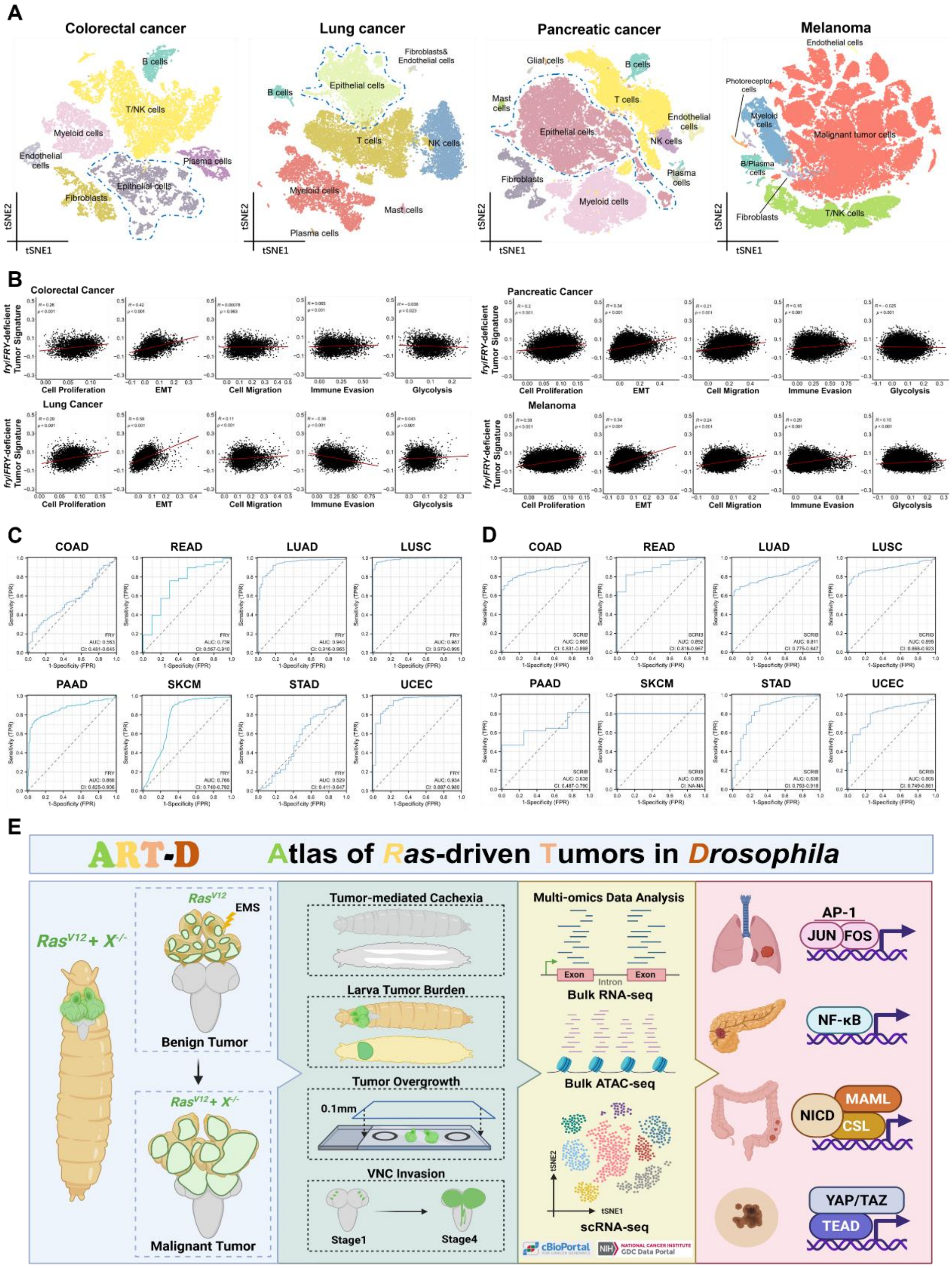

Figure EV5. Cross-species insights from ART-D on evolutionarily conserved oncogenic mechanisms.

(See legends in the revised manuscript)

Reviewer #3

Guo et al present an Atlas of Ras-driven tumors using Drosophila models and human cancer data to signature changes in gene expression during tumor initiation, promotion and growth. They divide the vast analyses of transcriptional dynamics into early and malignant tumorigenesis. Using fly and human cancer data sets and multi-OMICS approaches, and a plethora of bioinformatics they define key changes and attributes of Ras driven tumors. The multiple approaches and analyses reveal differences in the early vs late (invasive) phenotypes. However, the use of computational tools is confusing as the purpose of the analysis and their outcomes are not clearly explained. This manuscript will benefit immensely, if the following major and minor points are addressed by the authors.

Response: Thank you for recognizing the value of our study. In this revision, we have significantly rewritten the relevant sections to explicitly define the purpose, methodology, and biological implications of each bioinformatic tool used. We believe these changes have greatly improved the narrative flow and accessibility of our findings. Below, we provide a point-by-point response to the specific major and minor concerns raised.

MAJOR POINTS

1. Previous transcriptomic studies with RasV12 lgl-/- or scrib-/- Drosophila tumors have identified key transcriptional networks (Atkins et al., 2016; Kulshammer et al., 2015, Floc'hlay et al., 2023, and others), and have identified various transcriptomic changes. How does the approach taken by Guo et al., help generate a unified framework when the timepoints that are chosen for analyses are not comparable? Guo et al., use 5.5days AEL, 9.5 days AEL and 13.5 days AEL as initiation, progression and advanced stages for their study.

Response: Thank you for this insightful comment. We agree that while extensive research has characterized *Ras*^{V12}, *scrib*$^{-/-}$ and *Ras*^{V12}, *l(2)gl*$^{-/-}$ tumors, methodological heterogeneity across previous studies has indeed hindered direct comparability. To address this limitation, our study establishes a unified framework by systematically characterizing tumor progression, malignant phenotypes, and transcriptomic profiles for these models (and their controls) under identical experimental conditions. Crucially, we employed absolute quantification and standardized immunofluorescence staining to generate high-resolution phenotypic data across three defined developmental stages (5.5, 9.5, and 13.5 days AEL), representing initiation, promotion, and progression malignancy, respectively. By integrating our comprehensive gene expression matrices (**Dataset EV1**) with this rigorously quantified phenotypic source data, we provide a standardized resource. This allows the research community to directly compare diverse biological features, such as tumor growth kinetics, invasion potential, and tumor-induced cachexia, across different genetic backgrounds within a single, coherent analytical framework.

2. Guo et al also describe a genetic screen in which they identified novel tumor suppressors that synersize with RasV12 to drive malignant growth. From the screen, Furry is characterized in the study. Why were the other mutants not pursued is not explained.

Response: Thank you for your question regarding our candidate selection strategy. We prioritized *fry*-deficient tumors for in-depth multi-omics characterization based on two primary rationales: **1)**

Addressing a Knowledge Gap: While existing evidence suggests that *fry* and the associated NDR-kinase pathway possess tumor-suppressive functions, *Ras*-driven *fry*-deficient tumors have not been previously characterized. This represents a significant gap in our understanding of this specific tumor type. **2) Phenotypic Classification:** Based on statistical metrics of malignant progression and unbiased phenotypic clustering, *Ras*-driven *fry*-deficient tumors group within "Group A" alongside well-established malignant models (e.g., Ras^{V12},$scrib^{-/-}$ and Ras^{V12}, $l(2)gl^{-/-}$). Furthermore, we observed that *fry* deficiency, similar to *l(2)gl* and *Vps36* deficiencies, uniquely cooperates with various oncogenic drivers (Ras^{V12}, $Notch^{act}$, or Yki^{S168A}) to fuel malignancy.

Collectively, these findings indicate that Ras^{V12}, $fry^{-/-}$ tumors represent a **distinct and valuable model of malignancy**. We selected this model for deep interrogation to derive representative insights and to validate the utility of our **ART-D** integrated multi-omics and multi-timepoint framework. Our goal was to demonstrate how this paradigm can systematically elucidate the molecular mechanisms driving malignant progression. We acknowledge that characterizing the other hits from the screen is an important future direction. As noted in the revised **Discussion** section (**Page 21, Lines 538-545**), we propose that future studies should leverage this framework to integrate untargeted lipidomics and epigenomics across various *Ras*-driven tumors, thereby constructing an even more comprehensive and robust atlas of tumorigenesis.

3. The authors use statistical and other computational approaches - the purpose of selecting the tool is not clear and the resulting data is also not clearly explained. For example:
a. Why did the authors use the linear regression and calculate slope and intercept? Can the authors claim that a change in the slope or intercept unambiguously indicates initiation versus progression specific changes? How does the PCA help in this regard. A number of tools are mentions without explaining how that helps learn more about the datasets. For example, why did the authors choose "K means clustering"- is it routinely used for tumor subtyping? If yes, please reference the studies.

Response: Thank you for these insightful questions regarding our analytical strategy. Given that our phenotypic data on tumor progression constitute strictly sampled time-series data, we employed **robust linear regression** to model general progression patterns. Unlike conventional linear regression, the robust variant effectively mitigates the influence of outliers, ensuring a more accurate characterization of these trends.

As stated in the revised manuscript, the regression parameters serve specific biological interpretations: the **intercept** reflects initial-stage malignancy, the **slope** indicates the rate of progression from the initial to the advanced stage, and the **adjusted R^2** denotes the goodness-of-fit for each model. Following the construction of these models for each tumor across the three time points (**Appendix Fig. S2A-S2C'**), we extracted these three parameters to generate a feature matrix for downstream **PCA** and **K-means clustering**. In this context, PCA reduces the dimensionality of the data, where the loadings of each PC reveal the relative contributions of the intercept, slope, and R^2 values, thereby facilitating the identification of dominant malignancy metrics. Subsequently, K-means clustering was applied to these PCA-transformed features to objectively stratify tumors into distinct subtypes based on their progression dynamics, a method widely used in unsupervised tumor subtyping studies.

Inspired by suggestions from both you and *Reviewer #2*, we have added a **schematic diagram** illustrating the entire workflow, from robust linear regression to PCA and clustering, to clarify the rationale behind this multi-step analytical strategy. Please refer to the new **Appendix Fig. S2D** and the updated text on **page 7, lines 126-128 and 136-145**.

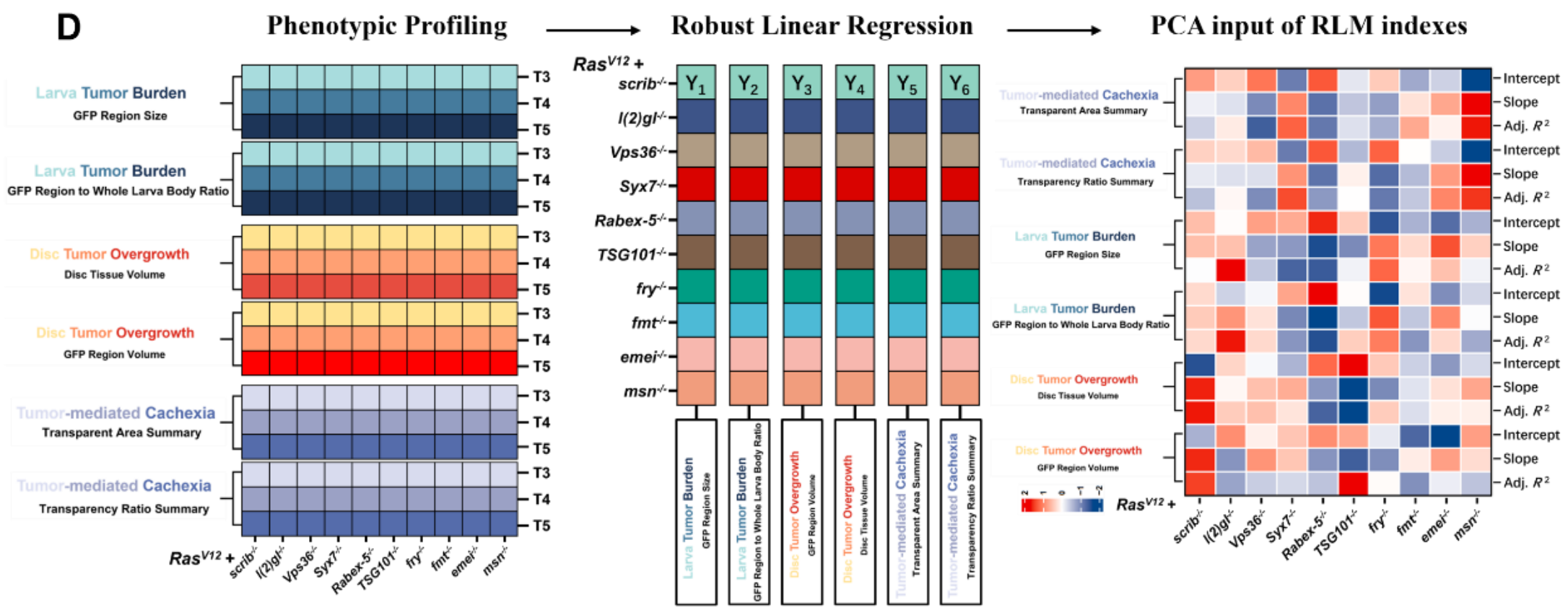

Appendix figure S2. The robust linear regression model of tumor malignancies in different *Drosophila Ras*-driven tumors.

(D) Schematic diagram illustrating principal component analysis (PCA) performed on robust regression models constructed from phenotypic data across various DRTs. Using the intercept, slope, and adjusted R-squared values of these models as the input matrix, the further PCA analysis was conducted in conjunction with K-means clustering. This approach facilitates the identification of subtypes based on malignant progression phenotypes while preserving a quantitative focus on phenotypic dynamics.

b. 5 categories of Drosophila controls and Ras-driven tumors (DRTs) are presented based on pseudo-timepoint based categorization. However, the analyses that follow are not corelated to the time points, as the authors write "However, not all observed dynamics ----tumor malignancy progression." (Line 150). The value of this data is therefore diminished or unclear. Authors should explain why this approach was important for establishing a framework for collating the transcriptomics data from DRTs.

Response: Thank you very much for your valuable feedback regarding the integration of our pseudo-timepoint categorization with downstream analyses. In traditional multi-timepoint tumor studies, the focus has predominantly been on analyzing gene expression trends in isolation. However, not all these trends were **significantly** associated with the tumor malignancy progression (**page 8, Line 159**), and the scarcity of high-quality, longitudinal phenotypic data often hinders the unbiased correlation of specific gene trends with distinct phenotypic stages.

To address this limitation, our **ART-D framework** reverses the conventional approach: it initiates analysis from the perspective of **malignant progression phenotypes** to first stratify tumor subtypes. By integrating multi-timepoint transcriptomic data with these high-quality phenotypic profiles via **Weighted Gene Co-expression Network Analysis (WGCNA)**, we first identify gene modules that are significantly associated with characteristic phenotypic states.

Subsequent analyses, including gene trend assessment, Over-Representation Analysis (ORA), and gene regulatory network reconstruction, are then performed **within** these phenotype-linked

modules. This strategy ensures that the identified candidate genes and signaling pathways are not only statistically correlated with tumor progression but also exhibit highly consistent expression trends aligned with the phenotypic trajectory. Thus, the pseudo-timepoint categorization serves as the essential scaffold that anchors our transcriptomic findings to biological reality.

We have clarified this logical framework in the revised manuscript. Please see **Fig. 3A** and the updated description on **page 20, lines 499-515**.

Fig 3.

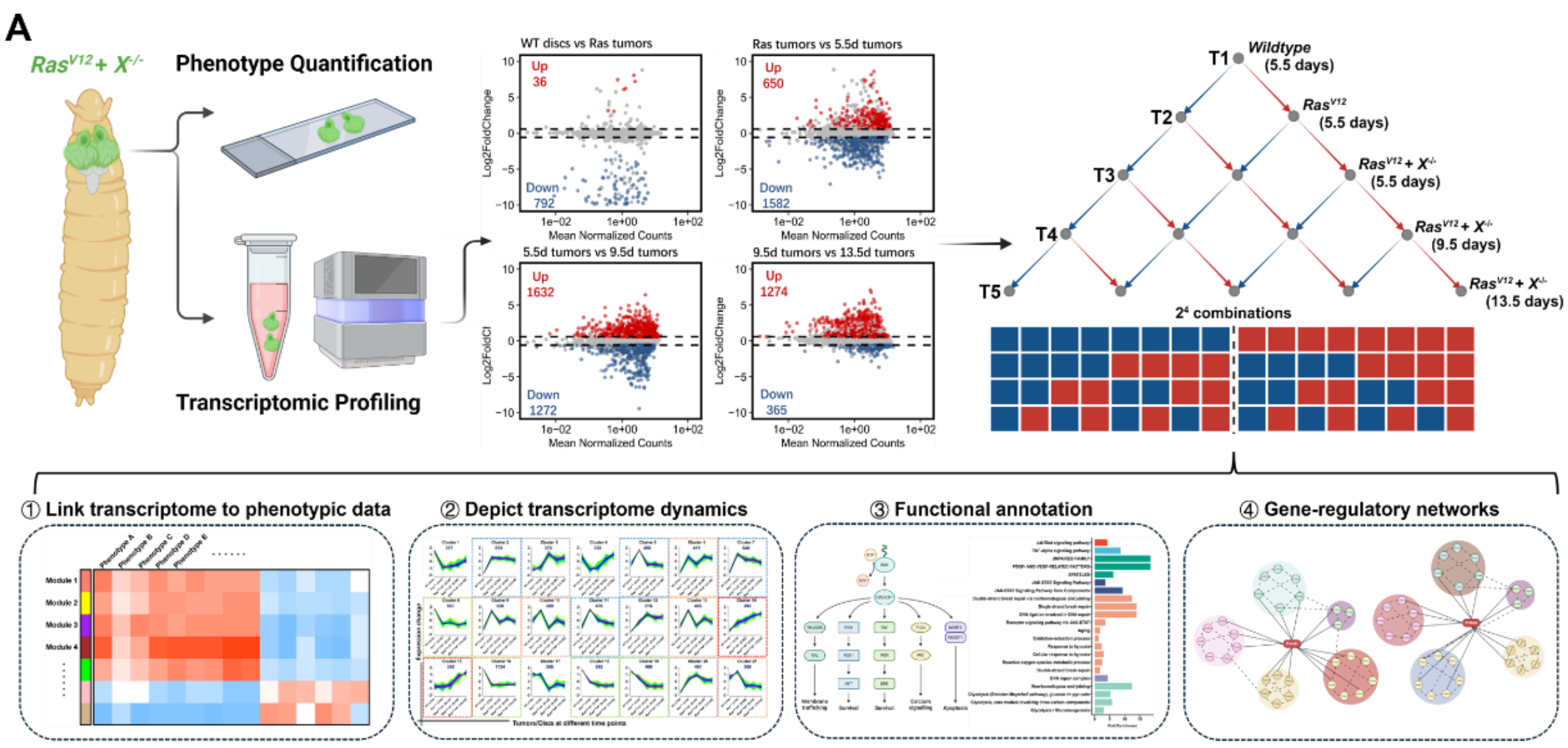

Figure 3. Dynamic transcriptomic profiles of different subtypes of *Drosophila Ras*-driven tumors.

(A) The schematic diagram outlining the workflow for identifying tumor transcriptomic dynamics. The process began with the acquisition of bulk RNA-seq data from various tumors at different time points (Step 1), followed by the identification of DEGs (Step 2), the analysis of gene dynamic changing patterns (Step 3), and the extension of analyses (①~④) link transcriptomic changes with phenotypic malignancies (Step 4).

4. The authors carry out extensive analyses of Drosophila cancer models and human cancers from several datasets. Interestingly, the key pathways identified in this study have been identified earlier. The authors should discuss if the pathways like Notch, JNK, NF-kB and Hippo are necessary and sufficient for all observed criteria for tumor malignancy, or these are initiators of the changes and other suspected tumor promoters are involved. The role of endocytic, polarity regulators is also widely described in flies, it would be useful to correlate the cell biological effects of these regulators and to place them in the initiator vs progression category, which is unclear in the current manuscript. If other tumor promoters are involved, the suspected genes or pathways revealed in the data analyses should be discussed.

Response: We sincerely thank you for this insightful comment. Our investigation into the temporal dynamics and malignant progression of *Ras*-driven tumors reveals that while Notch hyperactivation and Hippo inactivation facilitate excessive overgrowth, they function as part of a broader cooperative network rather than acting alone. To address your question on whether these

pathways are necessary and sufficient, or merely initiators, we performed functional validation experiments. We expressed constitutively active Notch (*Notch*^{act}) or Yki (*Yki*^{S168A}) in GFP-marked cells deficient for specific tumor suppressors (*l(2)gl*, *Vps36*, or *fry*). Our results demonstrate that loss of these three distinct tumor suppressors creates a unique permissive environment that cooperates with multiple oncogenic drivers (*Ras*^{V12}, *Notch*^{act}, or *Yki*^{S168A}) to drive malignancy. Given that *l(2)gl*, *Vps36*, and *fry* regulate distinct biological processes, we have expanded our discussion to analyze their cell biological effects and oncogenic mechanisms individually.

Specifically, we conducted an in-depth analysis of *Ras*^{V12}, *fry*$^{-/-}$ tumors. By integrating our findings with multi-omics data from human *KRAS/NRAS*-driven cancers, we identified four evolutionarily conserved signaling pathways—JNK, NF-κB/Toll, Hippo, and Notch—that act as **cooperative drivers** of tumorigenesis in *Ras*-activated, *fry*-deficient contexts.

To explicitly distinguish between “initiators” and “progression/maintenance” factors, we investigated the necessity of these pathways for tumor maintenance. We inhibited key effectors of JNK (*Tak1*, *hep*, *bsk*, *kay*, *Jra*), Toll (*Tl*, *Dorsal*, *Dif*), and Notch signaling (*Delta*, *Ser, Notch*), while simultaneously activating the Hippo pathway via overexpressing *wts*. Our results show that disrupting any of these pathways significantly suppresses tumor growth (quantified by total disc size, GFP^+ tumor area, and tumor occupancy; $p < 0.05$). Furthermore, these interventions significantly rescued the pupariation delay observed in larvae bearing *Ras*^{V12}, *fry*$^{-/-}$ tumors. These findings suggest that while the loss of polarity/endocytic regulators (like *fry*) may act as the **initiator** by creating a susceptible cellular context, the identified signaling pathways (JNK, Toll, Notch, Hippo) are **necessary drivers** required for the continued progression and maintenance of the malignant phenotype.

We have revised the manuscript to clearly articulate this distinction and discuss the suspected genes and pathways revealed by our data analyses. Please refer to **Figs. 4-5**, **Appendix Figs. S7-S10**, and the updated text on **pages 12-15 (lines 260-354)**.

Fig 4.

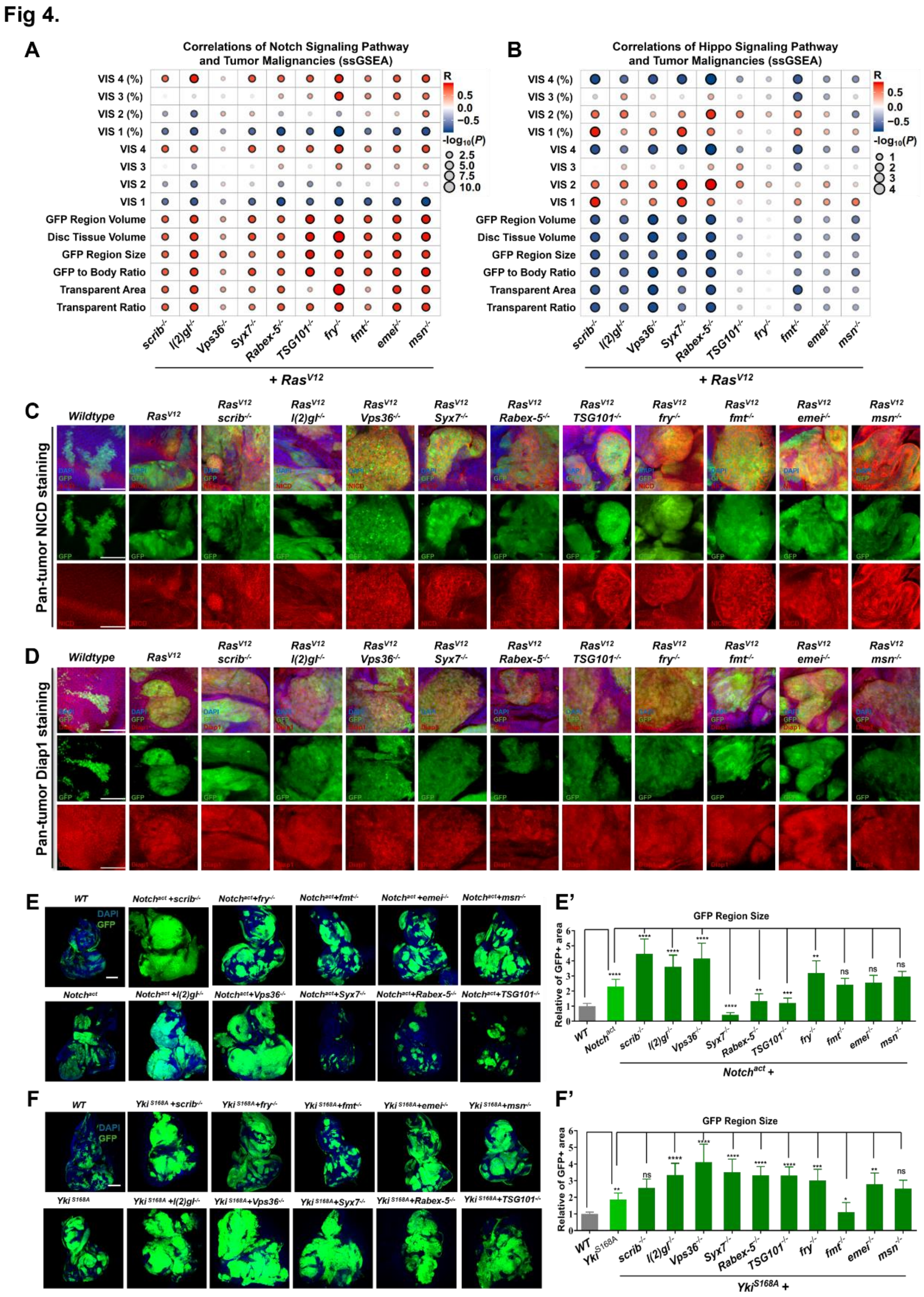

Figure 4. The tumor promoting role of Notch activation and Hippo inactivation within of *Drosophila Ras*-driven tumors.

(See legends in the revised manuscript)

Fig 5.

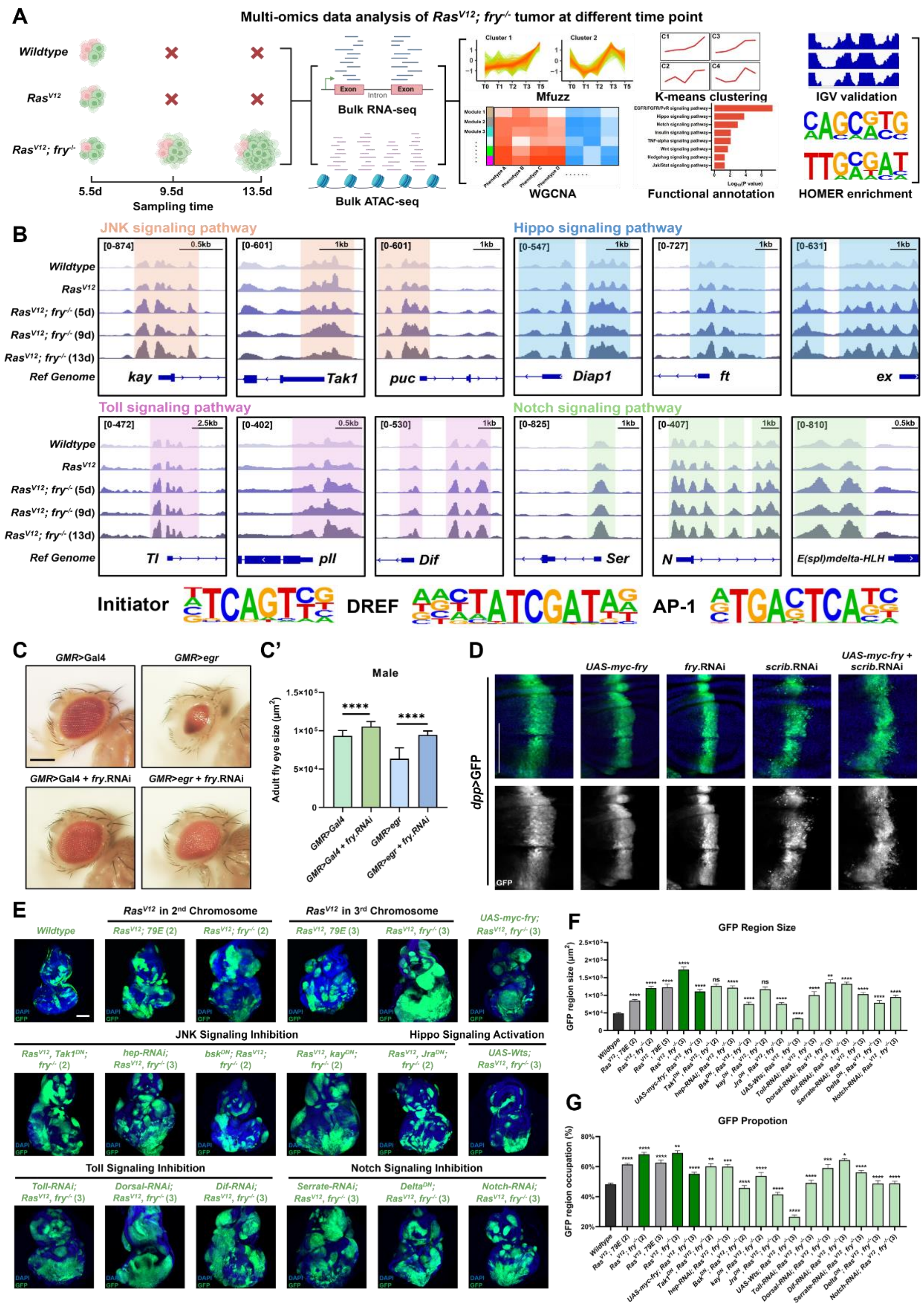

Figure 5. Multi-omics data analysis of Ras^{V12}; $fry^{-/-}$ tumors across different stages.

(See legends in the revised manuscript)

Appendix Fig S7.

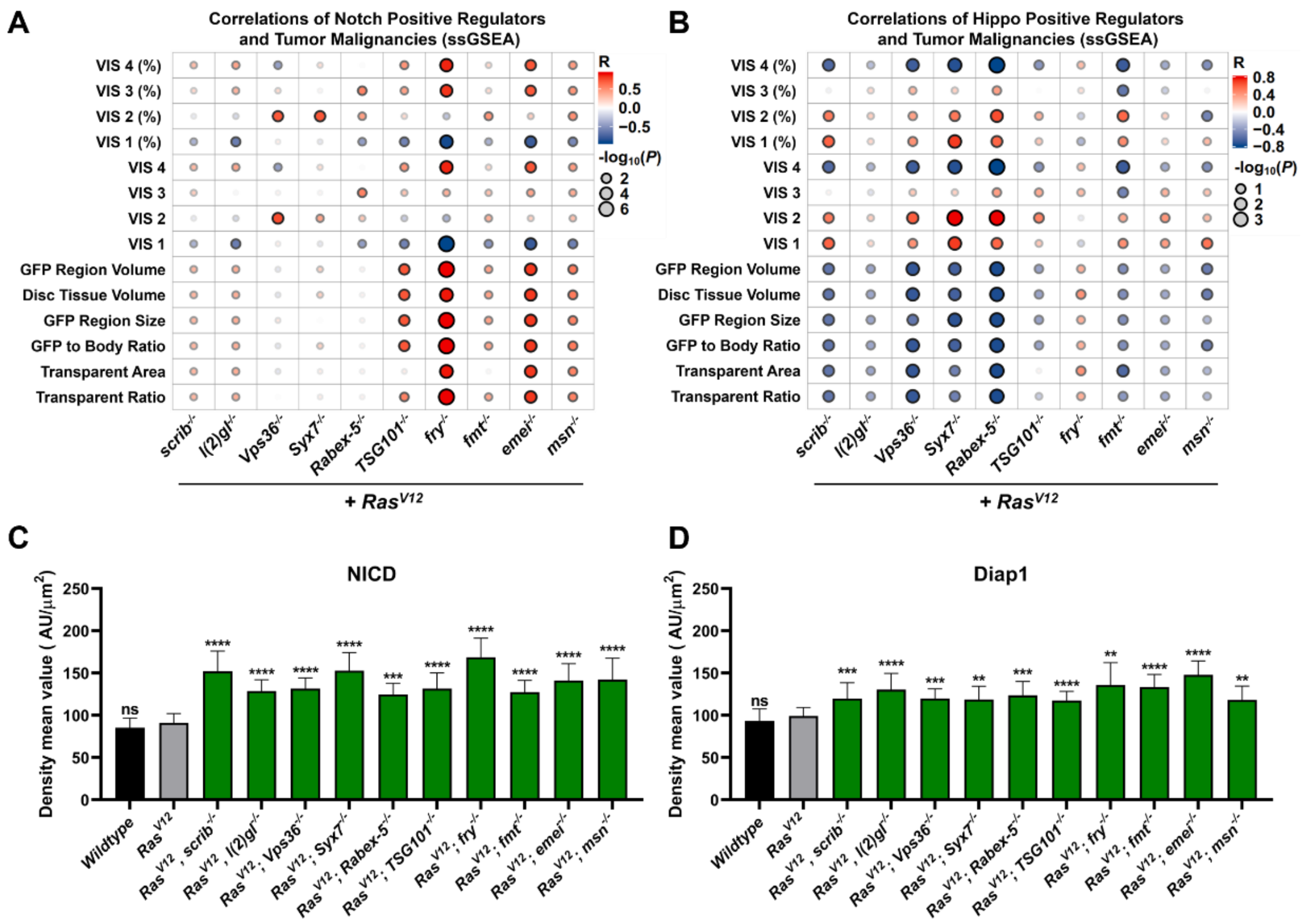

Appendix figure S7. The Notch activation and Hippo inactivation across different *Drosophila Ras*-driven tumors.

(See legends in the revised Appendix File)

Appendix Fig S8.

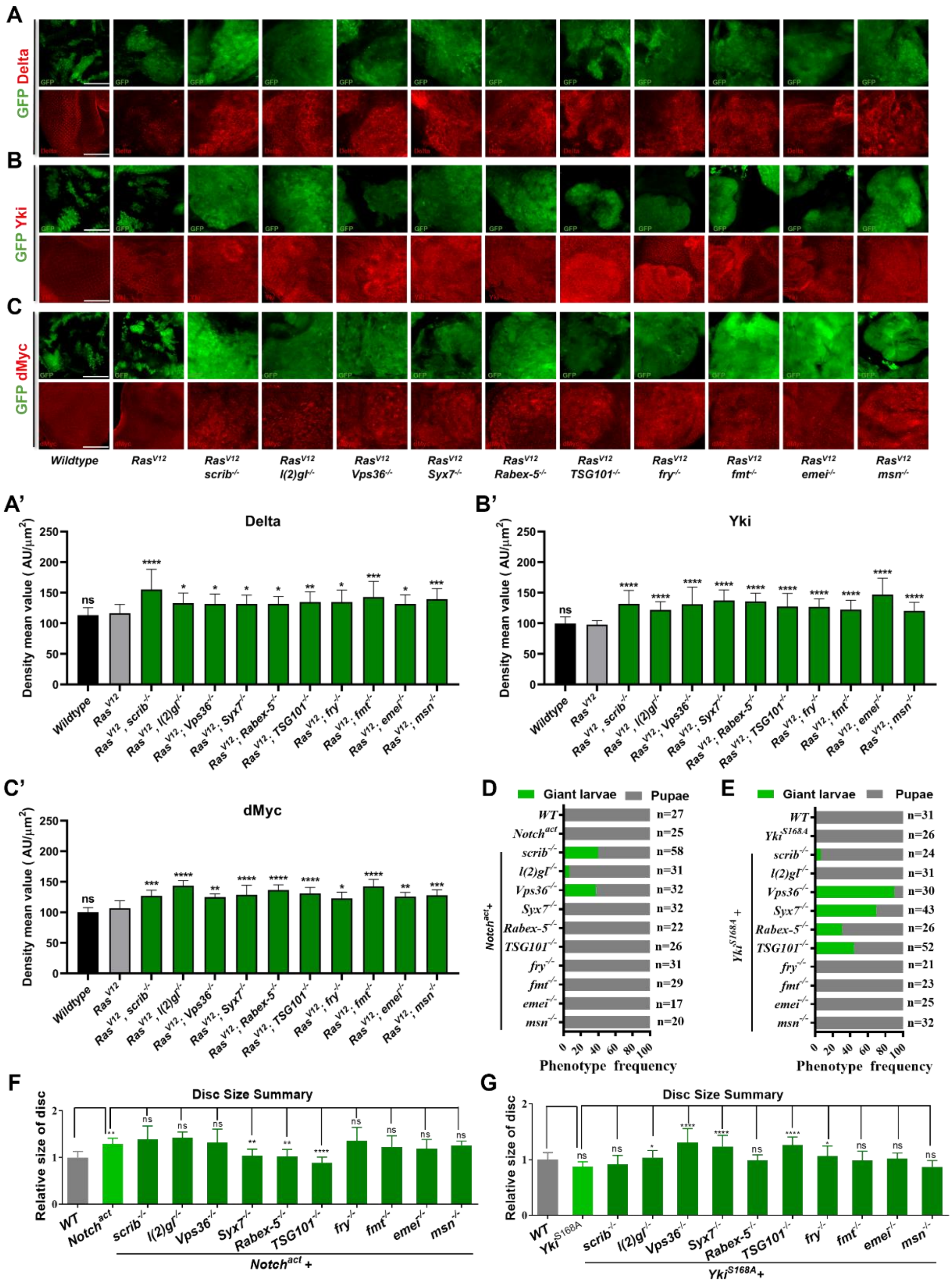

Appendix figure S8. The tumor promoting roles of Notch activation and Hippo inactivation across different *Drosophila Ras*-driven tumors.

(See legends in the revised Appendix File)

Appendix Fig S9.

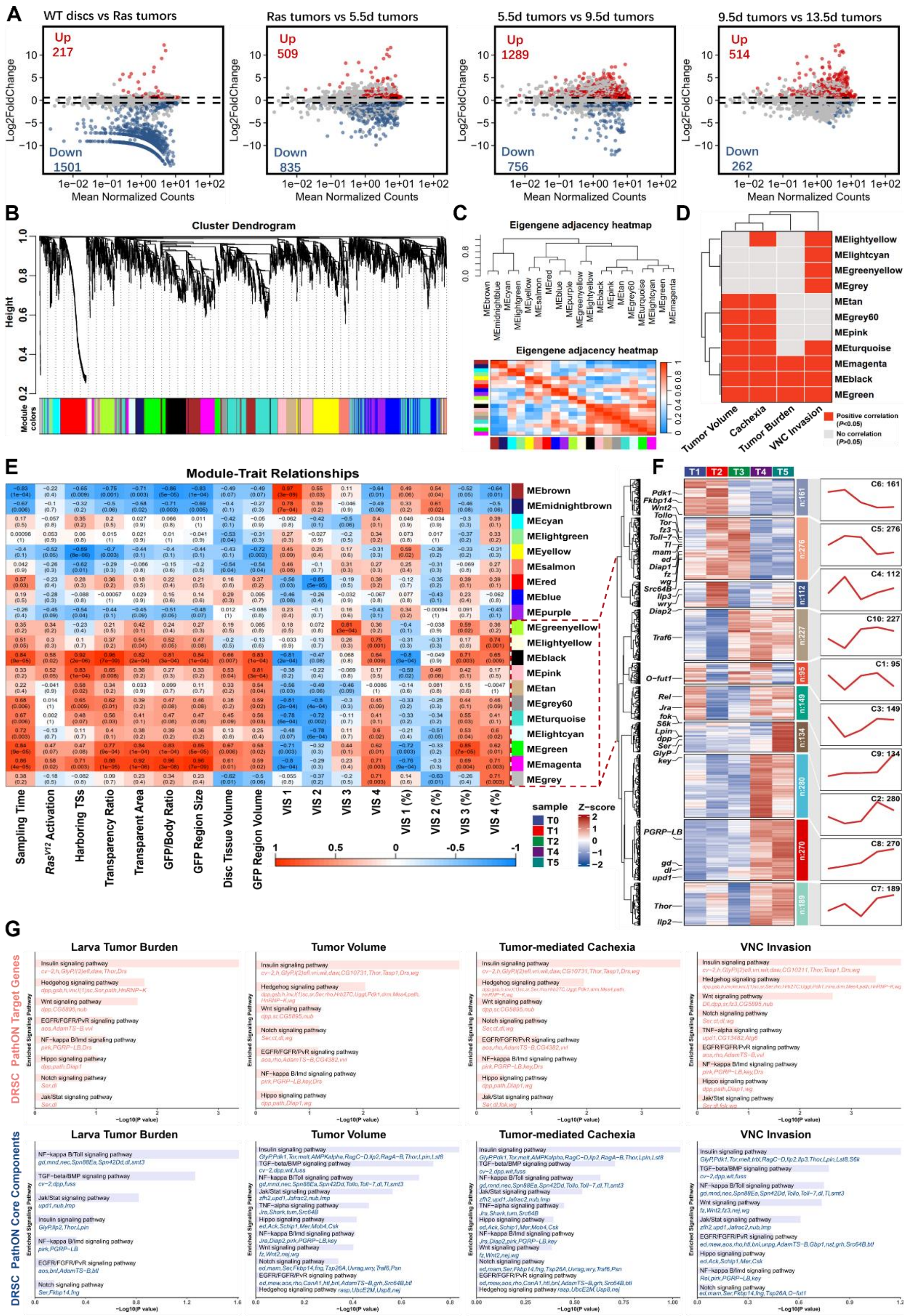

Appendix figure S9. Data characteristics and signaling pathways identified as responsible for phenotypes during *Ras*^{V12}*; fry*$^{-/-}$ tumor progression.

(See legends in the revised Appendix File)

Appendix Fig S10.

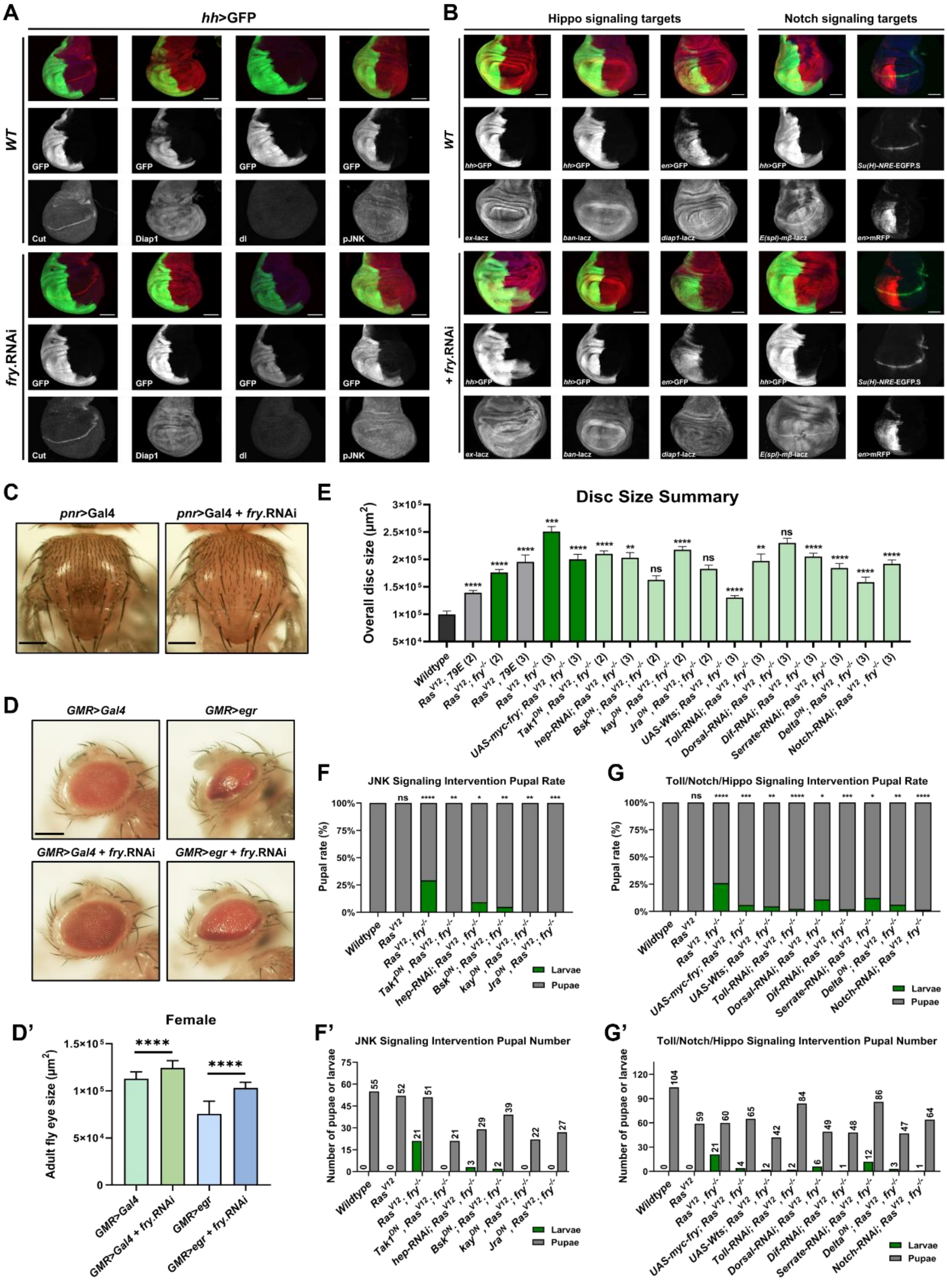

Appendix figure S10. Evaluation of physiological and pathological functions of *fry*.

(See legends in the revised Appendix File)

Minor points:

1. How did the PCA lead to formation of three groups? Did all genes within Groups A-C show similar effects? For example, did fry and scrib mutant cells show similar effects in the PCA? Was this classification used for any further analyses in this study?

Response: Thank you for this insightful question regarding our clustering methodology. As detailed in our response to **Major Point 3a**, we first constructed robust linear regression models for each tumor genotype across three time points (Appendix Figs. S2A–S2C'). We extracted three key parameters from these models to generate a new feature matrix, which served as the input for both PCA and K-means clustering (Appendix Fig. S2D; Figs. 2E–3G). While PCA provided a visual representation of the data distribution, it was the subsequent K-means clustering that formally identified three distinct tumor subtypes ($n = 3$). Regarding your specific example, the PCA results indeed revealed that ***fry* and *scrib* mutant cells exhibit similar profiles** concerning Larva Tumor Burden and Tumor-mediated Cachexia; **however, they diverge significantly in terms of Disc Tumor Overgrowth** (Appendix Fig. S2D). This classification was central to our downstream analyses. We found that these three subtypes possess distinct biological characteristics, ranging from specific signaling pathway activations to metabolic reprogramming (Figs. EV2–EV4; Figs. 3B–3D). For instance, while all *Ras*-driven genetic backgrounds correlated with Notch activation and Hippo inactivation, replacing Ras^{V12} with $Notch^{act}$ and Yki^{S168A} induced consistent malignant overgrowth **only** in Group A mutants ($l(2)gl^{-/-}$, $Vps36^{-/-}$, and $fry^{-/-}$). This confirms that our subtyping effectively stratifies tumors based on their underlying molecular dependencies and phenotypic outcomes.

2. Fry mutations are extensively analyzed and its role in Ras Driven tumors shows interesting effects. However, no deep analysis is provided for the observation that Fry knockdown has no effect on JNK, NFKB, Toll, Hippo or Notch pathways. What molecular mechanism(s) result in synergistic interactions between Fry and RasV12 to drive oncogenic malignant growth remains unanswered?

Response: Thank you for this critical observation. We wish to clarify that our data demonstrate that under **pathological conditions** (specifically in $fry^{-/-}$; Ras^{V12} tumors), the JNK, Hippo, Toll, and Notch pathways contribute significantly to tumor progression (**Figs. 5C-5G**). However, we acknowledge that the **direct molecular mechanisms** by which *fry* loss regulates these pathways under **physiological conditions** remain to be fully elucidated.

While we are actively designing follow-up experiments, including tag-specific immunoprecipitation coupled with tandem mass spectrometry (IP-MS/MS) to identify novel *Fry*-interacting proteins, to uncover the basis of this synergistic interaction, we believe these extensive mechanistic studies fall beyond the primary scope of the current manuscript. Instead, our current work focuses on establishing phenotypic synergy and pathway dependencies. We have added a dedicated discussion in the revised manuscript (**Page 21, Lines 546-553**) to explicitly address these limitations and outline our future directions for translating these findings into potential therapeutic targets.

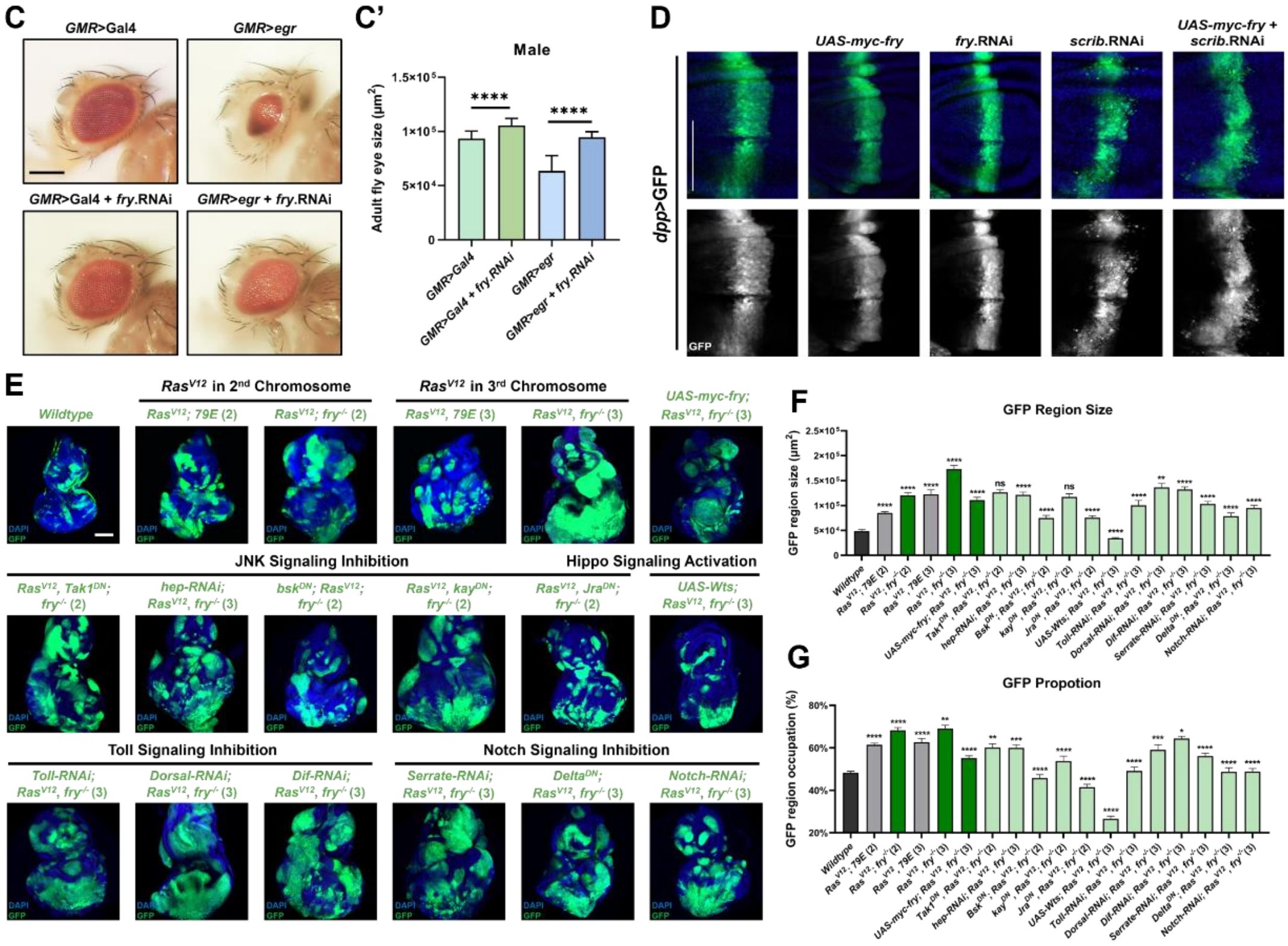

Figure 5. Multi-omics data analysis of *Ras*^{V12}; *fry*$^{-/-}$ tumors across different stages.

(C-C’) The light micrographs (C) and quantification results (C’) of adult eyes (Male) with the indicated genotypes. Scale bars: 200 μm. Statistical analysis for each group was performed using the Mann-Whitney U-test or Student’s t-test based on the variance. Results were presented as means ± SD, n >25. Significance of different genotypes were denoted as follows: ns > 0.05, **P* < 0.05, ***P* < 0.01, ****P* < 0.001, *****P* < 0.0001.

(D) The fluorescence micrographs of wing pouch regions. Wing discs were placed as anterior to the left and dorsal up with genotypes denoted. Scale bars: 100 μm.

(E) The confocal images of JNK signaling inhibition, Hippo signaling inactivation, NF-κB/Toll signaling inhibition, and Notch signaling inhibition in *Ras*-driven *fry*-deficient tumors. To ensure precise data quantification, we generated *fry*-deficient tumors expressing *Ras*^{V12} on either the second (2nd) or third (3rd) chromosome of *Drosophila*, designated as *Ras*^{V12}; *fry*$^{-/-}$ (2) and *Ras*^{V12}, *fry*$^{-/-}$ (3), respectively, with the chromosomal location of *Ras*^{V12} indicated in parentheses. This nomenclature was applied consistently to both experimental and control groups, followed by statistical analysis of the corresponding tumor samples. Samples were observed at 5.5-6.5 days AEL. Scale bar: 100 μm.

(F) Quantification of the GFP region size within eye-antennal disc across different genotypes in Fig. 5E. Statistical significance was assessed using either the Mann–Whitney U-test or Student's t-test, depending on variance homogeneity (n > 25). Experimental groups were compared against the corresponding *Ras* control group. Data are presented as means ± SEM. Significance levels are denoted as follows: ns > 0.05, **P* < 0.05, ***P* < 0.01, ****P* < 0.001, *****P* < 0.0001.

(G) Quantification of GFP region occupation within eye-antennal disc across different genotypes in Fig. 5E. Statistical significance was assessed using either the Mann–Whitney U-test or Student's t-test, depending on variance homogeneity ($n > 25$). Experimental groups were compared against the corresponding *Ras* control group. Data are presented as means ± SEM. Significance levels are denoted as follows: ns > 0.05, *$P < 0.05$, **$P < 0.01$, ***$P < 0.001$, ****$P < 0.0001$.

18th Apr 2026

Manuscript Number: MSB-2025-13410R
Title: Cross-Species Insights from ART-D to Uncover Evolutionarily Conserved Oncogenic Mechanisms
Author: Yifan Guo
Jiadong Zheng
Yixin Wu
Kun Zhao
Dian Lv
Wenhan Liu
Mengling Wang
Jinyu Lu
Wenyan Xu
Xianping Wang
Xianjue Ma

Dear Xianjue,

Thank you for submitting the revised version of your manuscript. We have now received feedback from the reviewer who was asked to re-evaluate your study. As you will see from the comments below, Reviewer #2 is generally satisfied with the revisions you have made, while still raised a -relatively minor- concern. We ask that you provide a response to the remaining comment.

On a more editorial level:

1. Remove the "Authors' contribution" section from the manuscript file.

2. Remove all figures from the manuscript file and retain only the main and EV figure legends at the end of the manuscript.

3. The references need to be formatted according to the Molecular Systems Biology reference style. Please list up to 10 co-authors of a paper before adding et al.
in the reference list. Citations should be listed in alphabetical order.

4. The following funding information is missing from the online submission system and should be included: Zhejiang Provincial Natural Science Foundation Project (LQKWL26C0701).

5. Appendix:
- The author list and affiliations should be removed from the cover page.
- The panels in Appendix Figure S8 do not match between the image and the caption (panels D-E are not listed in the caption, while panels H-I are listed in the caption but not present in the figure). Please correct this discrepancy.

6 "Materials and Methods" should be renamed to "Methods."

7. "Declaration of interests" should be renamed to "Disclosure and Competing Interests Statement."

8. Remove the references from the Reagents and Tools table.

9. Data availability
- "Data and code availability" should be renamed to "Data availability."
- Please provide the URL for PRJNA1209517.

10. Source Data should be uploaded as one zipped file per figure and named as "manuscriptID_SourceDataForFigure x."

11. BioRender should be acknowledged at the end of the Methods section in the following way:
Graphics:
(some of the... OR Figure #... OR synopsis) Graphics were created with BioRender.com.

12. Please address the following issues in figure legends:
- Please note that the exact p values are not provided in the legends of figures 1C-E'; 2A', B'; 4E', F', 5C', F, G; 6E, F; 7G, G'; EV1 C-E.
- Please indicate the statistical test used for data analysis in the legends of figures 6E, F; 7G, G'; EV2 A, EV3 A, EV4 A.
- Please note that for heatmap present in figures EV2 A, F, G; EV3 A, F, G; EV4 A, F, G a numbered scale bar is not provided.

This needs to be rectified.
- Please note that the scale bar needs to be defined for figures 4E, F.

13. The manuscript sections should be in the following order: Title page - Abstract - Introduction - Results - Discussion - Methods -Data Availability - Acknowledgments - Disclosure and Competing Interests Statement - References - Figure Legends - (Main Tables with legends if applicable) - Expanded View Figure Legends.

As a matter of course, please make sure that you have correctly followed the instructions for authors as given on the submission website.

Thank you for submitting this paper to Molecular Systems Biology.

Kind regards,
Jingyi

Jingyi Hou, PhD
Senior Editor
Molecular Systems Biology

*** PLEASE NOTE *** As part of the EMBO Press transparent editorial process initiative (see our Editorial at https://dx.doi.org/10.1038/msb.2010.72 , Molecular Systems Biology will publish online a Review Process File to accompany accepted manuscripts. When preparing your letter of response, please be aware that in the event of acceptance, your cover letter/point-by-point document will be included as part of this File, which will be available to the scientific community. More information about this initiative is available in our Instructions to Authors. If you have any questions about this initiative, please contact the editorial office (msb@embo.org).

Reviewer #2:

I appreciate the authors' thorough point-by-point response and the careful revisions. The revisions have addressed most of my previous concerns. However, I would recommend that authors consider revising their interpretations of their datasets before it becomes a staying record. Phenotypically (and perhaps even transcriptomically) these ten tumor types are too subtle to be distinguished which is totally fine and interesting. Using tranformations to categorize them into three groups may not be ideal. If the authors want to keep their analysis as it is, please include a direct PCA plot of all the transcriptomic data from their tumor samples for others to refer and interpret. Otherwise, this is a large amount of work that should be published.

Response to reviewer's comments (*MS# MSB-2025-13410R*):

Dear Editor and Reviewer,

We sincerely appreciate for your constructive feedback and insightful comments on our manuscript. We are particularly grateful for the opportunity to submit a revised version addressing your additional concerns. In response, we have now included the direct PCA plots of all ART-D phenotypic and transcriptomic data and thoroughly revised the manuscript. Below, we provide a point-by-point response to the extra concern. For your convenience, all changes in the revised text are **highlighted in red**.

We believe that these revisions adequately address your concerns and that the manuscript is now suitable for publication.

Reviewer #2:

I appreciate the authors' thorough point-by-point response and the careful revisions. The revisions have addressed most of my previous concerns. However, I would recommend that authors consider revising their interpretations of their datasets before it becomes a staying record. Phenotypically (and perhaps even transcriptomically) these ten tumor types are too subtle to be distinguished which is totally fine and interesting. Using tranformations to categorize them into three groups may not be ideal. If the authors want to keep their analysis as it is, please include a direct PCA plot of all the transcriptomic data from their tumor samples for others to refer and interpret. Otherwise, this is a large amount of work that should be published.

Response: Thank you very much for your constructive feedback and insightful comments. In the previous manuscript version, PCA results for ART-D transcriptomic data before and after batch effect correction were provided only in Appendix Fig. S3A. However, because this figure labeled only the sampling times (stages T1–T5), employed limited scaling of the PC1 and PC2 axes, and omitted specific genotype annotations, it failed to clearly distinguish between different *Ras*-driven tumor genotypes at the same stage. Although a general progression trend—from *wild-type* to benign *Ras* tumors and finally to malignant tumors (5.5–13.5 days AEL)—was discernible, the lack of resolution likely led to your concern that phenotypic and transcriptomic differences were "too subtle to be distinguished" and that "using transformations to categorize them into three groups may not be ideal."

To directly address these concerns, we have fully adopted your suggestions. We performed a new PCA using raw phenotypic and transcriptomic data, wherein batch effects were removed for each biological replicate, followed by $\log_2$(CPM+1) transformation and averaging. This analysis generated an interactive 3D space defined by PC1, PC2, and PC3 for both quantified malignancies and transcriptomic profiles **(provided as HTML files, Datasets EV1 and EV3)**, enabling readers to examine the distinct phenotypic and transcriptomic characteristics of various tumors within the ART-D framework. Additionally, we included **sequential views in the Appendix Figs. S1A and S3B**, depicting a clockwise rotation around the PC3 axis to reveal sample distributions from multiple angles. Using *Ras^{V12} scrib$^{-/-}$* and *Ras^{V12} TSG101$^{-/-}$* as representative examples, we delineated the spatial trajectories of malignant tumor progression within the PCA coordinate system, highlighted by orange and blue triangles, respectively.

Our results demonstrate that *Ras*-driven tumors in the ART-D model exhibit substantial spatial divergence across distinct genetic contexts, both phenotypically and transcriptomically. Notably, phenotypic PCA reveals that, while sampling time significantly influences the first three principal components (PC1–PC3, explaining >80% variance), it also drives a similar progression direction as indicated by arrows. Guided by these findings, we established a linear relationship between phenotypic progression and sampling time to examine the temporal evolution divergence in phenotypic malignancy across all *Ras*-driven tumors. We subsequently applied a robust linear regression model to fit tumor progression for each digitized phenotypic malignancy metric (six metrics total) within each DRT (ten tumor models total), using sampling time as the independent variable. Key parameters (slope, intercept, and R^2 values) were extracted, and PCA combined with K-means clustering were performed on these metrics to achieve the following phenotype-dependent tumor subtyping. Please refer to the revised manuscript on **page 7, lines 133–142**, and **page 9, lines 173-178, Datasets EV1 and EV3, Appendix Figs. S1A and S3B.**

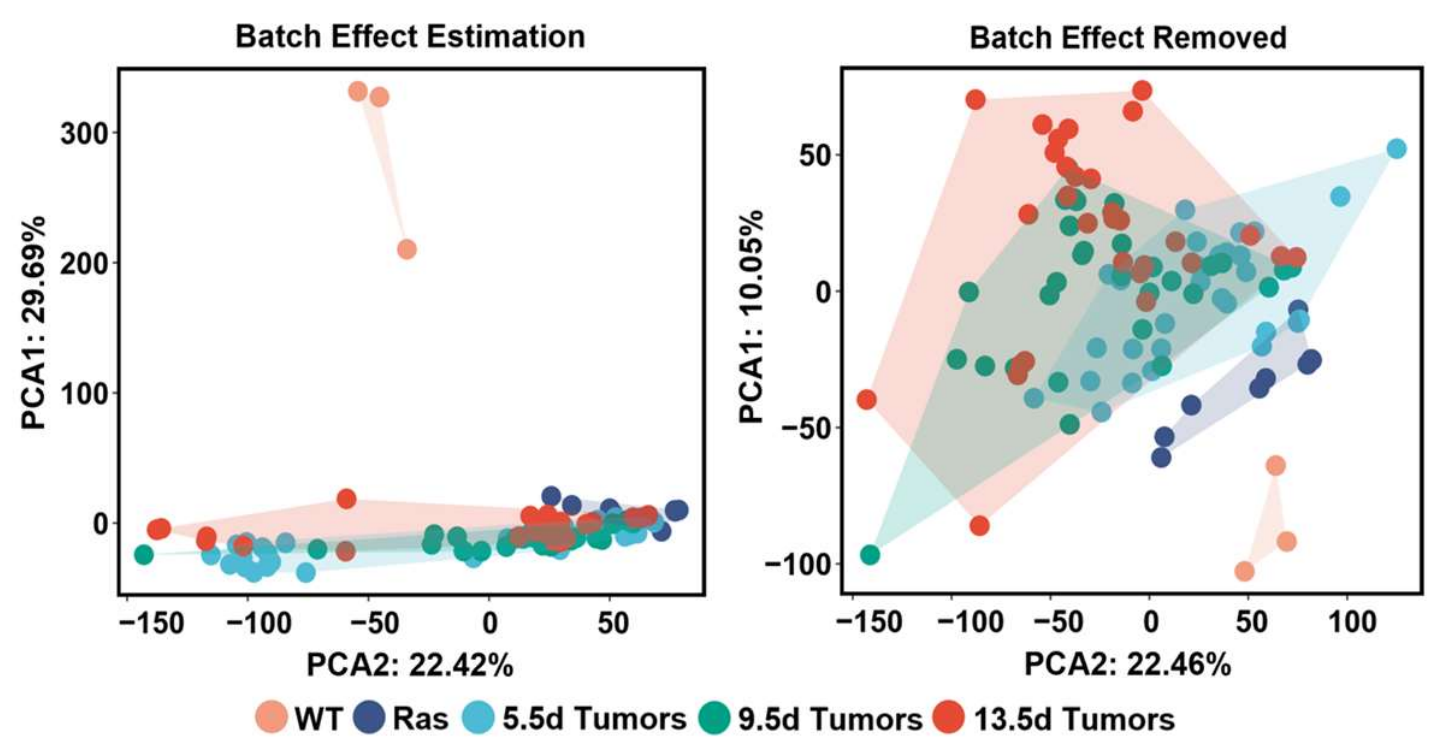

Appendix figure S3. The general transcriptomic dynamics of all *Drosophila Ras*-driven tumors.

(A) The PCA plot depicting the batch effect removal of bulk RNA-seq samples of all ART-D.

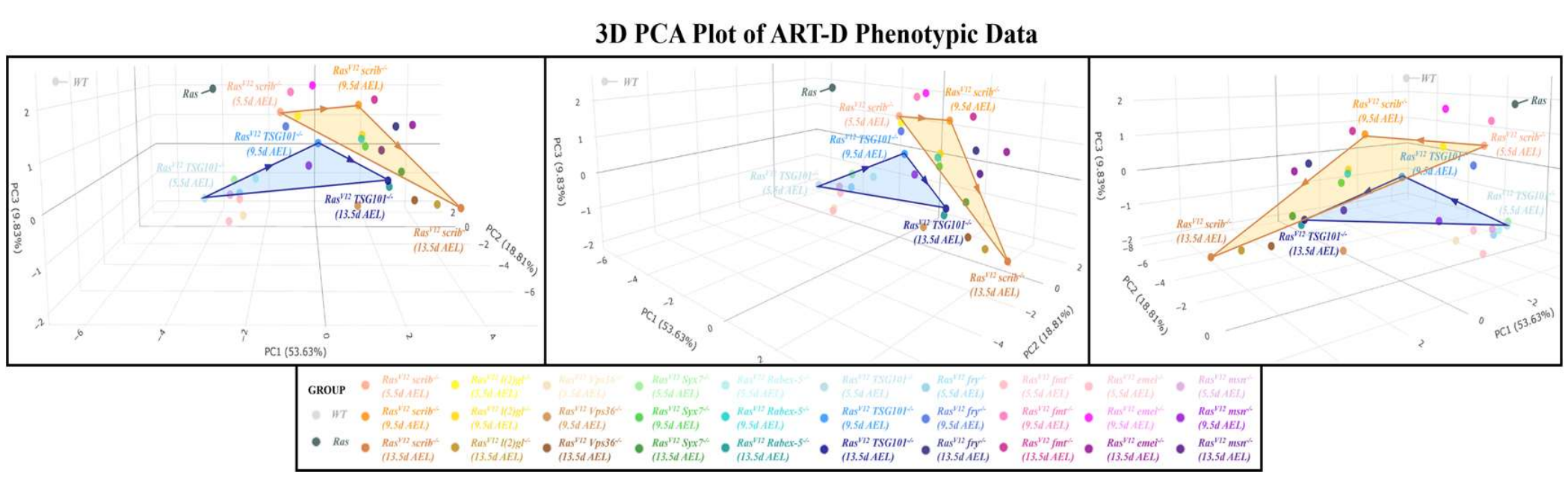

Appendix figure S1. The 3D PCA plot and robust linear regression model of tumor malignancies across all *Drosophila Ras*-driven tumors.

(A) The 3D PCA plot of ART-D phenotypic data. The plot illustrated the distribution of *WT*, *Ras*, and ten malignant tumors with distinct genotypes across three time points (5.5d, 9.5d, and 13.5d AEL) within a 3D space defined by PC1, PC2, and PC3 of quantified malignancies. Sequential views from left to right depicted a clockwise rotation around the PC3 axis to reveal sample distributions from various angles. Using *Ras*^{V12} *scrib*$^{-/-}$ and *Ras*^{V12} *TSG101*$^{-/-}$ as representative

examples, the spatial trajectories of malignant tumor progression were delineated within the PCA coordinate system, highlighted by orange and blue triangles, respectively. The arrows indicated the complete trajectory of malignant tumor progression.

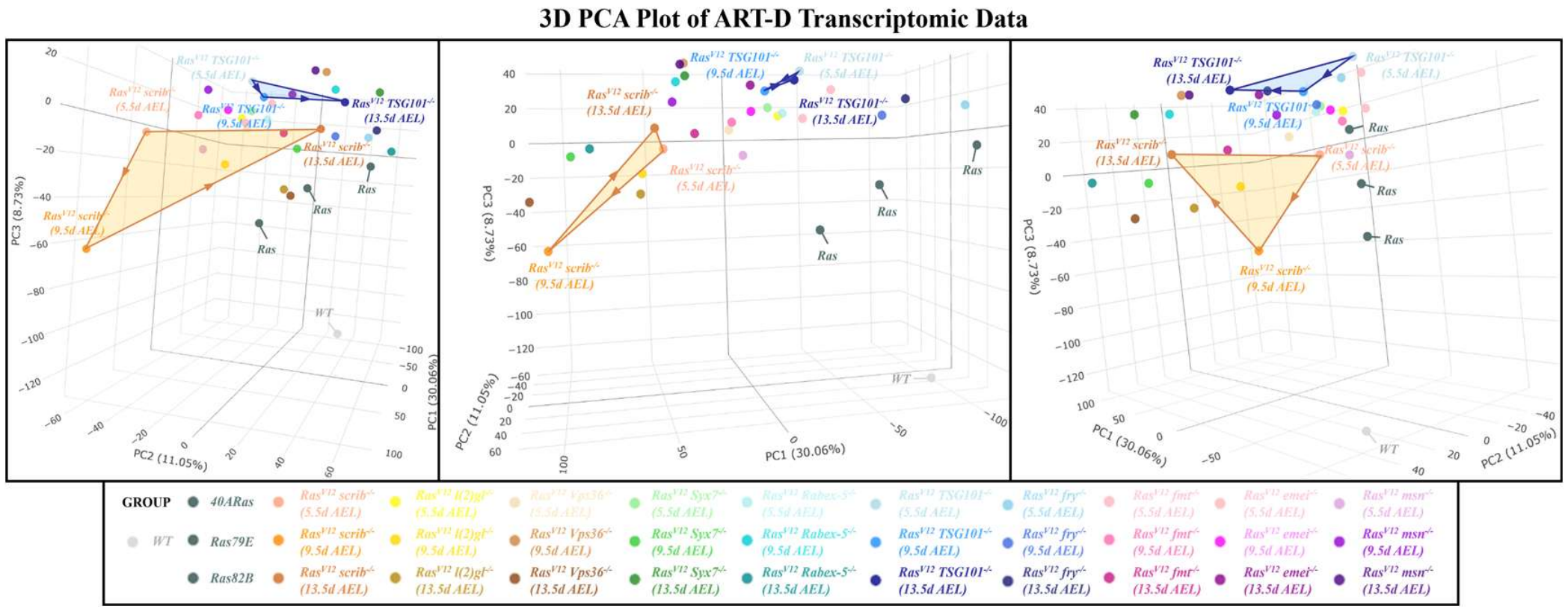

Appendix figure S3. The general transcriptomic dynamics of all *Drosophila Ras*-driven tumors.

(B) The 3D PCA plot of ART-D transcriptomic data. The plot illustrated the distribution of *WT*, *Ras*, and ten malignant tumors with distinct genotypes across three time points (5.5d, 9.5d, and 13.5d AEL) within a 3D space defined by PC1, PC2, and PC3. Data represent average log$_2$(CPM+1) transformed transcriptomic profiles following batch effect correction. Sequential views from left to right depicted a clockwise rotation around the PC3 axis to reveal sample distributions from various angles. Using *Ras*^{V12} *scrib*$^{-/-}$ and *Ras*^{V12} *TSG101*$^{-/-}$ as representative examples, the spatial trajectories of malignant tumor progression were delineated within the PCA coordinate system, highlighted by orange and blue triangles, respectively. The arrows indicated the complete transcriptomic trajectory of malignant tumor progression.

28th Apr 2026
Manuscript number: MSB-2025-13410RR
Title: Cross-Species Insights from ART-D to Uncover Evolutionarily Conserved Oncogenic Mechanisms

Dear Xianjue,

Thank you again for sending us your revised manuscript. We are now satisfied with the modifications made and I am pleased to inform you that your paper has been accepted for publication.

Your manuscript will be processed for publication by EMBO Press. It will be copy edited and you will receive page proofs prior to publication. Please note that you will be contacted by Springer Nature Author Services to complete licensing and payment information.

You may qualify for financial assistance for your publication charges - either via a Springer Nature fully open access agreement or an EMBO initiative. Check your eligibility: https://link.springer.com/journal/44320/how-to-publish-with-us

Should you be planning a Press Release on your article, please get in contact with embo_production@springernature.com as early as possible in order to coordinate publication and release dates.

If you have any questions, please do not hesitate to contact the Editorial Office. Thank you for your contribution to Molecular Systems Biology.

Sincerely,
Jingyi

Jingyi Hou, PhD
Senior Editor
Molecular Systems Biology

>>> Please note that it is Molecular Systems Biology policy for the transcript of the editorial process (containing referee reports and your response letter) to be published as an online supplement to each paper. If you do NOT want this, you will need to inform the Editorial Office via email immediately. More information is available here: https://link.springer.com/partners/embo-press/editorial-policies#Peer%20review

EMBO Press Author Checklist

Corresponding Author Name: Yifan Guo; Wenyan Xu; Xianping Wang; Xianjue Ma
Journal Submitted to: Molecular Systems Biology
Manuscript Number: #MSB-2025-13410

USEFUL LINKS FOR COMPLETING THIS FORM
The EMBO Journal - Author Guidelines
EMBO Reports - Author Guidelines
Molecular Systems Biology - Author Guidelines
EMBO Molecular Medicine - Author Guidelines

Reporting Checklist for Life Science Articles (updated January
This checklist is adapted from Materials Design Analysis Reporting (MDAR) Checklist for Authors. MDAR establishes a minimum set of requirements in transparent reporting in the life sciences (see Statement of Task: 10.31222/osf.io/9sm4x). Please follow the journal's guidelines in preparing your manuscript.
Please note that a copy of this checklist will be published alongside your article.

Abridged guidelines for figures
1. Data
The data shown in figures should satisfy the following conditions:
- the data were obtained and processed according to the field's best practice and are presented to reflect the results of the experiments in an accurate and unbiased manner.
- ideally, figure panels should include only measurements that are directly comparable to each other and obtained with the same assay.
- plots include clearly labeled error bars for independent experiments and sample sizes. Unless justified, error bars should not be shown for technical
- if n<5, the individual data points from each experiment should be plotted. Any statistical test employed should be justified.
- Source Data should be included to report the data underlying figures according to the guidelines set out in the authorship guidelines on Data

2. Captions
Each figure caption should contain the following information, for each panel where they are relevant:
- a specification of the experimental system investigated (eg cell line, species name).
- the assay(s) and method(s) used to carry out the reported observations and measurements.
- an explicit mention of the biological and chemical entity(ies) that are being measured.
- an explicit mention of the biological and chemical entity(ies) that are altered/varied/perturbed in a controlled manner.
- the exact sample size (n) for each experimental group/condition, given as a number, not a range;
- a description of the sample collection allowing the reader to understand whether the samples represent technical or biological replicates (including how many animals, litters, cultures, etc.).
- a statement of how many times the experiment shown was independently replicated in the laboratory.
- definitions of statistical methods and measures:
  - common tests, such as t-test (please specify whether paired vs. unpaired), simple χ2 tests, Wilcoxon and Mann-Whitney tests, can be unambiguously identified by name only, but more complex techniques should be described in the methods section;
  - are tests one-sided or two-sided?
  - are there adjustments for multiple comparisons?
  - exact statistical test results, e.g., P values = x but not P values < x;
  - definition of 'center values' as median or average;
  - definition of error bars as s.d. or s.e.m.

Please complete ALL of the questions below.
Select "Not Applicable" only when the requested information is not relevant for your study.

Materials

Newly Created Materials	Information included in the manuscript?	In which section is the information available? (Reagents and Tools Table, Materials and Methods, Figures, Data Availability Section)
New materials and reagents need to be available; do any restrictions apply?	Yes	Reagents and Tools Table

Antibodies	Information included in the manuscript?	In which section is the information available? (Reagents and Tools Table, Materials and Methods, Figures, Data Availability Section)
For **antibodies** provide the following information:<br>- Commercial antibodies: RRID (if possible) or supplier name, catalogue number and or/clone number<br>- Non-commercial: RRID or citation	Yes	Reagents and Tools Table

DNA and RNA sequences	Information included in the manuscript?	In which section is the information available? (Reagents and Tools Table, Materials and Methods, Figures, Data Availability Section)
**Short novel DNA or RNA including primers, probes**: provide the sequences.	Yes	Reagents and Tools Table

Cell materials	Information included in the manuscript?	In which section is the information available? (Reagents and Tools Table, Materials and Methods, Figures, Data Availability Section)
**Cell lines:** Provide species information, strain. Provide accession number in repository **OR** supplier name, catalog number, clone number, and/**OR** RRID.	Not Applicable	
**Primary cultures:** Provide species, strain, sex of origin, genetic modification status.	Not Applicable	
Report if the cell lines were recently **authenticated** (e.g., by STR profiling) and tested for mycoplasma contamination.	Not Applicable	

Experimental animals	Information included in the manuscript?	In which section is the information available? (Reagents and Tools Table, Materials and Methods, Figures, Data Availability Section)
**Laboratory animals or Model organisms:** Provide species, strain, sex, age, genetic modification status. Provide accession number in repository **OR** supplier name, catalog number, clone number, **OR** RRID.	Yes	Reagents and Tools Table, Materials and Methods
**Animal observed in or captured from the field:** Provide species, sex, and age where possible.	Not Applicable	
Please detail **housing and husbandry conditions**.	Yes	Materials and Methods

Plants and microbes	Information included in the manuscript?	In which section is the information available? (Reagents and Tools Table, Materials and Methods, Figures, Data Availability Section)
**Plants:** provide species and strain, ecotype and cultivar where relevant, unique accession number if available, and source (including location for collected wild specimens).	Not Applicable	
**Microbes:** provide species and strain, unique accession number if available, and source.	Not Applicable	

Human research participants	Information included in the manuscript?	In which section is the information available? (Reagents and Tools Table, Materials and Methods, Figures, Data Availability Section)
If collected and within the bounds of privacy constraints report on age, sex and gender or ethnicity for all study participants.	Not Applicable	

Core facilities	Information included in the manuscript?	In which section is the information available? (Reagents and Tools Table, Materials and Methods, Figures, Data Availability Section)
If your work benefited from core facilities, was their service mentioned in the acknowledgments section?	Yes	Materials and Methods and Acknowledgements

Design

Study protocol	Information included in the manuscript?	In which section is the information available? (Reagents and Tools Table, Materials and Methods, Figures, Data Availability Section)
If study protocol has been **pre-registered, provide DOI in the manuscript**. For clinical trials, provide the trial registration number **OR** cite DOI.	Not Applicable	
Report the **clinical trial registration number** (at ClinicalTrials.gov or equivalent), where applicable.	Not Applicable	

Laboratory protocol	Information included in the manuscript?	In which section is the information available? (Reagents and Tools Table, Materials and Methods, Figures, Data Availability Section)
Provide DOI OR other citation details if **external detailed step-by-step protocols** are available.	Not Applicable	

Experimental study design and statistics	Information included in the manuscript?	In which section is the information available? (Reagents and Tools Table, Materials and Methods, Figures, Data Availability Section)
Include a statement about **sample size** estimate even if no statistical methods were used.	Yes	Figures
Were any steps taken to minimize the effects of subjective bias when allocating animals/samples to treatment (e.g. **randomization procedure**)? If yes, have they been described?	Yes	Materials and Methods
Include a statement about **blinding** even if no blinding was done.	Not Applicable	
Describe **inclusion/exclusion criteria** if samples or animals were excluded from the analysis. Were the criteria pre-established?<br><br>If sample or data points were omitted from analysis, report if this was due to attrition or intentional exclusion and provide justification.	Not Applicable	
For every figure, are **statistical tests** justified as appropriate? Do the data meet the assumptions of the tests (e.g., normal distribution)? Describe any methods used to assess it. Is there an estimate of variation within each group of data? Is the variance similar between the groups that are being statistically compared?	Yes	Materials and Methods, Figure legends

Sample definition and in-laboratory replication	Information included in the manuscript?	In which section is the information available? (Reagents and Tools Table, Materials and Methods, Figures, Data Availability Section)
In the figure legends: state number of times the experiment was **replicated** in laboratory.	Yes	Materials and Methods, Figure legends
In the figure legends: define whether data describe **technical or biological replicates**.	Yes	Materials and Methods, Figure legends

Ethics

Ethics	Information included in the manuscript?	In which section is the information available? (Reagents and Tools Table, Materials and Methods, Figures, Data Availability Section)
Studies involving **human participants**: State details of **authority granting ethics approval** (IRB or equivalent committee(s), provide reference number for approval.	Not Applicable	
Studies involving **human participants**: Include a statement confirming that **informed consent** was obtained from all subjects and that the experiments conformed to the principles set out in the WMA Declaration of Helsinki and the Department of Health and Human Services Belmont Report.	Not Applicable	
Studies involving **human participants:** For publication of **patient photos**, include a statement confirming that consent to publish was obtained.	Not Applicable	
Studies involving experimental **animals**: State details of **authority granting ethics approva**l (IRB or equivalent committee(s), provide reference number for approval. Include a statement of compliance with ethical regulations.	Not Applicable	
Studies involving **specimen and field samples:** State if relevant **permits** obtained, provide details of authority approving study; if none were required, explain why.	Not Applicable	

Dual Use Research of Concern (DURC)	Information included in the manuscript?	In which section is the information available? (Reagents and Tools Table, Materials and Methods, Figures, Data Availability Section)
Could your study fall under dual use research restrictions? Please check biosecurity documents and list of **select agents and toxins** (CDC): https://www.selectagents.gov/sat/list.htm	Not Applicable	
If you used a select agent, is the security level of the lab appropriate and reported in the manuscript?	Not Applicable	
If a study is subject to dual use research of concern regulations, is the name of the **authority granting approval and reference number** for the regulatory approval provided in the manuscript?	Not Applicable	

Reporting

The MDAR framework recommends adoption of discipline-specific guidelines, established and endorsed through community initiatives. Journals have their own policy about requiring specific guidelines and recommendations to complement MDAR.

Adherence to community standards	Information included in the manuscript?	In which section is the information available? (Reagents and Tools Table, Materials and Methods, Figures, Data Availability Section)
State if relevant guidelines or checklists (e.g., **ICMJE, MIBBI, ARRIVE, PRISMA**) have been followed or provided.	Not Applicable	
For **tumor marker prognostic studies,** we recommend that you follow the **REMARK** reporting guidelines (see link list at top right). See author guidelines, under 'Reporting Guidelines'. Please confirm you have followed these guidelines.	Not Applicable	
For **phase II and III randomized controlled trials**, please refer to the **CONSORT** flow diagram (see link list at top right) and submit the CONSORT checklist (see link list at top right) with your submission. See author guidelines, under 'Reporting Guidelines'. Please confirm you have submitted this list.	Not Applicable	

Data Availability

Data availability	Information included in the manuscript?	In which section is the information available? (Reagents and Tools Table, Materials and Methods, Figures, Data Availability Section)
Have **primary datasets** been deposited according to the journal's guidelines (see 'Data Deposition' section) and the respective accession numbers provided in the Data Availability Section?	Yes	Reagents and Tools Table, Materials and Methods
Were **human clinical and genomic datasets** deposited in a public access-controlled repository in accordance to ethical obligations to the patients and to the applicable consent agreement?	Not Applicable	
Are **computational models** that are central and integral to a study available without restrictions in a machine-readable form? Were the relevant accession numbers or links provided?	Not Applicable	
If publicly available data were reused, provide the respective **data citations in the reference list**.	Yes	Materials and Methods, References
