# Supplementary material for: Cross-species insights from ART-D to uncover evolutionarily conserved oncogenic mechanisms
Source: Mol Syst Biol. 2026 May 14;22(8):1214–51. doi: 10.1038/s44320-026-00215-8 (PMC13433787; doi:10.1038/s44320-026-00215-8)
Supplement: Supplementary file 3 [file 44320_2026_215_MOESM2_ESM.pdf]

Cross-Species Insights from ART-D to Uncover Evolutionarily Conserved Oncogenic Mechanisms
(Appendix File)

Table of Contents

Appendix figure S1. The 3D PCA plot and robust linear regression model of tumor malignancies across all *Drosophila Ras*-driven tumors....2
Appendix figure S2. The robust linear regression model of tumor malignancies in different *Drosophila Ras*-driven tumors....4
Appendix figure S3. The general transcriptomic dynamics of all *Drosophila Ras*-driven tumors. ..6
Appendix figure S4. Activation status of core signaling pathways in *Drosophila Ras*-driven tumors from T1 to T5....7
Appendix figure S5. The activation of MAPK signaling pathways at the initial stage of *Drosophila Ras*-driven tumors....8
Appendix figure S6. The activation of NFκB/Imd and NFκB/Toll signaling pathways at the initial stage of *Drosophila Ras*-driven tumors....10
Appendix figure S7. The Notch activation and Hippo inactivation across different *Drosophila Ras*-driven tumors....11
Appendix figure S8. The tumor promoting roles of Notch activation and Hippo inactivation across different *Drosophila Ras*-driven tumors....12
Appendix figure S9. Data characteristics and signaling pathways identified as responsible for phenotypes during Ras^{V12}; $fry^{-/-}$ tumor progression....14
Appendix figure S10. Evaluation of physiological and pathological functions of *fry*....16
Appendix figure S11. Expression levels of human orthologs across different human *KRAS/NRAS*-driven cancers....18
Appendix figure S12. Annotation of cell types in different human *KRAS/NRAS*-driven cancers...19
Appendix figure S13. Scoring of evolutionarily conserved oncogenic signaling pathways in human *KRAS/NRAS-driven* cancers....20
Appendix figure S14. Correlations of evolutionarily conserved oncogenic signaling pathways in human *KRAS/NRAS-driven* cancers....21
Appendix figure S15. Correlations of *fry*/*FRY*-deficient tumor signature and tumor malignancies in *RAS*-activated cells of human *KRAS/NRAS-driven* cancers....23
Appendix figure S16. Effects of *FRY* in immune infiltration across different human *KRAS/NRAS-driven* cancers....25
Appendix figure S17. The predictive value of *Drosophila Ras*-driven tumors examined by 117 machine learning algorithms in COAD....26
Appendix figure S18. The predictive value of *Drosophila Ras*-driven tumors examined by 117 machine learning algorithms in LUAD....28
Appendix figure S19. The predictive value of *Drosophila Ras*-driven tumors examined by 117 machine learning algorithms in PAAD....30
Appendix figure S20. The predictive value of *Drosophila Ras*-driven tumors examined by 117 machine learning algorithms in SKCM....32

Appendix Fig S1.

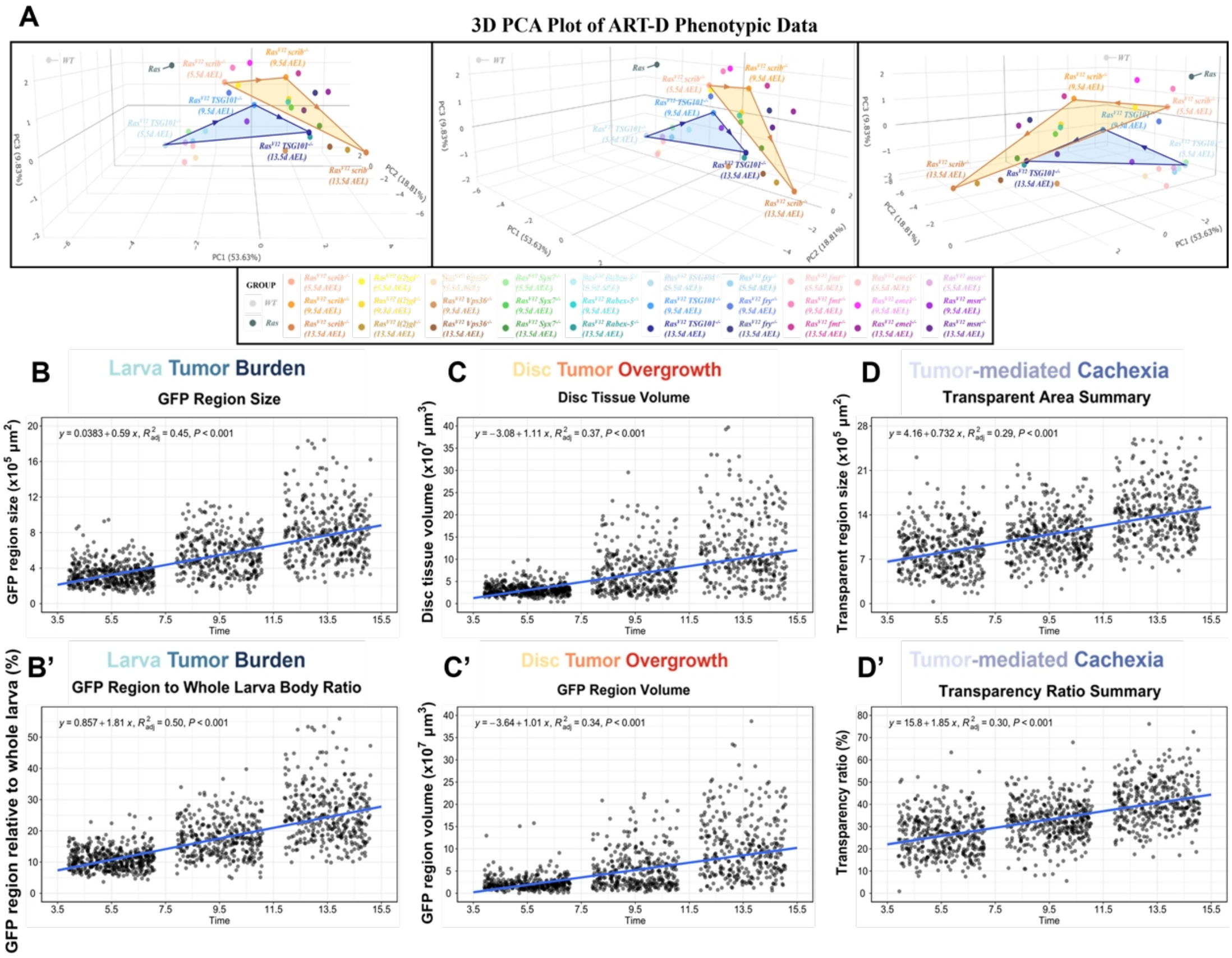

Appendix figure S1. The 3D PCA plot and robust linear regression model of tumor malignancies across all *Drosophila Ras*-driven tumors.

(A) The 3D PCA plot of ART-D phenotypic data. The plot illustrated the distribution of *WT*, *Ras*, and ten malignant tumors with distinct genotypes across three time points (5.5d, 9.5d, and 13.5d AEL) within a 3D space defined by PC1, PC2, and PC3 of quantified malignancies. Sequential views from left to right depicted a clockwise rotation around the PC3 axis to reveal sample distributions from various angles. Using Ras^{V12} $scrib^{-/-}$ and Ras^{V12} $TSG101^{-/-}$ as representative examples, the spatial trajectories of malignant tumor progression were delineated within the PCA coordinate system, highlighted by orange and blue triangles, respectively. The arrows indicated the complete trajectory of malignant tumor progression.

(B-B') The robust linear regression model established in parameters reflecting larva tumor burden across all *Drosophila Ras*-driven tumors. Each dot reflected one sampling point for a given parameter. The equations of GFP region size (B) and GFP region to whole larva body ratio (B') were denoted at the top left corner of each figure. The adjusted R^2 and *P* values were calculated by using M-Estimators.

(C-C') The robust linear regression model established in parameters reflecting disc tumor overgrowth across all *Drosophila Ras*-driven tumors. Each dot reflected one sampling point for a given parameter. The equations of disc tissue volume (C) and GFP region volume (C') were denoted at the top left corner of each figure. The adjusted R^2 and *P* values were calculated by using M-Estimators.

(D-D') The robust linear regression model established in parameters reflecting tumor-mediated

cachexia across all *Drosophila Ras*-driven tumors. Each dot reflected one sampling point for a given parameter. The equations of transparent area summary (D) and transparency ratio summary (D') were denoted at the top left corner of each figure. The adjusted R^2 and *P* values were calculated by using M-Estimators.

Appendix Fig S2.

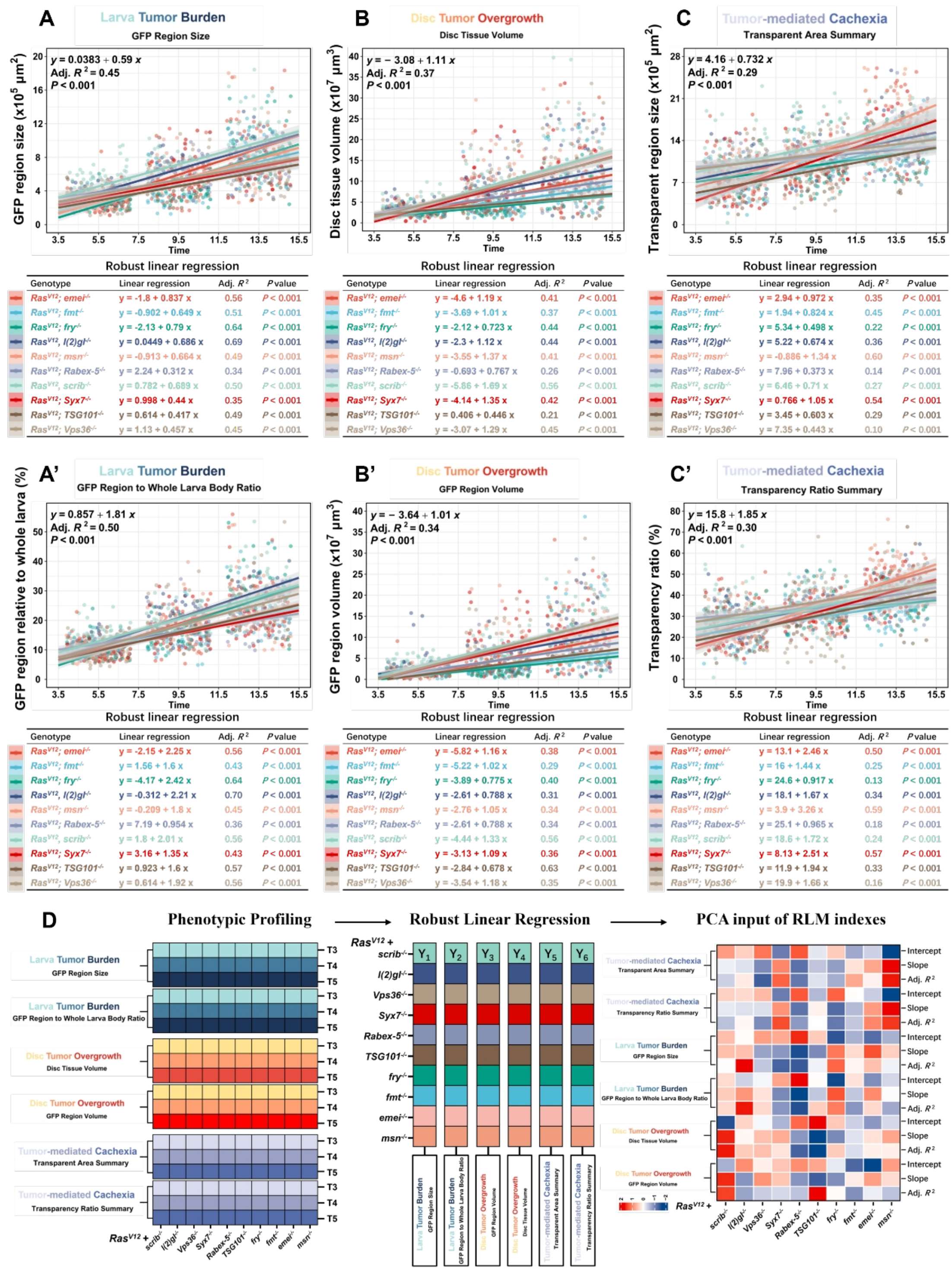

Appendix figure S2. The robust linear regression model of tumor malignancies in different *Drosophila Ras*-driven tumors.

(A-A') The robust linear regression models established in parameters reflecting larva tumor burden in different *Drosophila Ras*-driven tumors. The parameters encompassed GFP region size (A) and GFP region to whole larva body ratio (A'). Each dot reflected one sampling point for a certain parameter, different color indicated different *Drosophila* tumor models. The equations of all *Drosophila Ras*-driven tumors were denoted at the top left corner of each figure, while the equations

of each *Ras*-driven tumor model were shown in the table below. The adjusted R^2 and P values were calculated by using M-Estimators.

(B-B') The robust linear regression models established in parameters reflecting disc tumor overgrowth in different *Drosophila Ras*-driven tumors. The parameters encompassed disc tissue volume (B) and GFP region volume (B'). Each dot reflected one sampling point for a certain parameter, different color indicated different *Drosophila* tumor models. The equations of all *Drosophila Ras*-driven tumors were denoted at the top left corner of each figure, while the equations of each *Ras*-driven tumor model were shown in the table below. The adjusted R^2 and P values were calculated by using M-Estimators.

(C-C') The robust linear regression models established in parameters reflecting tumor-mediated cachexia in different *Drosophila Ras*-driven tumors. The parameters encompassed transparent area summary (C) and transparency ratio summary (C'). Each dot reflected one sampling point for a certain parameter, different color indicated different *Drosophila* tumor models. The equations of all *Drosophila Ras*-driven tumors were denoted at the top left corner of each figure, while the equations of each *Ras*-driven tumor model were shown in the table below. The adjusted R^2 and P values were calculated by using M-Estimators.

(D) Schematic diagram illustrating principal component analysis (PCA) performed on robust regression models constructed from phenotypic data across various DRTs. Using the intercept, slope, and adjusted R-squared values of these models as the input matrix, the further PCA analysis was conducted in conjunction with K-means clustering. This approach facilitates the identification of subtypes based on malignant progression phenotypes while preserving a quantitative focus on phenotypic dynamics.

Appendix Fig S3.

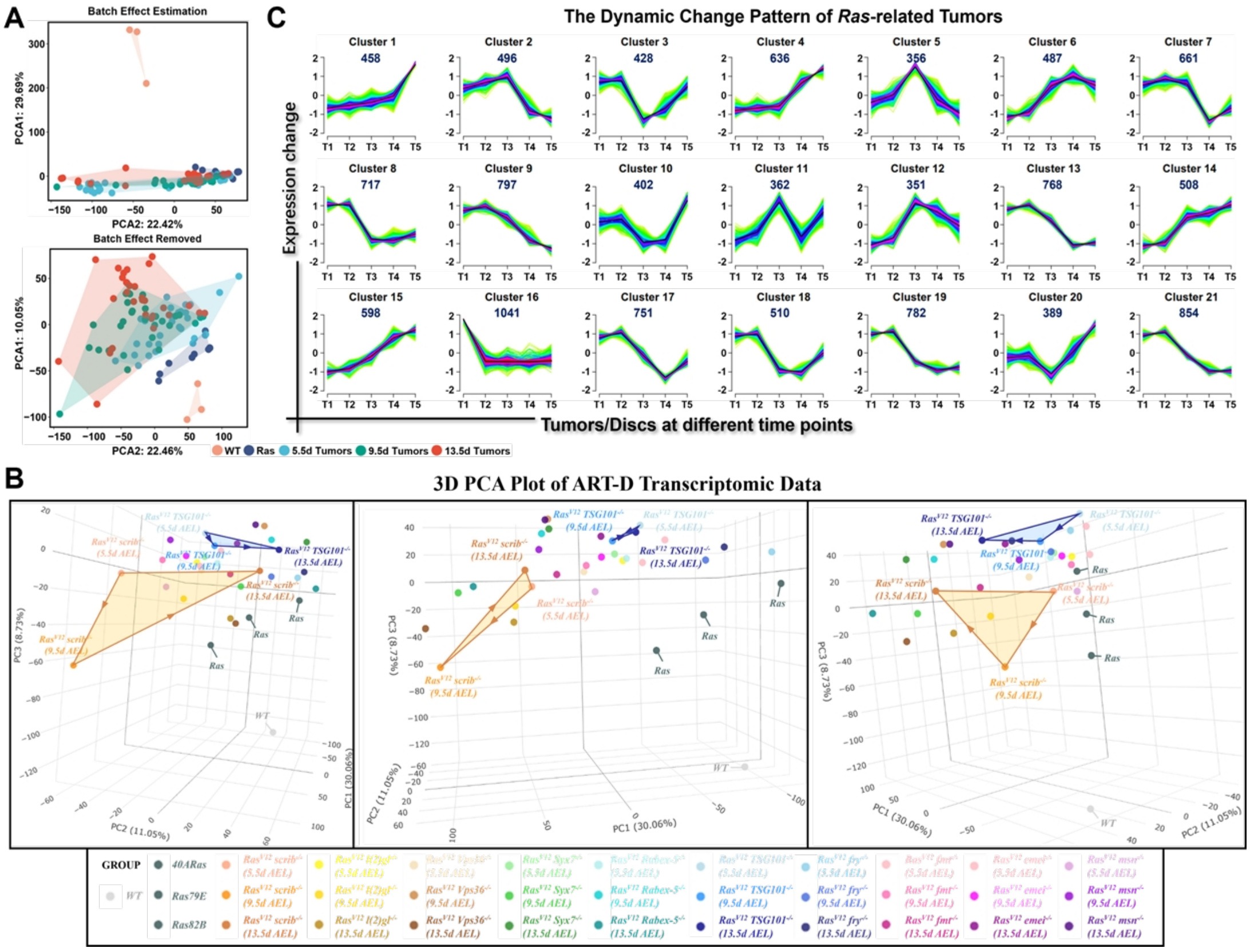

Appendix figure S3. The general transcriptomic dynamics of all *Drosophila Ras*-driven tumors.

(A) The PCA plot depicting the batch effect removal of bulk RNA-seq samples of all ART-D.

(B) The 3D PCA plot of ART-D transcriptomic data. The plot illustrated the distribution of *WT*, *Ras*, and ten malignant tumors with distinct genotypes across three time points (5.5d, 9.5d, and 13.5d AEL) within a 3D space defined by PC1, PC2, and PC3. Data represent average $\log_2$(CPM+1) transformed transcriptomic profiles following batch effect correction. Sequential views from left to right depicted a clockwise rotation around the PC3 axis to reveal sample distributions from various angles. Using Ras^{V12} $scrib^{-/-}$ and Ras^{V12} $TSG101^{-/-}$ as representative examples, the spatial trajectories of malignant tumor progression were delineated within the PCA coordinate system, highlighted by orange and blue triangles, respectively. The arrows indicated the complete transcriptomic trajectory of malignant tumor progression.

(C) The dynamic transcriptomic change patterns of all genes from *Drosophila Ras*-driven tumors. The transcriptomic dynamics were revealed by using Mfuzz with a threshold of 0.2 membership.

Appendix Fig S4.

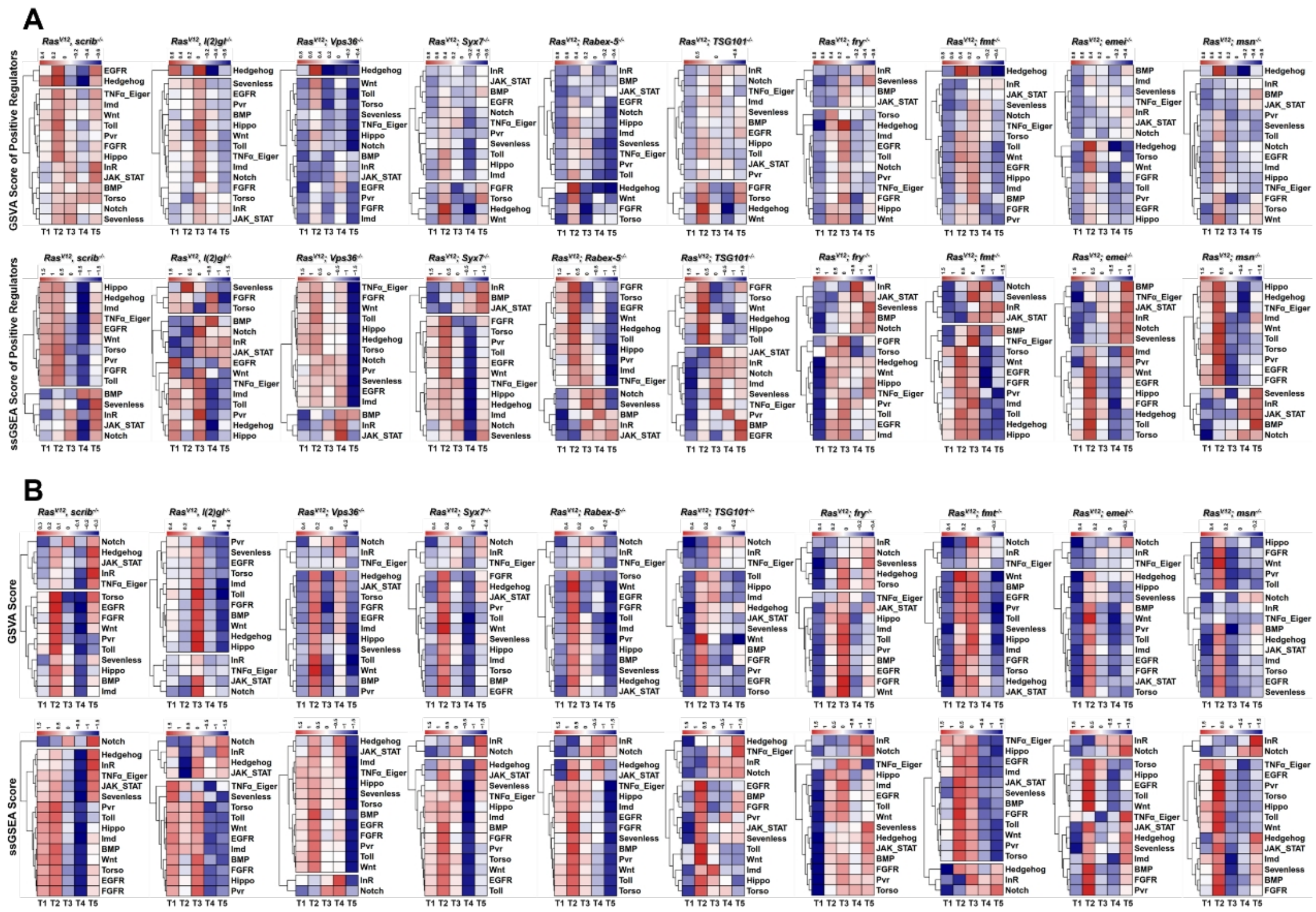

Appendix figure S4. Activation status of core signaling pathways in *Drosophila Ras*-driven tumors from T1 to T5.

(A) The heatmaps depicting the GSVA scores (Top) and the ssGSEA scores (Bottom) for 15 signaling pathways' positive regulators across various malignant tumors at different stages (from T1 to T5). The scores were normalized by row using Z-score scaling to highlight the most activated state of each signaling pathway. Pathway gene list was downloaded from Flybase.

(B) The heatmaps illustrate the GSVA scores (Top) and the ssGSEA scores (Bottom) for 15 signaling pathways across various malignant tumors at different stages (from T1 to T5). The scores were normalized by row using Z-score scaling to highlight the most activated state of each signaling pathway. Pathway gene lists were downloaded from Flybase.

Appendix Fig S5.

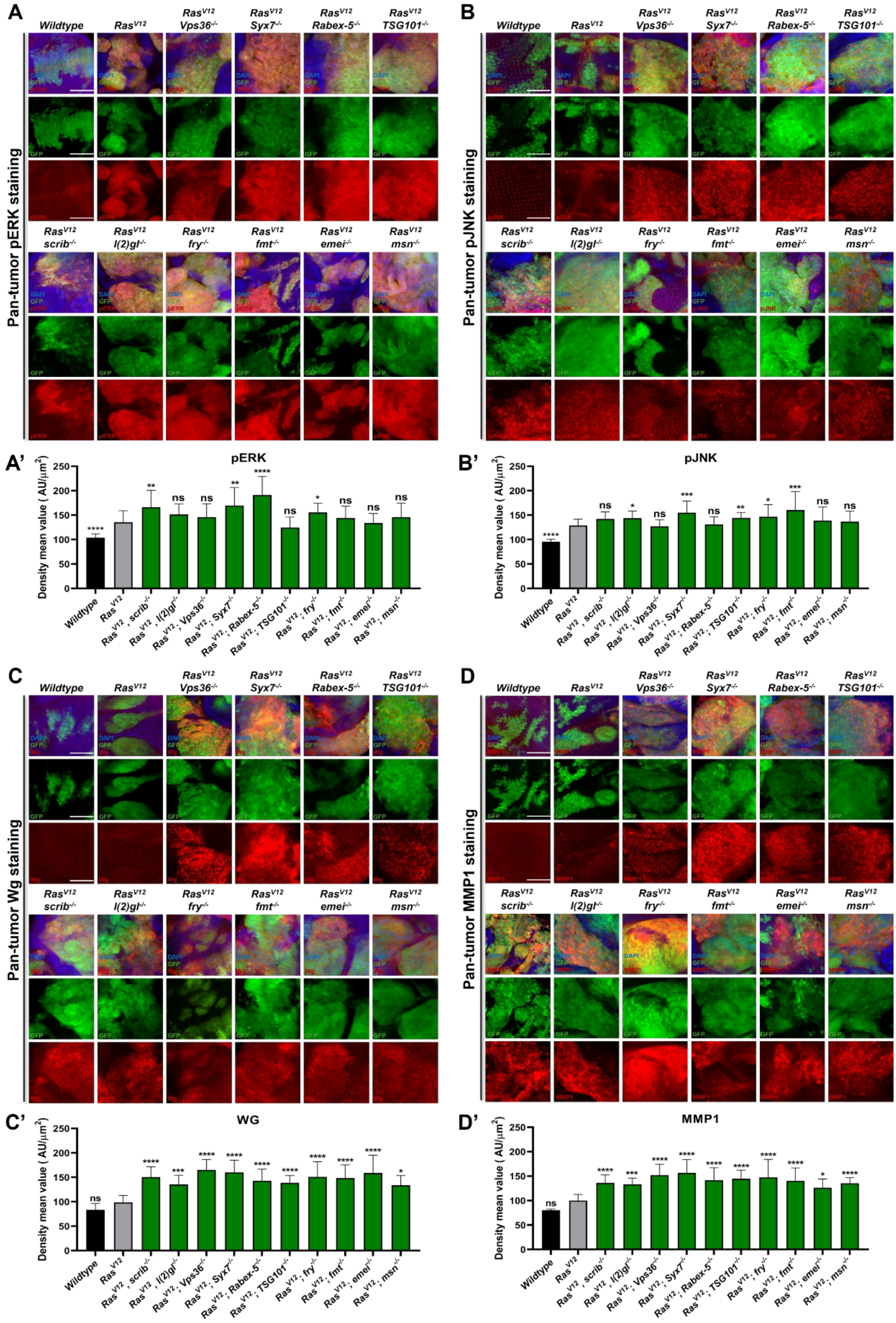

Appendix figure S5. The activation of MAPK signaling pathways at the initial stage of *Drosophila Ras*-driven tumors.

(A-D) Confocal images of eye-antennal discs or tumors harvested at 5.5-6.5 days AEL. Discs or tumors with indicated genotypes were stained with antibodies against pERK (A), pJNK (B), Wg

(C), and MMP1 (D). Scale bars: 50 μm.

(A'-D') Quantification results of immunofluorescence imaging of pERK (A'), pJNK (B'), Wg (C'), and MMP1 (D'). Kruskal-Wallis test was performed along with Dunn's multiple comparisons test against the *Ras* group, n >20. Results were presented as means ± SD. Significance of different genotypes were denoted as follows: ns > 0.05, *P < 0.05, **P < 0.01, ***P < 0.001, ****P < 0.0001.

Appendix Fig S6.

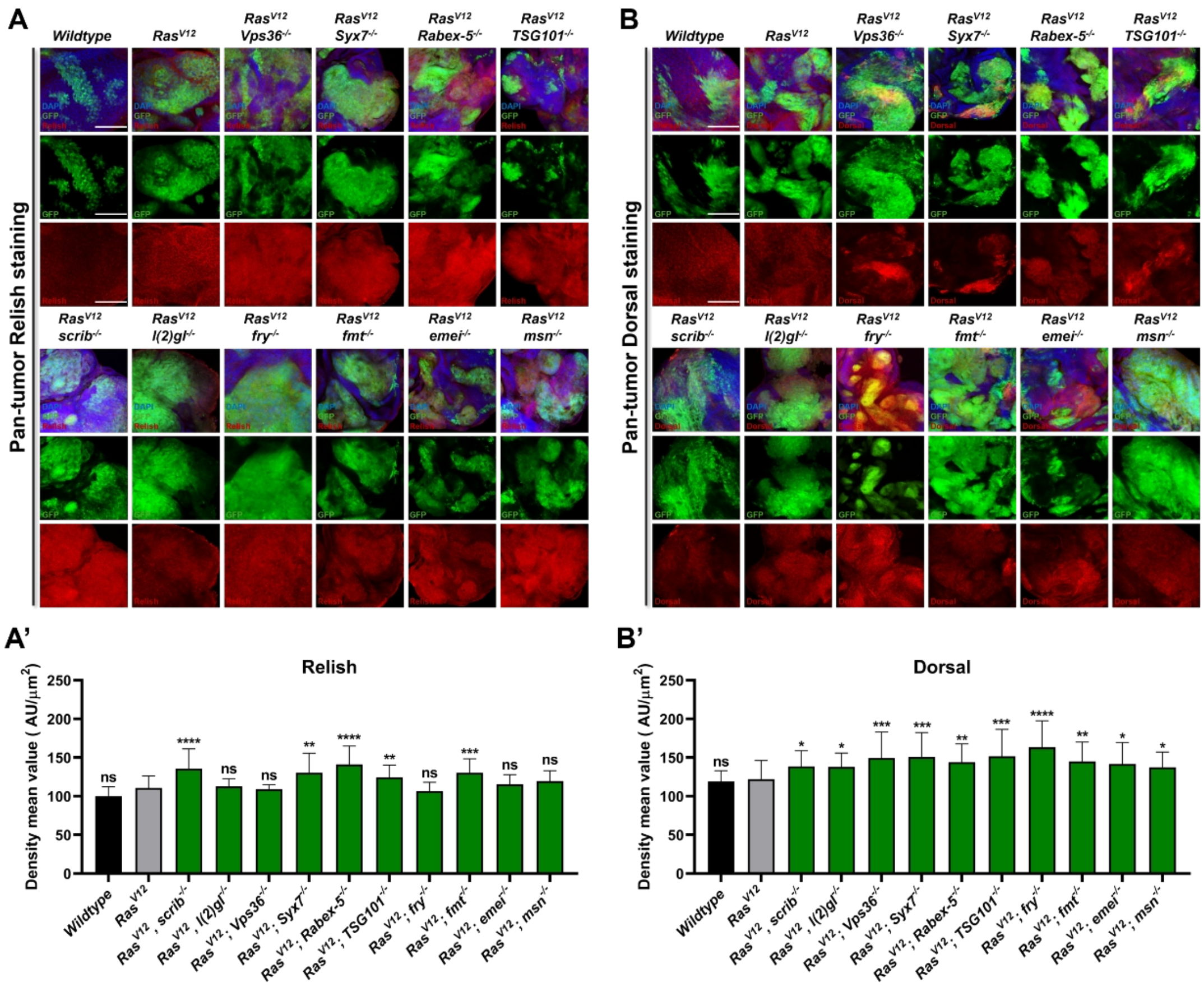

Appendix figure S6. The activation of NFκB/Imd and NFκB/Toll signaling pathways at the initial stage of *Drosophila Ras*-driven tumors.

(A-B) Confocal images of eye-antennal discs or tumors harvested at 5.5-6.5 days AEL. Discs or tumors with indicated genotypes were stained with antibodies against Relish (A) and Dorsal (B). Scale bars: 50 μm.

(A'-B') Quantification results of immunofluorescence imaging of Relish (A') and Dorsal (B'). Kruskal-Wallis test was performed along with Dunn's multiple comparisons test against the *Ras* group, n >20. Results were presented as means ± SD. Significance of different genotypes were denoted as follows: ns > 0.05, *P < 0.05, **P < 0.01, ***P < 0.001, ****P < 0.0001.

Appendix Fig S7.

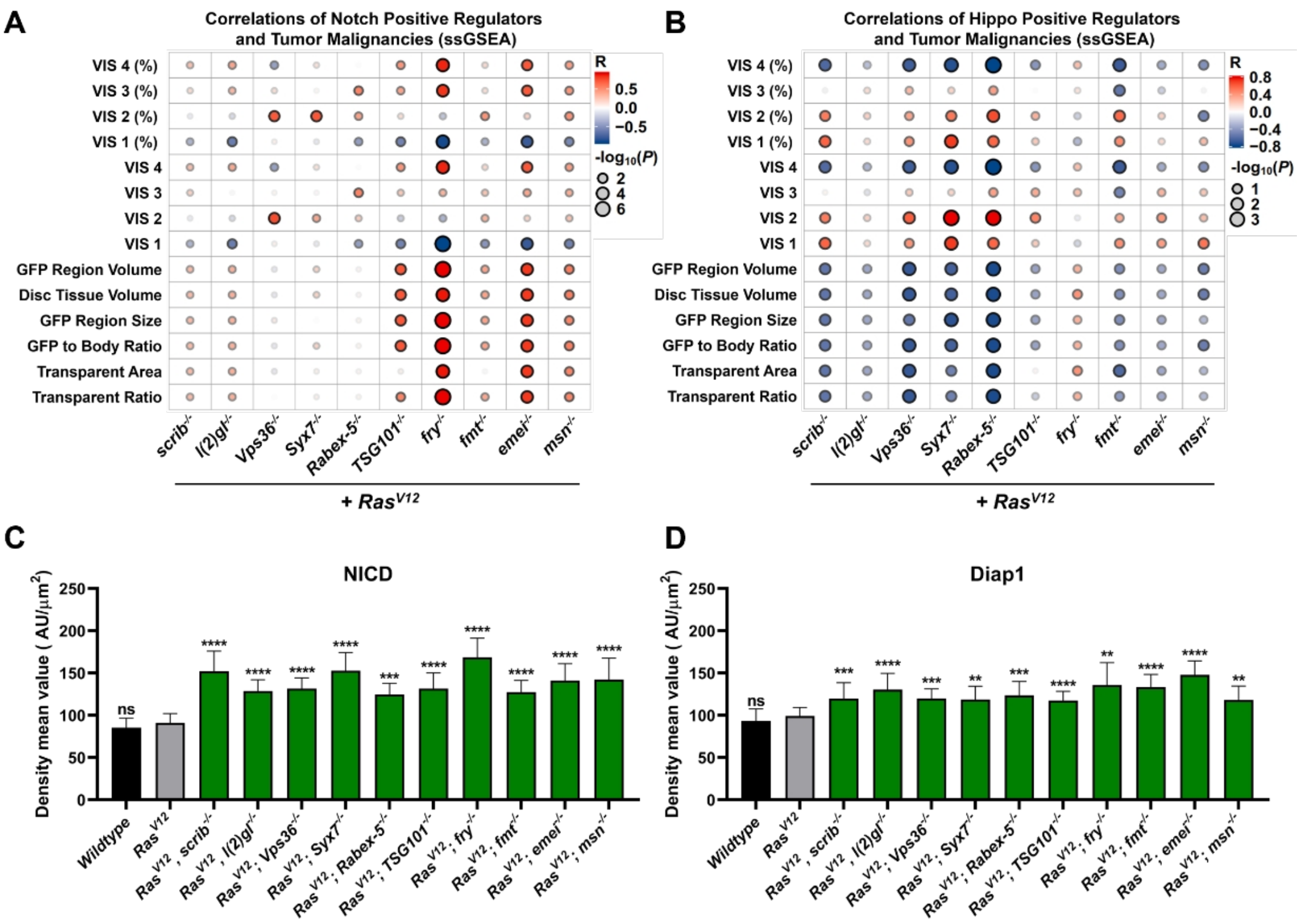

Appendix figure S7. The Notch activation and Hippo inactivation across different *Drosophila Ras*-driven tumors.

(A) The dot plots illustrate the correlations between positive regulators of Notch signaling pathway scores and the tumor malignancies. The signaling scores across different *Drosophila Ras*-driven tumors were evaluated by ssGSEA. The correlations were evaluated by Pearson correlation test. The color of the dots indicated the Pearson correlation coefficients (R), while the size of the dots reflected the negative log10-transformed *P* values.

(B) The dot plots illustrate the correlations between positive regulators of Hippo signaling pathway scores and the tumor malignancies. The signaling scores across different *Drosophila Ras*-driven tumors were evaluated by ssGSEA. The correlations were evaluated by Pearson correlation test. The color of the dots indicated the Pearson correlation coefficients (R), while the size of the dots reflected the negative log10-transformed *P* values.

(C-D) Quantification results of immunofluorescence imaging of NICD (C) and Diap1 (D). Kruskal-Wallis test was performed along with Dunn's multiple comparisons test against the *Ras* group, n >20. Results were presented as means ± SD. Significance of different genotypes were denoted as follows: ns > 0.05, **P* < 0.05, ***P* < 0.01, ****P* < 0.001, *****P* < 0.0001.

Appendix Fig S8.

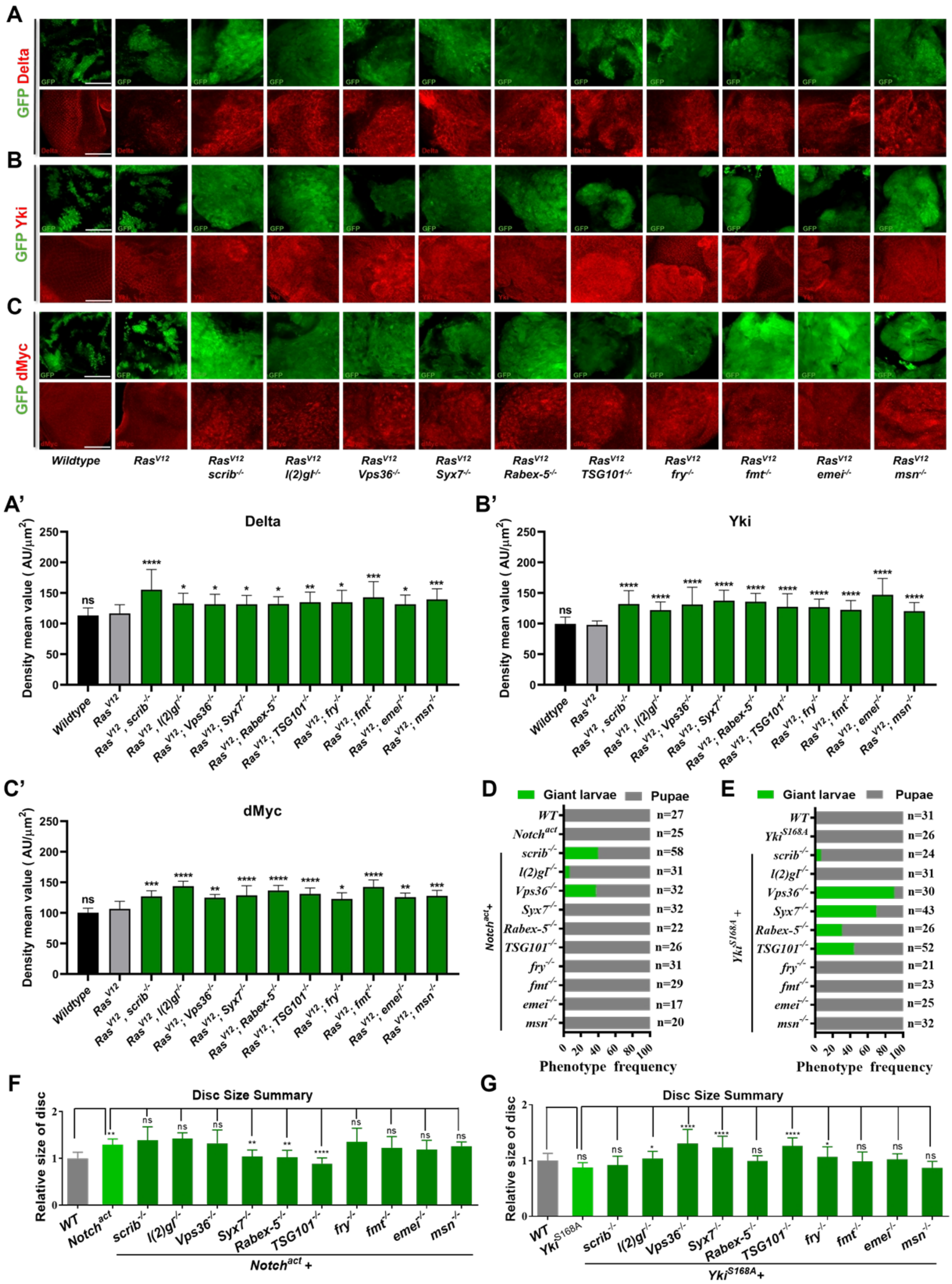

Appendix figure S8. The tumor promoting roles of Notch activation and Hippo inactivation across different *Drosophila Ras*-driven tumors.

(A-C) Confocal images of eye-antennal discs or tumors harvested at 5.5-6.5 days AEL. Discs or tumors with indicated genotypes were stained with antibodies against Delta (A), Yki (B) and dMyc (C). Scale bars: 50 μm.

(A'-C') Quantification results of immunofluorescence imaging of Delta (A'), Yki (B') and dMyc (C'). Kruskal-Wallis test was performed along with Dunn's multiple comparisons test against the *Ras* group, n >20. Results were presented as means ± SD. Significance of different genotypes were

denoted as follows: ns > 0.05, *P < 0.05, **P < 0.01, ***P < 0.001, ****P < 0.0001.

(D) Quantification of larval pupariation rate within discs or tumors overexpressing the active form of *Notch* ($Notch^{act}$) alongside corresponding tumor suppressors. n represents the total number of larvae/pupae observed.

(E) Quantification of larval pupariation rate within discs or tumors overexpressing the active form of *Yki* (Yki^{S168A}) alongside corresponding tumor suppressors. n represents the total number of larvae/pupae observed.

(F) The quantifications of overall disc size within discs or tumors overexpressing the active form of *Notch* ($Notch^{act}$) along with corresponding tumor suppressors. Statistical analysis for each group was performed using one-way ANOVA with Dunnett's multiple comparisons test. Data are presented as mean ± SD (n > 10). Significance levels are denoted as follows: ns > 0.05, *P < 0.05, **P < 0.01, ***P < 0.001, ****P < 0.0001.

(G) The quantifications of overall disc size within discs or tumors overexpressing the active form of *Yki* (Yki^{S168A}) along with corresponding tumor suppressors. Statistical analysis for each group was performed using one-way ANOVA with Dunnett's multiple comparisons test. Data are presented as mean ± SD (n > 10). Significance levels are denoted as follows: ns > 0.05, *P < 0.05, **P < 0.01, ***P < 0.001, ****P < 0.0001.

Appendix Fig S9.

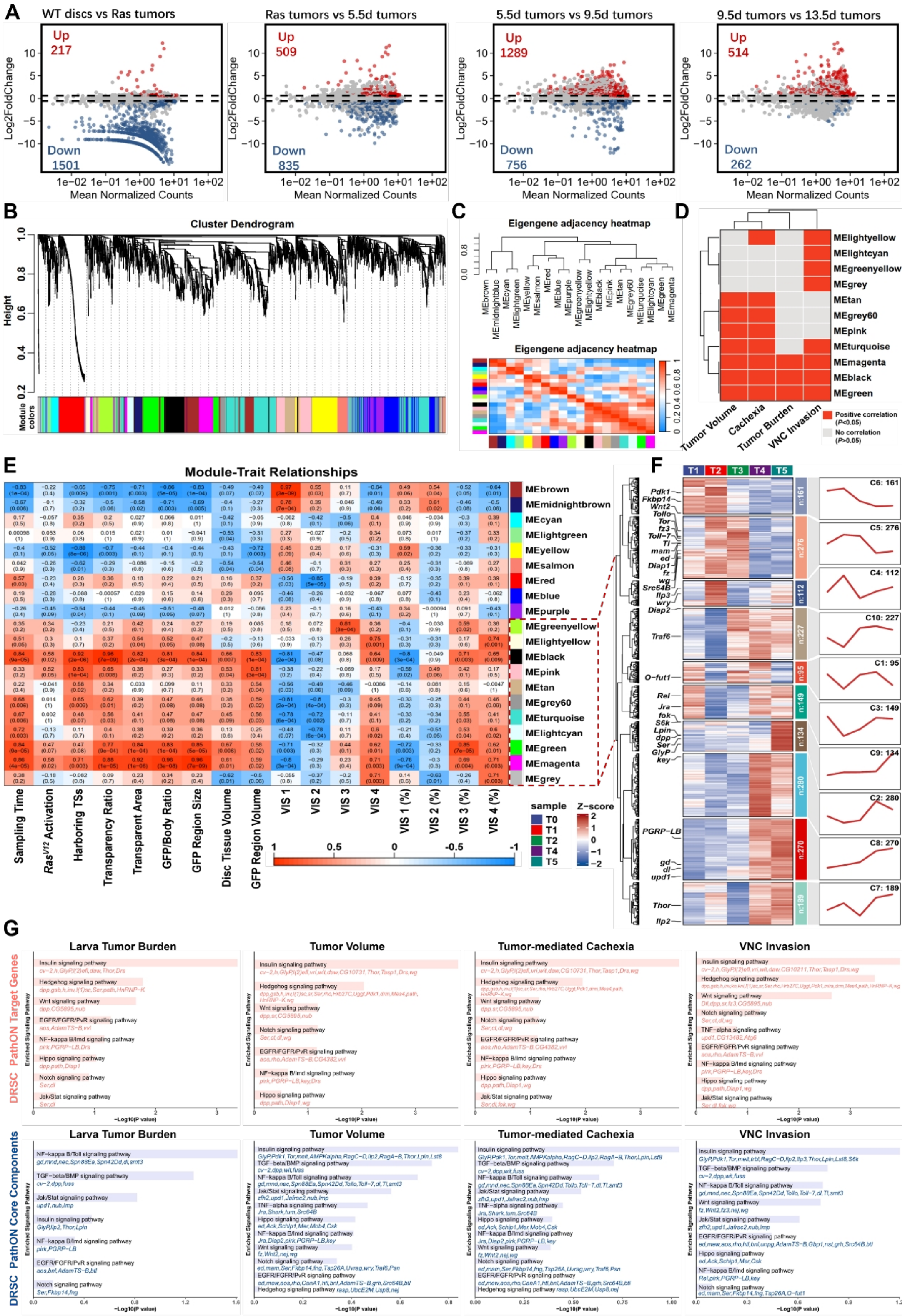

Appendix figure S9. Data characteristics and signaling pathways identified as responsible for phenotypes during *Ras*^{V12}*; fry*$^{-/-}$ tumor progression.

(A) The MA plots showing the DEGs of *Ras*^{V12}*; fry*$^{-/-}$ tumors at various stages.

(B) The cluster dendrogram of different modules and the distribution of genes within specific

modules.

(C) The correlation between different modules identified by WGCNA.

(D) The heatmap depicting the correlations between selected modules and four aspects of phenotypic malignancies. The relationships were revealed by using Pearson correlation test, $P < 0.05$ were shown in red.

(E) The WGCNA heatmap depicting the correlations between identified gene modules and phenotypic malignancies. The red dashed box highlighted the selected modules for further analysis. The color intensity of each module represented the correlation coefficient, which was also displayed in the top line of the respective cell. The corresponding *P*-value was shown in parentheses, with *P* values below 0.05 indicating statistically significant correlations.

(F) The heatmap depicting the expression profiles of the K-means clustering results. A total of 10 clusters were identified, with key genes from the JNK, Notch, Hippo, Toll, and Insulin signaling pathways denoted on the left. The dynamic gene expression patterns of each cluster were outlined on the right.

(G) The bar plots showing the enrichment of signaling pathways responsible for different phenotypes. Enrichment analysis of modules significantly correlated with the phenotype was performed by using PANGEA. The bar plots display the enriched terms and their corresponding genes from two databases: DRSC PathON target genes (red) and DRSC PathON core components (blue).

Appendix Fig S10.

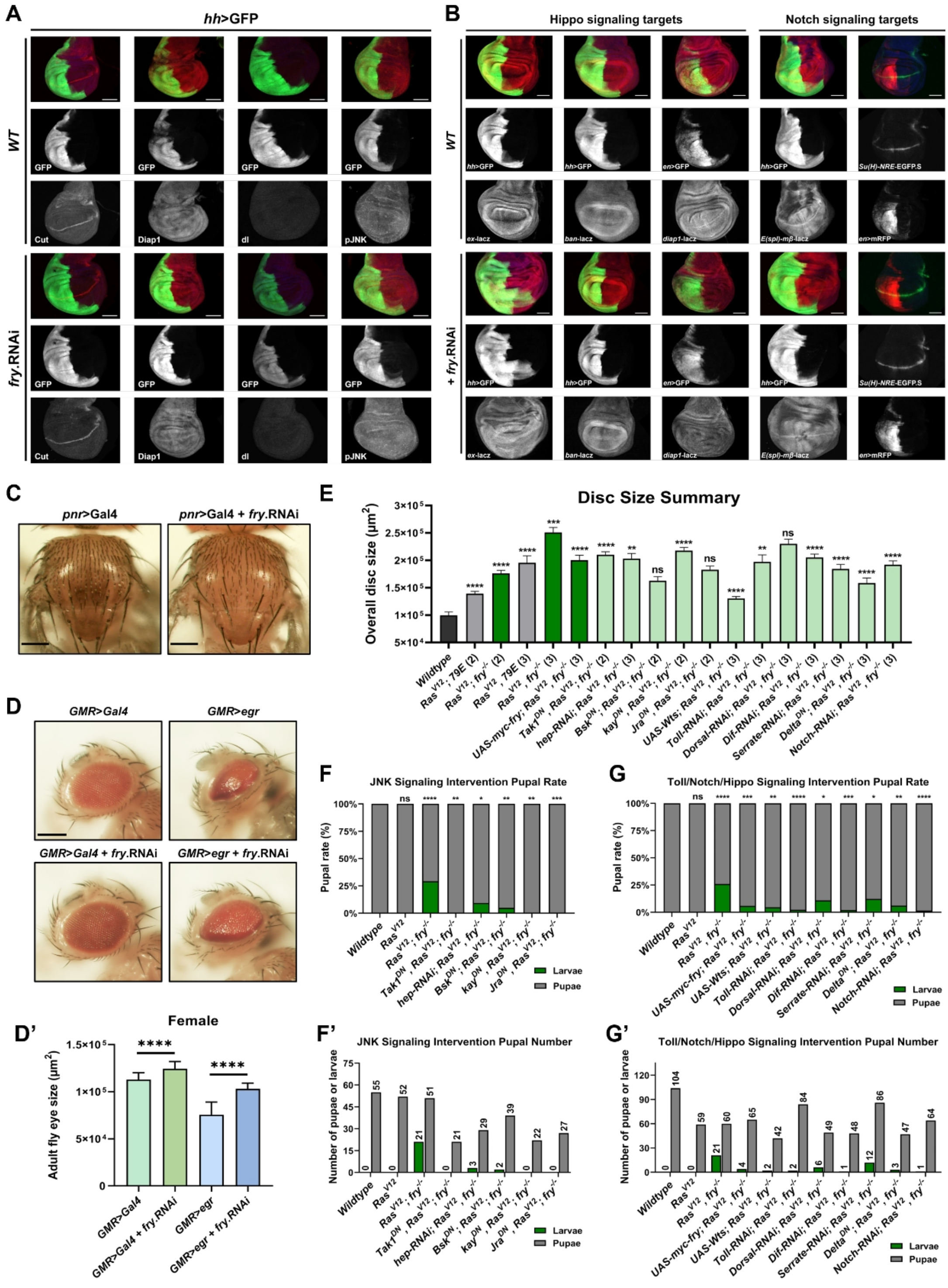

Appendix figure S10. Evaluation of physiological and pathological functions of *fry*.

(A) The expression levels of target genes or key kinases in the JNK, Hippo, NF-κB/Toll, and Notch signaling pathways were examined following *fry* gene knockdown under physiological conditions. Scale bar: 100 μm.

(B) Knockdown of the *fry* gene under physiological conditions to examine the expression levels of target genes associated with the Hippo and Notch signaling pathways. Scale bar: 100 μm.

(C) Investigation of the effects of altered *fry* gene expression levels on dorsal closure in *Drosophila*. Scale bars: 200 µm.
(D-D') The light micrographs (D) and quantification results (D') of adult eyes (Female) with the indicated genotypes. Scale bars: 200 µm. Statistical analysis for each group was performed using the Mann-Whitney U-test or Student's t-test based on the variance. Results were presented as means ± SD, n >25. Significance of different genotypes were denoted as follows: ns > 0.05, **P* < 0.05, ***P* < 0.01, ****P* < 0.001, *****P* < 0.0001.
(E) Quantification of overall eye-antennal disc size across different genotypes. Statistical significance was assessed using either the Mann–Whitney U-test or Student's t-test, depending on variance homogeneity (n > 25). Experimental groups were compared against the corresponding Ras control group. Data are presented as means ± SEM. Significance levels are denoted as follows: ns > 0.05, **P* < 0.05, ***P* < 0.01, ****P* < 0.001, *****P* < 0.0001.
(F-F') Quantification of larval pupariation rates following JNK signaling inhibition in Ras^{V12}*; fry*$^{-/-}$ tumors. Statistical analyses of pupal rate (F) and pupal number (F') were presented. Chi-square tests were performed to compare groups. Significance levels were indicated as follows: ns > 0.05, **P* < 0.05, ***P* < 0.01, ****P* < 0.001, *****P* < 0.0001.
(G-G') Quantification of larval pupariation rates following NF-κB/Toll signaling inhibition, Hippo signaling inactivation, and Notch signaling inhibition in Ras^{V12}*; fry*$^{-/-}$ tumors. Statistical analyses of pupal rate (G) and pupal number (G') were presented. Chi-square tests were performed to compare groups. Significance levels were indicated as follows: ns > 0.05, **P* < 0.05, ***P* < 0.01, ****P* < 0.001, *****P* < 0.0001.

Appendix Fig S11.

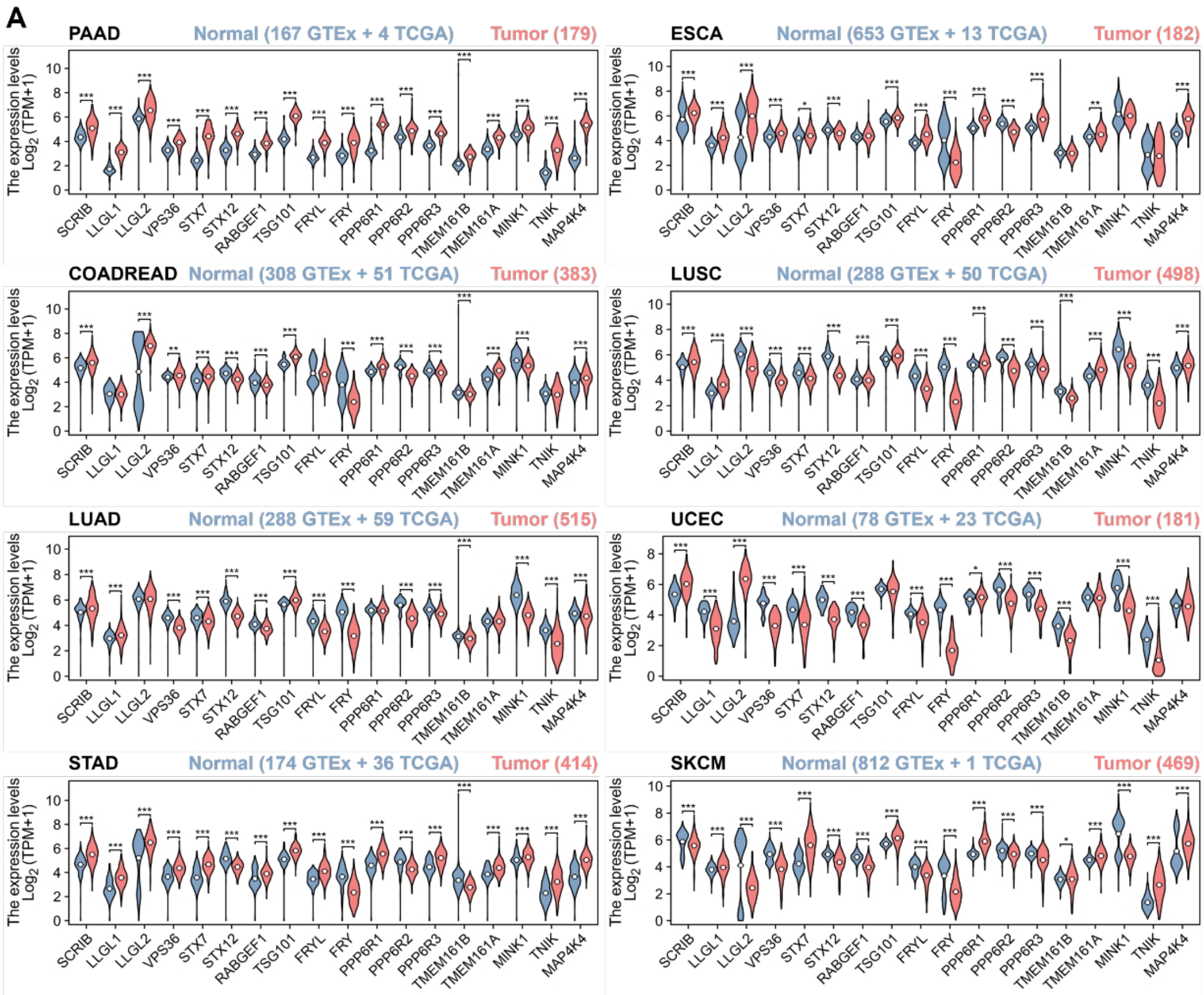

Appendix figure S11. Expression levels of human orthologs across different human *KRAS/NRAS*-driven cancers.

(A) The expression levels of eighteen human orthologs in eight cancers with high mutation rates of *RAS* family genes. UCS and THCA were excluded due to the limit number of samples and low gene mutation rates of human orthologs, respectively.

Appendix Fig S12.

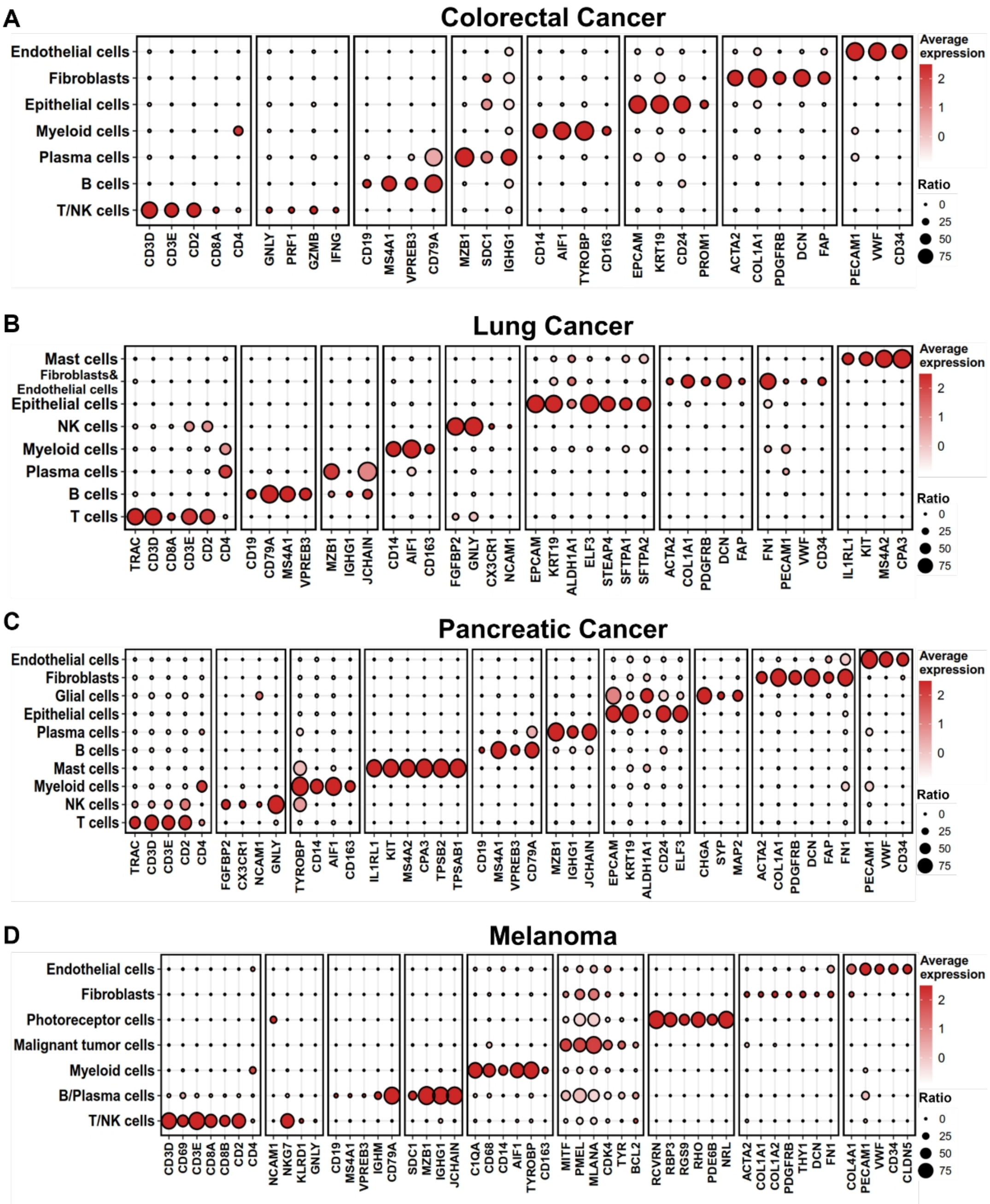

Appendix figure S12. Annotation of cell types in different human *KRAS/NRAS*-driven cancers.

(A-D) The dot plots depicting the cell type annotation across *KRAS*/*NRAS*-driven cancers. The cell types of maintained high-quality cells were annotated based on the canonical cell markers across *KRAS*/*NRAS*-driven cancers, CRC (A), LUAD (B), PAAD (C), and melanoma (D). Cell types included T cells, NK cells, B cells, myeloid cells, plasma cells, mast cells, epithelial cells, endothelial cells, fibroblasts, glial cells, and photoreceptor cells (only in SKCM).

Appendix Fig S13.

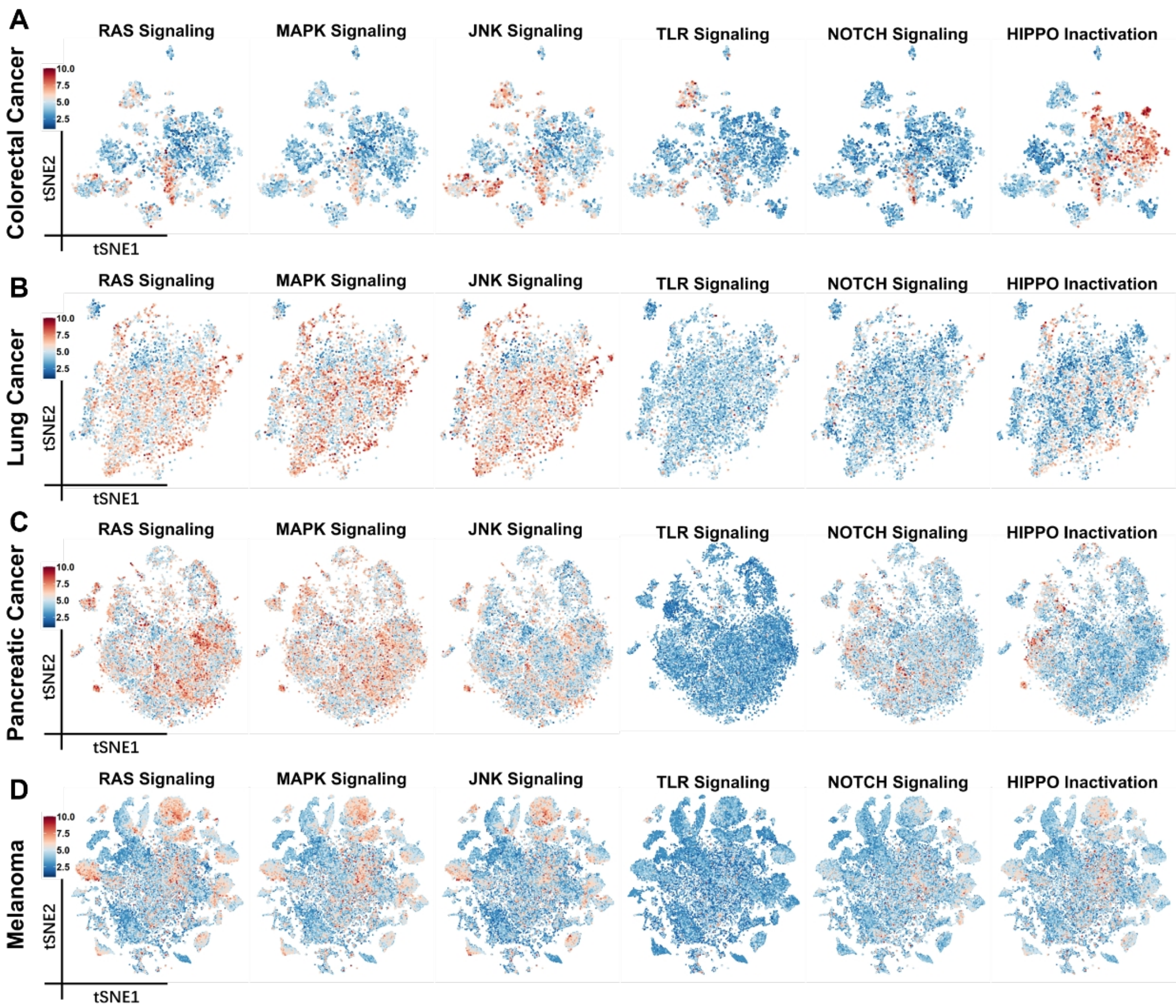

Appendix figure S13. Scoring of evolutionarily conserved oncogenic signaling pathways in human *KRAS/NRAS-driven* cancers.

(A-D) The tSNE representations depicting the scoring of six signaling pathway activity in re-clustered tumor epithelial cells. The human *KRAS*/*NRAS*-driven cancers included CRC (A), LUAD (B), PAAD (C), and melanoma (D). The evaluation of six signaling pathways encompassed the activation of RAS, MAPK, JNK, TLR, Notch and the inactivation of Hippo. The scoring values were scaled from 0 to 10.

Appendix Fig S14.

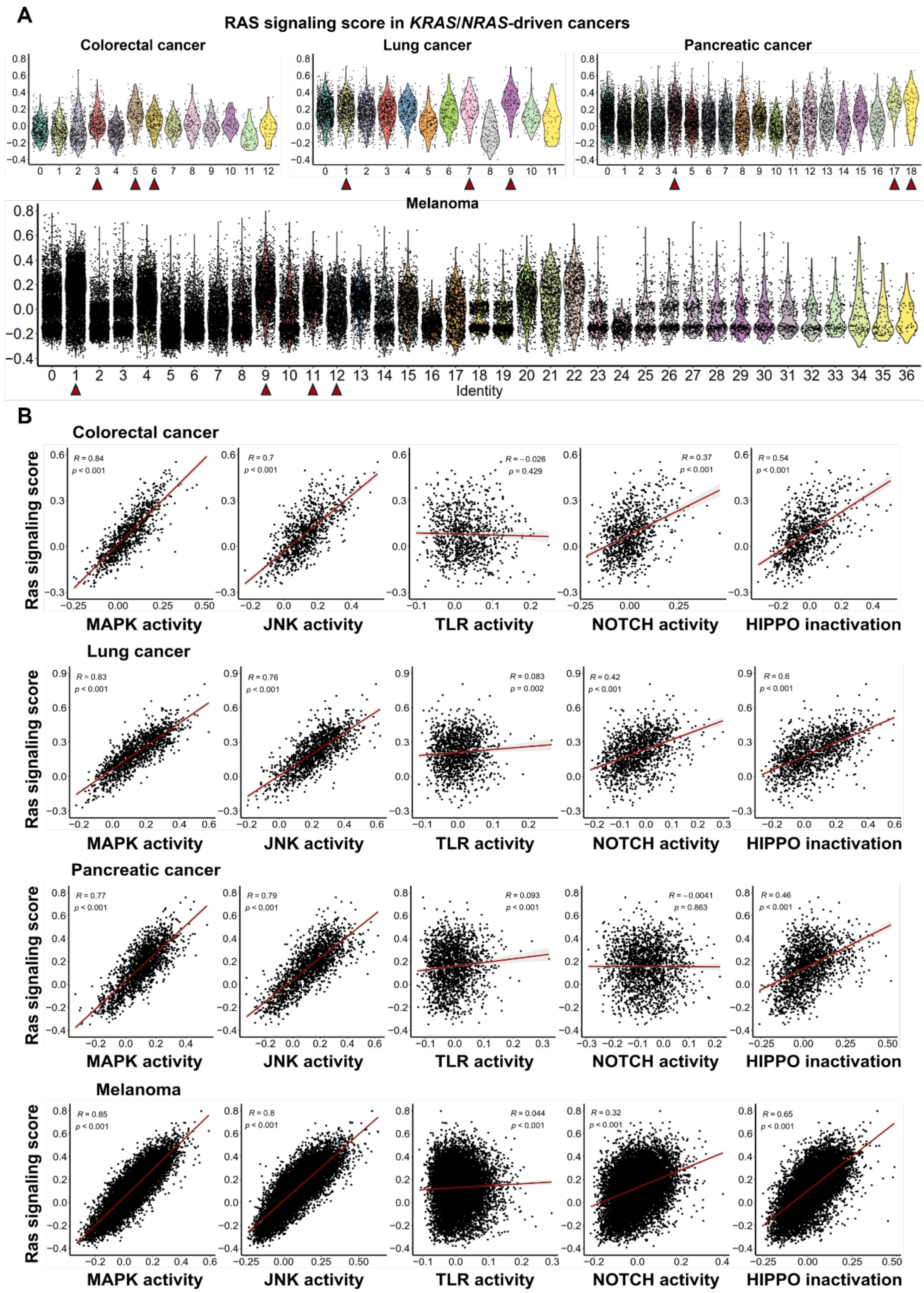

Appendix figure S14. Correlations of evolutionarily conserved oncogenic signaling pathways in human *KRAS/NRAS-driven* cancers.

(A) The violin plots depicting the average score of RAS signaling pathway across different cancer epithelial cell clusters. Based on the median expression, cell clusters with top-ranking RAS signaling scores were acknowledged as *RAS*-activated cells and were denoted by red arrowheads.

(B) The correlation scatter plots illustrate the relationships between RAS signaling pathway scores and those of the other five signaling pathways in *RAS*-activated cells. These correlations were determined using Spearman's correlation analysis, with coefficients R and *P*-values reported, where $P < 0.05$ indicates statistical significance.

Appendix Fig S15.

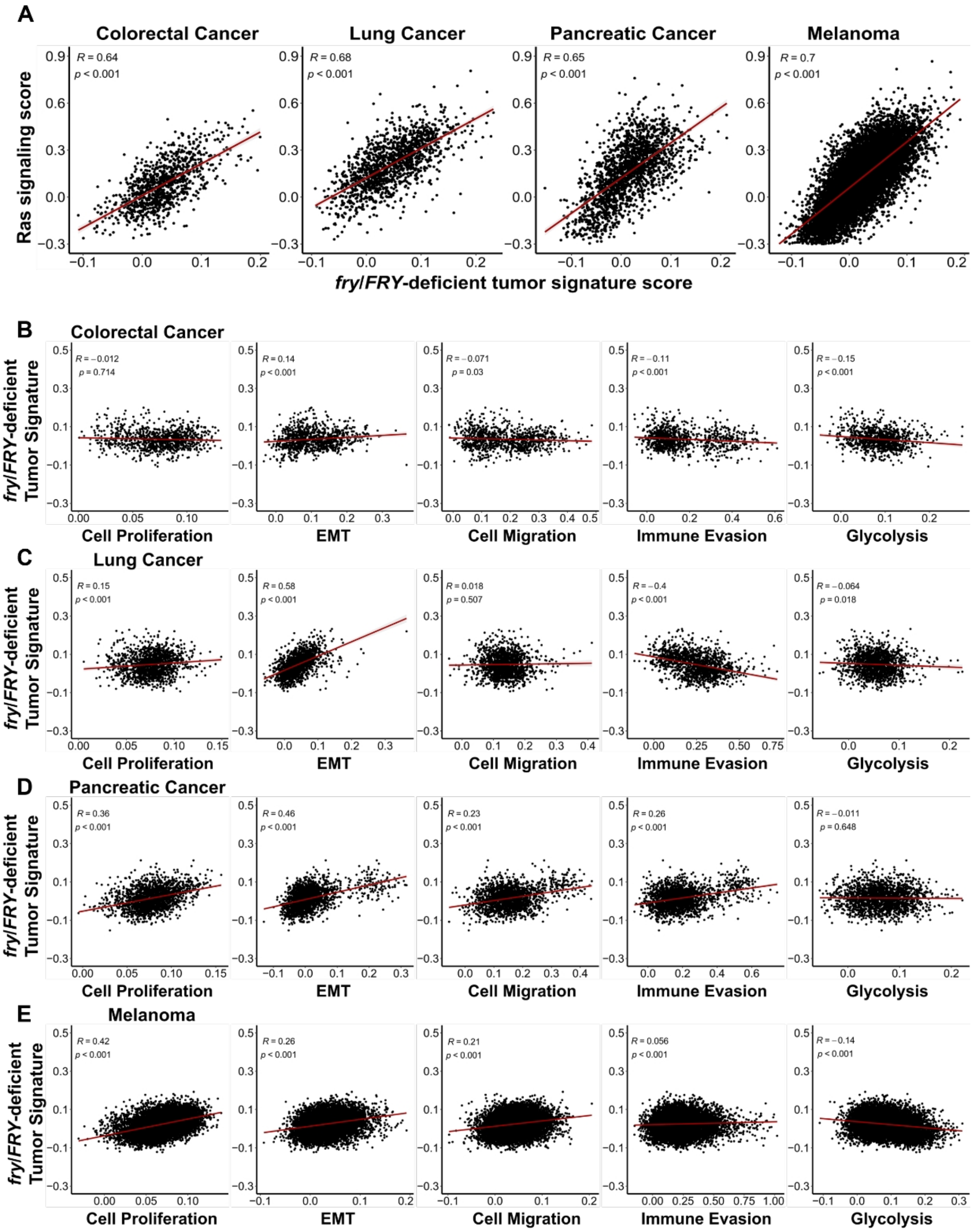

Appendix figure S15. Correlations of *fry*/*FRY*-deficient tumor signature and tumor malignancies in *RAS*-activated cells of human *KRAS/NRAS-driven* cancers.

(A) The correlation scatter plots illustrate the relationships between RAS signaling scores and *fry*/*FRY*-deficient tumor signature scores across various cancers. These correlations were determined using Spearman's correlation analysis, with coefficients R and *P* values reported, where $P < 0.05$ indicates statistical significance.

(B-E) The correlation scatter plots depicting the relationships between *fry*/*FRY*-deficient tumor signature scores and tumor malignancies scores in *RAS*-activated cells across *KRAS*/*NRAS*-driven cancers, CRC (B), LUAD (C), PAAD (D), and melanoma (E). These correlations were determined

using Spearman's correlation analysis, with coefficients R and P values reported, where $P < 0.05$ indicates statistical significance.

Appendix Fig S16.

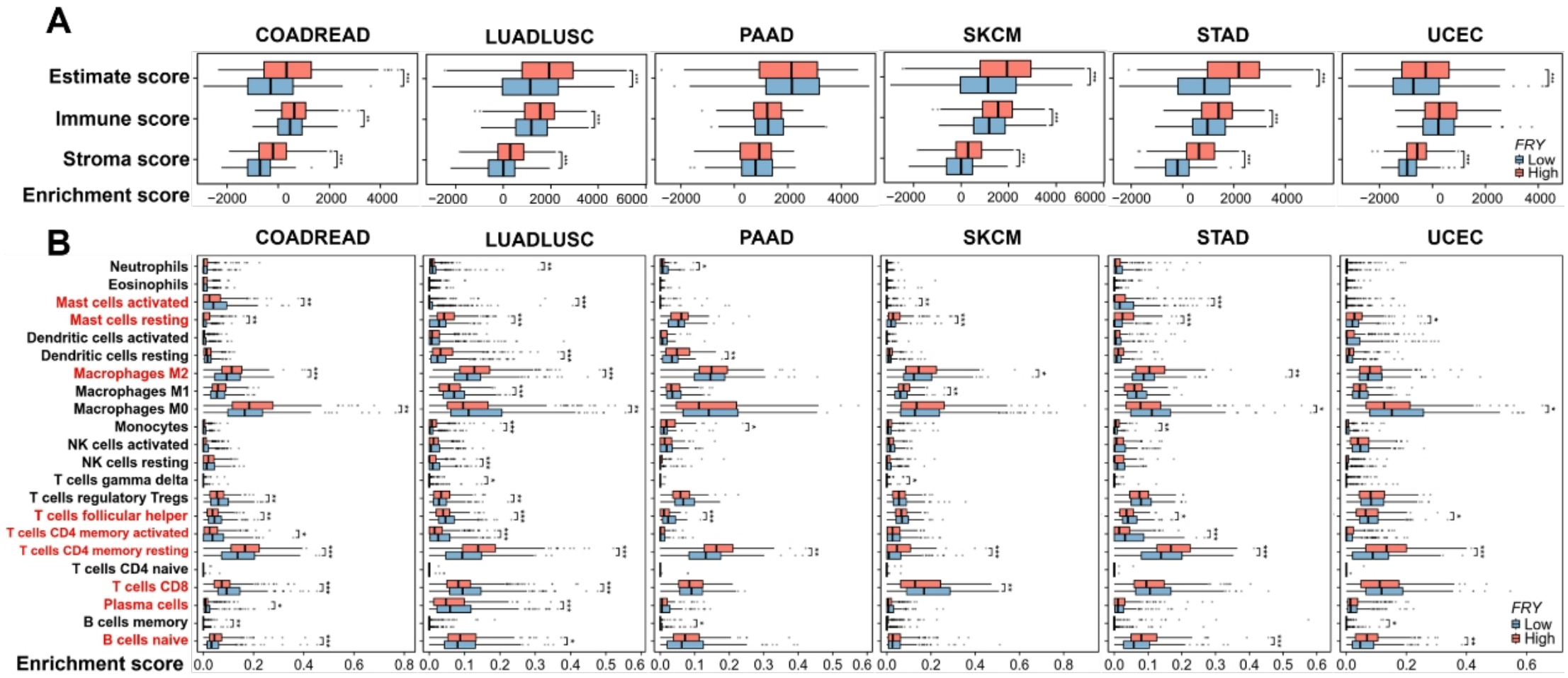

Appendix figure S16. Effects of *FRY* in immune infiltration across different human *KRAS/NRAS-driven* cancers.

(A) The estimate score, immune score, and stroma score within various *KRAS*/*NRAS*-driven cancers via ESTIMATE. Tumor samples obtained from TCGA were divided into two groups based on the medial expression level of *fry* ortholog gene *FRY*. The enrichment scores between two groups were calculated via Wilcoxon rank sum test, and the significance was denoted as *$P < 0.05$, **$P < 0.01$, ***$P < 0.001$, ****$P < 0.0001$.

(B) The estimated infiltration of various immune cells within various *KRAS*/*NRAS*-driven cancers via CIBERSORT. Tumor samples obtained from TCGA were divided into two groups based on the medial expression level of *fry* ortholog gene *FRY*. The CIBERSORT enrichment scores between two groups were calculated via Wilcoxon rank sum test, and the significance was denoted as *$P < 0.05$, **$P < 0.01$, ***$P < 0.001$, ****$P < 0.0001$.

Appendix Fig S17.

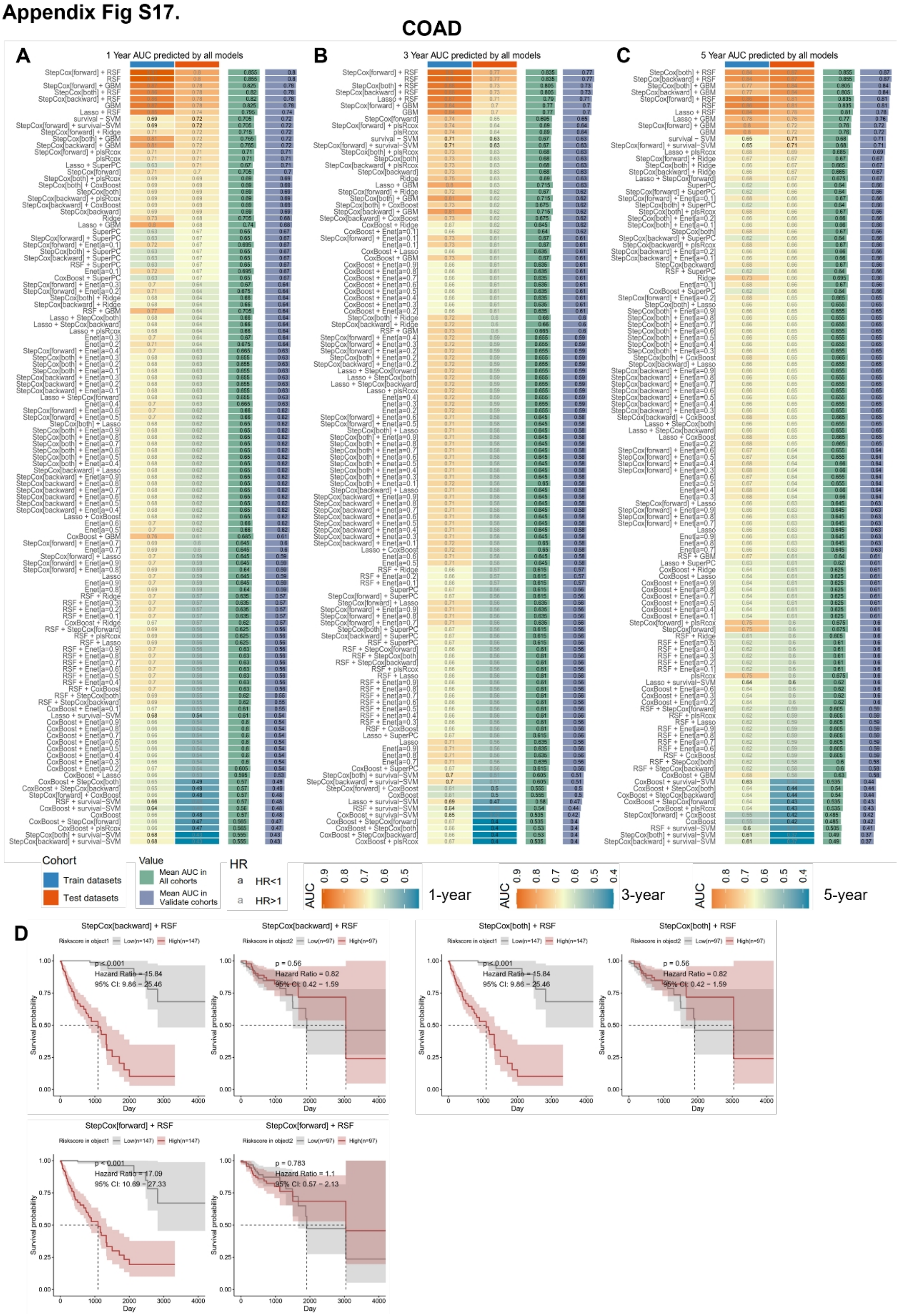

Appendix figure S17. The predictive value of *Drosophila Ras*-driven tumors examined by 117 machine learning algorithms in COAD.

(A-C) The applications of 117 machine learning algorithms to establish predictive models of COAD patient survival. The 1-year AUC (A), 3-year AUC (B), and 5-year AUC (C) of each model in both training dataset and test dataset were shown in left, while the mean AUC in all cohorts and validate

cohorts were shown in right.

(D) The Kaplan-Meier plots illustrated certain models' performance in predicting the overall survival of COAD patients. Patients were separated based on the median value of risk scores calculated by selected model. The object 1 and object 2 represent the training dataset and test dataset, respectively. The *P* value, hazard ratio, and 95% confidence interval (CI) of each model were shown. *P* values less than 0.05 were considered significant.

Appendix Fig S18.

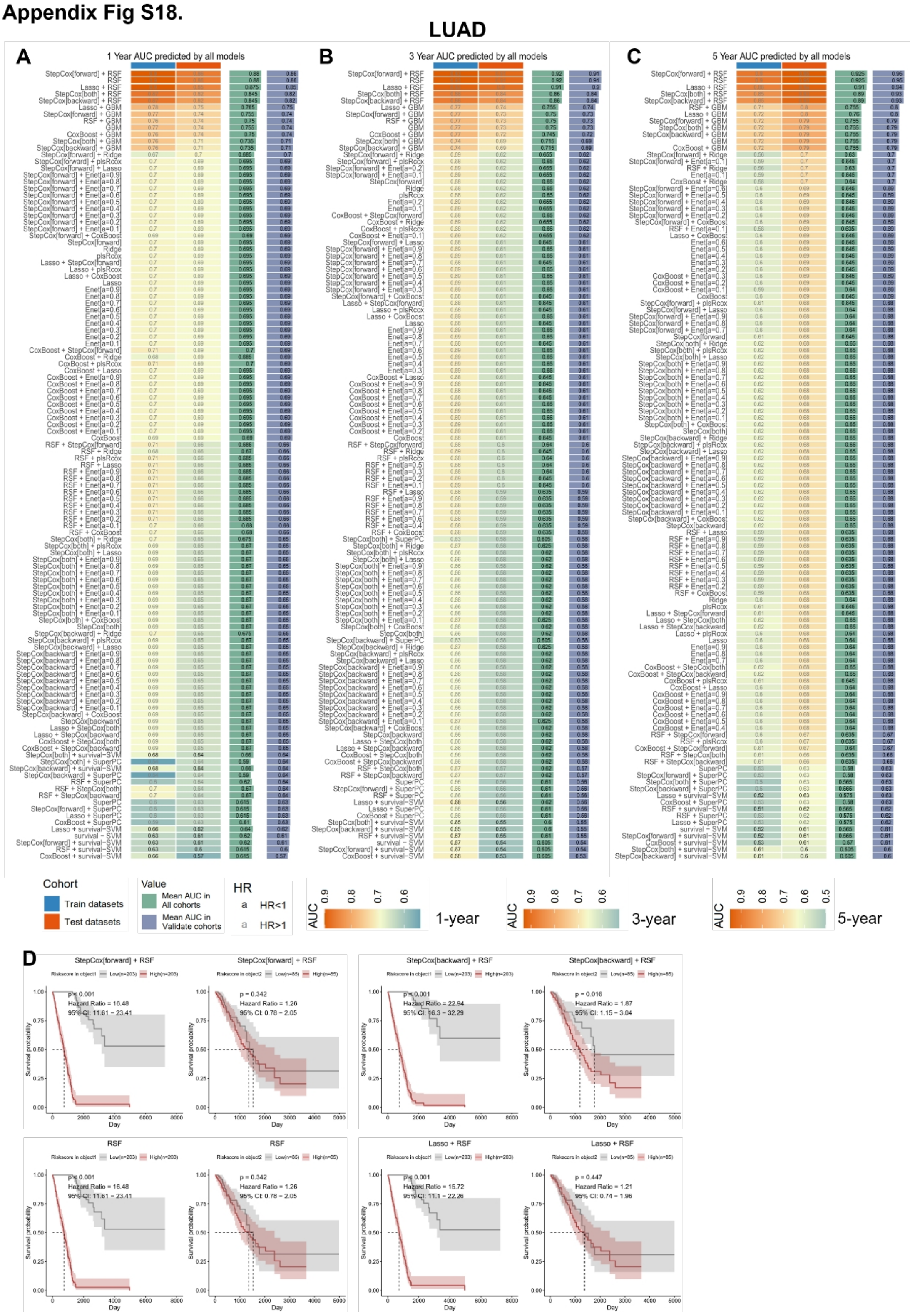

Appendix figure S18. The predictive value of *Drosophila Ras*-driven tumors examined by 117 machine learning algorithms in LUAD.

(A-C) The applications of 117 machine learning algorithms to establish predictive models of LUAD patient survival. The 1-year AUC (A), 3-year AUC (B), and 5-year AUC (C) of each model in both training dataset and test dataset were shown in left, while the mean AUC in all cohorts and validate

cohorts were shown in right.

(D) The Kaplan-Meier plots illustrated certain models' performance in predicting the overall survival of LUAD patients. Patients were separated based on the median value of risk scores calculated by selected model. The object 1 and object 2 represent the training dataset and test dataset, respectively. The *P* value, hazard ratio, and 95% confidence interval (CI) of each model were shown. *P* values less than 0.05 were considered significant.

Appendix Fig S19.

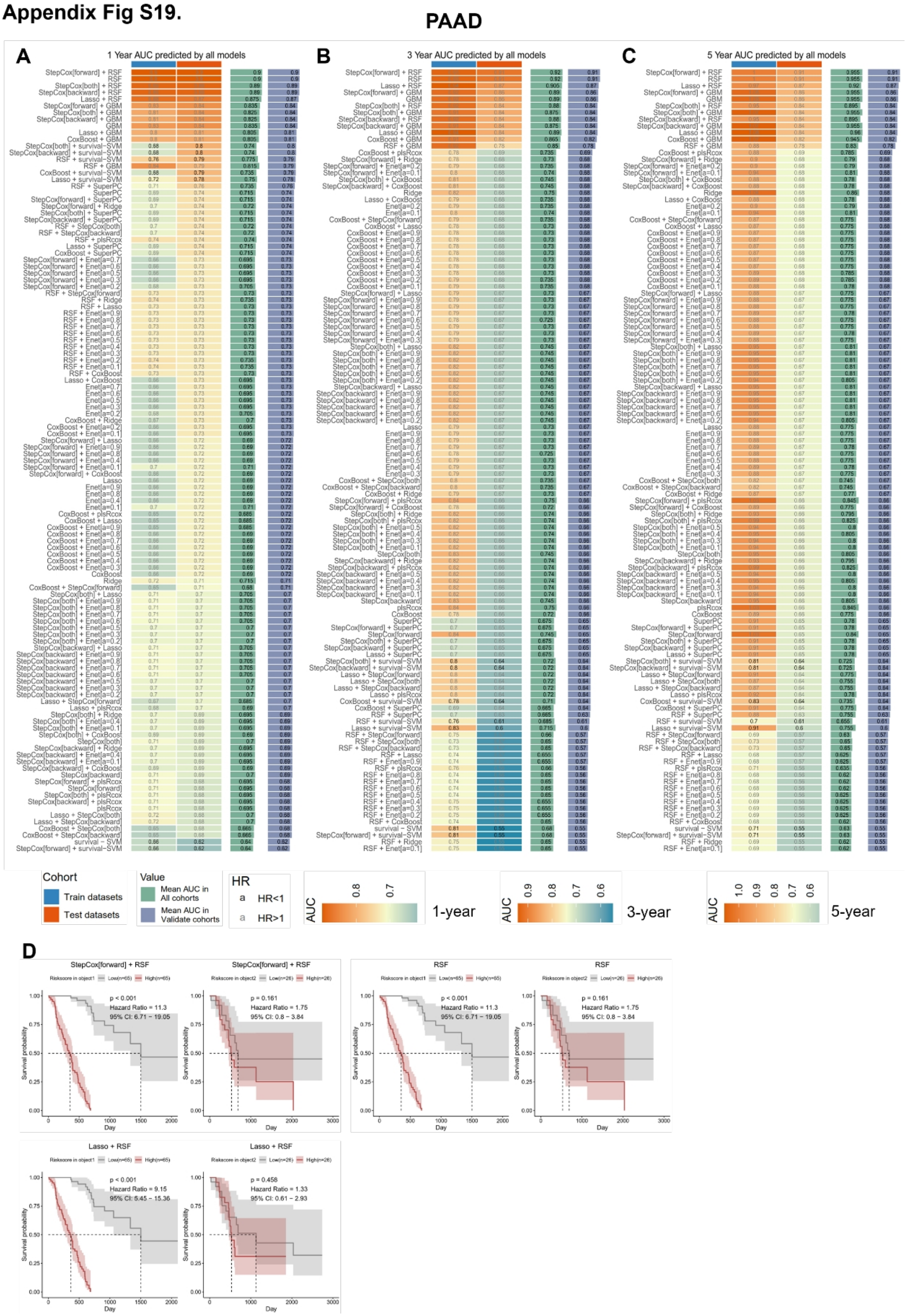

Appendix figure S19. The predictive value of *Drosophila Ras*-driven tumors examined by 117 machine learning algorithms in PAAD.

(A-C) The applications of 117 machine learning algorithms to establish predictive models of PAAD patient survival. The 1-year AUC (A), 3-year AUC (B), and 5-year AUC (C) of each model in both training dataset and test dataset were shown in left, while the mean AUC in all cohorts and validate

cohorts were shown in right. The 5-year AUC (C) showed AUC higher than 1 in top-ranking machine learning algorithms related to GBM, these algorithms combinations were excluded.
(D) The Kaplan-Meier plots illustrated certain models' performance in predicting the overall survival of PAAD patients. Patients were separated based on the median value of risk scores calculated by selected model. The object 1 and object 2 represent the training dataset and test dataset, respectively. The *P* value, hazard ratio, and 95% confidence interval (CI) of each model were shown. *P* values less than 0.05 were considered significant.

Appendix Fig S20.

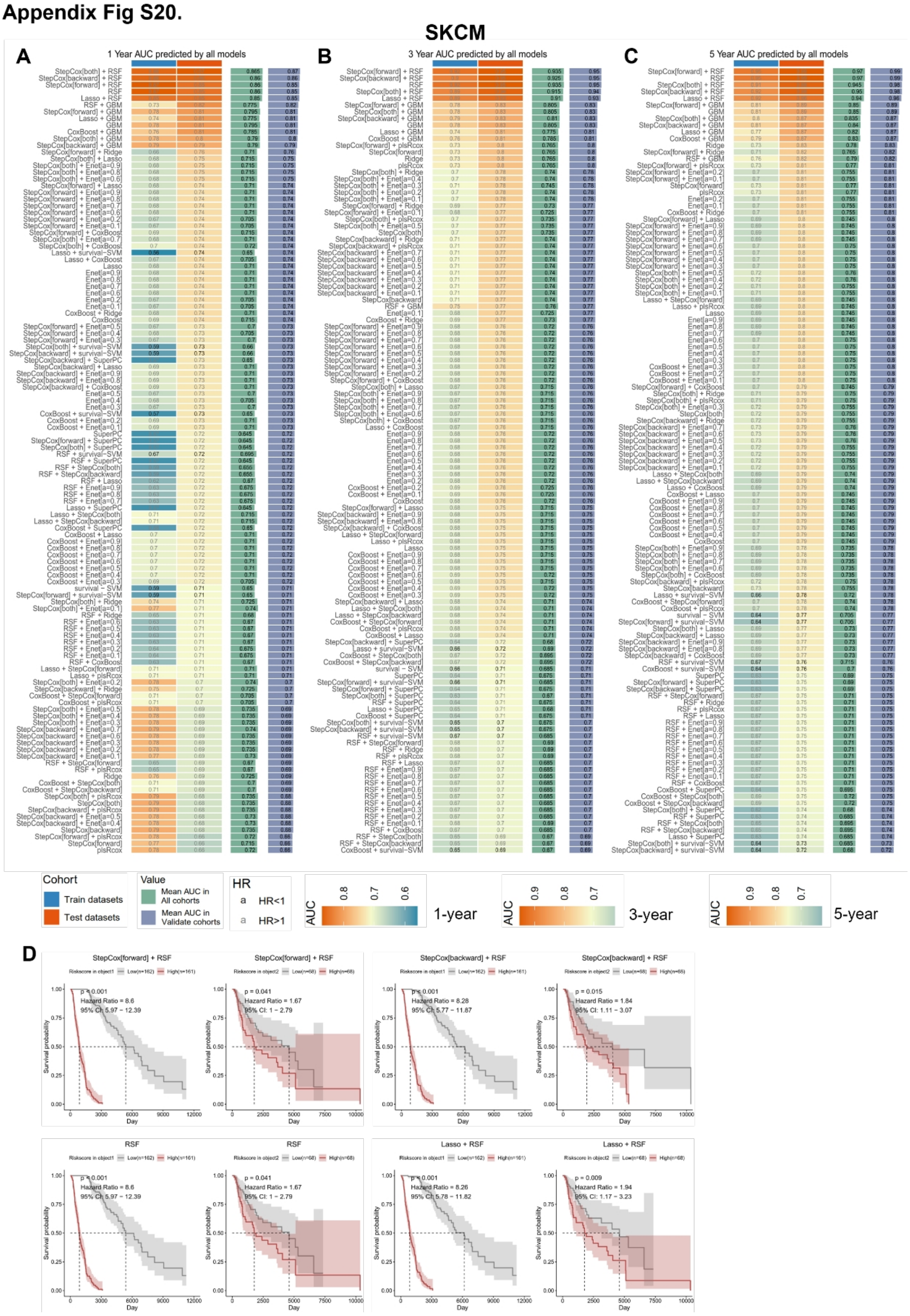

Appendix figure S20. The predictive value of *Drosophila Ras*-driven tumors examined by 117 machine learning algorithms in SKCM.

(A-C) The applications of 117 machine learning algorithms to establish predictive models of SKCM patient survival. The 1-year AUC (A), 3-year AUC (B), and 5-year AUC (C) of each model in both training dataset and test dataset were shown in left, while the mean AUC in all cohorts and validate

cohorts were shown in right.

(D) The Kaplan-Meier plots illustrated certain models' performance in predicting the overall survival of SKCM patients. Patients were separated based on the median value of risk scores calculated by selected model. The object 1 and object 2 represent the training dataset and test dataset, respectively. The *P* value, hazard ratio, and 95% confidence interval (CI) of each model were shown. *P* values less than 0.05 were considered significant.
